# Safety and immunogenicity of UB-612 heterologous booster in adults primed with mRNA, adenovirus, or inactivated COVID-19 vaccines: a randomized, active-controlled, Phase 3 trial

**DOI:** 10.1016/j.eclinm.2025.103349

**Published:** 2025-07-21

**Authors:** Alexander Rumyantsev, Lixia Wang, Shixia Wang, Tracy Kemp, Alana Wriggins, Amy Burks, Danielle Fisher, Katie Brokke, Amy Fix, Susan Hensley, Maggie Lewis, Ray Zhu, Kate Wang, Carolyn Shasha, Giulia Piccini, Alessandro Manenti, Emanuele Montomoli, Rodrigo DeAntonio, Xavier Saez-Llorens, Milagros Chan, Edison Alberto, Ma Dovie Lallaine Borra, Anjuli May Jaen, Gray Heppner, Ulo Palm, Thomas P. Monath

**Affiliations:** a Vaxxinity, Inc., FL, USA; b AvanSight, Inc., CA, USA; c VisMederi srl, Siena, Italy; d Cevaxin, Panama City, Panama; e Health Index Multispecialty Clinic, Bacoor, Philippines; f St. Pauls Hospital, Iloilo, Philippines; g Iloilo Doctor's Hospital, Iloilo, Philippines

**Keywords:** COVID-19, Vaccine, Subunit, RBD, Adjuvant, Clinical trial

## Abstract

**Background:**

Authorized COVID-19 vaccines require boosters for continued protection; however, the lack of cross-platform compatible boosters creates practical challenges to keeping populations protected.

**Methods:**

This Phase 3, multicenter, international, randomized, active-controlled trial compared UB-612 as a third-dose heterologous booster to BNT162b2, ChAdOx1-S, or BBIBP-CorV homologous boosters in healthy subjects aged ≥16 years. Participants were randomly assigned 1:1 to receive a single intramuscular injection of UB-612 or an active comparator matching the primary dose, and were stratified for age, sex, N-protein seropositivity, and time since the last dose of their primary series COVID-19 vaccination. The primary objective was to show non-inferiority of neutralizing antibody geometric mean titer (GMT) against live SARS-CoV-2 Wuhan strain after boosting with UB-612 or each of the licensed platform vaccines. Secondary and exploratory objectives covered short and long-term antibody responses. The safety analysis addressed subject and investigator reported adverse events. The study was registered on ClinicalTrials.gov, NCT05293665, and completed on September 12, 2023.

**Findings:**

Between March 22 and September 9, 2022, 469 subjects received UB-612 as a heterologous booster, and 467 received BNT162b2 (n = 204), ChAdOx1-S (n = 95), or BBIBP-CorV (n = 168) as homologous boosters. Over 90% of all subjects were positive for N-protein antibody at baseline. When compared to the respective homologous booster response, UB-612 stimulated Wuhan and Omicron BA.5 neutralizing antibody responses that were non-inferior, thus meeting all primary and secondary immunogenicity endpoints of the study. Importantly, UB-612 demonstrated superiority in neutralizing antibody GMT and seroresponse rates compared to ChAdOx1-S and BBIBP-CorV. UB-612 was also effective in stimulating neutralizing antibodies against a more recent Omicron XBB1.5 strain. Long-term immunity analysis through 6- and 12-month follow-ups favored UB-612 over ChAdOx1-S and BBIBP-CorV and supported comparable immunity to BNT162b2. All vaccines were well tolerated and had similar safety profiles.

**Interpretation:**

In a pivotal Phase 3 study, UB-612 demonstrated the potential for broad use as a cross-platform heterologous booster, restoring protective immunity in adults previously vaccinated with mRNA, adenovirus-vectored, or inactivated virus-based COVID-19 vaccines.

**Funding:**

The study was co-funded by the Coalition for Epidemic Preparedness Innovations (CEPI) and Vaxxinity.

**Research in context**

**Evidence before this study**

Since the 2020 SARS-CoV-2 outbreak, approximately 70% of the global population has been vaccinated with COVID-19 vaccines based on mRNA, adenovirus-vectored, or inactivated virus platforms, all of which require booster doses. Subsequently approved COVID-19 vaccines introduced challenges in aligning primary and booster regimens, underscoring the need for safe, immunogenic, and interchangeable boosters. We reviewed clinical studies reported in ClinicalTrials.gov and PubMed through March 1, 2024, and found limited data supporting a heterologous booster that is broadly interchangeable across different COVID-19 vaccine platforms and characterized in diverse populations.

**Added value of this study**

This is the first report of a novel subunit heterologous booster vaccine that successfully restored protective neutralizing antibodies stimulated by the primary series major vaccine platforms, including mRNA, adenovirus vectored, and inactivated virus, used worldwide. UB-612 stimulated broadly cross-reactive neutralizing antibodies against the prototype and subsequently emerging SARS-CoV-2 strains, including Omicron BA.5 and XBB1.5. At the same time, no safety concerns were identified following administration of the heterologous UB-612 booster dose.

**Implications of all the available evidence**

The collected evidence supports the interchangeable use of UB-612 as a cross-platform universal heterologous booster to restore protective immunity in adults following the primary COVID-19 immunization with mRNA, adenovirus vectored, or inactivated virus vaccines.

## Introduction

Since the onset of the SARS-CoV-2 pandemic, over 50 vaccines have received approval, in at least one country, to prevent COVID-19.^1^ These vaccines have been instrumental in immunizing approximately 70% of the global population.^2^ The limited longevity of vaccine-induced immunity and the emergence of new SARS-CoV-2 variants have underscored the imperative for more effective vaccine strategies.^3^ Strategies to enhance protective immunity have included reformulating vaccines with contemporary SARS-CoV-2 antigens and administering booster doses, including heterologous alternative platforms.^4^^,^^5^ The uneven availability of COVID-19 vaccines globally has introduced practical challenges in aligning booster doses with the original vaccine used for primary vaccination. Some heterologous vaccine combinations have demonstrated improvements in immunity, while others have not, with variations in reactogenicity.^6^^,^^7^

As of November 2023, over 16.5 billion doses of SARS-CoV-2 vaccines have been delivered globally, with the most common platforms being mRNA (Pfizer/BioNTech and Moderna), adenovirus-vectored (AstraZeneca and Janssen), and inactivated virus (Sinopharm and Sinovac).^8^ An ideal booster should effectively restore protective immunity established by these common vaccine platforms. However, novel COVID-19 vaccines have limited clinical experience as boosters in the population with diverse immunity against SARS-CoV-2.^9^

Vaxxinity has developed a subunit vaccine, UB-612, designed to activate both humoral and cellular responses. UB-612 features a recombinant RBD fused to a single-chain Fc fragment of human immunoglobulin (IgG1) formulated with synthetic peptides carrying T-helper and cytotoxic T lymphocyte epitopes selected from M, S2, and N proteins of the virus. Its composition includes key antigenic determinants present in authorized COVID-19 vaccines, making it a potential candidate for restoring protective antibodies generated by primary immunization.^10^

In collaboration with UBI Asia, which conducted Phase 1 and Phase 2 clinical trials in Taiwan, UB-612 underwent evaluation for safety and immunogenicity in over 3700 subjects 12 years and older.^10^ The primary two-dose series elicited neutralizing antibodies equivalent to those in convalescent sera from hospitalized COVID-19 patients. A homologous booster stimulated a significant increase in neutralizing antibodies, surpassing peak levels achieved after the primary series by over 9-fold. Importantly, the antibodies demonstrated broad activity against multiple SARS-CoV-2 variants, including Omicron strains, surpassing levels reported after primary immunization with authorized COVID-19 vaccines.^11^^,^^12^ The current study co-funded by CEPI compared the safety and immunogenicity of UB-612 head-to-head against three COVID-19 vaccines, mRNA (BNT162b2, Pfizer/BioNTech), adenovirus-vectored (ChAdOx1-S, AstraZeneca), and inactivated virus (BBIBP-CorV, Sinopharm), delivered as heterologous or homologous third-dose boosters in adults.

## Methods

### Trial design

This multicenter, international, randomized, active-controlled, non-inferiority trial was designed as a platform of three substudies with the common objectives of comparing the safety and immunogenicity of a heterologous UB-612 third-dose booster to homologous booster comparators (BNT162b2, ChAdOx1-S, or BBIBP-CorV). The trial was conducted at seven clinical sites internationally, including one in the USA (PanAmerican Clinical Research, Brownsville, TX), three in Panama (Cevaxin: Centro de Vacunación e Investigación, Panama City; Cevaxin 24 de Diciembre, Panama City; Cevaxin Chorrera, La Chorrera), and three the Philippines (Health Index Multispecialty Clinic, Bacoor; St. Pauls Hospital, loilo; Iloilo Doctor's Hospital, loilo). In the substudy evaluating UB-612 and BNT162b2, open-label and double-blind protocols were implemented to facilitate access to the comparator vaccine at one of the clinical sites in the Philippines; the other substudies were double-blind. The trial complied with the Declaration of Helsinki and Good Clinical Practices (GCP); the protocols were approved by the Institutional Review Boards (IRB)/Research Ethics Committees (REC) as dictated by local regulations.

### Participants

Eligible participants were 16 years of age or older, who had completed two-dose primary COVID-19 vaccine series with the last dose administered at least 5 months prior to enrollment for BNT162b2 or 3 months prior to enrollment for BBIBP-CorV and ChAdOx1-S. The main exclusion criteria were a known history of COVID-19 within 6 months, or receipt of a COVID-19 booster within the windows described above. The full list of exclusions is described in the protocol (Appendix 1, p. 35). Subjects provided written informed consent, and assent for underaged individuals, agreeing to the potential risks and benefits, and study requirements, including contraception.

### Randomization and masking

Subjects were randomized 1:1 using an Interactive Response Technology System into the two treatment groups for each respective substudy, stratified by age group (16–64 vs ≥65 years), sex at birth, nucleocapsid (N) protein seropositivity at baseline, and the time since last dose of primary immunization (≥3–≤5 months or >5 months) to receive either a single injection with UB-612 or a homologous comparator COVID-19 booster. For double-blind substudies, the sponsor study team, subjects, the investigator, study staff with subject contact, and laboratory testing personnel performing assays were blinded to the treatment assignment for the duration of the study. In the open-label portion of the BNT162b2 substudy, the treatment assignments were known to investigator, study staff, and subjects at one site in the Philippines, while the sponsor and laboratory teams remained blinded to minimize the potential bias.

### Procedures

Subjects were screened for N-protein antibodies by SARS-CoV-2 IgG/IgM antibody test kit (Biohit Healthcare, China). On day 1 subjects received a single intramuscular injection with a study vaccine. UB-612 is a subunit vaccine containing 100 μg of antigen (88 μg of RBD protein and 12 μg synthetic peptides combined) as a sterile Adju-Phos® (Croda, UK) adsorbed suspension in 0.5 mL dose. ChAdOx1-S (AstraZeneca, UK) is a chimpanzee adenovirus encoding SARS-CoV-2 spike protein; one dose (0.5 mL) contains approximately 5.0 × 10^10^ viral particles. BNT162b2 (Pfizer, USA and BioNTech, Germany) is an mRNA vaccine encoding viral spike loaded into lipid nanoparticles; one dose (0.3 mL) contains 30 μg of mRNA. BBIBP-CorV (Sinopharm, China) is an inactivated SARS-CoV-2 vaccine; one dose (0.5 mL) contains 6.5 U of inactivated SARS-CoV-2 antigen and 0.225 mg of aluminum hydroxide adjuvant. All vaccines are injected intramuscularly into the deltoid.

All subjects were assessed for safety and immunogenicity after the booster immunization. Solicited adverse events (AEs) were recorded into paper diaries daily for seven days; unsolicited treatment-emergent adverse events (TEAEs) were collected using a memory aid and during scheduled visits through day 29. Serious adverse events (SAEs), medically attended adverse events (MAAEs), and adverse events of special interest (AESI), including pericarditis and myocarditis, were collected throughout the study period and assessed for severity and relatedness to the study treatments (Appendix 1, p. 61).

For immunogenicity analysis, serum samples were collected at the baseline (day 1), days 15, 29, and 180. For a subset of the BNT162b2 substudy subjects enrolled in the US and Panama sites, samples were also collected on day 361. All samples were kept frozen and shipped on dry ice to the central laboratory (Vismederi, Siena, Italy) for all immune tests, except for Fc-mediated functional antibodies that were tested at Seromyx, Woburn, MA, USA.

To analyze anti-N antibodies, samples for ChAdOx1-S and BNT162b2-primed subjects were tested by Elecsys COBAS® N protein assay (Roche, USA). For BBIBP-CorV primed subjects immune to the N-protein present in the inactivated vaccine, the anti-N antibodies were tested by quantitative ELISA.

SARS-CoV-2 RBD-specific IgG titers were detected by a validated direct S1-RBD binding ELISA test; geometric mean titer (GMT) between the two replicates for each sample was calculated.

Antibody neutralization titers were determined in a microneutralization cytopathic effect (CPE) based assay, a semi-quantitative antibody test used for the primary and key secondary endpoints for the original live SARS-CoV-2 hCoV-19/Australia/VIC01/2020, or Wuhan strain, and Omicron B.1.1.529 sub-lineage BA.5.2.1, or BA.5. A subset of day 29 samples was tested for neutralization of Omicron variant XBB.1.5.6, or XBB1.5. The microneutralization titer was calculated as the reciprocal of the highest sample dilution that protects from CPE at least 50% of Vero E6 cells and reported as the GMT between two replicates. For the GMT and GMFI analyses, antibody titer values below the lower limit of quantification (LLOQ) were imputed as 0.5 × LLOQ, and those above the upper limit of quantification (ULOQ) were imputed as ULOQ.

Antibody-dependent cellular phagocytosis (ADCP) measures the ability of antibodies to induce the phagocytosis of antibody-opsonized targets by monocytes. The assay was carried out with the SARS-CoV-2 RBD-His as an antigen and subset of serum samples collected on days 1 and 29. A phagocytic score was calculated taking in account the proportion of effector cells that phagocytosed and the degree of phagocytosis.^13^

Additional information on immune assays can be found in Appendix 2, pp. 46–47.

### Objectives/outcomes

Four weeks after immunization is a key timepoint typically used across clinical trials and supported by regulatory agencies to consistently evaluate COVID-19 vaccine immune responses.^6^^,^^7^ The primary immunogenicity objective was to demonstrate non-inferiority of day 29 neutralizing antibody GMTs against the Wuhan strain after a UB-612 heterologous booster to those after a homologous comparator vaccine in each substudy: BNT162b2, ChAdOx1-S, and BBIBP-CorV. The key secondary objective was to demonstrate non-inferiority of day 29 neutralizing antibody seroresponse rates against the Wuhan strain. The other secondary objectives were to demonstrate non-inferiority of the same immune responses against the Omicron BA.5 strain. Additional objectives included assessments of short and long-term neutralizing antibody responses, including a recent Omicron XBB1.5 strain, RBD-binding IgG and Fc-mediated effector activity in the ADCP assay.

The comparison of safety and tolerability profiles of UB-612 and comparator vaccine pairs were the primary safety objectives, which included prompted reactogenicity through day 8 following receipt of a study vaccine, and TEAEs through day 29 Deaths, SAE, MAAE, and AESI were monitored until the end of study.

### Statistical analysis

The sample size for each substudy was determined by targeting at least 90% power (BNT162b2 and BBIBP-CorV) with 1-sided alpha of 0.025 to test non-inferiority based on the GMT using a margin ratio of 1.5, and considered an estimated 20–25% exclusion for overall dropouts and protocol deviations depending on the countries of the enrollment. In the ChAdOx1-S substudy, limited access to active comparator vaccine restricted the sample size, thus permitting for a maximum power of 85%, assuming no dropouts. In the BBIBP-CorV substudy, a standard deviation (SD) of 0.44 required a sample size of 266 with 334 subjects planned for enrollment.^14^ In the ChAdOx1-S substudy, a SD of 0.5 required a sample size of 190 for enrollment.^15^ In the BNT162b2 substudy, a SD of 0.38 for Wuhan naturalizing antibodies required a sample size of 320 with 400 subjects planned for enrollment.^7^

The immunogenicity analyses were performed on subjects who received study vaccine, had baseline and day 29 immunogenicity antibody titer values, and had no major protocol deviations which would directly impact assessments of immunogenicity. For the analysis of neutralizing antibody GMTs, a covariance (ANCOVA) model was performed with a dependent variable of log-transformed (base 10) titer values at day 29, and with factors of treatment, age group (16–64 vs ≥65 years old), gender at birth, time since the last primary immunization dose (≥3–<5 months vs ≥5 months), baseline N-protein seropositivity (positive, negative) and baseline log titers as covariates. Noninferiority was determined if the lower bound of the 2-sided 95% confidence interval (CI) for the GMT ratio (UB-612/comparator) was >0.67. For both, the double-blind and open-label components in the BNT162b2 substudy, the analyses were based on pooled data. A sensitivity analysis was performed using baseline N-protein seropositivity results from the COBAS/ELISA tests in place of results from the rapid test used for stratification. Seroresponse rate was determined based on proportions of subjects with a ≥4-fold rise from before study vaccine booster injection to day 29 post-boost. A difference in the seroresponse rates between UB-612 and each comparator was calculated with 95% CI based on normal approximation. Noninferiority was concluded when the lower bound of the 95% CI greater than −10%. A superiority analysis was performed if the corresponding non-inferiority criteria were met and superiority determined if the lower bound of the 95% CI of the GMT ratio was over 1.0 and the difference in seroresponse rates was over 0%. Additionally, geometric mean fold increase (GMFI), defined as the geometric mean of the ratio of the post-vaccination titer value to the pre-vaccination titer value, was calculated for all post-baseline visits along with 95% CI. A similar analysis of GMT, seroresponse rates, and GMFI was performed for RBD-binding IgG antibodies.

Solicited local and systemic reactions, TEAEs, MAAEs, SAEs, and AESIs were summarized descriptively.

An independent data monitoring committee was chartered to review the study data during the conduct of the trial.

### Role of the funding source

The co-funder of the study, CEPI, had no role in study design, data collection, data analysis, data interpretation, or writing of the report.

## Results

**Fig. 1:**
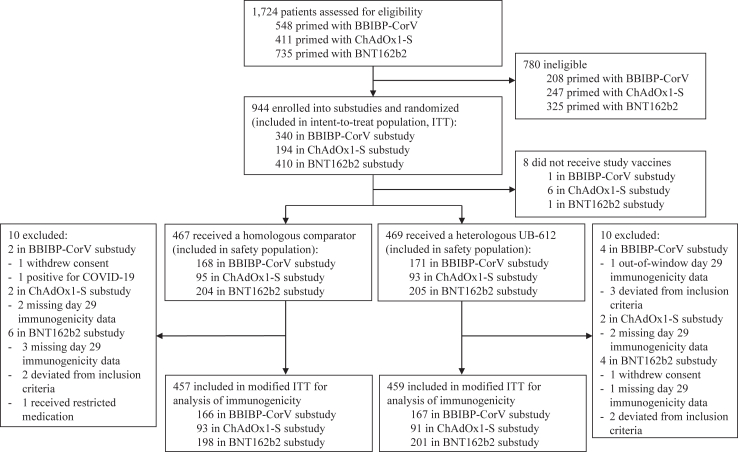
**Trial profile**.

Between March 22, 2022, and September 9, 2022, a total of 1724 subjects who completed primary immunization with one of the COVID-19 vaccines, including BNT162b2, ChAdOx1-S, or BBIBP-CorV, were recruited into three substudies (Fig. 1). From these subjects, 780 were found ineligible at screening mainly for the presence of a clinical or laboratory abnormality grade 2 or higher, or suspected HBV, HCV, HIV, or COVID-19. The remaining 944 subjects, representing the intent-to-treat population (ITT), were enrolled into the respective substudy according to their COVID-19 primary vaccination and randomized into groups to receive either a homologous booster with BBIBP-CorV (n = 168), ChAdOx1-S (n = 95), BNT162b2 (n = 204, including n = 39 open-label), or a heterologous booster with UB-612 in BBIBP-CorV-primed subjects (n = 171), ChAdOx1-S-primed subjects (n = 93), and BNT162b2-primed subjects (n = 205, including n = 42 open-label). From the overall safety population, comprised of all subjects who received the study vaccines (n = 936), twenty had missing key immunogenicity data, deviated from selection criteria, tested positive for COVID-19, or withdrew the informed consent, and, thus, were excluded to form a modified ITT population (mITT, n = 916) used for the primary and key secondary immunogenicity analyses.

Over 98% of subjects were between 16 and 64 years of age (Table 1). The gender at birth distribution was generally balanced. All subjects in the BBIBP-CorV and ChAdOx1-S substudies enrolled exclusively in Philippines and identified themselves as of Asian race. The BNT162b2 substudy was enrolled in the US, Panama, and Philippines, resulting in a diverse population, including mixed races reported as “Other” among 184 (44.9%) participants.

**Table 1:** Demographic of intent-to-treat populations and key baseline parameters used for stratification.

	BBIBP-CorV substudy		ChAdOx1-S substudy		BNT162b2 substudy	
	BBIBP-CorV (n = 168)	UB-612 (n = 172)	ChAdOx1-S (n = 98)	UB-612 (n = 96)	BNT162b2 (n = 205)	UB-612 (n = 205)
*Age, years*						
Mean (SD) range, min-max	35.3 (12.3), 19–71	36.7 (13.3), 18–72	31.7 (10.9), 19–63	33.0 (11.9), 18–63	32.1 (11.1), 16–62	31.8 (12.0), 16–70
18–64, n (%)	167 (99.4)	167 (97.1)	98 (100.0)	96 (100.0)	205 (100.0)	201 (98.0)
≥65, n (%)	1 (0.6)	5 (2.9)	0 (0.0)	0 (0.0)	0 (0.0)	4 (2.0)
*Sex, n (%)*						
Male	96 (57.1)	99 (57.6)	53 (54.1)	53 (55.2)	112 (54.6)	112 (54.6)
Female	72 (42.9)	73 (42.4)	45 (45.9)	43 (44.8)	93 (45.4)	93 (45.4)
*Race, n (%)*						
White	0 (0.0)	0 (0.0)	0 (0.0)	0 (0.0)	1 (0.5)	2 (1.0)
Black	0 (0.0)	0 (0.0)	0 (0.0)	0 (0.0)	0 (0.0)	1 (0.5)
Asian	168 (100.0)	172 (100.0)	98 (100.0)	96 (100.0)	77 (37.6)	80 (39.0)
Amer. Indian, Alaska Native	0 (0.0)	0 (0.0)	0 (0.0)	0 (0.0)	34 (16.6)	31 (15.1)
Other	0 (0.0)	0 (0.0)	0 (0.0)	0 (0.0)	93 (45.4)	91 (44.4)
*Time since the last immunization, n (%)*						
3≥–<5 months	0 (0.0)	0 (0.0)	0	1 (1.0)	0	0
≥5 months	168 (100.0)	172 (100.0)	98 (100.0)	95 (99.0)	205 (100.0)	205 (100.0)
*N-protein seropositivity by rapid test, n (%)*						
Positive	101 (60.1)	106 (61.6)	94 (95.9)	93 (96.9)	195 (95.1)	194 (94.6)
Negative	67 (39.9)	66 (38.4)	4 (4.1)	3 (3.1)	10 (4.9)	11 (5.4)

Except for one individual in the ChAdOx1-S substudy, all subjects completed the primary series with the last vaccination having occurred over 5 months prior to the booster dose. The initial screening for prior infection with SARS-CoV-2 was performed by a rapid test for nucleocapsid (N) protein specific antibodies with 207 (60.9%) seropositive subjects identified in BBIPB-CorV, 187 (96.4%) in ChAdOx1-S, and 389 (94.9%) in BNT162b2 substudies (Table 1). The results were further confirmed by ELISA or COBAS tests, and seropositivity for N-protein was found among 94.4% of subjects in BBIBP-CorV, 90.7% in ChAdOx1-S, and 93.7% in BNT162b2 substudies (Appendix 2, p. 1).

At baseline, there were no significant differences in RBD-binding IgG antibodies, or Wuhan or Omicron BA.5 neutralizing antibodies among the treatment groups in each substudy. The choice of COVID-19 vaccine platform for primary immunization resulted in variations in baseline antibody levels and antibody responses after the study vaccine boosting between substudies (Appendix 2, pp. 2–10).

**Fig. 2:**
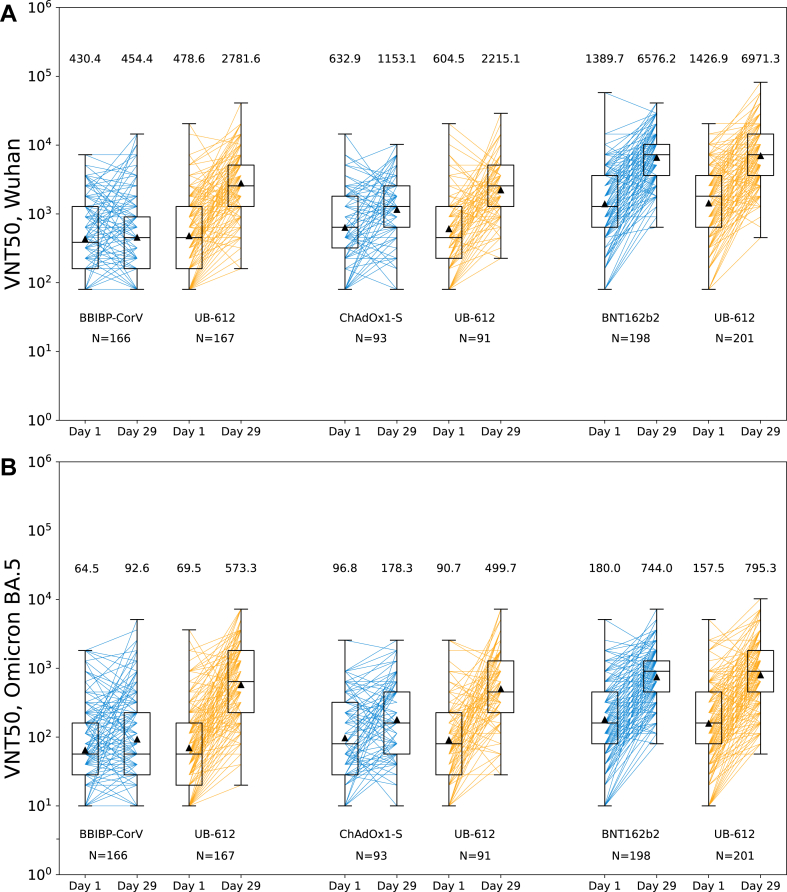
**Live virus neutralizing antibody titers against (A) Wuhan and (B) Omicron BA.5 at baseline and 28 days after booster immunization by substudy treatment pairs**. Lines connect values from the same participant. The boxes show interquartile range (IQR) with the medians as midlines. The geometric means are presented as black triangles with values shown above the graphs. Error bars extend to the last data point within 1.5 × the IQR. Virus neutralization titer (VNT50) is the reciprocal serum dilution achieving 50% neutralization of virus.

Upon analyzing antibody responses against the Wuhan strain, the neutralizing antibody GMT on day 29 after homologous boosting ranged from 454.4 after BBIBP-CorV to 1153.2 after ChAdOx1-S, and 6576.2 after BNT162b2 (Fig. 2A and Appendix 2, pp. 2, 5, and 8). The heterologous UB-612 boosters proved as or more effective than homologous boosting in stimulating day 29 neutralizing antibodies, with GMT of 2781.6 in BBIBP-CorV-primed subjects, 2215.1 in ChAdOx1-S-primed subjects, and 6971.3 in BNT162b2-primed subjects.

**Fig. 3:**
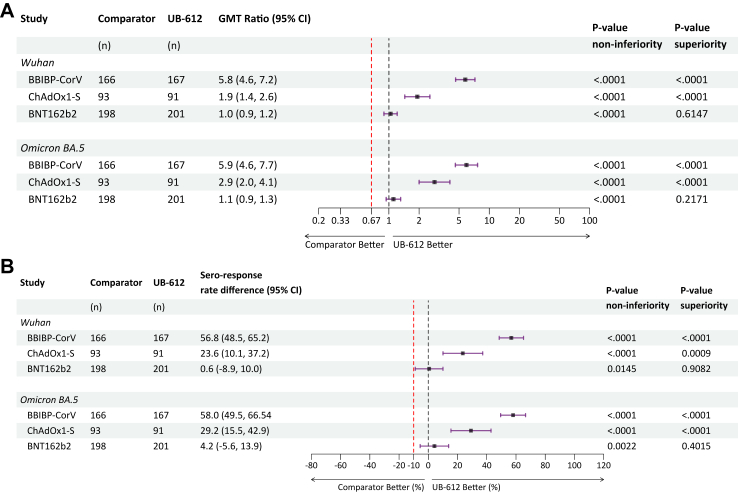
**Analysis of neutralizing antibody responses by (A) GMT ratio and (B) difference of seroresponse rates between substudy treatment pairs**. Red vertical dashed lines represent the threshold for non-inferiority, black vertical dashed line for superiority. GMT = geometric mean titer.

Day 29 post-boost Wuhan strain neutralizing antibody GMT ratio was the primary statistical endpoint of the study. Heterologous UB-612 booster vaccination was statistically superior relative to homologous regimens with BBIB-CorV GMT ratio of 5.8 (95% CI 4.6, 7.2, p < 0.0001) and ChAdOx1-S GMT ratio 1.9 (1.4, 2.6, p < 0.0001, Fig. 3A). Boosting with UB-612 was non-inferior to BNT162b2, with the GMT ratio 1.0 (0.9, 1.2, p = 0.6147).

In the key secondary immunogenicity endpoint, seroresponse rates, defined as the proportion of subjects with ≥4-fold increase in neutralizing antibody GMT from baseline to day 29, the heterologous UB-612 booster was superior compared to both homologous BBIBP-CorV and ChAdOx1-S (Fig. 3B). The difference in seroresponse rates of 56.8% (95% CI 48.4, 65.2, p < 0.0001) favored UB-612 over BBIBP-CorV, and the difference of 23.6% (10.1, 37.2, p = 0.0009) favored UB-612 over ChAdOx1-S. The difference in seroresponse rates of 0.6% (−8.92, 10.04, p = 0.9082) between UB-612 and BNT162b2 groups was non-inferior.

To explore the breadth of activity against subsequently emerging SARS-CoV-2 strains, neutralizing antibodies against Omicron BA.5, were analyzed. The peak Omicron BA.5 neutralizing antibody GMTs on day 29 were lower than those against the Wuhan strain across treatment groups (Fig. 2B, Appendix 2, pp. 3, 6, and 9). UB-612 was found to be superior at boosting Omicron BA.5 neutralizing antibodies to BBIBP-CorV with GMT ratio of 5.9 (95% CI 4.6, 7.6, p < 0.0001) and to ChAdOx1-S 2.9 (2.0, 4.1, p < 0.0001) (Fig. 3A). The neutralizing antibody response compared to BNT162b2 was confirmed to be non-inferior with the GMT ratio of 1.1 (0.9, 1.3, p = 0.2171). The superiority of UB-612 against BBIBP-CorV and ChAdOx1-S and its non-inferiority against BNT162b2 boosters were also demonstrated in the comparison of seroresponse rates of Omicron BA.5 neutralizing antibodies (Fig. 3B). An additional analysis of neutralizing antibody geometric mean fold increase (GMFI) from baseline provided additional evidence of comparable responses over Wuhan and Omicron BA.5 variants between BNT162b2 and UB-612, while favoring UB-612 over BBIBP-CorV or ChAdOx1-S 1.9 (Appendix 2, pp. 2–10).

**Fig. 4:**
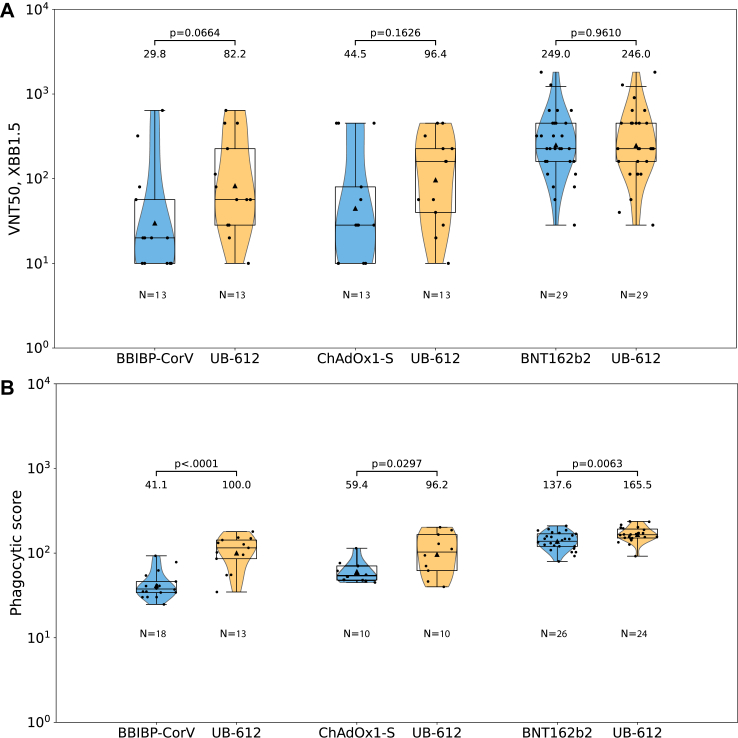
**Virus neutralizing activity against Omicron XBB1.5 (A) and Fc-mediated activity in ADCP (B)**. A subset of Day 29 visit samples from each substudy pair is represented by (N). The boxes show interquartile range (IQR) with the medians as midlines. The geometric means are presented as black triangles with values shown above the graphs. Error bars extend to the last data point within 1.5 × the IQR. Non-inferiority analysis of geometric mean ratios is summarized by p-values above the evaluated pairs connected by brackets. Virus neutralization titer (VNT50) is the reciprocal serum dilution achieving 50% neutralization of virus. ADCP = antibody-dependent cellular phagocytosis.

The exploratory analysis of neutralizing antibodies against a more recent SARS-CoV-2 Omicron variant, XBB1.5, was conducted in a subset of day 29 serum samples collected from participants representing each substudy. In this diverse population, UB-612 induced neutralizing antibodies with GMTs ranging from 82.2 in BBIBP-CorV-primed subjects to 96.4 in ChAdOx1-S, and 246.0 in BNT162b2-primed subjects (Fig. 4A).

In addition to virus neutralizing antibodies, the UB-612 booster was evaluated for other antibody responses potentially contributing to protection, including RBD-binding IgG antibodies and non-neutralizing antibodies with Fc-effector function in antibody-dependent cellular phagocytosis (ADCP). The UB-612 heterologous booster resulted in higher day 29 IgG antibodies binding to RBD compared to all homologous regimens, with GMT ratios of 8.4 (95% CI 6.9, 10.3) against BBIBP-CorV, 3.6 (2.7, 4.7) against ChAdOx1-S, and 1.7 (1.5, 2.0) against BNT162b2 (Appendix 2, pp. 4, 7, and 10). Seroresponse rates and GMFI analyses also favored IgG antibody responses after the heterologous UB-612 booster over any homologous comparator in the study (Appendix 2, pp. 4, 7, and 10). In the ADCP assay, performed on a subset of samples collected on day 29, the UB-612 booster stimulated higher titers of antibodies with ADCP Fc-effector functions compared to homologous regimens, with GMT ratios of 2.5 (95% CI 1.8, 3.4) for BBIBP-CorV, 1.6 (1.0, 2.5) for ChAdOx1-S, and 1.1 (1.0, 1.3) for BNT162b2 (Fig. 4B).

To assess the speed of recall and durability of antibody responses, all subjects provided serum samples on approximately days 15, 180, and 361 (day 361 for a subset of BNT162b2 substudy subjects only) for analyses of neutralizing antibodies against Wuhan and Omicron BA.5, and RBD-binding IgG1 (Appendix 2, pp. 2–10). The results of day 15 samples across GMT, seroresponse rates, and GMFI analyses closely mirrored those from day 29 analyses. By day 180, Wuhan neutralizing antibody GMTs declined to levels similar to baseline. However, at day 180 the heterologous UB-612 booster demonstrated modestly higher levels of the Wuhan neutralizing antibodies than the homologous comparators, with GMT ratios of 1.9 (95% CI 1.5, 2.3) for BBIBP-CorV, 1.2 (95% CI 0.9, 1.7) for ChAdOx1-S, and 1.1 (95% CI 0.9, 1.3) for BNT162b2. There was similarly a decline in Omicron BA.5 neutralizing antibody levels after the heterologous booster from day 29 to day 180, yet UB-612 demonstrated a trend of being more immunogenic than BBIBP-CorV with GMT ratios of 1.5 (95% CI 1.2, 2.0), ChAdOx1-S 1.4 (95% CI 1.0, 1.9), and BNT162b2 1.1 (95% CI 1.0, 1.4). Notably, among the homologous regimens, a decline in Omicron BA.5 neutralizing antibodies was observed only for BNT162b2, with day 29 GMT 744.0 and day 180 GMT 393.7. For ChAdOx1-S boosted subjects, the level of Omicron BA.5 neutralizing antibodies remained steady with GMT 178.3 on day 29 and 203.62 on day 180, and for BBIBP-CorV boosted subjects, the level increased from GMT 92.6 on day 29 to 181.4 on day 180. The results of seroresponse rates and GMFI further corroborated with the differences in the kinetics of Omicron BA.5 neutralizing antibodies between UB-612 and ChAdPx1-S or BBIB-CorV booted subjects.

Subset analyses did not reveal impacts of age (cohorts of 18–25, 26–49, and 50–64 years), gender at birth, comorbidities, and or baseline N-protein seropositivity on the overall antibody responses measured in treatment groups (Appendix 2, pp. 11–22). However, in the BNT162b2 substudy, conducted across the Americas and Asia–Pacific sites, geographical, and racial effects on immune responses were observed, with variability in results depending on SARS-CoV-2 strain and antibody analysis methods used. In the Americas, Wuhan and Omicron BA.5 neutralizing antibody GMT responses after the UB-612 heterologous booster were higher compared to the BNT162b2 homologous booster. In the Asia–Pacific region, Wuhan neutralizing antibody GMT was lower after the UB-612 booster compared to BNT162b2, while the Omicron BA.5 antibodies were found in line with the overall treatment effect.

**Safety analysis** was conducted on all subjects who received a study vaccine, with results covering approximately 12 months of safety monitoring included in this report. The safety profile following a heterologous boost of UB-612 was generally similar to a homologous boost with each of the active comparators and did not present any additional risks in subjects (Table 2).

**Table 2:** Safety summary.

	BBIBP-CorV substudy, n (%)		ChAdOx1-S substudy, n (%)		BNT162b2 substudy, n (%)	
	BBIBP-CorV (n = 168)	UB-612 (n = 171)	ChAdOx1-S (n = 95)	UB-612 (n = 93)	BNT162b2 (n = 204)	UB-612 (n = 205)
*Any solicited AEs*	11 (6.5)	17 (9.9)	6 (6.3)	5 (5.4)	105 (51.5)	89 (43.4)
Local AEs	5 (3.0)	8 (4.7)	3 (3.2)	5 (5.4)	97 (47.5)	68 (33.2)
Systemic AEs	7 (4.2)	10 (5.8)	4 (4.2)	2 (2.2)	72 (35.8)	62 (30.2)
*Reactogenicity*^a^*AEs*	11 (6.5)	17 (9.9)	6 (6.3)	5 (5.4)	110 (53.9)	91 (44.4)
*Any unsolicited TEAEs*	11 (6.5)	11 (6.4)	11 (11.6)	24 (25.8)	63 (30.9)	59 (28.8)
Treatment-related (at least possibly)	1 (0.6)	2 (1.2)	1 (1.1)	5 (5.4)	23 (11.3)	22 (10.7)
Serious TEAEs	1 (0.6)	0	1 (1.1)	0	1 (0.5)	0
Study discontinuation	1 (0.6)	0	0	0	0	0
MAAEs	7 (4.2)	6 (3.5)	9 (9.5)	12 (12.9)	57 (27.9)	43 (21.0)
AESIs	1 (0.6)	0	2 (2.1)	1 (1.1)	2 (1.0)	0
Death	1 (0.6)	0	0	0	0	0

TEAE = treatment emergent AE; MAAE = medically attended AE; AESI = AE of special interest.

a Note: Solicited adverse events (AEs) and reactogenicity AEs were recorded through 7 days after study product injection. Solicited AEs were reported on subject diaries only, reactogenicity AEs combined the solicited AEs reported on subjects' diaries and AE terms matching the definition of solicited AEs reported by investigators.

**Fig. 5:**
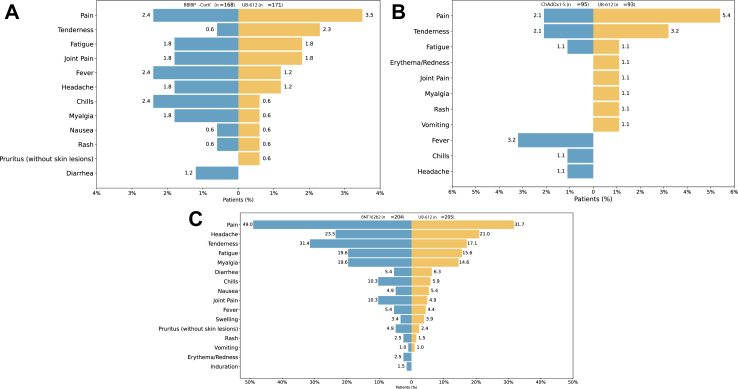
**Local and systemic adverse reactions in the first 7 days after booster immunization in intentto-treat-population**. (A) BBIBP-CorV substudy, (B) ChAdOx1-S substudy, (C) BNT162b2 substudy. Reactogenicity includes solicited AEs on subjects' diaries and AEs matching the definition of solicited reported by PIs. Analysis of solicited AEs on subject's diaries only is presented in Appendix 2, pp. 23–25.

Reactogenicity analysis obtained from subjects' diaries and matched solicited terms reported by investigators during the first 7 days after immunization, demonstrated a low frequency of adverse events, balanced between treatment pairs (Fig. 5). Solicited adverse events were reported by 6.5% of BBIBP-CorV subjects and 9.9% by UB-612 subjects in the BBIBP-CorV substudy, and by 6.3% for ChAdOx1-S and 5.4% by UB-612 in the ChAdOx1-S substudy, respectively. Most solicited adverse events were mild or moderate, with one severe fever reported in the UB-612 group of the BBIBP-CorV substudy (Appendix 2, pp. 23–25). The most frequent local adverse events included pain and tenderness at the injection site, and frequent systemic adverse events were joint pain, fatigue, chills, and fever. In the BNT162b2 substudy, the proportion of subjects experiencing any solicited adverse event was higher in the BNT162b2 group (53.9%) than in the UB-612 group (44.4%). Common local adverse events included injection site pain and tenderness, and systemic adverse events included headache, myalgia, and fatigue. There was a higher incidence of severe adverse events in the BNT162b2 group (3.9%) compared to the UB-612 group (2.0%). No subject in either group experienced solicited adverse events of severity Grade 4 or higher. In all substudies, reactogenicity symptoms lasted no longer than 4 days and typically resolved within 2 days.

No serious adverse events, adverse events leading to study discontinuation, or deaths were reported through day 29. Subsequent to day 29, three serious adverse events were reported, all assessed as unrelated, including one sudden cardiac death in a subject who received BBIBP-CorV (Appendix 2, p. 49), pre-eclampsia after ChAdOx1-S, and cholelithiasis after BNT162b2 boosters.

Treatment-emergent adverse events were analyzed through day 29 after the booster immunization (Table 2). The proportions of subjects experiencing TEAEs were similar between the BBIBP-CorV and UB-612 booster groups (6.5% vs 6.4%), and BNT162b2 and UB-612 booster groups (28.8% vs 30.9%), but higher for subjects who received UB-612 compared to ChAdOx1-S (25.8% vs 11.6%), driven largely by eosinophilia, hyperbilirubinemia, and hypertransaminasemia resulting from laboratory abnormalities (Appendix 2, pp. 26–28). Treatment-related events were low and balanced between the treatment pairs. Most TEAEs, including treatment-related, were mild or moderate in severity across the substudies (Appendix 2, pp. 29–31).

Throughout the study's safety follow-up, the incidence of MAAEs was similar between treatment arms in each substudy (Table 2). MAAEs were reported for 3.5% of subjects in the UB-612 and 4.2% in the BBIBP-CorV groups, 12.9% in the UB-612 and 9.5% in the ChAdOx1-S groups, and 21.0% in the UB-612 and 27.9% in the BNT162b2 groups. AESIs were monitored, with one mild arrhythmia reported as unrelated to BBIBP-CorV booster, one case of coronary artery disease possibly related to UB-612, and two cases of myocardial ischemia unrelated to ChAdOx1-S booster. In the BNT162b2 group, one subject experienced moderate peripheral facial paralysis and another mild rosacea, both assessed as unrelated.

Subgroup analyses were performed for stratification factors, and also for comorbidities, and confirmatory test baseline N-protein seropositivity. Additionally, for the BNT162b2 substudy, race and geographical region were analyzed. No imbalance was observed in reactogenicity and TEAEs among treatment groups in each substudy. However, within the treatment pairs, N-protein seropositivity at baseline was associated with higher reporting of reactogenicity or treatment-emergent adverse events (Appendix 2, pp. 32–39). Imbalance in reactogenicity and TEAEs based on race and region was noted in the BNT162b2 substudy, with subjects of Asian race, and Asian-Pacific region nationals, representing the same population in the Philippines, reporting lower rates compared to other subgroups.

## Discussion

The evolution of SARS-CoV-2 and the rapid decline of protective antibodies after primary immunization has necessitated boosting to maintain protective immunity. To address this, “mix-match” schedules using heterologous boosters, typically authorized via immunobridging to already licensed comparators, have gained wide acceptance.^16^ However, most authorized heterologous boosters have evidence of restoring immunity stimulated by only one or two COVID-19 vaccine platforms. A few subunit vaccines that were explored in more diverse populations were underpowered or reportedly fell short of matching neutralizing antibodies against the prototype or Omicron SARS-CoV-2 variants in comparison to mRNA homologous boosters.^17^, ^18^, ^19^, ^20^ Restoring immunity initially induced by primary immunization with three approved and widely used COVID-19 vaccine platforms, particularly through the generation of virus neutralizing antibodies, was a primary objective of this trial. All prespecified primary and secondary objectives aimed to demonstrate non-inferiority of neutralizing antibodies against SARS-CoV-2 after heterologous booster with UB-612 compared to homologous active comparators, including mRNA, were successfully achieved. Moreover, UB-612 demonstrated superiority in stimulating neutralizing antibody responses compared to inactivated and adenovirus vaccines. Short-term antibody response results indicated rapid reinstatement of protective immunity as early as two weeks post-immunization, favoring UB-612 over inactivated virus and adenovirus-vectored platforms.

With the exception of inactivated virus vaccines comprised of multiple viral antigens, all other COVID-19 vaccine platforms target the S protein, the primary viral antigen responsible for eliciting neutralizing antibodies, a key mediator of protection against COVID-19.^21^ Boosting with traditional COVID-19 vaccines may lead to preferential recognition of suboptimal conserved epitopes, resulting in diminished recall of neutralizing antibodies, especially those specific against the emerging SARS-CoV-2 variants.^22^^,^^23^ Updating booster vaccines composition to include antigens derived from circulating SARS-CoV-2 strains mitigates this risk.^24^ RBD-based immunization, however, maintains immune focus on key antigenic determinants responsible for stimulating over 90% of all SARS-CoV-2 neutralizing antibodies.^25^ UB-612 is characterized by a unique antigen composition, including the RBD subunit for stimulating broadly cross-reactive neutralizing antibodies against multiple SARS-CoV-2 variants, including Delta and Omicron, as observed during Phase 1 and Phase 2 studies.^11^^,^^12^ Based on the results of this pivotal study, a heterologous booster of UB-612 provided cross-reactive neutralizing antibodies to the Omicron variants, BA.5 and XBB1.5. Though neutralizing antibodies are crucial for COVID-19 protection, non-neutralizing antibodies inducing Fc-dependent effector functions have also been recognized as important in clearing infection, especially caused by highly mutated SARS-CoV-2 variants.^26^ Heterologous booster immunization with UB-612 stimulated higher titers of IgG antibodies binding to RBD compared to any active comparators in the study. Additional analysis of Fc-mediated effector functions further supported higher activity in ADCP tests, favoring heterologous booster immunization with UB-612 over homologous boosting.

Long-lasting immune responses may provide additional advantages when selecting booster programs. Most original COVID-19 vaccines were found to stimulate short-lived antibody responses rapidly waning over three to eight months post-homologous booster immunization.^27^ Heterologous boosters were found to stimulate more durable antibodies, as supported by this trial's results showing generally higher antibody levels six months post-immunization with UB-612 compared to other COVID-19 vaccine platforms.^23^

Safety profiles of heterologous booster regimens have depended on the primary series' COVID-19 vaccine platform, with mRNA and adenovirus-vectored combinations often displaying higher reactogenicity.^6^ Across the entire UB-612 clinical development program, encompassing over 4200 subjects exposed to varying dose levels and regimens, including both primary and booster schedules, follow-up immunizations were not found to pose additional risks compared to booster comparators. Reactogenicity rates were typically mild to moderate and comparable between treatment arms, with UB-612 appearing to have a slightly more favorable profile than BNT162b2. The incidence of unsolicited TEAEs was generally balanced between treatment groups in all substudies. In the ChAdOx1-S substudy, the overall low rate of unsolicited TEAEs appeared slightly higher in the UB-612 group. This observed imbalance was driven by laboratory abnormalities possibly linked to HBV prevalence and helminth infestations in the Philippines, not accounted for by stratification, and likely incidental.^28^^,^^29^ There was no impact on the frequency of solicited adverse reactions and unsolicited TEAEs attributed to subpopulations in all substudies except for race and geographical region across both treatment groups in the BNT162b2 substudy. This observed difference is attributed to unique cultural behaviors in safety reporting among Filipino nationals.^30^^,^^31^

The trial has several limitations, including limited enrollment of elderly and adolescent subjects. A model analysis from a combined UB-612 third-dose immunogenicity dataset from Phase 2 and Phase 3 studies, including over 690 elderly subjects, revealed no significant age-dependent decline in antibodies in subjects over 65 years, although a drop was noted at age 35 compared to younger subjects. These results were consistent with other reports showing limited age differences in antibody responses among immunologically diverse subjects after a third COVID-19 immunization (the model analysis and reference benchmarks are presented in Appendix 2, pp. 40–45). Additionally, the UB-612 database primarily comprises an Asian population, with limited representation of other populations. The study could not assess vaccine efficacy directly due to a low number of COVID-19 breakthroughs. There were suspected immunological signatures of possible Omicron breakthrough infections observed only among the subjects boosted with ChAdOx1-S and BBIBP-CorV vaccines which may provide limited indirect evidence of a more protective immune response in the reciprocal groups boosted with UB-612 or BNT162b2. Due to technical issues, we couldn't evaluate T-cell responses post-booster immunizations. In prior clinical studies, primary immunization of sero-naïve subjects with UB-612 stimulated long-lasting cellular immunity.^10^^,^^12^

In summary, the results of this pivotal Phase 3 study, based on well-understood surrogate markers of protection, demonstrate the benefits of a third-dose booster with subunit UB-612 vaccines in restoring protective neutralizing antibodies, including against recent SARS-CoV-2 strains, in immunologically diverse adult populations. The balanced risks and benefits support the potential broad use of UB-612 as a heterologous booster across the most widely used COVID-19 vaccines, including mRNA, inactivated virus, or adenovirus-vectored, with market authorization review underway.

## Contributors

AR, LW, AF, RZ, KW, GH, UP, and TPM contributed to the conceptualization and the study design. SW and TK contributed to the study methodology. AR, TPM, GH, and TK were responsible for funding acquisition. AR, TK, AW, KB, DF, SH, XSL, and EA were involved in project administration. AR, SW, TK, GP, AM, EM, RD, XSL, EA, MDLB, and AMJ were involved in the investigation and data collection. TK, AW, XSL, EA, UP provided supervision. AR, LW, SW, AF, RZ, KW, RD, XSL, and TPM were responsible for data interpretation and data curation. AR, RZ, KW, CS, RD, and XSL performed formal analysis. XSL was responsible for data validation. AR, KW, and CS worked on visualization. AR, LW, RD, and CS contributed to the original draft. AR, AW, AB, AF, SH, ML, GP, AM, EM, RD, XSL, EA, MDLB, AMJ, GH, UP, and TPM were involved in reviewing and editing the manuscript. AR, LW, RZ, and KW accessed and verified the data. The authors take full responsibility for all aspects of the work, ensuring that any concerns regarding accuracy or integrity are properly addressed. Each author had unrestricted access to all study data and held the final authority in the decision to submit the manuscript for publication.

## Data sharing statement

Study information is available online. The study protocol and additional analysis are available in the Supplemental Appendixes 1 and 2. Additional information submitted to the corresponding author may be considered upon request.

## Declaration of interests

AR, LW, SW, TK, AW, AB, DF, KB, AF, SH, ML, GH, UP, and TPM were Vaxxinity salaried employees with AR, SW, AB, AF, ML, and GH also reported to hold the company stocks. GH was affiliated with Crozet Biopharma and Quigley Bio. AF held stocks of ILiAD. TK and GH received grants from CEPI. RZ, KW, CS, GP, AM, EM, RD, MC, and EA provided services under a contact with Vaxxinity. XSL provided services under contract with Cevaxin. AF, RZ, KW, and CS received consulting fees from Vaxxinity. GH received consulting fees from General Dynamics Information Technology and Global Health Innovation Technology Fund. AF received consulting fees from ILiAD, Replicate, Telum and Bloom. All other authors declare no competing interests.

## Appendix A Supplementary data

Appendix 2

Vaxxinity_UB-612-305_SAP
