# Supplementary material for: Safety and immunogenicity of UB-612 heterologous booster in adults primed with mRNA, adenovirus, or inactivated COVID-19 vaccines: a randomized, active-controlled, Phase 3 trial
Source: eClinicalMedicine. 2025 Jul 21;86:103349. doi: 10.1016/j.eclinm.2025.103349 (PMC12301762; doi:10.1016/j.eclinm.2025.103349)
Supplement: Appendix 1 [file mmc1.pdf]

CLINICAL TRIAL PROTOCOL

Investigational Product:
UB-612

A Phase 3 Multi-Center International, Randomized, Active-Controlled Platform Trial to Compare Homologous Boost of Authorized COVID-19 Vaccines and Heterologous Boost with UB-612 Vaccine

Protocol Number:	*Confidential*	Version: 10.0
UB-612-305		Date: 23 September 2022

This document contains confidential information and is the property of Vaxxinity. The receiver(s) of this document understands and agrees that this document is not to be disclosed to any unauthorized third party without written agreement issued by the sponsor. The need to disclose certain information contained in this document for the purpose of obtaining informed consent/assent from potential subjects or his/her legally acceptable representative(s) of this study will be considered as exceptions.

PRINCIPAL INVESTIGATOR'S SIGNATURE PAGE

I understand the obligations as a clinical trial investigator and agree to perform and report the study in compliance to the protocol, good clinical practice (GCP), and the current rules and regulations set forth by the applicable health authorities. I will assure that no deviation from, or changes to the protocol will take place without prior agreement from the sponsor and documented approval from the Institutional Review Board (IRB)/Independent Ethics Committee (IEC), except where necessary to eliminate an immediate hazard(s) to trial participants.

Name: ______________________________

Title: ______________________________

Affiliation: ______________________________

Signature: ______________________________

SPONSOR SIGNATURE PAGE

I have reviewed this Clinical Trial Protocol and approve its contents.

Ulo Palm, MD, PhD, MBA
Chief Medical Officer
Vaxxinity, Inc.

Date

Sarah Luijpers
SVP, Head Clinical Operations
Vaxxinity, Inc.

Date

Sasha Rumyantsev MD, PhD, MBA
Therapeutic Area Head Infectious Diseases
Vaxxinity, Inc.

Date

Lixia Wang PhD
SVP, Data Science
Vaxxinity, Inc.

Date

TABLE OF CONTENTS

1 SUMMARY OF CHANGES 8
1.1 Version History 8
1.2 Changes 8
2 SYNOPSIS 11
2.1 Study Summary 11
2.2 Schedule of Assessments 14
3 LIST OF ABBREVIATIONS 19
4 GENERAL INFORMATION 22
5 INTRODUCTION AND RATIONALE 23
5.1 Disease Overview 23
5.2 UB-612 23
5.2.1 Summary of Nonclinical Findings 24
5.2.2 Clinical Experience 24
5.3 Study Rationale 24
5.4 Risk/Benefit Assessment 27
5.4.1 Potential Benefit 27
5.4.2 Potential Risks 27
5.5 Risk of Myocarditis and Pericarditis Associated with COVID-19 Vaccines 29
6 OBJECTIVES AND ENDPOINTS 30
6.1 Surveillance for Enhanced COVID-19 31
6.2 Surveillance for Myocarditis and Pericarditis 32
7 STUDY DESIGN 33
7.1 Overall Study Design 33
7.1.1 Rules for Suspension or Pause of the Study 34
7.1.2 Restarting After Suspending or Pausing the Study 34
7.2 Scientific Rationale for Study Design 35
7.3 Justification for Dose 35
7.4 End of Study Definition 35
8 STUDY POPULATION 36
8.1 Inclusion Criteria 36
8.2 Exclusion Criteria 37
8.3 Lifestyle Considerations 39
8.4 Screen Failures 39
9 TREATMENT OF SUBJECTS 40
9.1 Description of the Study Vaccine 40
9.1.1 UB-612 40
9.1.2 Comparator Vaccine 40
9.1.3 Dosing Regimens 40

9.2 Preparation/Handling/Storage/Accountability of the Study Drug....41
9.2.1 Acquisition and Accountability....41
9.2.2 Formulation, Appearance, Packaging, and Labelling....41
9.2.3 Product Storage and Stability....41
9.2.4 Preparation....41
9.3 Measures to Minimize Bias: Randomization and Blinding....42
9.4 Study Vaccine Compliance....42
9.5 Concomitant Medications....43
9.5.1 Medications Restricted During the Study....43
9.5.2 Medications Permitted During the Study....44
10 STUDY PRODUCT DISCONTINUATION AND PARTICIPANT DISCONTINUATION/WITHDRAWAL....45
10.1 Discontinuation of Study Product....45
10.2 Participant Discontinuation/Withdrawal from the Study....45
10.2.1 Withdrawal of Consent/Assent for Disclosure of Future Information....45
10.2.2 Lost to Follow-up....45
10.2.3 Contraception Requirements....46
11 STUDY ASSESSMENTS AND PROCEDURES....48
11.1 Timing and Events....48
11.1.1 Screening....48
11.1.2 Booster Vaccination....48
11.1.3 Post Vaccination Follow-up....48
11.1.4 Unscheduled Visit....49
11.2 Study Assessments....50
11.2.1 Demographics....50
11.2.2 Medical History and Baseline Characteristics....50
11.2.3 SARS-CoV-2 Testing....50
11.2.4 Physical and Neurological Examination....50
11.2.5 Height and Weight....50
11.2.6 Vital Signs....51
11.2.7 Laboratory Assessments....51
11.2.8 Pregnancy Test....51
11.2.9 Electrocardiogram....52
11.2.10 Collection of Solicited Local and Systemic Adverse Events....52
11.2.11 COVID-19 Surveillance....55
11.2.12 Immunogenicity....60
11.3 Adverse Events....61
11.3.1 Definitions....61
11.3.2 Collection of AEs, MAAEs, SAEs, and AESIs....64
11.3.3 Reporting and Follow-up of AEs, SAEs, and AESIs....64

11.3.4 Assessment of Severity 65
11.3.5 Relationship to Study Vaccine Administration 66
11.3.6 Abnormal Laboratory Values 67
11.3.7 Medication Errors 68
11.3.8 Safety Monitoring Plan 68
11.3.9 Independent Data and Monitoring Committee (IDMC) 68
12 STATISTICS 69
12.1 Hypothesis Testing 69
12.2 Power and Sample Size 69
12.2.1 BNT162b2 Sub-studies 69
12.2.2 ChAdOx1-S Sub-studies 70
12.2.3 BIBP Sub-study 70
12.3 Interim Analysis 70
12.4 There is no interim analysis planned for this study. Missing Data 70
12.5 Analysis Sets 70
12.6 Statistical Methods 71
12.6.1 Analysis of Immunogenicity 71
12.6.2 Analysis of Safety 72
13 QUALITY CONTROL AND QUALITY ASSURANCE 74
13.1 Responsibility of the Investigator(s) 74
13.2 Responsibility of the Sponsor 74
13.3 Source Document Requirements 74
13.4 Use and Completion of Case Report Forms (CRFs) and Additional Request 75
14 ETHICS 76
14.1 Declaration of Helsinki and Ethical Review 76
14.2 Patient Information and Consent/Assent 76
14.3 Patient Data Protection 76
14.3.1 Biological Samples 76
15 DATA HANDLING AND RECORD KEEPING 78
15.1 Data Management 78
15.2 Record Retention in Study Sites 78
16 FINANCING AND INSURANCE 79
17 PUBLICATION POLICY 80
18 REFERENCES 81
19 APPENDICES 82
Appendix 1: Double-Blind Evaluation of UB-612 as a Heterologous Boost and BNT162b2 SARS CoV-2 Vaccine as a Homologous Boost. Sub Study to the Platform Protocol: A Phase 3 Multi-Center Platform Randomized, Active-Controlled Trial to Compare Homologous Boost of Authorized COVID-19 Vaccines and Heterologous Boost with UB-612 83

Appendix 2: Open-Label Evaluation of UB-612 as a Heterologous Boost and BNT162b2 SARS CoV-2 Vaccine as a Homologous Boost. Sub-study to the Platform Protocol: A Phase 3 Multi-Center Platform Randomized, Active-Controlled Trial to Compare Homologous Boost of Authorized COVID-19 Vaccines and Heterologous Boost with UB-612 85
Appendix 3: Double-Blind Evaluation of UB-612 as a Heterologous Boost and ChAdOx1-S SARS CoV-2 Vaccine as a Homologous Boost. Sub-study to the Platform Protocol: A Phase 3 Multi-Center Platform Randomized, Active-Controlled Trial to Compare Homologous Boost of Authorized COVID-19 Vaccines and Heterologous Boost with UB-612 87
Appendix 4: Open-Label Evaluation of UB-612 as a Heterologous Boost and ChAdOx1-S SARS CoV-2 Vaccine as a Homologous Boost. Sub-study to the Platform Protocol: A Phase 3 Multi-Center Platform Randomized, Active-Controlled Trial to Compare Homologous Boost of Authorized COVID-19 Vaccines and Heterologous Boost with UB-612 89
Appendix 5: Double-Blind Evaluation of UB-612 as a Heterologous Boost and BIBP COVID-19 Vaccine as a Homologous Boost. An Appendix to the Platform Protocol: A Phase 3 Multi-Center Platform Randomized, Active-Controlled Trial to Compare Homologous Boost of Authorized COVID-19 Vaccines and Heterologous Boost with UB-612 91
Appendix 6: Laboratory Abnormality Grading Scale 94
Appendix 7: List of Potentially Immune-Mediated Medical Conditions 95
Appendix 8: WHO Clinical Progression Scale 99

LIST OF TABLES

Table 1: Schedule of Assessments 15
Table 2: Study Objectives and Endpoints 30
Table 3: Clinical Laboratory Testing 51
Table 4: Local (Injection Site) Adverse Event Grading Scale 53
Table 5: Systemic Adverse Event Grading Scale 54
Table 6: Fever Grading Scale (Oral Temperature) 55
Table 7: CDC Interim Case Definition Criteria of COVID-19 56
Table 8: SARS-CoV-2 Infection Definitions for Exploratory Endpoints 57
Table 9: Active Collection Period for Adverse Events 64

LIST OF FIGURES

Figure 1: Platform Trial Design Flow Chart 33

1 SUMMARY OF CHANGES

1.1 Version History

Protocol version	Date	Global/Regional
Original V1.0	January 6, 2022	Global
Amendment 1 (V2.0)	February 15, 2022	Global
Amendment 2 (V3.0)	March 21, 2022	Global
Amendment 3 (V4.0)	May 12, 2022	US
Amendment 4 (V5.0)	June 7, 2022	Global
Amendment 5 (V6.0)	June 7, 2022	US
Amendment 6 (V7.0)	July 11, 2022	Global
Amendment 7 (V8.0)	August 15, 2022	Global
Amendment 8 (V9.0)	August 15, 2022	US
Amendment 9 (V10.0)	September 23, 2022	Global
Amendment 10 (V11.0)	September 23, 2022	US

1.2 Changes

Amendment to the Protocol Version 8.0 dated August 15, 2022			
**Protocol Section(s)**	**Previous Version**	**Current Version**	**Rationale**
Section 12.3 Interim Analysis	For each sub-study, an interim analysis is planned when at least 30% of subjects have been randomized and complete the Day 15 visit. The analysis will look at the difference in Day 15 log GMT between UB-612 and the comparator and the pooled standard deviation of the Day 15 log titers for an assessment of conditional power. Though the assessment will be made using Day 15 titers and not Day 29 titers that comprise the primary endpoint, the alpha spending will be set at 0.0001, leaving 0.0499 for the primary analysis. This interim analysis is purely administrative, and no action will be taken based on the results. The analysis will be conducted by the independent IDMC statistical team, reviewed by the IDMC, and the results confidentially reported directly to sponsor personnel not involved in study conduct decisions. The	There is no interim analysis planned for this study.	Due to the speed of enrollment, all subjects are expected to reach Day 29, the primary readout timepoint, prior to the planned interim analyses. Therefore, the interim analyses are superfluous and have been removed.

Amendment to the Protocol Version 8.0 dated August 15, 2022			
**Protocol Section(s)**	**Previous Version**	**Current Version**	**Rationale**
	details will be described in the Statistical Analysis Plan.		
Section 11.3.9 Independent Data and Monitoring Committee (IDMC)	An external, independent IDMC will be chartered to review the study data during the conduct of the trial, provide recommendation regarding safety which may impact the conduct of the trial, and review the interim analysis per protocol design.	An external, independent IDMC will be chartered to review the study data during the conduct of the trial, provide recommendation regarding safety which may impact the conduct of the trial.	Removed text related to interim analysis as there is no interim analysis planned (refer to row above).
Section 12.2 Power and Sample Size	The sample size is determined by each sub-study targeting at least 90% power with 1-sided alpha of 0.025 to test non-inferiority based on the GMT ratio using a margin ratio of GMT of 1.5.<br>The sample size calculation for the non-inferiority evaluation is based on the seronegative subjects, with an additional 25% of that sample size allocated for dropouts and major protocol deviations.	The sample size is determined by targeting at least 90% power (BNT162b2 Sub-studies and BIBP Sub-study) and 80 to 85% power (ChAdOx1-S Sub-studies) with 1-sided alpha of 0.025 to test non-inferiority based on the GMT ratio using a margin ratio of GMT of 1.5.<br>The sample size calculation for the non-inferiority evaluation is based on all subjects (seronegative and seropositive), with an additional 25% of that sample size allocated for dropouts and major protocol deviations in the BNT162b2 Sub-studies and BIBP Sub-study. No availability of unexpired ChAdOx1-S precluded attainment of the full planned enrolment. Therefore, the final sample size achievable does not account for dropouts for any reason.	No access to the unexpired supply of ChAdOx1-S vaccine in the countries of participating sites.
Section 12.2.2	For the primary endpoint of GMT ratio, assuming a log10 standard deviation of 0.40 (based on neutralizing antibodies vs alpha strain [Flaxman et al, 2021]), a sample size of 220 (110 per arm) will have 90% power to establish non-inferiority, with a non-inferiority margin of 1.5. A total of 276 subjects will be required enrolled to account for an additional 25% of that sample	For the primary endpoint of GMT ratio, assuming a log10 standard deviation of 0.40 (based on neutralizing antibodies vs alpha strain [Flaxman et al, 2021]), the achievable sample size in these sub-studies is approximately 190. This sample size will have 80 to 85% power to establish non-inferiority, with a non-inferiority margin of 1.5, if no subjects are lost prior to the	No access to the unexpired supply of ChAdOx1-S vaccine in the countries of participating sites.

Amendment to the Protocol Version 8.0 dated August 15, 2022			
**Protocol Section(s)**	**Previous Version**	**Current Version**	**Rationale**
	size allocated for dropouts and major protocol deviations.	assessment of the primary endpoint at Day 29.	
Table 1 Schedule of Assessments, footnote l	PBMC samples will be collected at selected sites from a sub-population (approximately 10% of total sample size, selected by principal investigators at participating sites based on a quota defined by Sponsor) for ICS and IFN-γ and IL-4 ELISpot assays. The subjects selected to provide PBMC should remain the same throughout the study visits.	PBMC samples will be collected at selected sites from a sub-population in BNT162b2 sub studies (approximately 10% of total sample size, selected by principal investigators at participating sites based on a quota defined by Sponsor) for ICS and IFN-γ and IL-4 ELISpot assays. The subjects selected to provide PBMC should remain the same throughout the study visits.	Laboratory supply constrains precluded from timely collecting PBMCs for sub-studies evaluating boosting in ChAdOx-1S and Sinopharm primed populations.

2 SYNOPSIS

2.1 Study Summary

CLINICAL STUDY SYNOPSIS	
Study Number	UB-612-305
Title of Study	A Phase 3 Multi-Center International, Platform Randomized, Active-Controlled Platform Trial to Compare Homologous Boost of Authorized COVID-19 Vaccines and Heterologous Boost with UB-612 Vaccine
Study Centers (Country)	Approximately 10 study centers (US, Mexico, Philippines, Panama, and other countries)
Development Phase	III
Objective	Primary Objectives.<br>• Safety: To evaluate the safety and tolerability of UB-612 and a comparator vaccine in subjects who have received primary immunization with a COVID-19 vaccine*<br>• Immunogenicity: To compare UB-612 vaccine to a comparator vaccine in the ability to boost short-term neutralizing antibody immunity against Wuhan strain measured in a live SARS-CoV-2 or pseudotyped virus assay, in subjects who have received primary immunization*<br>Secondary immunogenicity objectives.<br>• To compare UB-612 vaccine to a comparator vaccine in the ability to boost short-term neutralizing antibody immunity to the SARS-CoV-2 Omicron variant<br>• To evaluate the kinetics and duration of neutralizing antibodies to the SARS-CoV-2 Wuhan strain and Omicron variant or S1-RBD binding IgG antibodies after UB-612 or the comparator vaccine boosting dose<br>Exploratory immunogenicity objectives (in a subset of subjects).<br>• To evaluate the ability of UB-612 and a comparator vaccine to boost cellular immunity.<br>• To compare the ability of UB-612 vaccine or the comparator vaccine to boost Fc-mediated functional antibodies and neutralizing antibodies against additional SARS-CoV-2 variants<br>* Primary immunization is defined as 2 doses of a comparator vaccine spaced approximately 3-16 weeks apart. *Additional details are in Table 2.*
Methodology	This is a multicenter, international, randomized, active-controlled platform study with each sub-study designed to randomize subjects to receive a single injection with UB-612 or a comparator COVID-19 vaccines in 1:1 ratio (Figure 1). A double-blinded or an

	open label sub-studies were designed for BNT162b2 and ChAdOx1-S, while Sinopharm has only a double-blinded sub-study.<br><br>Subjects 16 years and older with completed primary immunization at least 3 months (Pfizer at least 5 months) prior to study vaccine injection will be randomized 1:1 into each treatment, stratified by age group (16-64 and 65+ years), gender, N-protein seropositivity, or the time since last dose of primary immunization. Participants will be screened for antibodies to the nucleoprotein (N) of SARS-CoV-2.<br><br>All subjects will be assessed for safety and immunogenicity after a single booster immunization with UB-612 or a comparator vaccine. All study participants will have up to 6 scheduled visits, including Screening, Day 1, 15, 29, Month 6, and 12, and safety calls on Day 8 and 57.<br><br>*Specific methodology details can be found in Section 7.*
Number of Patients	The proposed five sub-studies will enroll approximately 930 subjects in total. The open-label and double-blinded sub-studies evaluating BNT162b2 will enroll 400 subjects, ChAdOx1-S will enroll approximately 190 subjects, and the double-blinded Sinopharm sub-study will enroll 334 subjects.
Diagnosis and Main Criteria for Inclusion	To be eligible for the platform study subjects should satisfy main criteria:<br>• Males or females of childbearing potential, 16 years or older, willing to adhere to practice abstinence or use acceptable methods of contraception. Females subject of non-childbearing potential may be enrolled.<br>• Documented completed primary series with a comparator vaccine with the last dose administered at least 3 months (Pfizer at least 5 months) prior to Day 1 of the study.<br>• Healthy or with stable pre-existing medical condition.<br>Main exclusion criteria are the following:<br>• Known history of COVID-19 or SARS-CoV-2 infection within six (6) months prior to vaccination (Day 1).<br>• Receipt of a booster COVID-19 vaccination in addition to the primary vaccine series<br>• Presence of COVID-19 symptoms within 1 week prior to study Day 1<br>*Additional details on study population can found in Section 8.*
Test Product, Dosage, and Mode of Administration	• UB-612: single IM injection of 100 µg (0.5 mL)<br>• BNT162b2: single IM injection 30 µg (0.3mL)<br>• ChAdOx1-S: single IM injection 5x10^{10}vp (0.5mL)<br>• Sinopharm BIBP: single IM injection of 4 µg/6.5U (0.5 mL)
Duration of Treatment	The maximum duration of a subject's participation is 13 months.

Criteria for Evaluation	Safety and immunogenicity
Primary Endpoints	The primary safety endpoints in this study will be:<br>• Solicited adverse events (AEs): Day 1-8 after immunization<br>• Unsolicited AEs: Day 1- 29 days after immunization<br>• Serious adverse events, medically attended adverse events, and adverse events of special interest: throughout the study.<br>The primary immunogenicity endpoint is based on SARS-CoV-2 neutralizing antibody titers tested using replicating or pseudotyped virus (prototype Wuhan strain):<br>• Geometric mean antibody titer ratio (GMR) at Day 29
Secondary Endpoint	SARS-CoV-2 neutralizing antibody titers measured using replicating or pseudotyped virus, Omicron variant:<br>• GMR at Day 29<br>SARS-CoV-2 neutralizing antibody titers measured using replicating or pseudotyped virus, Wuhan and Omicron variants:<br>• Area under the curve (AUC) of neutralizing antibody from Day 15 to Month 12<br>• Neutralizing antibody geometric mean titer (GMT) on Days 15, 29, and Months 6 and 12<br>• Geometric mean fold increase (GMFI) in neutralizing antibodies titers from Day 1 to Day 15, Day 29, and Months 6 and 12<br>• Seroconversion rate (SCR), proportion of subjects with ≥4-fold antibody titer rise from Day 1 to Day 15, Day 29, and Months 6 and 12<br>• Distribution of neutralizing antibody titers determined on Day 29 and Month 6 and 12, displayed as reverse cumulative distribution curves by the treatment group and virus variant<br>SARS-CoV-2 immunoglobulin G (IgG) antibody titers measured by direct S1-RBD binding ELISA:<br>• GMT on Days 15, 29, and Months 6 and 12<br>• GMFI from Day 1 to Day 15 and Day 29, and Months 6 and 1<br>• SCR from Day 1 to Days 15 and 29, and Months 6 and 12<br>• Distribution of IgG antibody titers determined on Day 29 and Months 6 and 12, displayed as reverse cumulative distribution curves by the treatment group and virus variant<br>• AUC by the treatment group and the virus from Day 15 to Month 12
Exploratory Endpoint	• The number of cytokine secreting spots per million cells and % cells staining for cytokines at Days 1, 15 and 29, and Months 6 and 12

	• Fc-mediated antibody ADCP (antibody dependent cell-mediated phagocytosis) responses at Day 1 and Day 29<br>• SARS-CoV-2 neutralizing antibody titers measured using additional replicating variant live or pseudotyped viruses: GMT, GMR, GMFI, and SCR based on neutralizing titers determined at Day 1 and Day 29
Statistical Methods	The primary immunogenicity analysis will evaluate the non-inferiority of heterologous to homologous boost of GMT ratio immune responses.<br>Analysis of GMT ratio will be performed on the log-transformed (base 10) titers, a standard approach since log-transformation produces values that are approximately normally distributed. Non-inferiority is determined if the lower bound of the 2-sided 95.0% CI for the GMTR (GMT UB-612 group / GMT Comparator group) is >0.67. Analyses will be stratified by age group, gender, and the time since the last primary immunization dose.<br>The sample size is determined by targeting at least 90% power (BNT162b2 Sub-studies and BIBP Sub-study) and 80 to 85% power (ChAdOx1-S Sub-studies) with 1-sided alpha of 0.025 to test non-inferiority based on the GMT ratio using a margin ratio of GMT of 1.5.<br>The sample size calculation for the non-inferiority evaluation is based on all subjects (seronegative and seropositive), with an additional 25% of that sample size allocated for dropouts and major protocol deviations in the BNT162b2 Sub-studies and BIBP Sub-study. During the enrollment of the ChAdOx1-S Sub-studies, operational difficulties precluded attainment of the full planned enrollment. Therefore, the final sample size achievable does not account for dropouts for any reason<br>Safety endpoints will be analyzed descriptively by treatments per each sub study as well as pooled cross all sub studies. It will include solicited local and systemic AEs through 7 days after study product injection, unsolicited AEs through 29 days after study product injection, MAAEs, SAEs, and AESIs 12 months after study product injection.<br>*Additional details are described in the Section 12.*

2.2 Schedule of Assessments

An overview of the protocol visits and procedures is presented in Table 1. Refer to Section 11.2 for detailed information on each procedure and assessment required for compliance with the protocol.

The investigator may perform visits outside of the scheduled study visits (unscheduled visits) listed in the table to conduct evaluations or assessments required to protect the well-being of the subject.

Table 1: Schedule of Assessments

**Visit Number**		**1**	**1a****	**1b****	**1c****	**2**	**3**	**4**	**5**	**6**	**7**	**Unscheduled**		
**Visit Description**	**Screen-ing***	**Injection**	**Random-ization**	**Injection**	**Tele-phone**	**7-day Tele-phone**	**14-day F/Up**	**28-day F/Up**	**56-day tele-phone**	**6-month F/Up**	**12-month F/Up (EoS)**	**Un-scheduled visit**	**Sus-pected COVID-19 Illness[v]**	**COVID-19 Conva-lescence**
**Study Day**	**Day -28[a]**	**Day 1**	**Day 1a**	**Day 1b**	**Day 1c**	**Day 8**	**Day 15**	**Day 29**	**Day 57**	**Day 180**	**Day 361**	**As needed**	**As needed**	**As needed**
**Visit Window (Days)**	**+28d**	**-**	**+2-0d**	**-**	**-**	**±1 d**	**±1 d**	**±3 d**	**±3 d**	**±14 d**	**±14 d**	**-**	**-**	**-**
Informed consent/assent	X													
Inclusion/exclusion criteria	X	X	X											
Demographics	X													
Medical history	X													
Concomitant Medications	X	X	X			X	X	X	X	X	X	X	X	X
Physical examination	X	X[b]	X[b]				X[b]	X[b]		X[b]	X[b]	X[b]	X[b]	X[b]
Measure height and weight	X													
Vital signs[c]	X	X	X				X	X		X	X	X	X	X
Hematology, Chemistry[d,s]	X	X[u]	X				X	X						
Hemoglobin A1c [s]	X													
High sensitivity C-reactive protein (hs-CRP) [s]		X[u]	X				X	X						
Serology (HIV, HBsAg, HBcAb, HCV)[e, s]	X													
Confirm use of contraceptive	X	X	X			X	X	X						
Pregnancy test[f, s]	X	X	X				X	X						
SARS-CoV-2 RT-PCR and/or antigen test[g, s]	X	X	X										X[h]	
ECG 12 lead[x]	X						X							

**Visit Number**		**1**	**1a****	**1b****	**1c****	**2**	**3**	**4**	**5**	**6**	**7**	**Unscheduled**		
**Visit Description**	**Screen-ing***	**Injection**	**Random-ization**	**Injection**	**Tele-phone**	**7-day Tele-phone**	**14-day F/Up**	**28-day F/Up**	**56-day tele-phone**	**6-month F/Up**	**12-month F/Up (EoS)**	**Un-scheduled visit**	**Sus-pected COVID-19 Illness[v]**	**COVID-19 Conva-lescence**
**Study Day**	**Day -28[a]**	**Day 1**	**Day 1a**	**Day 1b**	**Day 1c**	**Day 8**	**Day 15**	**Day 29**	**Day 57**	**Day 180**	**Day 361**	**As needed**	**As needed**	**As needed**
**Visit Window (Days)**	**+28d**	**-**	**+2-0d**	**-**	**-**	**±1 d**	**±1 d**	**±3 d**	**±3 d**	**±14 d**	**±14 d**	**-**	**-**	**-**
Injection of study product		X		X										
30-minute post-injection observation [i]		X		X										
Provision/confirmation of emergency contact card		X	X			X	X	X	X	X	X	X		X
Blood draw SARS-CoV-2 antibody tests[j,t]		X[uw]	X[w]				X	X[w]		X	X		X[k]	X
Blood draw for PBMC (ELISpot & intracellular cytokine staining) [l,t]		X[u]	X				X	X		X	X			
Rapid N-protein antibody test	X[m1,s]													
N-protein antibody test		X[m2,t,u]	X[m2,t]				X [t]	X [t]		X [t]	X [t]		X[k,t]	X [t]
Provide thermometer, measuring device (ruler) and Diary[n]		X	X											
Explain communication methods (including Diary training) [n]	X	X	X			X	X	X	X	X	X	X		
Blood draw biorepository for future immunologic tests[t]		X[u]	X				X	X		X	X			
Solicited AEs[o]		X			X	X								
Unsolicited AEs[p]		X			X	X	X	X						
SAEs, MAAEs, AESI		X			X	X	X	X	X	X	X			

**Visit Number**		**1**	**1a****	**1b****	**1c****	**2**	**3**	**4**	**5**	**6**	**7**	**Unscheduled**		
**Visit Description**	**Screen-ing***	**Injection**	**Random-ization**	**Injection**	**Tele-phone**	**7-day Tele-phone**	**14-day F/Up**	**28-day F/Up**	**56-day tele-phone**	**6-month F/Up**	**12-month F/Up (EoS)**	**Un-scheduled visit**	**Sus-pected COVID-19 Illness[v]**	**COVID-19 Conva-lescence**
**Study Day**	**Day -28[a]**	**Day 1**	**Day 1a**	**Day 1b**	**Day 1c**	**Day 8**	**Day 15**	**Day 29**	**Day 57**	**Day 180**	**Day 361**	**As needed**	**As needed**	**As needed**
**Visit Window (Days)**	**+28d**	**-**	**+2-0d**	**-**	**-**	**±1 d**	**±1 d**	**±3 d**	**±3 d**	**±14 d**	**±14 d**	**-**	**-**	**-**
COVID-19 disease surveillance[q]		X	X			X	X	X	X	X	X	X	X	X
COVID-19 Convalescent Visit [r]														X

Abbreviations: AE = adverse event; AESI = adverse event of special interest; d=day; EoS = end of study; MAAE = medically attended adverse event; RT-PCR = real time-polymerase chain reaction; SAE = serious adverse event

* The screening and Day 1 visits can be combined assuming all assessments are completed, results received, and subject deemed eligible. All assessments should be completed prior to injection of study product, except for the post-dose observation vital signs.

** The events assigned to Visit 1a, 1b, and 1c represent an alternative Day 1 schedule applicable only to the subjects randomized to a comparator treatment in the open-label sub-studies.

Day 1a (Randomization): Occurs 0-48 hours prior to vaccination. Subjects randomized into a comparator treatment must complete all necessary assignments as described.

Day 1b (injection): Subjects randomized in a comparator treatment arm should independently identify a local vaccination location to receive injection with a comparator vaccine included in a relevant sub-study protocol. Subjects must secure comparator vaccine administration details (name, lot, expiration). Subjects administered study vaccine at the local vaccination location are to be observed post-vaccination as per standard institutional practice; vital signs are not collected. Diary should capture any self-reported observations within 30 (+/- 5) minutes after the injection.

Day 1c (telephone): On the same day after the injection, site staff checks on subject's status confirming administration of a comparator vaccine and any immediate AEs.

[a] Screening assessments can be conducted any time between Day -28 and Day 1 up to and including Day 1 prior to receipt of study injection.

[b] Targeted physical examination to be conducted if indicated by medical history or review of systems.

[c] Vital signs include blood pressure, heart rate, respiratory rate, and temperature.

[d] Complete blood count (Hgb, Hct, RBC, and reticulocytes), WBC with differential, platelets, prothrombin time (PT), partial thromboplastin time (PTT), international normalized ratio (INR), alanine aminotransferase (ALT), aspartate aminotransferase (AST), creatinine, and total and direct bilirubin. The hematology/chemistry tests performed during Screening within 48 hours prior to IP injection can be combined with Day1.

[e] Serology: HIV= human immunodeficiency virus; HBsAg = hepatitis B surface antigen; HBcAb = hepatitis B core antibody; HCV = hepatitis C. Serology can be performed within 48 hours prior to study vaccine injection on Day 1, including authorized rapid antibody test systems.

[f] Serum or urine pregnancy testing is required for WOCBP during Screening. A urine or serum pregnancy test will be done on the day of the study product injection as well as on Days 15 and 29. Pregnancy testing can be performed at a more frequent occurrence, upon investigator discretion and/or in accordance with local requirements.

[g] Saliva, nasopharyngeal, or nasal mid-turbinate swab is to be collected in accordance with the package instruction of the test used. The SARS-CoV-2 test can be performed at any time during the Screening, and if conducted within 48 hours window prior to Day 1, it does not need to be repeated on Day 1.

[h] Both antigen tests and RT-PCR can be used to confirm suspected COVID-19 illness, with RT-PCR being a preferred test. Antigen test results can be optionally confirmed with an RT-PCR test within 24 hours. Biological sample from suspected COVID-19 should be collected and stored for future viral sequencing, should SARS-CoV-2 infection be confirmed.

[i] 30 (±5) minutes post-injection. Vital signs (blood pressure, heart rate, respiratory rate and temperature) will be measured while seated and at rest. Applies to the subjects injected with a comparator or UB-612 vaccines at a research site only. Subjects injected with a comparator vaccine in vaccination centers in an open-label sub-study will be observed per the vaccination centers' protocol.

[j] Antibody tests for IgG S1-RBD ELISA, neutralization (Wuhan and Omicron). All subjects will be tested for up to 3 variants, including Wuhan and Omicron strains, and as required, additional SARS-CoV2 variants.

[k] If visit conducted at a research site.

[l] PBMC samples will be collected at selected sites from a sub-population in the BNT162b2 sub studies (approximately 10% of total sample size, selected by principal investigators at participating sites based on a quota defined by Sponsor) for ICS and IFN-γ and IL-4 ELISpot assays. The subjects selected to provide PBMC should remain the same throughout the study visits.

[m1] An authorized by sponsor rapid N-protein antibody test can be performed at any time during the screening period.

[m2] The presence of N-protein antibodies in the serum collected after the Screening should be analyzed via a sponsor directed central laboratory using a qualitative N-protein antibody test (Elecsys cobas Anti-SARS-CoV-2)except for the population immunized with the inactivated vaccine comparators, which will be tested by a quantitative N-protein ELISA.

[n] Subjects will be instructed (or study staff may assist) and trained on how to complete the subject Diary to record AEs and events requiring reporting to study staff.

[o] Collected daily via diary for first 7 days post-injection.

[p] Collected daily via diary for first 28 days post-injection.

[q] Throughout the duration of the study subjects with any suspected COVID-19 illness will be evaluated per institutional guidelines and Centers for Disease Control (CDC) recommendations.

[r] To be scheduled within 28 to 35 days from the Suspected COVID-19 Illness Visit.

[s] Laboratory test results are to be processed and reported locally.

[t] Laboratory test results are to be obtained, processed, stored, and sent to central laboratory for processing.

[u] Blood sample collection should be completed prior to study vaccine injection.

[v] Suspected COVID 19 illness visit, may be conducted via in person, telemedicine or via phone. If visit is conducted via telemedicine or via phone SARS testing will be conducted remotely.

[w] Sera for the ADCP test will be collected from a sub-population (approximately 10% of total sample size selected by principal investigators based on a quota defined by Sponsor) on Day 1 and Day 29. The subjects selected to provide the sera should remain the same.

[x] In those participants in whom tests are abnormal and suggestive of myocarditis or pericarditis, the participant will be recommended for cardiac consultation with evaluation and management following current practice guidelines (e.g., AHA), and functional cardiac evaluation (e.g., stress test echocardiogram) to detect potential late onset of cardiac function impairment associated with initially subclinical manifestation.

3 LIST OF ABBREVIATIONS

Abbreviation	Definition
ADCP	Antibody dependent cell-mediated phagocytosis
ADE	Antibody-dependent enhancement
AE	Adverse event
AESI	Adverse event of special interest
APC	Antigen-presenting cells
ARDS	Acute respiratory distress syndrome
AUC	Area under the curve
CDC	Centers for Disease Control
CI	Confidence interval
COVID-19	Coronavirus disease 2019
CpG1	CpG oligonucleotide
CRF	Case report form
CRO	Contract research organization
CTL	Cytotoxic T lymphocyte
DNA	Deoxyribonucleic acid
E	Envelope
ECG	Electrocardiogram
ECMO	Extracorporeal membrane oxygenation
ELISA	Enzyme-linked immunosorbent assay
ELISpot	Enzyme-linked immunosorbent spot assay
GCP	Good Clinical Practice
GMFI	Geometric mean fold increase
GMR	Geometric mean titer ratio
GMT	Geometric mean titer
hACE2	Human angiotensin-converting enzyme 2
HBsAg	Hepatitis B surface antigen
HCG	Human chorionic gonadotropin
HCV	Hepatitis C virus
HIV	Human immunodeficiency virus
HLA	Human leukocyte antigen
hs-CRP	High-sensitivity C-reactive protein
ICF	Informed consent form
ICH	International Council for Harmonisation
ICS	Intracellular cytokine staining

Abbreviation	Definition
IDMC	Independent Data Monitoring Committee
IEC	Independent Ethics Committee
IFN-γ	Interferon-gamma
IgG	Immunoglobulin G
IL	Interleukin
IM	Intramuscular
IP	Investigational Product
IRB	Institutional Review Board
IRT	Interactive Response Technology
ITT	Intent-to-treat
IU	International units
M	Membrane
MAAE	Medically attended adverse event
MERS	Middle East Respiratory Syndrome
MHC	Major histocompatibility complex
mRNA	Messenger ribonucleic acid
MVF	Measles virus fusion
N	Nucleocapsid
nAb	Neutralizing antibody
NHS	National Health Service
NIV	Non-invasive ventilation
O_2	Oxygen
PBMC	Peripheral blood mononuclear cell
PCR	Polymerase chain reaction
RBD	Receptor binding domain
RCDC	Reverse cumulative distribution curve
RNA	Ribonucleic acid
RSV	Respiratory syncytial virus
RT-PCR	Reverse transcriptase-polymerase chain reaction
S	Spike
SAE	Serious adverse event
SAP	Statistical Analysis Plan
SARS	Severe Acute Respiratory Syndrome
SARS-CoV-2	Severe acute respiratory syndrome coronavirus 2
SCR	Seroconversion rate
SOP	Standard operating procedure

Abbreviation	Definition
SP BIBP	Sinopharm BIBP inactivated COVID-19 vaccine
Th	T helper
UK	United Kingdom
VAERD	Vaccine-associated enhanced respiratory disease
WHO	World Health Organization
WOCBP	Women of childbearing potential
WONCBP	Women of non-childbearing potential

4 GENERAL INFORMATION

Role in Study	Name / Title	Address and Telephone Number
Sponsor's Medical Expert	Sasha Rumyantsev, MD, PhD, MBA<br>Therapeutic Area Head Infectious Diseases	Vaxxinity, Inc.<br>1717 Main St, Suite 3388<br>Dallas TX 75201<br>Mobile: 240-899-1304<br>Email: sasha@vaxxinity.com
Sponsor's Representative	Ulo Palm, MD, PhD, MBA<br>Chief Medical Officer	Vaxxinity, Inc.<br>1717 Main St, Suite 3388<br>Dallas TX 75201<br>Mobile (862) 296-6743<br>Email: Ulo@vaxxinity.com

5 INTRODUCTION AND RATIONALE

5.1 Disease Overview

The first cluster of cases of new coronavirus disease 2019 (COVID-19) caused by severe acute respiratory syndrome coronavirus 2 (SARS-CoV-2), were identified in Wuhan, China in late December 2019 (Wang, Horby et al. 2020). The outbreak was declared by the World Health Organization (WHO) as a Public Health Emergency of International Concern on 30 January 2020 and on 11 March 2020, WHO declared the outbreak had reached pandemic status. As of June 2022, the ongoing COVID-19 pandemic has caused over 530 million cases in all continents, over 6.3 million deaths and disrupted life and the global economy. Approximately 80% of infected patients showed asymptomatic or mild symptoms, 20% of them had severe symptoms requiring hospital admission, and 5% progressed to critical condition, especially those over 60 or with comorbidities. Although the mortality rate of COVID-19 is lower than Severe Acute Respiratory Syndrome (SARS) and Middle East Respiratory Syndrome (MERS) (1.04% vs 9.5% and 34.4%, respectively), the number of COVID-19-associated deaths has far outpaced those of SARS and MERS owing to the high transmissibility of SARS-CoV-2(Petrosillo, Viceconte et al. 2020). The serious global pandemic of COVID-19 and rising death tolls necessitate the urgent development of effective vaccines to continue to protect against SARS-CoV-2.

5.2 UB-612

Vaxxinity Inc. has developed a vaccine (UB-612) against SARS-CoV-2 that is designed to activate both humoral and cellular responses. UB-612 harnesses the power of “functional antigenics” through proprietary platform technologies. Functional antigenics means identifying highly immunogenic sites or regions within the protein sequence as targets, and then modifying these sequences to produce designer peptide/protein UBITh® immunogens in suitable formulations for a vaccine. The concept of functional antigenics has been validated in vivo by commercialization of veterinary vaccines against foot-and mouth disease virus and porcine reproductive and respiratory syndrome virus.

Briefly, SARS-CoV-2 consists of single, positive-stranded ribonucleic acid (RNA) that encodes for 29 proteins. Four of these proteins make up the actual structure of the virus: spike (S), membrane (M), envelope I, and a nucleocapsid (N) protein. The virus enters host cells through binding of the S protein to the human angiotensin-converting enzyme 2 (hACE2) receptor. The S protein is composed of two subunits, S1 and S2, with S1 containing the hACE2 receptor binding domain (RBD). Most of the vaccines currently in clinical studies target the S protein for induction of neutralizing antibody (nAb) response; however, substantial activation of CD4+ and CD8+ T cells and induction of effective nAb responses are all required for preventing infection and to facilitate the clearance of virus. It has been observed that both CD4+ and CD8+ T cells from recovered COVID-19 patients can recognize large pools of SARS-CoV-2 peptides derived from structural proteins S, M, N and other nonstructural proteins, and only 3 of 29 shared epitopes identified in SARS CoV-2 across the 6 common human leukocyte antigen (HLA) types are in the S protein. Therefore, it appears likely that vaccines containing immunodominant SARS-CoV-2 epitopes derived from other viral proteins, and not just the S protein, could allow generation of balanced B cell and broad T cell responses, making a more efficacious vaccine. UB-612 is based on this multi-epitope approach.

UB-612 contains an RBD-sFc fusion protein, formulated with designer T helper (Th) and cytotoxic T lymphocyte (CTL) epitope peptides selected from S2, M, and N regions of the

virus. The formulation also includes an extrinsic major histocompatibility complex (MHC) class II epitope (UBITh®1a), modified from measles virus fusion (MVF) protein. The Th and CTL peptides are highly potent epitopes that are known to bind to human MHC I and II, which would allow for induction of memory recall and T-cell activation and effector functions. In a preliminary study in guinea pigs, the RBD-sFc vaccine induced high levels of neutralizing antibodies that were effective in inhibiting ACE2 receptor: S1-RBD interactions, with neutralizing activity far exceeding the levels observed in high titer convalescent sera from patients with COVID-19.

In addition to the multi-epitope design, the immunogen components are combined in the presence of an oligodeoxynucleotide containing unmethylated CpG motifs (e.g., CpG1), which bind to the positively charged designer immunogens through charge neutralization to form immunostimulatory complexes. The maximum amount of CpG1 in a dose is 2 µg, which is significantly lower than in the licensed hepatitis B vaccine (HEPLISAV®) containing 3000 µg/dose. The immunostimulatory complexes are added to Adju-Phos (a Th2 adjuvant), which improves the attraction and uptake of these immunogens by antigen-presenting cells (APC) and upregulates immunity via the inflammasome pathway. The newly formulated UB-612 vaccine containing a combination of RBD-sFc and 6 designer peptides (Th/CTL/UBITh®1a) as core immunogen is designed to elicit a balanced humoral and cellular immunity to prevent SARS-CoV-2 infections and reduce severity of acute respiratory distress syndrome (ARDS) and hospitalization requirements.

5.2.1 Summary of Nonclinical Findings

A detailed summary of the UB-612 nonclinical development program is provided in the Investigator's Brochure (UB-612 Investigator's Brochure).

5.2.2 Clinical Experience

A detailed summary of the UB-612 clinical development program is provided in the Investigator's Brochure (UB-612 Investigator's Brochure).

5.3 Study Rationale

A severe respiratory disease was first reported in Wuhan, China in December 2019. In January 2020, the etiologic agent was identified as a new coronavirus closely related to SARS virus and subsequently named SARS-CoV-2. The outbreak was declared by the WHO as a Public Health Emergency of International Concern on 30 January 2020 and on 11 March 2020, WHO declared the outbreak had reached pandemic status. As of June, 2022, the ongoing COVID-19 pandemic has caused over 530 million cases in all continents, over 6.3 million deaths and disrupted life and the global economy.

There are currently 24 vaccines approved in at least in one country for prevention of COVID-19 disease and 194 countries implemented vaccination. Over 56% of population received at least one dose of vaccine against COVID-19, and approximately 7.6 billion doses of these vaccines have been deployed to date. However, development, approval and distribution of additional safe, affordable, and effective SARS-CoV-2 vaccines remain of critical importance to control the pandemic globally where >6 billion people are exposed. Moreover, the emergence of variant strains of concern of SARS-CoV-2, some of which (e.g., Omicron) may reduce the neutralization activity and efficacy of current vaccines.

The immunity stimulated by authorized primary series vaccines was found to be not long lived, with the elderly and immunocompromised individuals impacted by the rapid antibody decay the most. Moreover, because of the decrease in neutralizing activity induced by current vaccines against the aforementioned variant strains, it may be useful to boost neutralizing antibody levels to offset the decrease in titer. Recently to counter the decreased efficacy against viral variants, many vaccines were authorized to add additional doses to the populations at risk. Numerous countries have introduced policy changes recommending an additional booster to restore protective immunity in the most vulnerable populations. The additional doses were typically recommended as soon as 3 months after the previous immunization.

In addition to vaccines currently authorized, there is an expectation of more to follow. This creates the practical difficulty of matching booster doses to the original vaccine used for primary vaccination. It will therefore be important to determine whether an alternative vaccine against COVID-19 can be used to boost immunity, in particular with respect to overcoming the decreased neutralizing activity against variant strains. Some COVID-19 vaccine combinations were already shown to be more reactogenic than others. Therefore, it will be also important to understand the safety and tolerability of an alternative COVID-19 vaccine used in heterologous boosting. As vaccine coverage increases, immunization against COVID-19 will increasingly be indicated for maintaining immunity through booster doses given annually or at an interval to be determined. It will be important to create an arsenal of COVID-19 vaccines with optimal and balanced safety and immunogenicity profile in the population with diverse immunity against SARS-CoV-2.

Vaxxinity has developed a vaccine (UB-612) against SARS-CoV-2 that is designed to activate both humoral and cellular responses. UB-612 contains a recombinant spike protein, S1 subunit receptor binding domain linked to a single chain Fc fragment of human immunoglobulin G 1 (IgG1) (S1-RBD-sFc) formulated with synthetic peptide Th and cytotoxic T lymphocyte (CTL) epitopes selected from M, S2, and N regions of the virus. The formulation includes aluminum phosphate adjuvant. CpG1 is used primarily as an excipient to bind together the peptides via charge interactions; whereas CpG1 has adjuvant activity in appropriate concentrations, the level in the vaccine is likely too low to serve as an immune stimulant. Studies in animal models have shown generation of high levels of IgG and neutralizing antibodies, and a Th1 oriented cytokines and T cell responses. The composition of UB-612 includes key antigenic determinants, such as S1-RBD and S2 Th/CTL epitopes, that are present in all currently authorized vaccines. Therefore, boosting with UB-612 should be able to restore not only neutralizing antibodies but also protective T-cells generated by the primary immunization with the authorized vaccines.

In a Phase 1 open-label clinical study in 60 healthy adults 20-55 years of age, 20 of whom received two vaccinations of either 10, 30 or 100 µg at 28-day intervals, UB-612 was well tolerated, with a relatively low frequency of mild local and systemic adverse events (Protocol V-122). Importantly, adverse events were not increased on the second vaccination. The vaccine elicited RBD binding IgG antibodies and neutralizing antibodies levels that exceeded or were equivalent, respectively, to binding and neutralizing antibodies in convalescent sera from COVID-19 patients. Importantly, the antibody response was surprisingly durable, with a half-life of 195 days, substantially longer than reported for other vaccines.

A Phase 2 placebo-controlled, randomized, observer-blind study evaluated the immunogenicity, safety, and tolerability of UB-612 vaccine against COVID-19 in approximately 3,850 adolescents, younger and elderly adults in Taiwan (Protocol V-205).

Within this population, approximately 350 participants were adolescents, 2800 were adults 18-64 years and 700 were adults >65 years. The results indicated that the vaccine was safe and well-tolerated. Immunogenicity in younger adults was similar to that observed in Phase 1, and somewhat decreased in elderly subjects. Details of the V-122 and V-205 studies are provided in the Investigator's Brochure.

To investigate the ability of a booster dose of UB-612 to boost prior vaccine responses, 50 re-consented subjects from the Phase 1 study received a third dose of 100 µg UB-612 on days 227-288 after the second immunization. The booster dose stimulated very high levels of neutralizing antibodies exceeding those observed in all subjects after primary immunization by over 40-fold.

The RBD antigen is a component of all COVID-19 vaccines. In addition, most COVID-19 vaccines also contain or express the S2 portion of the spike protein which is represented in UB-612 by three peptides against epitopes in S2. Therefore, it is it is expected that prior vaccination with a heterologous vaccine will have established immunologic memory to the RBD and S2 antigens in UB-612 and that a booster dose of UB-612 will elicit an anamnestic immune response.

The current platform protocol is designed to determine the safety and immunizing activity of a booster dose of 100 µg UB-612 in patients who have received a different vaccine 3 months or more before the study start (i.e., Day 1). The randomized, active-controlled multicenter study sponsored by Vaxxinity will be conducted in several countries in under a master platform protocol outlining common objectives, endpoints, population, study design, and data analysis. The platform protocol is designed for multiple sub-studies to be implemented at any time, each addressing independently the same set of scientific questions aimed to evaluate the immune responses after a booster injection with UB-612 SARS CoV-2 vaccine and a particular comparator SARS CoV-2 vaccine product. Comparator COVID-19 vaccines included in the current protocol are authorized under the Emergency Use or have limited supply controlled by National and local health authorities. Therefore, during the ongoing SARS-CoV-2 outbreak, the sponsor and clinical trial sites participating in the multicenter study might have uneven access to certain comparator vaccines which may limit their evaluation performed in a double-blinded fashion. The immune responses measured under the current protocol are objective and, thus, are not expected to be influenced by the blinding procedures. Therefore, to evaluate comparator vaccines not available for dosing in a double blinded fashion at participating research sites, the platform protocol incorporates open-label sub-studies. In open-label sub-studies, after randomization to a comparator treatment, subjects will be requested to independently identify local vaccination centers to receive a standard of care matching to the assigned comparator vaccine. Safety from the sub-studies evaluating the same comparator will be analyzed by taking in account the blinded and open-label nature of the data.

All subjects in the study will be evaluated for non-inferiority of neutralizing antibody response to the Wuhan and Omicron variants, and a subset analysis will measure the neutralization of additional SARS-CoV-2 variants.

Multiple measures of the immune response to COVID-19 vaccines are used in this study to indirectly evaluate the effect of boosting. Neutralizing antibodies are considered to be the mediator of protection, and therefore constitute the primary endpoint. Escape from neutralizing antibodies due to mutation of the virus and selection of resistant variant strains is a recognized problem for the use of vaccines and will be assessed by measuring neutralizing antibodies to the major variants of interest and concern. T cells, particularly CD8+ cytotoxic T cells, are

involved in clearance of infected cells and recovery from infection. T cells are required for immunologic memory and helper functions for antibody responses. Therefore, T cell responses will be evaluated, and the quality of the T cell response assessed by measuring Th1 and Th2 cytokine release. Finally, exploratory studies will evaluate non-neutralizing but potentially important functional antibodies that mediate cellular anti-viral activities via Fc receptors, such as ADCP (antibody dependent cell-mediated phagocytosis).

5.4 Risk/Benefit Assessment

5.4.1 Potential Benefit

Preliminary clinical data from a Phase 1 Study (ClinicalTrials.gov Identifier: NCT04545749) show UB-612 to be very well tolerated and immunogenic. All available safety, tolerability and immunogenicity data identify the 100 µg UB-612 dose as safe, well-tolerated, immunogenic, and suitable for continued development. In addition, nonclinical data showed UB-612 had no toxicities in a Good Laboratory Practice repeat dose study in rats and elicited strong neutralizing antibodies and Th1-oriented cellular immunity in rodent models. In a mouse model transduced to express the hACE2 receptor, UB-612 provided solid protection against challenge with SARS-CoV-2, and in a non-human primate model significantly reduced viral load (UB-612 Investigator's Brochure). The nonclinical data showing safety, immunogenicity, and efficacy support the potential of the vaccine to have similar characteristics in human studies.

Due to the decrease in neutralizing activity induced by the currently available vaccines against variant strains of SARS-CoV-2, many countries have implemented policy to re-vaccinate those already vaccinated to boost neutralizing antibody levels to offset the decrease in titer.

Subjects participating in this study all will receive a “booster” vaccination with a COVID-19 vaccine, either the investigational UB-612 vaccine or another authorized COVID-19 vaccine. Those receiving booster vaccines will potentially have their immune response to the SARS-CoV-2 virus boosted and be better protected, reduce disease severity, increase longevity of immunity, and overcome reduced neutralization to variant strains.

5.4.2 Potential Risks

5.4.2.1 Novel CpG Excipient

The vaccine antigens composed of recombinant subunit protein and synthetic peptides are not expected to have any toxicities. The vaccine contains a small amount of a novel Type B CpG (designated CpG1), a 32-mer oligodeoxynucleotide (ODN) with a phosphothiorate backbone. The amount of CpG1 in the highest dose used in the Phase 1 study (100 µg) of UB-612 is 2 µg, which is significantly less than the 3,000 µg of CpG 1018 22-mer ODN in the approved HEPLISAV-B® hepatitis B vaccine. CpG1 has been administered in several vaccine studies, including 2 studies under US IND (IND #17481). The low concentration of CpG1 in a dose of UB-612, the similarity to an oligodeoxynucleotide adjuvant in an approved product with similar mode of action (TLR-9 activation), and the tolerability in clinical studies of another peptide vaccine containing CpG1, make it unlikely that CpG1 presents a safety concern. Despite the low concentration of CpG1, AESIs associated with the use of novel adjuvants will be monitored in the study.

UB-612 contains aluminum phosphate adjuvant. Aluminum-containing adjuvants are widely used in multiple approved vaccines and has a long record of safety. In rare cases, aluminum adjuvants have been associated with chronic post-vaccination macrophagic myofascitis

syndrome, but a causal link has not been proven. This syndrome and its characteristic clinical presentation of myalgia, arthralgia, weakness, fatigue, and muscle tenderness are listed under AESIs.

5.4.2.2 Immunotoxicity

A hypothetical risk associated with UB-612 is the possibility of antibody-dependent enhancement (ADE), an *in vitro* phenomenon for which there is no direct evidence that it plays a role *in vivo* or in vaccine-associated enhanced respiratory disease (VAERD) in vaccinated subjects exposed later to the wild-type virus.

VAERD was observed in humans immunized with formalin-inactivated respiratory syncytial virus (RSV) and measles vaccines. It was also observed in animals immunized with formalin-inactivated SARS-CoV-1 vaccine. To date, no evidence for this phenomenon has been found for SARS-CoV-2 infections and in fact, all vaccines that have been tested for efficacy have shown protection against severe disease rather than enhancement.

Based on data from RSV, VAERD is believed to be an immunopathologic disease mediated by dysregulated T cell responses biased towards Th2, as well as immune complex deposition and associated inflammation. Consequently, a goal in coronavirus vaccination is to orient the immune response towards a Th1 bias. In the Phase 1 clinical study of UB-612, a balanced Th1/Th2 response determined by the ratio of IFN-γ to IL-4 secreting cells was seen for CD4+ lymphocytes, whereas there was a Th2 bias for CD8+ T cells. In the recent Phase 2 study of UB-612, a strong Th1 orientation of the T cell response was documented in both enzyme-linked immunosorbent spot assay (ELISpot and intracellular cytokine staining assays.

The Th1 bias of UB-612 responses in this Phase 3 study will be evaluated by performing enzyme-linked immunosorbent spot assay (ELISpot) assays for interferon-gamma (IFN-γ) (Th1) and IL-4 (Th2) and intracellular cytokine staining for IFN-γ, interleukin (IL)-2 (Th1), IL-4 (Th2) cytokines. The ratio of IFN-γ and IL-4 responses will be measured.

5.4.2.3 Local and Systemic Adverse Events

Local injection site AEs such as pain/tenderness, itching, redness or swelling, and systemic AEs such as fever, chills, malaise, fatigue, headache, anorexia, nausea, vomiting, diarrhea, myalgia, and arthralgia are common AEs observed with many vaccines. These symptoms were mild to moderate in severity in the Phase 1 study and were self-limited.

In this study, subjects will be observed by a trained site staff member in the clinic for at least 30 (±5) minutes after each vaccination. Local and systemic AEs will be closely monitored using a diary that will allow capture of AEs starting on Day 1. An Independent Data Monitoring Committee (IDMC) will be chartered to review all AEs and provide recommendations.

5.4.2.4 Unknown Adverse Events and Laboratory Abnormalities Associated with a Novel Vaccine

Local and systemic AEs will be closely monitored using a subject diary to report of AEs. Rules for discontinuing treatment in individual subjects and for halting the study based on conservative reporting of AEs will be implemented. In addition to clinical symptoms and signs, laboratory tests (hematology, chemistry, and high-sensitivity C-reactive protein (hs-CRP) [a marker of inflammation]) will be monitored after the study product injection.

Immediate systemic allergic reactions (e.g., anaphylaxis) can occur following any vaccination. These reactions are very rare and are estimated to occur once per 450,000 vaccinations for

vaccines that do not contain allergens such as gelatin or egg protein (Zent et al 2002). Although immediate systemic allergic reactions have not occurred with UB-612, subjects will be observed by a trained site staff member in the clinic for 30 (±5) minutes post-study product injection and reassessed prior to discharge.

5.4.2.5 Risks for Pregnancy

UB-612 is not considered likely to be unsafe for pregnant women or the developing fetus. Pregnant women will be excluded, and the requirement for use of acceptable contraceptives will be applied in the study. Due to the unknown risks to nursing infants, lactating mothers also are excluded.

5.4.2.6 Other Potential Risks Associated with the Study

Subjects will be required to attend healthcare facilities for scheduled assessments, phlebotomy, and assessment of illness during the ongoing SARS-CoV-2 pandemic. Social distancing and personal protective equipment will be required to reduce the potential for infection with SARS-CoV-2. The sites of the participating countries will be required to follow the local and/or national recommendations for SARS-CoV-2 control and for limiting subject exposure to SARS-CoV-2. Telephone visits will be used when there is no requirement for obtaining blood samples or diagnostic polymerase chain reaction (PCR) specimens.

Venipuncture will be performed during the study. (The maximum amount of blood to be collected per visit and during the study are specified in the informed consent/assent form.) There is the risk of bleeding, bruising, hematoma formation, and infection at the venipuncture site. Experienced and qualified personnel will perform the blood draws. Blood draws will not exceed national guidelines for blood donation.

More detailed information about the known and expected benefits and risks and reasonably expected AEs is found in the Investigator's Brochure.

5.5 Risk of Myocarditis and Pericarditis Associated with COVID-19 Vaccines

With the availability of several FDA-approved COVID-19 mRNA vaccines, myocarditis and pericarditis have been reported. The greatest numbers have been reported in males under the age of 40 years following a second dose of mRNA vaccines, but cases have been reported in older males and in females as well, and also following other doses. The observed risk is highest in males 12 to 17 years of age. While some cases required intensive care support, available data from short-term follow-up suggest that symptoms resolve in most individuals with conservative management. Information is not yet available about potential long-term sequelae.

6 OBJECTIVES AND ENDPOINTS

The study objectives and endpoints are summarized in Table 2.

Table 2: Study Objectives and Endpoints

PRIMARY	
**Primary Safety Objective**	**Primary Safety Endpoint**
• To evaluate the safety and tolerability profiles of UB-612 and a comparator vaccine in subjects who have received primary immunization with a COVID-19 vaccine at least 3 months or more before the study start	• Injection site adverse events (AEs) for up to 7 days following receipt of the study vaccine<br>• Prompted systemic AEs for up to 7 days following receipt of the study vaccine<br>• Unsolicited AEs following receipt of the study vaccine to 28 days after the dose<br>• Serious adverse events (SAEs), medically attended adverse events (MAAEs) and adverse events of special interest (AESIs) from receipt of the study vaccine to 12 months after the dose
**Primary Immunogenicity Objective**	**Primary Immunogenicity Endpoints**
• To compare UB-612 vaccine to a comparator vaccine in the ability to boost short-term neutralizing antibody immunity against SARS-CoV-2, Wuhan strain, who have received primary immunization[1] with the same comparator vaccine at least 3 months or more before the day of immunization	• SARS-CoV-2 neutralizing antibody titers measured using replicating or pseudotyped virus (prototype Wuhan strain)<br>– Neutralizing antibody geometric mean titer ratio (GMR) at Day 29 post-boost<br>–
**SECONDARY**	
**Secondary Immunogenicity Objectives**	**Secondary Immunogenicity Endpoint**
• To compare UB-612 vaccine to a comparator vaccine in the ability to boost short-term neutralizing antibody immunity to the SARS-CoV-2 Omicron variant of concern (VOC)<br>• To evaluate the kinetics and duration of humoral immunity to the SARS-CoV-2 Wuhan strain and Omicron variant after UB-612 or the comparator vaccine boosting dose	SARS-CoV-2 neutralizing antibody titers measured using replicating or pseudotyped virus, Omicron variant<br>• Neutralizing antibody geometric mean titer ratio (GMR) at Day 29 post-boost<br>SARS-CoV-2 neutralizing antibody titers measured in Vero cells using replicating or pseudotyped virus, Wuhan and Omicron variants<br>• Area under the curve (AUC) of neutralizing antibody response by treatment group and virus variants from Day 15 to Month 12<br>• Neutralizing antibody geometric mean titer (GMT) on Days 15, 29, and Months 6 and 12 post-boost<br>• Geometric mean fold increase (GMFI) in neutralizing antibodies titers from before study product injection on Day 1 to Day 15, Day 29, and Months 6 and 12 post-boost<br>• Proportion of subjects with ≥4-fold rise of neutralizing antibodies from before study product injection to Days 15 and 29, and Months 6 and 12 post-boost<br>• Distribution of neutralizing antibody titers determined on Day 29 and Month 6 and 12 post-

	boost, displayed as reverse cumulative distribution curves by the treatment group and virus variant<br>SARS-CoV-2 immunoglobulin G (IgG) antibody titers measured by direct S1-RBD binding ELISA<br>• IgG antibody GMT on Days 15, 29, and Months 6 and 12 post-boost<br>• GMFI in of IgG antibodies titers from before study product injection on Day 1 to Day 15 and Day 29, and Months 6 and 12 post-boost<br>• Proportion of subjects with ≥4-fold rise of IgG antibodies from before study product injection to Days 15 and 29, and Months 6 and 12 post-boost<br>• Distribution of IgG antibody titers determined on Day 29 and Months 6 and 12 post-boost, displayed as reverse cumulative distribution curves by the treatment group and virus variant<br>• Area under the curve (AUC) of IgG antibody response by the treatment group and the virus variant from Day 15 to Month 12 post-boost
**EXPLORATORY**	
**Exploratory Objectives**	**Exploratory Endpoints**
• To evaluate the ability of UB-612 and a comparator vaccine to boost cellular immunity in a subset of subjects	The number of cytokine secreting spots per million cells and % cells staining for cytokines at Days 1, 15 and 29, and Months 6 and 12 post-boost<br>• Interferon-gamma (IFN-γ) and interleukin (IL)-4 secreting cells/10^6 cells in enzyme-linked immunosorbent spot (ELISpot) assays and intracellular cytokine staining (ICS) for IFN-γ, IL-2, IL-4, IL-10, tumor necrosis factor-alpha (TNF-α), and granzyme B measured against the protein and pooled peptide components separately
• To compare the ability of UB-612 vaccine or the comparator vaccine to boost humoral immunity in a subset of subjects	• Fc mediated (non-neutralizing) functional antibody responses at Day 1 (baseline) and Day 29 post-boost<br>– ADCP (antibody dependent cell-mediated phagocytosis)<br>• SARS-CoV-2 neutralizing antibody titers measured using replicating additional variant live or pseudotyped viruses.<br>– GMT, GMR, GMFI, and seroresponse rates based on neutralizing titers determined at Day 1 (baseline) and Day 29 post-boost

1 Primary immunization is defined as 2 doses spaced approximately 3-16 weeks apart. The time interval to booster is 3 months or more, taking into consideration local and national regulations, is described in detail in a relevant sub-study section linked to the master protocol.

6.1 Surveillance for Enhanced COVID-19

The study is not designed to detect imbalances in COVID-19 incidence and severity between treatment arms. However, during the course of the study, confirmed COVID-19 cases will be followed per protocol, per local standard of care and captured in the case report form (CRF).

6.2 Surveillance for Myocarditis and Pericarditis

Case definition criteria for myocarditis or pericarditis is provided in Centers for Disease Control and Prevention (CDC) case definition criteria, (https://www.cdc.gov/mmwr/volumes/70/wr/mm7027e2.htm) or the Brighton Collaboration case definition (https://brightoncollaboration.us/wp-content/uploads/2021/11/Myocarditis-and-Pericarditis-manuscript_validated.pdf).

Participants reporting acute chest pain, shortness of breath, palpitations, or other signs or symptoms of myocarditis or pericarditis within 4 weeks after vaccination must be referred to a cardiologist for evaluation and management.

Cases of myocarditis or pericarditis occurring within 4 weeks of vaccination are considered to be potentially related, unexpected, and serious (as important medical events, even if other seriousness criteria are not met) and therefore, to meet criteria for expedited reporting, as required by 21 CFR 312.32. [THIS WILL NOT APPLY FOR PFIZER COVID VACCINE for which myocarditis/ pericarditis is labelled/ expected.

Cases of myocarditis and pericarditis will be followed until resolution of symptoms and abnormal test findings. Monitoring for subclinical myocarditis/ pericarditis will be carried out for all participants. This will consist of ECGs testing at baseline and at Day 15 post-vaccination In those participants in whom tests are abnormal and suggestive of myocarditis or pericarditis, the participant will be recommended for cardiac consultation with evaluation and management following current practice guidelines (e.g., AHA), and functional cardiac evaluation (e.g., stress test echocardiogram) to detect potential late onset of cardiac function impairment associated with initially subclinical manifestation.

7 STUDY DESIGN

7.1 Overall Study Design

This is a multicenter, international, randomized, active-controlled platform study to evaluate the ability of UB-612 vaccine to boost immunity in subjects 16 years of age and older who previously received a COVID-19 vaccine primary series. Data from subjects who receive an authorized COVID-19 vaccine will be compared with data from subjects who receive UB-612 vaccine as a booster vaccine (Figure 1). The study will be conducted in conformance with Good Clinical Practices (GCP) at multiple sites internationally.

Figure 1: Platform Trial Design Flow Chart

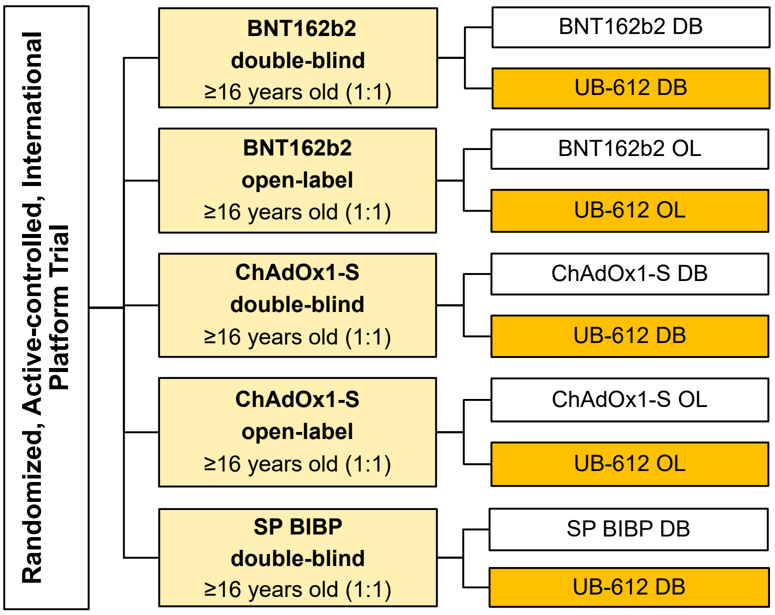

Under this platform study, each sub-study is designed to randomize subjects to receive either an authorized COVID-19-vaccine or UB-612 vaccine as shown in Figure 1 following the overall study objectives, investigation and analysis plans. A double-blinded or an open label sub-study is designed for several specific comparator vaccines, i.e., BNT162b2 and ChAdOx1-S. With double blinded studies being a priority, open-label sub-studies enable recruitment at participating sites that otherwise do not have capabilities to administer comparators vaccines in a double-blinded fashion. The study will enroll a required number of subjects, as determined by the sub-study, who completed a primary vaccine series with an authorized comparator vaccine at least three (3) months prior to Day 1, taking into consideration the local and national regulations. Subjects in each sub-study will be randomized with 1:1 ratio to each treatment and stratified by the following factors: age group (16-64 vs. ≥65 years old), gender at birth (male vs female), N-protein seropositivity at Screening (positive vs. negative), and the time since the last primary immunization dose (≥3-<5 months, vs ≥5 months). In places where country or state jurisdictions define the start of legal age of adulthood different than 16 years, an assent is required to enroll minor subjects between 16 years and the age of adulthood in accordance with the local laws and regulations.

All subjects will be assessed for safety and immunogenicity endpoints after a single booster immunization with UB-612 or a comparator vaccine. All study participants will have up to 6 scheduled visits, including Screening, Day 1, 15, 29, Month 6, and 12, and safety calls on

Day 8 and 57. Participants will be provided with Diaries to report solicited adverse events (AEs) for 7 days and unsolicited AEs for 28 days after a booster vaccine administration. All participants will provide serum samples collected at the scheduled visits for RBD-binding IgG antibodies and neutralization test against Wuhan and Omicron variants. Subjects will be tested for up to 3 variants, including Wuhan, Omicron strains, and as required, additional SARS-CoV2 variants. Approximately 10% of subjects in the Pfizer sub-studywill provide peripheral blood mononuclear cell (PBMC) samples to measure vaccine specific T-cell responses.

7.1.1 Rules for Suspension or Pause of the Study

Safety will be continuously monitored throughout the study as defined in the Safety Monitoring Plan. This safety monitoring will include the review of all AEs, including severe (Grade 3), serious, medically attended adverse events (MAAEs), and AESIs. Moreover, if any of the following events are reported, they will be reviewed in a timely manner by the Medical Monitor and other sponsor personnel in conjunction with the investigator:

- A serious adverse event (SAE) that is assessed by the investigator as possibly, probably or definitely related, or for which there is no alternative, plausible, attributable cause.
- A Grade 4 local AE, systemic AE, or fever that is assessed as possibly, probably or definitely related by the investigator, or for which there is no alternative, plausible, attributable cause.
- An AESI assessed as possibly probably or definitely related by the investigator, or for which there is no alternative, plausible, attributable cause.
- If 2 of the same SAEs or Grade 4 local or systemic AEs or if a single AESI occurs,
- An imbalance in the occurrence of SAEs reported for subjects randomized into the different stratum or treatment groups. Licensed comparator product will be monitored for new safety concerns which may limit the use in respective sub-studies
- Authorized comparator product will be monitored for new safety concerns which may limit the use in respective sub-studies

If any of the above occurs, the Medical Monitor and other sponsor personnel, and the investigator, will review all relevant safety data to determine whether any additional information is required. Based upon this review, the Medical Monitor and sponsor personnel will determine whether the study should be paused. If the decision is made to pause the study, no new subjects will be vaccinated until it has been determined that it is safe to continue.

7.1.2 Restarting After Suspending or Pausing the Study

In the event the study is paused to allow a thorough safety review to be conducted, the following actions will be taken:

- Randomization and administration of study product to new study subjects will be paused for all treament groups.
- For subjects who already received study product, all other routine study conduct activities, including ongoing data entry, reporting of AEs, Diary completion, blood sample collection, and subject follow-up, will continue during the pause.

- Diary data confirmed by the investigator as having been recorded in error will not contribute toward a pausing or stopping vaccinating new subjects.

After the review of all relevant safety data, the Medical Monitor and other Sponsor personnel, in conjunction with IDMC and the investigator, will reach consensus on how the study will proceed.

7.2 Scientific Rationale for Study Design

Booster vaccinations have been recommended in over 70 countries by health authorities to people most at risk from COVID-19 who completed primary series of a vaccine at least 3 months ago.

Randomization is being used to minimize bias in the assignment of subjects to treatment groups, to increase the likelihood that known and unknown subject attributes (e.g., demographic and baseline characteristics) are evenly balanced across treatment groups and enhance the validity of statistical comparisons across treatment groups.

A Platform design is considered to enable the comparison of UB-612 to each licensed vaccine individually in a sub-study setting under the same overall study objectives, investigation plan and analyses. This will ensure the consistency, improve the quality and efficiency cross all sub-studies.

Multiple measures of the immune response to COVID-19 vaccines are used in this study to indirectly evaluate efficacy and the effect of boosting. Neutralizing antibodies are considered to be the mediator of protection, and therefore constitute the primary endpoint. Escape from neutralizing antibodies due to mutation of the virus and selection of resistant variant strains is a recognized problem for the use of vaccines and will be assessed by measuring neutralizing antibodies to the major variants of interest and concern. Finally, exploratory studies in a subject of subjects will evaluate non-neutralizing but potentially important functional antibodies that mediate cellular anti-viral activities via Fc receptors.

7.3 Justification for Dose

Subjects in the Phase 1 study received two doses at 10, 30, or 100 µg of UB-612 at a 28-day interval (Day 0 and Day 28). Overall, approximately 3738 healthy participants or participants with stable and well-controlled comorbidity have received at least one dose of UB-612.
Based on the safety and immunogenicity results, the 100 µg dose level was selected for use in future clinical studies of UB-612.

7.4 End of Study Definition

A subject is considered to have completed the study if he or she has completed all phases of the study, including the last visit (12-months after booster vaccination). The end of the study is defined as the date of last visit of the last subject in the study.

8 STUDY POPULATION

8.1 Inclusion Criteria

To be eligible for the platform study, each subject must satisfy all of the following criteria. Additional sub-study-specific inclusion criteria are specified in the sub-study-specific appendix to the protocol.

1. Signed and dated informed consent/assent after reading the consent/assent form and having adequate opportunity to discuss the study with an investigator or designee.
2. Documented fully vaccinated with primary series of a comparator vaccine. Primary immunization is defined as 2 doses spaced approximately 3-16 weeks apart. The last dose of the previous vaccine must have been administered at least three (3) months prior to Day 1, taking into consideration the current local and national regulations, and according to details related to individual comparators provided in relevant sub-studies. Documentation, such as the National Health Service (NHS) COVID Pass, United States Centers for Disease Control vaccine card, or equivalent documentation (e.g., medical records, vaccine passport; in accordance with local approved vaccination record documentation) will be required for proof of vaccination, vaccine manufacturer and vaccination dates.
3. No clinically significant health problems that could affect the safety of the subject, as determined by the investigator by medical history, laboratory tests and physical examination.

   May have a stable pre-existing medical condition that did not require significant change in medication or hospitalization in 3 months before screening or which, in the judgement of the investigator is unlikely to require a significant change in therapy or hospitalization for worsening disease in the 3 months after Day 1.
4. Negative SARS-CoV-2 reverse transcriptase-polymerase chain reaction (RT-PCR) or antigen test within 24-48 hours prior to receipt of injections on Day 1.
5. Female subjects of non-childbearing potential may be enrolled. Non-childbearing potential defined as the following: must be either surgically sterile (hysterectomy, bilateral tubal ligation, bilateral salpingectomy, and/or bilateral oophorectomy at least 26 weeks before Screening) or postmenopausal, defined as spontaneous amenorrhea for at least 1 year. Female subjects not meeting this criterion will be considered of childbearing potential (WOCBP).
6. Males and WOCBP, 16 years or older, may be enrolled in the study if they are willing to practice abstinence from sexual intercourse or are willing to use acceptable methods of contraception as described below, from the time of signing the informed consent/assent during the screening period through study product injection on Day 1 and until completion of Day 29. An assent required to enroll minor subjects between 16 years and the age of adulthood will be defined by the laws in the territory of each site location. Acceptable methods of contraception should be consistent with local availability/regulations regarding the use of contraceptive methods for those participating in clinical trials and include the following:

An acceptable barrier method:

- Condom for male participants.
- Condom used by male partner plus spermicide for female participants.
- At least one of the following: combined (estrogen and progestogen containing) hormonal contraception associated with inhibition of ovulation (oral, intravaginal, or transdermal), progestogen-only hormonal contraception associated with inhibition of ovulation (oral, injectable, or implantable), intrauterine device, or intrauterine hormone-releasing system for at least 12 weeks before Screening.
- Bilateral tubal occlusion or vasectomized partner at least 26 weeks before Screening. A vasectomized partner is a highly effective method of contraception, provided that the male partner is the sole sexual partner of the study subject who is a WOCBP and that the vasectomized partner has received medical assessment of surgical success.
- Sexual abstinence. True abstinence, when in line with the preferred and usual lifestyle of the subject, is considered a highly effective method only if defined as refraining from heterosexual intercourse during the entire period of risk associated with the study drug treatment. Periodic abstinence (e.g., calendar, ovulation, symptothermal, post ovulation methods) and withdrawal are NOT acceptable methods of contraception.

7. For WOCBP, a serum or urine pregnancy test must be negative at Screening and on the day of study product injection.
8. Must be able to read, understand, and complete questionnaires and diary entries.
9. Plans to reside within study area for the duration of the study.
10. Able to comply with study procedures for the full duration of the study, in the opinion of the investigator.

8.2 Exclusion Criteria

Subjects with any of the following criteria will be excluded from the platform study. Additional sub-study-specific exclusion criteria are specified in the sub-study-specific appendix to the protocol.

1. Known history of COVID-19 or SARS-CoV-2 infection within six (6) months prior to vaccination (Day 1).
2. Receipt of a booster COVID-19 vaccination in addition to the primary vaccine series.
3. Presence of fever ≥100.4°F/38°C or other signs or symptoms of COVID-19 (e.g., chills, cough, shortness of breath or difficulty breathing, fatigue, muscle or body aches, headache, new loss of taste or smell, sore throat, congestion or runny nose, nausea or vomiting, or diarrhea) within 1 week before Day 1 study product injection. Screening and/or study product injection may be rescheduled at the discretion of the investigator.
4. Clinical manifestations of systemic diseases considered by the investigator to impact safety or immunogenicity.
5. Prior history of pericarditis or myocarditis of any etiology.

6. Prior history of thrombosis of major vessels, including cerebrovascular or splanchnic thrombosis or of thrombosis with thrombocytopenia syndrome
7. History of anaphylaxis (vaccine related or not).
8. Chronic kidney disease with dialysis.
9. Receipt of systemic corticosteroids (≥0.5 mg/kg per day of prednisone or equivalent) for ≥7 days is prohibited from 28 days before enrollment through conclusion of the study. Topical, inhaled, intra-nasal, intra-articular or intra-bursal administration of corticosteroids is permitted.
10. Receipt of any cytotoxic or immunosuppressive drug or biologics within six (6) months prior to vaccination (Day 1).
11. Receipt of any investigational drug within six (6) months prior to vaccination (Day 1).
12. Subject received or plans to receive a live attenuated vaccine or licensed adjuvanted (non-aluminum compound) vaccination within 28 days before or after planned administration of study vaccine (Day 1) or another type of vaccine (including influenza vaccine) within 14 days prior to or after vaccination (Day 1).
13. Human immunodeficiency virus (HIV) or hepatitis B surface antigen (HBsAg) positive; hepatitis C virus (HCV) antibody positive subjects may be tested for RNA and if negative may be enrolled.
14. Any Grade 2 or greater clinical or laboratory abnormalities at screening.

    Grade 1 abnormal clinical or laboratory screening test results which, according to the investigator, are non-clinically significant would not disqualify a potential subject. Clinical or laboratory screening tests may be repeated once to exclude transient abnormalities. Reference is made to Appendix 6: Laboratory Abnormality Grading Scale.

15. Immunocompromised state (weakened immune system) from solid organ transplant, immunosuppressive or immunodeficient state, autoimmune diseases, asplenia and, recurrent severe infections.
16. Have an active malignancy or history of metastatic or hematologic malignancy except non-melanoma skin cancers.
17. Pregnant or breastfeeding female, or female who intends to become pregnant during the study period.
18. Administration of immunoglobulins and/or any blood products within the 120 days preceding Day 1 or planned administration during the study period.
19. Bleeding disorder considered a contraindication to intramuscular injection or phlebotomy.
20. Bilateral tattoos or scars at the deltoid sites of intramuscular (IM) injection that would obscure examination of injection site reactions.
21. Behavioral, cognitive, or psychiatric disease that, in the opinion of the Principal Investigator or his or her representative physician, affects the subject’s ability to understand and cooperate with all study protocol requirements.

22. Any alcohol or drug abuse over the 12 months prior to enrollment in the study that has caused medical, professional, or family problems, indicated by clinical history.
23. Grade 2 or higher hypertension (systolic >160 mm Hg and/or diastolic >100 mm Hg).
24. Any other condition that, in the opinion of the Principal Investigator or his/her representative physician, could put the safety/rights of potential subjects at risk or prevent them from complying with the study protocol.

8.3 Lifestyle Considerations

Not applicable.

8.4 Screen Failures

Screen failures are defined as subjects who consent to participate in the clinical study but do not meet eligibility criteria and are not randomized. Minimal information to be collected on screen failures includes, but may not be limited to, the reason for screen failure, demographic data, and any SAEs that occurred during the screening period.

Subjects who do not meet the criteria for participation in this study (screen failure) may be rescreened up to two (2) times.

9 TREATMENT OF SUBJECTS

9.1 Description of the Study Vaccine

9.1.1 UB-612

UB-612 vaccine product contains a combined recombinant SARS-CoV-2 subunit fusion protein (S1-RBD-sFc) and rationally selected synthetic peptides (Th/CTL) as core immunogens, which are mixed with a proprietary CpG oligonucleotide (CpG1) excipient in solution and formulated with Adju-Phos® to form a wet gel suspension. The S1-RBD-sFc fusion protein is produced in CHO cells; the Th and CTL epitope peptides are selected from M, S2 and N regions; and the UBITh®1a is a T helper peptide adapted from MVF protein.

The S1-RBD-sFc fusion protein includes both linear and conformation epitopes, inducing high binding antibodies to the RBD, inhibits its binding to the hACE2 receptor and neutralizes replicating virus in CPE-based neutralization assays. The Th and CTL peptides are promiscuous and highly potent epitopes that are expected to bind to human MHC I and II for induction of memory recall and T cell activation and effector functions.

The vaccine components are combined in the presence of CpG as an excipient and added to aluminum phosphate (Adju-Phos®) as an adjuvant for production of the final UB-612 vaccine. UB-612 is supplied as a sterile Adju-Phos® suspension in multi-dose glass vials for a 0.5 mL IM injection.

9.1.2 Comparator Vaccine

Refer to the sub-study-specific appendix for details regarding the comparator vaccine.

9.1.3 Dosing Regimens

Subjects will be randomized 1:1 in either in a double-blind or open-label fashion, as defined in the relevant sub-study, to receive either UB-612 or a comparator vaccine on Day 1. Refer to the sub-study-specific appendix for the treatment arms in the applicable sub-study.

The study vaccine is to be administered as an IM injection into the deltoid muscle, preferably of the nondominant arm.

The study vaccine is to be prepared and administered at either the study center by an appropriately qualified and trained member of the study staff (e.g., physician, nurse, physician's assistant, nurse practitioner, pharmacist, or medical assistant) as allowed by national, local, state, and/or institutional guidance. or by qualified staff at a local vaccination location (e.g., doctor's office, clinic, pharmacy) of the subject's choice, as indicated in the relevant sub-study. At the study center, at a minimum, the date, time, and vaccine injection site will be recorded in the source documentation and the appropriate eCRF. Subjects will be required to provide documentation of vaccination if conducted outside of the center, at a minimum, the date, time (if applicable), the vaccine injection site, the name of vaccine, lot number, and expiration date.

All subjects administered study vaccine at the study center will be observed by a trained site staff member for at least 30 (±5) minutes after being vaccinated. Appropriate medication and other supportive measures for management of an acute hypersensitivity reaction will be available at the site in accordance with local guidelines for standard immunization practices. Subjects administered study vaccine at the local vaccination location are to be observed post-

vaccination as per standard institutional practice. Subjects must secure details of a comparator vaccine administration described above.

9.2 Preparation/Handling/Storage/Accountability of the Study Drug

9.2.1 Acquisition and Accountability

For vaccine administered at the study center, the investigator is responsible for study product accountability (UB-612 and vaccine comparator), reconciliation, and record maintenance (i.e., receipt, reconciliation, and final disposition records). All study vaccine will be accounted for using a study vaccine accountability form/record.

Study vaccine (UB-612 and vaccine comparator) will be stored in its original containers.

The study pharmacist or designee will keep a record of the study vaccine identity for each subject. Details will be provided in the Study Pharmacy Manual.

The investigator (or designee) must confirm appropriate temperature conditions have been maintained during transit for all study vaccine received and any discrepancies are reported and resolved before use of the study product. Any temperature excursions from the required storage conditions should be reported to Vaxxinity, or designee, upon discovery along with investigation and appropriate corrective actions taken. Guidance for reporting deviations in transport or storage is given in the Study Pharmacy Manual.

Information for the final disposition of unused investigational product is provided in the UB-612 Pharmacy Manual. All destruction must be adequately documented.

9.2.2 Formulation, Appearance, Packaging, and Labelling

9.2.2.1 UB-612

UB-612 is cloudy white suspension supplied in 10-mL type I glass vials (each vial contains 6.5 mL for 10 x 0.5 mL doses) for 100 µg per subject IM administration. UB-612 should be stored at 2 to 8°C (36 to 46°F) until the time of administration. Do NOT freeze the product. The vaccine should be kept in its original carton when not in use and protected from light. UB-612 use will be appropriately labeled in accordance with national laws and regulations, including required translations.

9.2.3 Product Storage and Stability

9.2.3.1 UB-612

UB-612, should be stored at 2 -8ºC (36 to 46ºF) in the original carton until the time of administration. The vaccine should be protected from exposure to light. DO NOT freeze the product. Resuspend the suspension by swirling before dosing. The multidose vial should be used within 6 hours after the first withdrawal of dosing material.

9.2.4 Preparation

9.2.4.1 UB-612

Doses of UB-612 will be prepared by a qualified and trained unblinded site staff member in the pharmacy. Before drawing into the syringe for administration, the vial should be swirled to mix the aluminum adsorbed suspension. Details for preparation and administration will be provided in the Study Pharmacy Manual.

9.3 Measures to Minimize Bias: Randomization and Blinding

In each sub-study, subjects will be randomized in 1:1 ratio to receive either UB-612 or a comparator vaccine. The randomization will be stratified by the following factors: age group (16-64 vs. ≥65 years old), gender at birth (male vs female), N-protein seropositivity at Screening (positive vs. negative), and the time since the last primary immunization dose (≥3-<5 months, vs. ≥5 months).

For double-blind sub-studies, sponsor study team, subjects, the investigator, and study staff, including those who administer study injections and who evaluate subject safety will all be blinded to the subject's treatment assignment for the duration of the study. All laboratory testing personnel performing assays will also remain blinded to the study treatment received throughout the study. As the study vaccines have different physical appearance, the syringes will be masked to maintain the blind.

While the majority of the site's staff will remain blinded to the treatment assignment, the site staff responsible for receiving, storing, dispensing, and preparing the study treatments will be unblinded.

In case of a medical emergency where the identity of the study treatment is required, the investigator will be responsible for determining if unblinding is warranted. If the investigator decides that unblinding is required, he/she will make every effort to contact the Medical Monitor or other sponsor personnel prior to unblinding unless this would delay further management of the subject. The sponsor must be notified within 24 hours after breaking the blind. The date and reason for unblinding must be recorded in the source documentation and CRF. Procedures for this individual unblinding will be provided in an appropriate study manual.

In the open-label sub-studies, the treatment assignments are known to the investigator, study staff, and subjects, while the sponsor study team who are responsible for the design, data cleaning and analysis will remain blinded to minimize the potential bias.

9.4 Study Vaccine Compliance

Doses of study vaccine will be prepared and administered by site staff at a study center visits. Study vaccine compliance is dependent on proper vaccine preparation and administration by study center personnel.

Study vaccine administration date, time, and vaccine injection site will be recorded in the source documentation and CRFs.

9.5 Concomitant Medications

The following prior and concomitant medications and vaccinations will be recorded in the CRF:

- Both prior administrations of COVID-19 vaccine, as specified in the comparator arm-specific sub-study. The last dose of the previous vaccine must have been administered at least three (3) months from Day 1, taking into consideration the local and national regulations. The NHS COVID Pass, Centers for Disease Control (CDC) vaccine card, or equivalent documentation (e.g., medical records) will be required for proof of vaccination, vaccine manufacturer and vaccination dates. The exact date of prior vaccination must be recorded.
- All non-COVID-19 vaccinations received from 28 days before study Day 1 until the 6-month follow-up visit.
- Restricted medications (if any) listed in Section 9.5.1.
- All current medications at baseline.
- All new medications prescribed after randomization.

The start and stop dates, name of medication, dose, unit, route, and frequency of administration will be documented in the CRF.

9.5.1 Medications Restricted During the Study

Receipt of vaccines and medications listed below may exclude a subject from the Evaluable Population from the time of receipt onwards and may lead to screen failure if it triggers an exclusion criterion before study product injection; however, any subject vaccinated on Day 1 (Visit 1) will not be withdrawn from the study and will continue with follow-up study visits. Medications will not be withheld if required for a subject's medical care.

The following medications are restricted:

- Systemic corticosteroids (≥0.5 mg/kg per day of prednisone or equivalent) for ≥7 days is prohibited from 28 days before enrollment through conclusion of the study. Topical, inhaled, intra-nasal, intra-articular or intra-bursal administration of corticosteroids is permitted.
- Receipt of any cytotoxic or immunosuppressive drug or biologics within six (6) months before Day 1 through conclusion of the study.
- Licensed live attenuated vaccines or licensed adjuvanted (non-aluminum compound) vaccines within 28 days before or after administration of study vaccine on Day 1 (Visit 1), and any other vaccines (including influenza) are not permitted within 14 days before or after planned administration of vaccination (Day 1).
- Receipt of other COVID-related treatments including ivermectin, monoclonal antibodies, etc, within six (6) months prior to vaccination (Day 1)
- Blood/plasma products or immunoglobulins within 120 days before enrollment through conclusion of the study.
- Any other (non-study-specified) coronavirus vaccine at any time prior to or during study participation.

- Prophylactic antipyretics, H1 antihistamines, and pain medication to prevent symptoms associated with study product injection are not permitted 24 hours prior to receipt and 24 hours after receipt of injection on Day 1. However, if a subject is taking a medication for another condition, even if it may have antipyretic, H1 antihistamine, or pain-relieving properties, it should not be withheld prior to study product injection.

9.5.2 Medications Permitted During the Study

The use of antipyretics and other pain medication to treat symptoms associated with study product injection or ongoing medical conditions is permitted within the guidelines stated above.

Except for medications listed in Section 9.5.1 and, as applicable, the comparator arm-specific appendices, other treatments or therapies for preexisting stable conditions are permitted.

Topical, inhaled, intra-nasal, intra-articular or intra-bursal administration of corticosteroids is permitted.

The use of antivirals (i.e., Paxlovid, Lagevrio, and Veklury) to treat mild to moderate COVID-19 in subjects at risk of developing severe disease (“rescue medicine”) is permitted throughout the study. The receipt of immunoglobulins and monoclonal antibodies to treat COVID-19 remains restricted.

10 STUDY PRODUCT DISCONTINUATION AND PARTICIPANT DISCONTINUATION/WITHDRAWAL

10.1 Discontinuation of Study Product

This is a single-dose study; therefore, there are no criteria for discontinuation of study product.

10.2 Participant Discontinuation/Withdrawal from the Study

This study will attempt to follow all subjects for safety and immunogenicity for 12 months post-injection unless they withdraw consent/assent to do so. Subjects can withdraw from the study at any time for any reason, without prejudice, and without having to justify their decision. When a subject discontinues or withdraws from the study, the primary reason(s) for discontinuation or withdrawal must be recorded in the CRF and source document. All efforts will be made to complete End of Study (Visit 7) assessments. Lack of completion of all or any of the withdrawal/early termination procedures will not be viewed as a protocol deviation so long as subject safety is preserved.

If a subject does not return for a scheduled visit, every effort should be made to contact him or her. All attempts to contact the subject and information received during each attempt must be documented in the subject's source document. In any circumstance, every effort should be made to document subject outcome, if possible.

If a subject withdraws from the study, he/she may request destruction of any remaining samples taken and not tested, and the investigator must document any such requests in the site study records and notify the sponsor accordingly.

If the subject withdraws from the study and withdraws consent/assent for disclosure of future information (see Section 10.2.2), no further evaluations should be performed, and no additional data should be collected. The sponsor may retain and continue to use any data collected before such withdrawal of consent/assent.

Any subject with a positive or presumptive SARS-CoV-2 diagnosis with or without symptoms will be followed as medically indicated and does not have to be withdrawn from the study.

10.2.1 Withdrawal of Consent/Assent for Disclosure of Future Information

Subjects who are randomized and vaccinated will be encouraged to complete all protocol-specified follow-up procedures. The only exception to this is when a subject (or person(s) previously authorized by the subject) specifically withdraws consent/assent. Whenever possible, subjects should notify the investigator of the decision to withdraw consent/assent from future follow-up in writing. The withdrawal of consent/assent should be documented in detail in the source document and CRF (i.e., whether the subject withdraws consent/assent from receipt of study vaccine or from performing study procedures and/or post-treatment study follow-up).

10.2.2 Lost to Follow-up

A subject will be considered lost to follow-up if he/she repeatedly fails to return for scheduled visits and is unable to be contacted by the study site. The following actions must be taken if a subject fails to attend a required study visit:

- The site must attempt to contact the subject and reschedule the missed visit as soon as possible and counsel the subject on the importance of maintaining the assigned visit

schedule and ascertain whether or not the subject wishes to and/or should continue in the study;

- Before a subject is deemed lost to follow-up, the investigator or designee will try to contact the subject as follows:
    - Two separate telephone calls or message to subject or subject's alternative contact person.
    - If unsuccessful in gaining a response, a certified letter will be sent to the subject's last known mailing address. These three (3) contact attempts will each be made on each of three (3) separate days. These contact attempts and their outcomes will be documented in the subject's source documents.
- Should the subject continue to be unreachable, he/she will be considered discontinued from the study.

10.2.3 Contraception Requirements

Women of non-childbearing potential (WONCBP) may be enrolled in the study. WONCBP must be either surgically sterile (hysterectomy, bilateral tubal ligation, salpingectomy, and/or bilateral oophorectomy at least 26 weeks before Screening) or postmenopausal, defined as spontaneous amenorrhea for at least one (1) year. Female subjects not meeting this criterion will be considered of childbearing potential (i.e., WOCBP).

Males and females, 16 years or older, must be willing to practice abstinence from sexual intercourse or are willing to use acceptable methods of contraception as described below, from the time of signing the informed consent/assent during the screening period through study product injection on Day 1 and until completion of Day 29. (An assent required to enroll minor subjects between 16 years and the age of adulthood will be defined by the laws in the territory of each site location.) Acceptable methods of contraception should be consistent with local availability/regulations regarding the use of contraceptive methods for those participating in clinical trials and include the following:

- An acceptable barrier method (male participants – condom; female participants - condom used by male partner plus spermicide).
- At least one of the following: combined (estrogen and progestogen containing) hormonal contraception associated with inhibition of ovulation (oral, intravaginal, or transdermal), progestogen-only hormonal contraception associated with inhibition of ovulation (oral, injectable, or implantable), intrauterine device, or intrauterine hormone-releasing system for at least 12weeks before Screening.
- Bilateral tubal occlusion or vasectomized partner at least 26 weeks before Screening. A vasectomized partner is a highly effective method of contraception, provided that the male partner is the sole sexual partner of the study subject who is a WOCBP and that the vasectomized partner has received medical assessment of surgical success.
- Sexual abstinence. True abstinence, when in line with the preferred and usual lifestyle of the subject, is considered a highly effective method only if defined as refraining from heterosexual intercourse during the entire period of risk associated with the study drug treatment. Periodic abstinence (e.g., calendar, ovulation, symptothermal, postovulation methods) and withdrawal are NOT acceptable methods of contraception.

10.2.3.1 Female Subjects

A serum or urine human chorionic gonadotropin (HCG) pregnancy test will be performed on WOCBP during Screening and a urine HCG pregnancy test will be performed on Day 1 (prior to study product injection) and on Days 15 and 29.

Female subjects will be asked to refrain from donating reproductive tissue (eggs) from the time of informed consent/assent until 28 days after being vaccinated.

10.2.3.2 Male Subjects

A male subject is considered of sexual reproductive potential if he is anatomically and physiologically capable of causing a pregnancy in a female partner and will be or could possibly be sexually active with a female partner (who is or may become pregnant) while undergoing study treatment with the possibility of posing harm to a fetus.

Male subjects must agree to use an acceptable method of birth control, as defined in Section 10.2.3, during sexual intercourse between Screening and at least 28 days after study product administration. Male subjects must ensure non-pregnant female partners of childbearing potential comply with the contraception requirements in the inclusion criteria #5. A surgically sterile male (vasectomy >26 weeks prior to screening and medical assessment of surgical success) must also use a second highly effective method of contraception.

Male subjects will be asked to refrain from donating reproductive tissue (sperm) from the time of informed consent/assent until 28 days after being vaccinated.

11 STUDY ASSESSMENTS AND PROCEDURES

11.1 Timing and Events

The schedule of assessments for the study is presented in Table 1. The investigator (or an appropriate delegate at the investigator site) must obtain a signed and dated informed consent/assent form (ICF) before performing any study-specific procedures. Adherence to the study design requirements specified in the schedule of assessments is essential.

All subjects must provide written informed consent/assent before the performance of any study-related procedures.

If a protocol-required test or blood collection cannot be performed, the investigator will document the reason for the missed test. Any corrective and preventive actions taken to ensure that required processes are adhered to will also be documented as soon as possible. The site staff must be informed of these incidents in a timely manner.

Instructions for sample collection, processing, storage, and shipment, including contact information, will be provided in the Laboratory Manual prior to initiation of the study. The investigator should ensure that designated staff follow established site standard operating procedures (SOPs) and the study Laboratory Manual and are trained to collect, process, store, and ship samples properly. PBMC samples, particularly, from designated subjects should be timely and carefully processed, and staff trained accordingly.

Additional blood samples may be taken for safety assessments (i.e., chemistry, hematology) for the diagnostic evaluation of treatment-emergent adverse events, provided the total volume taken during the study does not exceed the amount specified in sub-study-specific informed consent/assent form.

Visits will not require overnight admission in the unit.

11.1.1 Screening

Screening assessments can be conducted at multiple visits (if necessary) any time between Day -28 and Day 1 up to and including Day 1 prior to receipt of study vaccine administration. The investigator will maintain a screening log to record details of all screened subjects and to record confirmation of eligibility or reasons for screening failure, as applicable.

The subject will receive verbal information concerning the aims and methods of the study, its constraints and risks, and the study duration. The written informed consent/assent must be signed and dated by the subject and the investigator prior to any assessments/procedures. No screening procedures or assessments can be performed until the subject is fully informed of the study and signs the informed consent/assent form. Subjects who are diagnosed with a medical condition during the screening process will be notified, referred for medical care and excluded from the study.

11.1.2 Booster Vaccination

Subjects who meet all study screening criteria will be randomized and receive a single injection of study vaccine according to their treatment assignment on Day 1.

11.1.3 Post Vaccination Follow-up

The follow-up period starts after study vaccine administration on Day 1 (Visit 1), and includes the following post-treatment visits:

- Telephone follow-up on Days 8 (±1 day) and 57 (±3 days) (Visits 2 and 5).
- Study center visits on Day 15 (±1 day) and Day 29 (±3 days) (Visits 3 and 4)
- Study center visits at Months 6 and 12 (±14 days) (Visits 6 and 7). The Month 12 visit is the End-of-Study visit.

11.1.4 Unscheduled Visit

If a subject reports a Grade 3 local AE, systemic event, or fever, they must be instructed to contact the site staff to discuss the details and determine whether a site visit is clinically indicated.

If a subject suspects a Grade 4 local AE, systemic event, or fever, they must be instructed to contact the site staff to discuss the details and determine if a site visit should occur to confirm whether the event meets the criteria for Grade 4.

A clinic visit must be scheduled as soon as possible to assess the subject unless any of the following is true:

- The subject is unable to attend the unscheduled visit.
- The local AE or systemic event is no longer present at the time of the telephone contact.
- The subject recorded an incorrect value in the reactogenicity subject diary (confirmation of a reactogenicity subject diary data entry error).
- The investigator or designee determined it was not needed.

Each telephone contact will be recorded in the source documentation and CRF.

If the subject is unable to attend the unscheduled visit, or the investigator or designee determined it was not needed, any ongoing solicited local AEs or solicited systemic AEs must be assessed at the next study visit.

During the unscheduled visit, the investigator, or a medically qualified member of the site staff (e.g., study physician, nurse) will assess the AE and perform the following:

- Measure body temperature (°F/°C)
- Measure minimum and maximum diameters of redness (if present)
- Measure minimum and maximum diameters of swelling (if present)
- Assess injection site itching and pain/tenderness (if present) and assign severity grade
- Assess solicited systemic AEs (if present) and assign severity grade
- Assess for other findings associated with the AE and record in the CRF, if appropriate

In addition, if deemed necessary by the Investigator or designee, a subject may be asked to return to the clinic for an unscheduled visit for safety reasons.

The investigator or designee will complete the unscheduled visit assessment page of the CRF.

11.2 Study Assessments

11.2.1 Demographics

Subject demographic data, including gender, date of birth, age, race, and ethnicity will be obtained during the screening visit. When allowed by local and national regulations, the full date of birth will be collected to critically evaluate the immune response and safety profile by age and documented as appropriate per national, local, and institutional requirements.

11.2.2 Medical History and Baseline Characteristics

Medical history, also collected during the screening visit, will include all relevant prior medical history and current medical conditions. In particular, the presence of co-morbidities associated with COVID-19 will be documented, including the following conditions (laboratory or clinical toxicities of Grade 2 or greater are exclusionary):

- Chronic kidney disease. Subjects with chronic kidney disease with dialysis are excluded.
- Chronic obstructive pulmonary disease.
- Down Syndrome.
- Heart conditions, such as heart failure, coronary artery disease, or cardiomyopathies.
- Obesity (body mass index of ≥30 kg/m^2).
- Sickle cell disease.
- Smoking.
- Type 1 or Type 2 diabetes mellitus.
- Asthma (moderate to severe).
- Cerebrovascular disease.
- Chronic liver disease.

11.2.3 SARS-CoV-2 Testing

A SARS-CoV-2 RT-PCR and/or antigen test is to be administered at the time points indicated in Table 1. Saliva, nasopharyngeal, or nasal mid-turbinate swab may be collected in accordance with the package instructions for the test used. The SARS-CoV-2 test performed as part of Screening within 48 hours prior to study vaccine injection can be combined with Day 1. Antigen test results can be optionally confirmed by RT-PCR testing within 24 hours.

11.2.4 Physical and Neurological Examination

A general physical examination will be performed during Screening and will include the overall appearance, head and face, ear, nose, throat, mouth, skin, lymph nodes, respiratory, cardiovascular, abdomen, musculoskeletal and nervous, and neurological systems. A targeted physical examination should be performed if indicated by medical history or review of systems, at discretion of investigator.

11.2.5 Height and Weight

The subject's height and weight will be recorded during Screening.

11.2.6 Vital Signs

Vital sign measurements will be taken at the time points indicated in Table 1 and will include systolic and diastolic blood pressure, heart rate, respiratory rate, and body temperature (oral, axillary, or temporal). Assessments may be conducted while the subject is either seated or supine.

11.2.7 Laboratory Assessments

Blood and urine samples will be collected at the time points indicated in Table 1 to complete the protocol-required laboratory assessments are presented in Table 3. Note that the hematology/chemistry tests performed during Screening within 48 hours prior to study vaccine injection can be combined with Day 1.

Samples for serology and glycated hemoglobin will only be collected at the screening visit. These samples will be processed according to the instructions from the local laboratories. Samples will be reported by the local laboratories against their normal reference ranges. Serology can be performed within 48 hours prior to study vaccine injection on Day 1, including authorized rapid antibody test systems.

Unscheduled clinical laboratory measurements may be obtained at any time during the study to assess any intercurrent safety issues.

Table 3: Clinical Laboratory Testing

**Hematology**	Hemoglobin, hematocrit, red blood cells, reticulocytes, white blood cell with differential, platelets
**Coagulation**	Prothrombin time, partial thromboplastin time, international normalized ratio
**Chemistry**	Alanine aminotransferase, aspartate aminotransferase, creatinine, total and direct bilirubin, hs-CRP[†]
**Hemoglobin A1c**[*]	Only performed at Screening
**Serology**[*]	HIV (human immunodeficiency virus), HBsAg (hepatitis B surface antigen), HBcAb (hepatitis core antibody), HCV (hepatitis C)

[*]Only collected at Screening visit.
[†] hs-CRP collected at Day 1, Day 15, and Day 29

11.2.8 Pregnancy Test

Pregnancy tests will be performed for WOCBP and must have a sensitivity of at least 25 mIU/mL. Serum or urine HCG pregnancy tests will be performed at the screening visit. Urine or serum pregnancy tests are to be performed at all other time points as designated in Table 1. Pre-injection pregnancy tests (Screening and Day 1) must be negative for the subject to be eligible for the study product administration on Day 1. If the Screening and Day 1 visits are combined, serum pregnancy test should be used for screening.

Pregnancy testing can be performed at a more frequent occurrence, upon investigator discretion and/or in accordance with local requirements.

In the case of a confirmed pregnancy during the study, the subject will be encouraged to remain in the study and be followed up for safety. A pregnancy registry will be established to document all pregnancy outcomes.

11.2.9 Electrocardiogram

A 12-lead electrocardiogram (ECG) is to be performed with the subject either seated or supine at the time points designated in Table 1.

11.2.10 Collection of Solicited Local and Systemic Adverse Events

Subjects will be required to complete a subject diary for the collection of local and systemic adverse events starting on Day 1 through Day 7. Subjects will record solicited local and systemic AEs as well as antipyretic medication usage following administration of the study product daily for the seven (7) days following receipt of study product injection (up to and including Day 8). The subject diary allows recording of these assessments only within a fixed time window, thus ensuring an accurate representation of the subject's experience at that time. The investigator (or designee) will review reactogenicity data at frequent intervals as part of the ongoing safety review. The investigator or designee must obtain stop dates from the subject for any ongoing local AEs, systemic AEs, or use of antipyretic medication on the last day that the diary was completed. Data recorded on solicited local and systemic AEs reported in the subject diary will be reviewed by study staff and recorded in source documents and transferred to the CRF.

11.2.10.1 Solicited Local (Injection Site) AEs

During the seven (7) days following receipt of study product injection (up to and including Day 8), subjects will be asked to assess solicited local AEs (i.e., redness, swelling, itching, and pain/tenderness at the injection site) and record the symptoms in the reactogenicity subject diary. If a local AE persists beyond the seven-day reporting period, the subject will be requested to report information on the AE until symptoms have resolved. As stated above, site staff will review this information with the subject and record it in the source documentation and in the CRF.

Subjects will assess solicited local AEs as absent, mild, moderate, or severe based on the grading scale in Table 4. Redness and swelling will be measured and recorded using a metric ruler.

If a subject reports a Grade 3 local AE, they must be instructed to contact site staff to discuss the details and determine whether a site visit is clinically indicated.

If a subject experiences a confirmed Grade 4 local AE, the investigator must immediately notify the sponsor if it is determined to be related to the administration of the study vaccine.

Table 4: Local (Injection Site) Adverse Event Grading Scale

Local Adverse Event	Mild (Grade 1)	Moderate (Grade 2)	Severe (Grade 3)	Potentially Life Threatening (Grade 4)
Pain	Does not interfere with activity	Repeated use of nonnarcotic pain reliever >24 hours or interferes with activity	Any use of narcotic pain reliever or prevents daily activity	Emergency room visit or hospitalization
Tenderness	Mild discomfort to touch	Discomfort with movement	Significant discomfort at rest	ER visit or hospitalization
Erythema/Redness*	>2.0 to 5.0 cm (5 to 10 measuring device units	>5.0 to 10.0 cm (11 to 20 measuring device units)	>10 cm (≥21 measuring device units)	Necrosis or exfoliative dermatitis
Induration/Swelling	>2.0 to 5.0 cm (5 to 10 measuring device units)	>5.0 to 10.0 cm (11 to 20 measuring device units)	>10 cm (≥21 measuring device units)	Necrosis or exfoliative dermatitis
Pruritus (without skin lesions)	Itching causing no or minimal interference with usual social & functional activities	Itching causing greater than minimal interference with usual social & functional activities	Itching causing inability to perform usual social & functional activities	N/A

* In addition to grading the measured local reaction at the greatest single diameter, the measurement should be recorded as a continuous variable.

11.2.10.2 Solicited Systemic Adverse Events

During seven (7) days following receipt of study product injection (up to and including Day 8), subjects will be asked to record fever, fatigue, headache, chills, vomiting, nausea, diarrhea, rash, new or worsened muscle pain, and new or worsened joint pain in the reactogenicity subject diary. If a systemic AE persists beyond the seven-day reporting period, the subject will be requested to report information on the AE until symptoms have resolved. As stated above, site staff will review this information with the subject and record it in the source documentation and in the CRF.

The systemic symptoms will be assessed by the subject as absent, mild, moderate, or severe according to the grading scale in Table 5.

If a subject reports a Grade 3 systemic event, they must be instructed to contact the site staff to discuss the details and determine whether a site visit is clinically indicated.

If a subject experiences a confirmed Grade 4 systemic AE, the investigator must immediately notify the sponsor if it is determined to be related to the administration of the study vaccine.

Table 5: Systemic Adverse Event Grading Scale

Systemic Adverse Event	Mild (Grade 1)	Moderate (Grade 2)	Severe (Grade 3)	Potentially Life Threatening (Grade 4)
Nausea/vomiting	No interference with activity or 1 – 2 episodes/24 hours	Some interference with activity or > 2 episodes/24 hours	Prevents daily activity, requires outpatient IV hydration	ER visit or hospitalization for hypotensive shock
Diarrhea	2 – 3 loose stools or < 400 gms/24 hours	4 – 5 stools or 400 – 800 gms/24 hours	6 or more watery stools or > 800gms/24 hours or requires outpatient IV hydration	ER visit or hospitalization
Headache	No interference with activity	Repeated use of nonnarcotic pain reliever > 24 hours or some interference with activity	Significant; any use of narcotic pain reliever or prevents daily activity	ER visit or hospitalization
Fatigue	No interference with activity	Some interference with activity	Significant; prevents daily activity	ER visit or hospitalization
Myalgia	No interference with activity	Some interference with activity	Significant; prevents daily activity	ER visit or hospitalization
Chills	No interference with activity	Some interference with activity	Significant; prevents daily activity	Not applicable
Joint Pain	No interference with activity	Some interference with activity	Significant; prevents daily activity	Disabling joint pain causing inability to perform basic self-care functions
Rash	No interference with activity (Localized rash)	Some interference with activity (Scattered, but not generalized rash)	Significant; prevents daily activity (Generalized rash, associated with pain)	ER visit or hospitalization (life-threatening or disabling condition)

Fever

A digital thermometer will be given to subjects with instructions on how to measure oral temperature at home. Oral temperature will be collected in the reactogenicity subject diary in the beginning the evening after the injection and at approximately the same time daily during the seven (7) days following receipt of study product injection (up to and including Day 8). It will also be collected at any time during the diary data collection periods when subjective fever is reported. Fever is defined as an oral temperature of ≥38.0°C (100.4°F). The highest temperature for each day will be recorded in the reactogenicity subject diary.

Temperature will be categorized in the CRF during analysis according to the scale shown in Table 6.

If a subject reports a fever of ≥39.0°C (102.1°F), they must be instructed to contact the site staff to discuss the details and determine whether a site visit is clinically indicated.

Only an investigator or medically qualified person can confirm a subject's fever is >40.0°C (>104.0°F). If a subject experiences a confirmed fever >40.0°C (>104.0°F), the investigator must immediately notify the sponsor if it is determined to be related to the administration of the study vaccine.

Table 6: Fever Grading Scale (Oral Temperature)

**Mild (Grade 1)**	≥38.0 to 38.4°C (100.4 to 101.1°F)
**Moderate (Grade 2)**	>38.4 to 38.9°C (101.2 to 102.0°F)
**High (Grade 3)**	>38.9 to 40.0°C (102.1 to 104.0°F)
**Extremely high (Grade 4)**	>40.0°C (>104.0°F)

Antipyretic Medication

The use of antipyretic medication to treat symptoms associated with study vaccine administration will be recorded in the reactogenicity subject diary daily during the seven (7) days following receipt of study product injection (up to and including Day 8).

11.2.11 COVID-19 Surveillance

If a subject experiences any of the COVID-19 symptoms listed in the following subsections, irrespective of perceived etiology or clinical significance, he or she is instructed to contact the site immediately and, if deemed necessary by the study investigator, participate in an in-person or telephone visit as soon as possible (optimally within 3 days of symptom onset and at the latest, 4 days after symptom resolution). Potential COVID-19 symptoms that overlap with solicited systemic AEs (i.e., fever, chills, new or increased muscle pain, diarrhea, vomiting) should not trigger a suspected COVID-19 illness visit unless, in the investigator's opinion, the event is more indicative of a possible COVID-19 illness than vaccine reactogenicity.

For all subjects with confirmed SARS-CoV-2 infection, a biological sample is to be collected and stored for future viral sequencing.

11.2.11.1 Criteria for Case Definition of COVID-19

The following definitions will be used to classify SARS-CoV-2 infection and COVID-19 cases.

11.2.11.1.1 CDC Interim Case Definition Criteria of COVID-19

The CDC Interim Case Definition, approved 5 August 2020, is presented in Table 7.

Table 7: CDC Interim Case Definition Criteria of COVID-19

Case	Definition
**Suspected based on clinical symptoms**	• At least 2 of the following symptoms: fever (measured or subjective), chills, rigors, myalgia, headache, sore throat, nausea or vomiting, diarrhea, fatigue, congestion or runny nose, ***OR***<br>• At least 1 of the following symptoms: cough, shortness of breath, difficulty breathing, new olfactory disorder, new taste disorder, ***OR***<br>• Severe respiratory illness with at least 1 of the following:<br>– clinical or radiographic evidence of pneumonia ***OR***<br>– acute respiratory distress syndrome (ARDS)
**Confirmed by laboratory testing**	To meet the case definition, a virological diagnosis must be made using a validated test for RNA (detection of severe acute SARS-CoV-2 RNA in a clinical or autopsy specimen using a molecular amplification test).<br>If the point-of-care test is negative, the test will be repeated, and a sample will be sent to a central laboratory for confirmation.

Subjects with suspected or confirmed COVID-19, as per the definitions in Table 7 are to be followed on a daily basis for 14 days and weekly thereafter until resolution of symptoms or the end of the study to facilitate the identification and documentation of COVID-19-related symptoms (see Section 11.2.11.2).

11.2.11.2 SARS-CoV-2 Infection With and Without Symptoms

The following definitions are used for exploratory endpoints:

Symptomatic SARS-CoV-2 Infection

- At least 2 of the following symptoms: fever (measured or subjective), chills, rigors, myalgia, headache, sore throat, nausea or vomiting, diarrhea, fatigue, congestion or runny nose.

OR

- Any of the following symptoms: cough, shortness breath, difficulty breathing, new olfactory disorder, new taste disorder.

Indeterminate SARS-CoV-2 Infection

- Suggestion of illness or prodromal illness that does not meet the CDC clinical citeria (see Section 11.2.11.1.1).
- May have 1 symptom among the following: fever (measured or objective), chills, rigors, myalgia, headache, sore throat, nausea or vomiting, diarrhea, fatigue, congestion or runny nose; absence of cough, shortness of breath, difficulty breathing, loss of taste or smell.

Asymptomatic SARS-CoV-2 Infection

- No respiratory symptoms (absence of coryza, sore throat, cough, or other respiratory signs or symptoms, change in sense of taste or smell).
- No systemic symptoms or signs (absence of fever, chills, gastrointestinal symptoms, fatigue, myalgia, arthralgia).

Pre-symptomatic SARS-CoV-2 Infection

- Meets definition of asymptomatic infection but, at a later time, develops symptoms consistent with symptomatic or indeterminate SARS-CoV-2 infection. The case definition of SARS-CoV-2 infection will follow the criteria described below, irrespective of clinical presentation.

Table 8: SARS-CoV-2 Infection Definitions for Exploratory Endpoints

Case	Definition
**Confirmed case of SARS-CoV-2 infection, irrespective of clinical presentation**	Virologic diagnosis of SARS-CoV-2 by:<br>• Positive test on saliva or, nasopharyngeal or nasal mid-turbinate swab measured by real-time reverse-transcription polymerase chain reaction (RT-PCR), ***AND***<br>• Confirmation by a repeat positive test on nasopharyngeal swab, or a lower respiratory tract sample by RT-PCR, or<br>• Confirmation by seroconversion in paired acute (closest to positive RT-PCR test) and convalescent serum samples (≥ 2 weeks after acute sample) or between the screening test and a convalescent serum sample using a test specific for natural SARS-CoV-2, e.g., N-protein IgG ELISA (does not recognize vaccine immunity)
**Probable case of SARS-CoV-2 infection, irrespective of clinical presentation**	Virologic diagnosis of SARS-CoV-2 by:<br>• Saliva, nasopharyngeal swab or nasal mid-turbinate positive by real-time RT-PCR on a single sample
**Serologic evidence only (applies to Exploratory Endpoint only)**	4-fold increase in N protein antibody titer by IgG ELISA between serum samples taken ≥14 days after the 2nd dose of investigational product and any timepoint up to end of study
**Clinical definition for SARS-CoV-2 infection for Primary and Secondary Endpoints**	Clinical classification applies only to confirmed and probable cases as described above.

11.2.11.3 Severe COVID-19 and WHO Clinical Progression Scale for Classification of Severity

11.2.11.3.1 Severe COVID-19

These clinical criteria for the definition of severe COVID disease are used for the purpose of efficacy endpoint determination and for monitoring for vaccine enhanced disease:

In subjects with RT-PCR positive on a single sample AND Severe COVID-19 (≥ 1 of the following):

a. Tachypnea: ≥ 30 breaths per minute at rest
b. Resting heart rate ≥125 beats per minute
c. Oxygen saturation (SpO_2): ≤93% on room air or partial pressure of oxygen/fraction of inspired oxygen <300 mmHg
d. High flow oxygen (O_2) therapy or non-invasive ventilation (NIV)/non-invasive positive pressure ventilation (e.g., continuous positive airway pressure or bilevel positive airway pressure)
e. Mechanical ventilation or extracorporeal membrane oxygenation (ECMO)
f. One or more major organ system dysfunction or failure to be defined by diagnostic testing/clinical syndrome/interventions, including any of the following:
    - Acute respiratory failure, including acute respiratory distress syndrome
    - Acute renal failure
    - Acute hepatic failure
    - Acute right or left heart failure
    - Septic or cardiogenic shock (with shock defined as systolic blood pressure <90 mmHg OR diastolic blood pressure <60 mmHg)
    - Acute stroke (ischemic or hemorrhagic)
    - Acute thrombotic event: acute myocardial infarction, deep vein thrombosis, pulmonary embolism
    - Requirement for: vasopressors, systemic corticosteroids, or hemodialysis
g. Admission to an intensive care unit
h. Death

11.2.11.3.2 WHO Clinical Progression Scale for Classification of Severity

All SARS-CoV-2 infections in the study will be classified using the WHO clinical progression scale (see Appendix 8) to allow comparison of outcomes across studies. This classification will be used for a pre-specified exploratory analysis (i.e., severity of COVID-19).

11.2.11.4 Procedures for Confirmed or Suspected SARS-CoV-2 Infection and COVID-19

11.2.11.4.1 Suspected COVID-19 Illness Visit

This visit may be conducted as an in-person or telemedicine or telephone visit, optimally within 3 days after onset of illness. As a subject's COVID-19 illness may evolve over time, several contacts may be required to obtain the following information:

- Record AEs, as appropriate.

  Potential COVID-19 illnesses that are consistent with the clinical endpoint definition will be recorded on a COVID-19 CRF and will not be listed as AEs.

- Record details of concomitant medication including any prohibited medications specified in Section 9.5.1 received by the subject if required for his or her clinical care.
- Obtain a nasopharyngeal swab (collected by site staff during an in-person visit or self collected by the participant during a telemedicine or telephone visit) for COVID-19 testing.

  If visit is conducted by telemedicine or telephone, the subject is to be instructed to provide a nasal mid-turbinate swab or salivia sample at the local laboratory. The result from this swab will be provided to the site once it is available. As results from this test will not be available in real time, the test cannot be relied upon to direct clinical care. Therefore, the subject should also be encouraged to seek care, if appropriate, from his or her usual provider.

  Virus replication should be tested with an antigen test or RT-PCR, with the latter being the preferred testing method.

  An antigen test result can be optionally confirmed by a nasopharyngeal swab for RT-PCR within 24 hours if possible.

  For all subjects with confirmed SARS-CoV-2 infection, a biological sample is to be stored for future viral sequencing.

- Collect blood samples for immunogenicity testing (if visit is in-clinic):
  - SARS-CoV-2 N protein antibody (Elecsys cobas or ELISA)
  - IgG S1-RBD ELISA and neutralization tests
- If the the subject has been hospitalized, collect COVID-19-related standard-of-care clinical and laboratory information. This includes, but is not limited to:
  - Symptoms and signs, including:
    - Clinical signs at rest indicative of severe systemic illness (respiratory rate ≥30 breaths per minute, heart rate ≥125 beats per minute, SpO_2 ≤93% on room air at sea level, or partial pressure of arterial oxygen/fraction of inspired oxygen <300 mm Hg)
    - Evidence of shock (systolic blood pressure <90 mm Hg, diastolic blood pressure <60 mm Hg, or requiring vasopressors)
    - Significant acute renal, hepatic, or neurologic dysfunction

        - Respiratory failure (defined as needing high-flow oxygen, noninvasive ventilation, mechanical ventilation, or ECMO)
    - Clinical diagnosis
    - Results from any local laboratory testing including:
        - COVID-19 test results
        - Full blood count, blood chemistry (specifically creatinine, urea, liver function tests, and hs-CRP)
    - Outcome of imaging results (e.g., computed tomography or magnetic resonance imaging scan) to document neurologic dysfunction
    - Document details of any healthcare contact and duration of hospitalization
    - If ifection results in a fatality, collect date of death.
- Schedule an appointment for the subject to return for the COVID-19 Convalescent Visit once he or she has recovered.
- The investigator or designee completes source documentation of visit and CRF.

11.2.11.4.2 COVID-19 Convalescent Visit

This visit is to be conducted in clinic, 28 to 35 days after the Suspected COVID-19 Illness Visit.

- Record AEs, as appropriate.

  Potential COVID-19 illnesses that are consistent with the clinical endpoint definition should not be recorded as AEs. These data will be captured as efficacy assessment data only on the relevant pages of the CRF, as these are expected endpoints.
- Record details of concomitant medication including any prohibited medications specified in Section 9.5.1 received by the subject if required for his or her clinical care.
- Collect blood samples for immunogenicity testing:
    - SARS-CoV-2 N protein antibody (Elecsys cobas or ELISA)
    - IgG S1-RBD ELISA and neutralization tests
- Collect/update COVID-19–related clinical and laboratory information
- The investigator or designee completes source documentation of visit and CRF.

11.2.12 Immunogenicity

Serum samples will be collected from subjects to test for antibodies elicited by study vaccine at time points shown in Table 1.

- SARS CoV-2 N protein antibody testing. (Arapid N-protein antibody test (authorized by the sponsor) can be performed any time during the screening period. The presence of N-protein antibodies in the serum collected after screening should be analyzed using a qualitativeimmuno test (Elecsys cobas Anti-SARS-CoV-2), except for the sub-study population immunized with the inactivated vaccines which will be tested using a quantitative N-protein ELISA test.

- SARS-CoV-2 neutralizing antibody titers (GMT) measured in Vero cells using replicating virus.
- SARS-CoV-2 IgG RBD ELISA antibody.

In addition, serum samples will be drawn from all subjects in the study potential further testing of vaccine-specific responses in subjects.

In selected subjects, the following cellular immune assays will be performed using frozen PBMC to determine the Th1/Th2 profile of the immune response to T-cell epitopes within the RBD and the peptide components of the vaccine.

- IFN-γ and IL-4 secreting cells/10^6 cells in ELISpot assays
- IFN-γ, IL-2, IL-4, and perforin by intracellular staining (ICS).

Also in selected subjects, serum samples will be collected to study Fc-mediated antibody dependent cellular responses (systems serology), including ADCP (antibody dependent cell-mediated phagocytosis).

Note: The subjects participating in the PBMC and ADCP blood draws may not be the same, but if selected to participate in one of the tests (PBMC and/or ADCP) the same subjects should remain contributing blood draws throughout the study visits.

11.3 Adverse Events

11.3.1 Definitions

11.3.1.1 Adverse Events

An AE is any untoward medical occurrence in a patient or clinical study subject, temporally associated with the use of study product, whether considered related to the study product. An AE can therefore be any unfavorable and unintended sign (including an abnormal laboratory finding), symptom, or disease (new or exacerbated) temporally associated with the use of study product.

Events Meeting the Adverse Event Definition

- Any abnormal laboratory test results (hematology, clinical chemistry, or urinalysis) or other safety assessments (e.g., ECG, radiological scans, vital sign measurements), including those that worsen from baseline, considered clinically significant in the medical and scientific judgment of the investigator. Any abnormal laboratory test results that meet any of the conditions below must be recorded as an AE:
    - Is associated with accompanying symptoms.
    - Requires additional diagnostic testing or medical/surgical intervention.
    - Leads to a change in study dosing (outside of any protocol-specified dose adjustments) or discontinuation from the study, significant additional concomitant drug treatment, or other therapy.
- Exacerbation of a chronic or intermittent preexisting condition including either an increase in frequency and/or intensity of the condition.
- New conditions detected or diagnosed after study product administration even though it may have been present before the start of the study.

- Signs, symptoms, or the clinical sequelae of a suspected drug-drug interaction.
- Signs, symptoms, or the clinical sequelae of a suspected overdose of either study product or a concomitant medication. Overdose per se will not be reported as an AE/SAE unless it is an intentional overdose taken with possible suicidal/self- harming intent. Such overdoses should be reported regardless of sequelae.

Events Not Meeting the Adverse Event Definition

- Any clinically significant abnormal laboratory findings or other abnormal safety assessments which are associated with the underlying disease, unless judged by the investigator to be more severe than expected for the subject's condition.
- The disease/disorder being studied or expected progression, signs, or symptoms of the disease/disorder being studied, unless more severe than expected for the subject's condition.
- Medical or surgical procedure (e.g., endoscopy, appendectomy): the condition that leads to the procedure is the AE.
- Situations in which an untoward medical occurrence did not occur (social and/or convenience admission to a hospital).
- Anticipated day-to-day fluctuations of preexisting disease(s) or condition(s) present or detected at the start of the study that do not worsen.

Solicited AEs are provided in Section 11.2.10.

11.3.1.2 Unsolicited Adverse Event

An unsolicited AE is any AE reported by the subject that is not specified as a solicited AE in the protocol or is specified as a solicited AE in the protocol but starts outside the protocol-defined period for reporting solicited AEs (seven (7) days following receipt of study product injection (up to and including Day 8)). Unsolicited adverse events will be recorded through Day 29 on a reactogenicity subject diary.

The severity of unsolicited AEs will be graded according to the FDA's toxicity grading scale (FDA, 2007). For the local and systemic AEs, the severity grading will be based upon the investigator's assessment of intensity for each AE following the categories: Grade 1 (Mild) = symptom present but does not interfere with activities of daily living; Grade 2 (Moderate) = symptom causes some interference with activities of daily living not requiring medical intervention; Grade 3 (Severe) = symptom prevents activities of daily living and requires medical intervention; Grade 4 (Potentially Life-Threatening) = Life-Threatening consequences; ER visit or hospitalization.

11.3.1.3 Medically Attended Adverse Event

An MAAE is an AE that leads to an unscheduled visit (including a telemedicine or telephone visit) to a healthcare practitioner (e.g., doctor's office). Subjects will be instructed to inform the site if a MAAE occurs. In addition, investigators will review unsolicited AEs for the occurrence of any MAAEs. All MAAEs will be reported on the MAAE page of the CRF. The site will collect information to include reason for consultation, diagnosis, severity (grade) of symptoms, treatment provided and outcome, and the Investigator will assess relatedness to study product injection.

11.3.1.4 Adverse Events of Special Interest (AESI)

AESIs identified in Appendix 7 represent a list suggested by the United States Food and Drug Administration as potential immune-mediated medical conditions. Any occurrence of these conditions during the study will be reported with a narrative and the assessment of the seriousness of the event and causal relationship to the vaccine.

11.3.1.5 Serious Adverse Events

An AE is considered serious (SAE) if, in the opinion of either the investigator or sponsor, it results in any of the following:

- Results in death
- Is life-threatening. Life-threatening means the subject was at immediate risk of death from the AE as it occurred (i.e., it does not include a AE which hypothetically might have caused death had it occurred in a more severe form).
- Requires inpatient hospitalization or prolongation of existing hospitalization. Hospitalizatios solely for the purpose of receiving supportive care treatment or hospitalization admissions and/or surgical operations scheduled to occur during the study period, but planned prior to study entry are not considered AEs if the illness or disease existed before the subject was enrolled in the study, provided that it did not deteriorate in an unexpected manner during the study (e.g., surgery performed earlier than planend).
- A persistent or significant incapacity or substantial disruption of the ability to conduct normal life functions. This definition is not intended to include experiences of relatively minor medical significance such as uncomplicated headache, nausea, vomiting, diarrhea, influenza, and accidental trauma (e.g., sprained ankle) which may interfere with or prevent everyday life functions but do not constitute a substantial disruption.
- A congenital anomaly or birth defect in a neonate/infant born to a mother exposed to study treatment.
- Important medical event. An important medical event is an event that may not result in death, be life-threatening, or require hospitalization but may be considered an SAE when, based upon appropriate medical judgment by the investigator or the sponsor, it may jeopardize the subject and may require medical or surgical intervention to prevent one of the outcomes listed in the definitions for SAEs. Examples of such medical events include allergic bronchospasm requiring intensive treatment in an emergency room or at home, blood dyscrasias or convulsions that do not result in in-subject hospitalization, or the development of drug dependency or drug abuse.

Suspected transmission of an infectious agent, pathogenic or nonpathogenic, via a Vaxxinity product is considered serious. The event may be suspected from clinical symptoms or laboratory findings indicating an infection in a subject exposed to a Vaxxinity product. The terms “suspected transmission” and “transmission” are considered synonymous. These cases are considered unexpected and handled as serious expedited cases by pharmacovigilance personnel. Such cases are also considered for reporting as product defects, if appropriate.

The terms “severe” and “serious” are not synonymous. Severity refers to the intensity of an AE (rated as mild, moderate, or severe); the event itself may be of relatively minor medical significance (such as severe headache without any further findings).

Severity and seriousness need to be independently assessed for each AE recorded on the CRF.

11.3.2 Collection of AEs, MAAEs, SAEs, and AESIs

Monitoring of AEs will be conducted throughout the study. All reported AEs will be recorded in the source documentation and CRF for each subject from the time the subject provides informed consent/assent, which is obtained before the subject's participation in the study (i.e., before undergoing any study-related procedure and/or receiving study product) through the 12-month study period. The active collection period for solicited local AEs and systemic events, unsolicited AEs, MAAE, SAEs, and AESIs is shown in Table 9.

Table 9: Active Collection Period for Adverse Events

Event	Collection Period	
	**From**	**To**
Solicited local and systemic AEs	Injection (Day 1)	Day 8<br>7 days (±1 day) after study product injection on Day 1
Unsolicited AEs	Injection (Day 1)	Day 15<br>14 days ((±1 day) after study product injection on Day 1
Unsolicited AEs	Injection (Day 1)	Day 29<br>28 days (±3 days) after study product injection on Day 1
MAAEs	Injection (Day 1)	Month 12<br>±14 days after study product injection on Day 1
SAEs and AESIs	Injection (Day 1)	Month 12<br>±14 days after study product injection on Day 1

Abbreviations: AE = adverse event; AESI = adverse event of special interest; MAAE = medically attended adverse event; SAE = serious adverse event

Follow-up on ongoing AEs by the investigator will continue throughout and after the active collection period and until the AE, MAAE, AESI, or SAE or its sequelae resolve or stabilize.

For subjects who are screen failures, the active collection of AEs period ends when screen failure status is determined.

If the subject withdraws from the study and withdraws consent/assent for the collection of future information, the active collection period ends when consent/assent is withdrawn.

If a subject definitively discontinues or temporarily discontinues study because of an AE, MAAE, SAE, or AESI, the event must be recorded on the CRF. The SAE and AESI must be reported using the SAE and AESI Report Form.

Investigators are not obligated to actively seek AEs, MAAEs, AESIs, or SAEs after the subject has concluded study participation. However, if the investigator learns of any SAE or AESI, including death, at any time after a subject has completed the study, and he or she considers the event to be reasonably related to the study product, the investigator must promptly report the event using the SAE and AESI Report Form.

11.3.3 Reporting and Follow-up of AEs, SAEs, and AESIs

All AEs (serious and non-serious) reported by the subject and/or in response to an open question from study personnel or revealed by observation, physical examination, or other

diagnostic procedures will be recorded on the appropriate page of the CRF. Any clinically relevant deterioration in laboratory assessments (see Section 11.3.6 for capturing abnormal laboratory findings as AEs) or other clinical findings is considered an AE and must be recorded on the appropriate pages of the CRF.

All SAEs and AESIs that occur during the study must be promptly reported by the investigator. Deaths and AEs assessed as life-threatening are to be reported immediately; SAEs or AESIs are to be reported within 24 hours from the time when the investigator becomes aware of the event. All SAEs and AESIs must be reported whether or not they are considered causally related to administration of study vaccine. An SAE and AESI Report Form will be completed, and the information collected will include the subject number, a narrative description of the event, and an assessment by the investigator as to the severity of the event and relationship to study vaccine.

After the initial SAE or AESI report, the investigator is required to proactively follow each subject at subsequent visits/contacts. For each event, the investigator must pursue and obtain adequate information until resolution, stabilization, the event is otherwise explained, or the subject is lost to follow-up.

In general, follow-up information will include a description of the event in sufficient detail to allow for a complete medical assessment of the case and independent determination of possible causality. Any information relevant to the event, such as concomitant medications and illnesses, must be provided. In the case of a subject death, a summary of available autopsy findings must be submitted to the sponsor as soon as possible.

If there are serious, unexpected adverse drug reactions associated with the use of the study vaccine under clinical investigation, the sponsor has a legal responsibility to notify the appropriate regulatory agency(ies) and all participating investigators on an expedited basis. The local International Review Board (IRB)/Independent Ethics Committee (IEC) will be promptly notified based on local regulations where required by the IRB/IEC of all serious, unexpected adverse drug reactions involving risk to human subjects.

11.3.3.1 Reporting of Myocarditis and Pericarditis

Cases of myocarditis or pericarditis occurring within 4 weeks of vaccination are considered to be potentially related, unexpected, and serious (as important medical events, even if other seriousness criteria are not met) and therefore, to meet criteria for expedited reporting, as required by 21 CFR 312.32. [THIS WILL NOT APPLY FOR PFIZER COVID VACCINE for which myocarditis/pericarditis is labeled/expected].

11.3.4 Assessment of Severity

The severity of all AEs, including clinically significant treatment-emergent laboratory abnormalities, will be assessed by the investigator as follows:

- **Mild**: The event is noticeable to the subject but doe not interfere with routine activity.
- **Moderate**: The event interferes with routine activity but responds to symptomatic therapy or rest.
- **Severe**: The event significantly limits the subject’s ability to perform routine activities despite symptomatic therapy.

- **Life-threatening**: An event in which the subject was at risk of death at the time of the event.
- **Fatal**: An event that results in the death of the subject.

11.3.5 Relationship to Study Vaccine Administration

The relationship to study vaccine administration will be determined by the investigator according to the following criteria:

- **Definitely related**: An AE is definitely related to study participation if it is clear that the event was caused by study participation. A definitely related event has a strong temporal relationship and an alternative cause is unlikely.
- **Probably related**: An AE is probably related when there is a reasonable possibility that the event is likely to have been caused by study participation. The AE has a timely relationship to the study procedure(s) and follows a known pattern of response, but a potential alternative cause may be present.
- **Possibly related**: An AE is possibly related when there is a reasonable possibility that the event might have been caused by study participation. A possibly related event may follow no known pattern of response and an alternative cause seems more likely. In other circumstances there may be significant uncertainty about the cause of the event, or a possible relationship to study participation cannot reasonably be ruled out.
- **Unlikely related:** An AE is unlikely related when there is no medical evidence to suggest that the AE is related to study participation. The event can be readily explained by the subject’s underlying medical condition or concomitant therapy or lacks a plausible temporal relationship to the study vaccine.
- **Unrelated**: The cause of the AE is known and the event is in no way related to any aspect of study participation. If there is any uncertainty regarding AE causality then the event must be assessed as unlikely related to research participation and reported to the IRB/IEC as indicated. Often, the cause of an unrelated AE is disease progression.

Investigators should use their knowledge of the subject, the circumstances surrounding the event, and an evaluation of any potential alternative causes to determine whether or not an AE is considered to be related to the study vaccine. The following guidance should be taken into consideration:

- Temporal relationship of event onset to the initiation of study vaccine.
- Course of the event, considering especially the effects of dose reduction, discontinuation of study vaccine, or reintroduction of study vaccine (where applicable).
- Known association of the event with the study vaccine or with similar treatments.
- Known association of the event with the disease under study.
- Presence of risk factors in the subject or use of concomitant medications known to increase the occurrence of the event
- Presence of nontreatment-related factors that are known to be associated with the occurrence of the event.

Investigators should use correct medical terminology/concepts when recording AEs on the CRF and avoid colloquialisms and abbreviations. Only 1 AE term should be recorded in each event field on the CRF.

11.3.6 Abnormal Laboratory Values

Not every laboratory abnormality qualifies as an AE. A laboratory test result should be reported as an AE if it meets any of the following criteria:

- Is accompanied by clinical symptoms
- Results in a change in study treatment (e.g., discontinuation of study vaccine)
- Results in a medical intervention (e.g., potassium supplementation for hypokalemia) or a change in concomitant therapy
- Is clinically significant in the investigator's judgment

The investigator will review all laboratory test results and assess for clinical significance using the grading scale in Appendix 6. Clinically significant abnormal laboratory findings are those which are not associated with a stable underlying disease, unless judged by the investigator to be more severe than expected for the subject's condition. Medical and scientific judgment should be exercised in deciding whether an isolated laboratory abnormality should be classified as an AE. Any clinically relevant changes judged by the investigator as an AE will be recorded in the CRF.

All laboratory tests with values considered clinically significantly abnormal during participation in the study or within 28 days after study product injection should be repeated until the values return to normal or baseline or are no longer considered clinically significant by the investigator or medical monitor. If such values do not return to normal/baseline within a period of time judged reasonable by the investigator, the etiology should be identified, and the sponsor notified.

11.3.6.1 Pregnancy

Pregnancy is neither an AE nor an SAE, unless a complication relating to the pregnancy occurs (e.g., spontaneous abortion, which may qualify as an SAE). Subjects who become pregnant during the study will continue to have all study defined safety and immunogenicity assessments.

Pregnancies and suspected pregnancies (including a positive pregnancy test regardless of age or disease state) of a female subject or partner of a male subject occurring during the study are considered immediately reportable events. If a female partner of a male subject taking the study vaccine becomes pregnant, the male subject should notify the investigator, and the pregnant female partner should be advised to call her healthcare provider immediately. The pregnancy, suspected pregnancy, or positive pregnancy test must be reported immediately using the Pregnancy Report form. The investigator must follow-up and document the course and outcome of all pregnancies even if the subject was discontinued from the study or if the subject has completed the study. The female subject or partner of a male subject should receive any necessary counseling regarding the risks of continuing the pregnancy and the possible effects on the fetus. Monitoring should continue until conclusion of the pregnancy.

All outcomes of pregnancy (from a female subject or the sexual partner of a male subject) must be reported by the investigator to the sponsor or medical monitor within 30 days after he/she has gained knowledge of the delivery or elective abortion.

Any SAE that occurs during pregnancy must be recorded on the SAE and AESI report form (e.g., maternal serious complications, spontaneous or therapeutic abortion, ectopic pregnancy, stillbirth, neonatal death, congenital anomaly, birth defect) and reported **within 24 hours in accordance with the procedure for reporting SAEs and AESIs.**

11.3.7 Medication Errors

Medication errors may result from the administration or consumption of the study product by the wrong subject, or at the wrong time, or at the wrong dosage strength. Medication errors include:

- Medication errors involving subject exposure to the study product;
- The administration of an incorrect study product;
- The administration of an incorrect dosage;
- The administration of study vaccine that has undergone temperature excursion from the specified storage range, unless it is determined by the sponsor that the study product under question is acceptable for use.

All medication errors during the study are to be captured on the medication error page of the CRF, which is a specific version of the AE page. Whether or not the medication error is accompanied by an AE, as determined by the investigator, the medication error is recorded on the medication error page of the CRF and, if applicable, any associated AE(s), serious and nonserious, are recorded on the AE page of the CRF.

In the event of a medication dosing error, the sponsor should be notified within 24 hours. If the medication error is associated with an SAE or AESI, the sponsor should be notified within 24 hours using a Vaccine SAE and AESI Report Form.

11.3.8 Safety Monitoring Plan

The Safety Monitoring Plan (SMP) will describe the procedures for review of safety data during the study to ensure the safety of study subjects. The procedures will include a blinded review of all Grade 3 AEs, SAEs, MAAEs, and AESIs at regular intervals throughout the study.

For double-blind sub-studies, care will be taken to prevent the unblinding of any study subjects when safety reviews are conducted. However, there may be specific circumstances that occur that may necessitate unblinding to properly investigate a reported event. In these circumstances knowledge of the unblinding will be carefully controlled and not provided to sponsor or contract research organization (CRO) personnel directly involved with study conduct.

11.3.9 Independent Data and Monitoring Committee (IDMC)

An external, independent IDMC will be chartered to review the study data during the conduct of the trial, provide recommendation regarding safety which may impact the conduct of the trial. The IDMC will be composed of at least 3 clinicians knowledgeable about vaccine clinical research and a biostatistician.

The details of membership and responsibilities, including frequency of review, will be delineated in the Safety Monitoring Plan and IDMC Charter.

12 STATISTICS

This section describes the statistical methods and analyses for the primary and secondary endpoints which intend to be used across all sub-studies under this platform study. If there is any specific analysis required for a sub-study, it will be described under the similar section in the sub-study. The statistical analysis plan (SAP) for the platform trial will be developed to describe all elements for the execution of the analysis before the first database lock of the sub-study. Should any changes of the analysis plan for a sub-study, it will be amended in the sub-study SAP before the database lock of the sub-study.

12.1 Hypothesis Testing

The primary hypothesis of the study is to demonstrate non-inferiority of a heterologous third-dose booster with UB-612 following primary vaccine sequences with a comparator vaccine relative to a homologous third-dose booster with the same comparator vaccine as measured by neutralizing antibody GMR post-boost at Day 29 using SARS-CoV-2 Wuhan strain. The secondary hypothesis is to demonstrate non-inferiority of a heterologous third-dose booster with UB-612 relative to a homologous third-dose booster with the same comparator vaccine as measured by neutralizing antibody GMR post-boost at Day 29 using Omicron variant.

All hypotheses will be tested to establish conclusions separately for each comparator vaccine for each sub-study; therefore, no statistical adjustment for multiplicity across sub-studies will be applied. In the situation of both double-blinded and open-label sub-studies are performed with the same comparator, the hypothesis test will be based on both studies pooled data, and the test of the impact of the study type will be performed, and the study type will be added as a covariate in the analysis to reduce the variability if there are any.

A statistical test for superiority of a heterologous boost with UB-612 vs homologous boost will be performed if the primary analysis results in a conclusion of non-inferiority. Since the analysis of superiority is conditional on significance of non-inferiority, no further alpha-adjustment for multiplicity is required.

12.2 Power and Sample Size

The sample size is determined by targeting at least 90% power (BNT162b2 Sub-studies and BIBP Sub-study) and 80 to 85% power (ChAdOx1-S Sub-studies) with 1-sided alpha of 0.025 to test non-inferiority based on the GMT ratio using a margin ratio of GMT of 1.5.

The sample size calculation for the non-inferiority evaluation is based on all subjects (seronegative and seropositive), with an additional 25% of that sample size allocated for dropouts and major protocol deviations in the BNT162b2 Sub-studies and BIBP Sub-study. During the enrollment of the ChAdOx1-S Sub-studies, operational difficulties precluded attainment of the full planned enrollment. Therefore, the final sample size achievable does not account for dropouts for any reason.

There is limited data available from the comparator vaccines to establish the sample size calculation. Below is a summary of the required sample size for the hypothysis test of each comparasion and the assumptions used.

12.2.1 BNT162b2 Sub-studies

For the primary endpoint of GMT ratio, assuming a log10 standard deviation of 0.38 and a difference in log10 GMT of -0.038 (equivalent to a GMT ratio of 0.92), a sample size of

320 (160 per arm) will have 90% power to establish non-inferiority, with a non-inferiority margin of 1.5.

A total of 400 subjects will be required from both double-blinded and open label sub-studies enrolled to account for an additional 25% of that sample size allocated for dropouts and major protocol deviations.

12.2.2 ChAdOx1-S Sub-studies

For the primary endpoint of GMT ratio, assuming a log10 standard deviation of 0.40 (based on neutralizing antibodies vs alpha strain [Flaxman et al, 2021]), the achievable sample size in these sub-studies is approximately 190. This sample size will have 80 to 85% power to establish non-inferiority, with a non-inferiority margin of 1.5, if no subjects are lost prior to the assessment of the primary endpoint at Day 29.

12.2.3 BIBP Sub-study

For the primary endpoint of GMT ratio, assuming a log10 standard deviation of 0.44 (based on 6 µg group [Kanokudom et al, 2021]), a sample size of 266 (133 per arm) will have 90% power to establish non-inferiority, with a non-inferiority margin of 1.5. A total of 334 subjects will be enrolled and the sample size also includes an additional 25% of participants of that sample size allocated for dropouts and major protocol deviations.

12.3 Interim Analysis

There is no interim analysis planned for this study.

12.4 Missing Data

Immunogenicity analysis will be performed on all available immune parameters at any given timepoint; imputation of missing immunogenicity results will not be performed. Participants who test positive for SARS-CoV-2 exposure after boost vaccination may be excluded from the primary immunogenicity analysis as the study progresses.

12.5 Analysis Sets

For the purposes of analysis, the following populations are defined:

- **Intent to Treat (ITT) Population**: All eligible subjects who are randomized to the study treatment. This population will be used for summary of baseline characteristics
- **Modified ITT Population**: all ITT subjects who recieve study vaccine with no major protocol deviations which would directly impact assessments of immunogenicity. This population will be used for the primary analysis of immunogenicity.
- **Safety Population**: All ITT subjects who received study vaccine. This population will be used for all safety analyses.

12.6 Statistical Methods

12.6.1 Analysis of Immunogenicity

The primary immunogenicity analysis will evaluate non-inferiority between UB-612 heterologic boosting and the comparator homologous boosting. Separate conclusions will be made regarding vaccine non-inferiority of heterologous to homologous boost for each comparator.

The comparison to the authorized product is to show that UB-612 can be used interchangeably with the homologous vaccine as a booster to evoke a non-inferior increase in neutralizing antibody titer. Humoral immunity endpoints (neutralizing antibody titers) will be determined at baseline (Day 1), Day 15, Day 29, and 6- and 12-months post-injection, with the primary analyses conducted on immunogenicity results at Day 29. The justification for choosing Day 29 for primary analysis is that humoral responses typically achieve peak titers 2-4 weeks after immunization, and regulatory authorizations for comparator vaccines typically described neutralizing antibodies after the primary series or the booster dose determined 1 month after the injection.

The primary immunogenicity analyses will employ the SARS CoV-2 Wuhan strain in a replicating, or pseudotyped virus neutralization test controlled with the WHO reference antibody reagent and expressed in international units (IU)/mL. A second justification for use of the Wuhan strain is that the comparator vaccines were originally shown in large placebo-controlled studies to be effective in preventing COVID-19 caused by this virus strain. Other variants of concern, including Omicron, will be evaluated in the secondary analysis using the same methodology. Analyses of other immunogenicity objectives will be to descriptively compare the treatment groups in terms of neutralizing antibody GMT, geometric mean fold increase (GMFI) and reverse cumulative distribution curve (RCDC) responses to SARS-CoV-2 variants of concern. The ratio of the GMT for the prototype and the variant strain will be evaluated using descriptive statistics. These tests will also employ replicating or pseudotyped virus assays, but, for lack of reference sera, results are expressed as 50% endpoint titers.

Durability of the neutralizing antibody response to Wuhan and Omicron variant viruses of concern will be assessed by the area under the curve (AUC) of the antibody response between Day 15 and Month 12. The half-life of neutralizing antibodies will be reported for the Month 6 and 12 timepoints.

12.6.1.1 Primary Endpoints and Analysis

The primary endpoint will be tested with intention to demonstrate that the immune response to UB-612 100 μg at 29 days after boosting is non-inferior to that observed for homologous boosting with a comparator vaccine (pairwise comparison of treatment subgroups) based on SARS-CoV-2 50% neutralizing titers to Wuhan in IU/mL (using the WHO reference serum). The criteria for non-inferiority are prespecified margins for the ratio of GMT assessed 1 month after the boost. Analysis of GMT ratio will be performed on the log-transformed (base 10) titers, a standard approach since log-transformation produces values that are approximately normally distributed. Noninferiority is determined if the lower bound of the 2-sided 95.0% confidence interval (CI) for the GMTR (GMT UB-612 group/GMT Comparator group) is >0.67. Analyses will be stratified by age group, gender, N-protein seropositivity, and the time interval from the last dose of completed primary vaccination and adjusted for log baseline titers; however, no separate subgroup inferences are intended. For descriptive purposes, results may

be presented by age group, gender, and the time interval from the last dose of the primary series subgroups.

12.6.1.2 Secondary Endpoints and Analysis

The secondary immunogenicity endpoints are neutralizing antibody responses for determination of GMT and seroresponse rate measured in live or pseudotyped virus tests employing the Wuhan and Omicron variant at Day 15 and 29, and Months 6 and 12. Duration of immunity will be compared by GMT, GMFI, geometric mean titer ratio and seroresponse rates, with additional analyses for AUC and antibody half-life. Additional secondary analyses, including GMT, GMFI, GMR, AUC, half-life, and seroresponse rates, will be performed for IgG antibodies binding S1-RBD in ELISA.

12.6.1.3 Subset Analyses

Each sub-study will select a subset of approximately 10% of subjects, in proportion to the study size, serum samples for Fc-mediated assays and neutralization against additional SARS-CoV2 variants. Approximately 10% of subjects selected from the Pfizer sub-study will provide PBMC samples. The exploratory immunogenicity objectives will be assessed by cellular immunity endpoints (the number of IFN-γ and IL-4 secreting cells/10^6 cells determined by ELISpot assays and the percent of cells staining for IFN-γ, IL-2, IL-4, IL-10, TNF-α and granzyme B) and summarized at baseline (Day 1), Day 15, Day 29, and 6- and 12-months post-injection.

Exploratory analyses will also include:

- Determination of Fc-mediated (systems serology) assays: ADCP (antibody dependent cell-mediated phagocytosis)
- Live or pseudotyped virus neutralization titers to additional SARS-CoV-2 variants

12.6.2 Analysis of Safety

Subjects will complete Subject Diaries through Day 29 following study product injection. Subjects will be asked to report any MAAE, SAE and AESI through 12 months after vaccination.

Safety endpoints (solicited local and systemic AEs through 7 days after study product injection, unsolicited AEs through 29 days after study product injection, and MAAEs, SAEs, and AESIs through 12 months after study product injection) will be summarized descriptively by pre-study treatment (i.e., previous vaccine), study treatment, and age and gender group. An independent IDMC will be engaged to review safety at specific time points. AESIs monitored will include pericarditis, myocarditis, and thrombosis with thrombocytopenia syndrome, as well as all AESIs listed in Appendix 7.

Solicited local and systemic reactions after study product injection will be presented by severity and cumulatively across severity levels. Descriptive summary statistics will include counts and percentages of subjects with the indicated endpoint and the associated 95% confidence intervals. Refer to Table 4 and Table 5 for local and systemic solicited AEs, respectively, and for the severity grade on the ordinal integer scale from 1 to 4. A category of 0 will denote instances where the event did not occur at a given time. Summaries will be presented by treatment group within each previous vaccination group. To assess the strength of association between age and severity, presence/absence of comorbidities and severity, and treatment and

severity, an ordinal logistic regression (cumulative logit) model will be employed. Odds ratios and nominal (i.e., unadjusted) 95% CIs will be constructed to assess the impact of the independent variable (age, comorbidities, or treatment) on the likelihood of a more severe outcome. In addition, a multivariable logistic regression model will be employed to assess the impact of treatment on severity, adjusting for age and presence/absence of comorbidities. These analyses will be performed on the pooled previous vaccination groups. An additional exploratory analysis that subdivides participants into those who are seropositive vs seronegative at Screening will be performed for solicited local and systemic reactions. Also, the analysis of blinding impact will be performed on subjects reporting safety after a common IP injection with the data collected in a blinded and open-label fashion.

Descriptive statistics will be provided for abnormal hematology chemistry and hs-CRP laboratory values at 7 and 28 days after study product injection, including grading shifts in hematology and chemistry laboratory assessments from baseline. Descriptive statistics for systolic blood pressure, diastolic blood pressure, heart rate and temperature at baseline, each scheduled post-baseline visit, the last study visit, and the change from baseline will be provided.

13 QUALITY CONTROL AND QUALITY ASSURANCE

13.1 Responsibility of the Investigator(s)

The investigator(s) undertake(s) to perform the clinical study in accordance with this clinical study protocol, International Council for Harmonisation (ICH) guidelines for GCP and the applicable regulatory requirements.

The investigator is required to ensure compliance with all procedures required by the clinical study protocol and with all study procedures provided by the sponsor, including security rules. The investigator agrees to provide reliable data and all information requested by the clinical study protocol in an accurate and legible manner according to the instructions provided and to ensure direct access to source documents by sponsor representatives.

In the event there is any transfer of data, particular attention should be paid to the confidentiality of the subject's data to be transferred.

The investigator may appoint such other individuals, as he/she may deem appropriate as sub-investigators to assist in the conduct of the clinical study in accordance with the clinical study protocol. All co-investigators shall be appointed and listed in a timely manner. The co-investigators will be supervised by and work under the responsibility of the investigator. The investigator will provide them with a copy of the clinical study protocol and all necessary information.

13.2 Responsibility of the Sponsor

The sponsor of this clinical study is responsible to health authorities for taking all reasonable steps to ensure the proper conduct of the clinical study protocol with regard to ethics, clinical study protocol compliance, and integrity and validity of the data recorded on the case report forms. Thus, the main duty of the sponsor or sponsor's delegate is to help the investigator and the sponsor maintain a high level of ethical, scientific, technical, and regulatory quality in all aspects of the clinical study.

At regular intervals during the clinical study, the site will be contacted, through monitoring visits, letters, or telephone calls, by a representative of the sponsor or sponsor's delegate to review study progress, investigator and subject compliance with clinical study protocol requirements and any emergent problems. These monitoring visits will include but are not limited to review of the following aspects: subject informed consent/assent, subject recruitment and follow-up, SAE documentation and reporting, AE documentation, study drug allocation, subject compliance with the study drug regimen, study drug accountability, concomitant therapy use and quality of data.

13.3 Source Document Requirements

According to the ICH guidelines for GCP, the sponsor or sponsor's delegate must check the CRF entries against the source documents, except for the pre-identified source data directly recorded in the CRF. The informed consent/assent form will include a statement by which the subject allows the sponsor's duly authorized personnel, the IRB/IEC, and the regulatory authorities to have direct access to original medical records which support the data on the CRF (e.g., subject's medical file, appointment books, original laboratory records). These personnel, bound by professional secrecy, must maintain the confidentiality of all personal identity or personal medical information, according to confidentiality rules.

13.4 Use and Completion of Case Report Forms (CRFs) and Additional Request

It is the responsibility of the investigator to maintain adequate and accurate CRFs designed by the sponsor to record (according to sponsor instructions) all observations and other data pertinent to the clinical investigation. All CRFs should be completed in their entirety to ensure accurate interpretation of data.

Data are available within the system to the sponsor as soon as they are entered in the CRF.

The computerized handling of the data by the sponsor after receipt of the CRFs may generate additional requests to which the investigator is obliged to respond by confirming or modifying the data questioned. The requests with their responses will be managed through the CRF.

14 ETHICS

14.1 Declaration of Helsinki and Ethical Review

The study will be performed in accordance with the principles stated in the Declaration of Helsinki. The IRB/IEC must approve the study protocol and informed consent/assent form before the enrolment of subjects. The views of the IRB/IEC should be dated and filed. The names and titles of those who attend the IRB/IEC meeting should be attached. After receiving the approval letter from the IRB/IEC, the investigators have the responsibility to forward the copy of the approved letter to the Sponsor or its designee before the commencement of the clinical study.

The investigator is responsible for informing the IRB/IEC of any serious adverse events and/or major amendments to the protocol as per local requirements. The investigator should file all correspondence with the IRB/IEC.

14.2 Patient Information and Consent/Assent

The investigator will ensure that the subject and his/her caregiver or legal guardian, as needed, are given full and adequate verbal and written information about the nature, purpose, possible risk and benefit of the clinical study. Subjects and their caregivers or legal guardians must also be informed that they are free to discontinue their participation in the clinical study at any time. The investigator must see the signed informed consent/assent before enrolment.

The subjects should have a copy of the ICF and, if applicable, Assent Form. If any modifications are to be made according to local requirements, the new version must be approved by the Sponsor and IRB/IEC as well.

14.3 Patient Data Protection

In the CRFs, all subjects should be identified by number, initials, date of birth and sex at birth, as allowed by national and local regulations. The investigator is responsible for keeping a name list of all subjects including subjects' numbers, full name and last known address.

The subjects will be informed in the ICF about the possibility of audits by authorized representatives of the Sponsor and/or regulatory authorities in which case a review of those parts of the hospital records relevant to the study may be required. However, the investigator will follow all applicable privacy laws in order to protect a subject's privacy and confidentiality.

14.3.1 Biological Samples

Blood samples will be labeled with a code so that the laboratory personnel testing the samples will not know the subject's identity. Samples for future research as outlined in the protocol may be stored by Vaxxinity for up to 25 years, or as per local requirements, after the end of the study and then destroyed.

If permitted by the IEC-approved ICF, stored samples may be used for additional tests on immune responses to the vaccine under study in this protocol or related vaccines. The samples may be shared with other researchers as long as anonymity is maintained, and no testing of the subject's DNA is performed.

No genetic testing of the subject's DNA will be performed.

The subject may request that his or her samples, if still identifiable, be destroyed at any time; however, any data already collected from those samples will still be used for this research.

15 DATA HANDLING AND RECORD KEEPING

15.1 Data Management

The investigator will ensure that the data are correctly recorded in the CRF. Any data corrections should be verified and recorded by the investigator.

The sponsor or its delegate will be responsible for data management of the study. The responsibilities include database setup, entry screen generation, data entry and verification, data query/resolution, data clean-up, and data lock. Database will be converted into SAS dataset for statistical analysis.

15.2 Record Retention in Study Sites

The investigator must maintain all confidential study documentation and take measures to prevent accidental or premature destruction of these documents.

The investigator must retain the study documents at least 25 years after the end of the study, however, applicable regulatory requirements should be taken into account in the event that a longer period is required.

The investigator must notify the sponsor prior to destroying any study essential documents following the clinical study completion or discontinuation.

If the investigator's personal situation is such that archiving can no longer be ensured by him/her, the investigator shall inform the sponsor and the relevant records shall be transferred to a mutually agreed upon designee.

16 FINANCING AND INSURANCE

The sponsor certifies that it has taken out a liability insurance policy covering all clinical studies under its sponsorship. This insurance policy is in accordance with local laws and requirements. The insurance of the sponsor does not relieve the investigator and the collaborators from maintaining their own liability insurance policy. An insurance certificate will be provided to the IEC or health authorities in countries requiring this document.

17 PUBLICATION POLICY

Vaxxinity follows local regulatory requirements relating to clinical study registration and disclosure of results.

Vaxxinity commits to seek publication of results of its completed applicable clinical studies on any marketed product in the peer-reviewed scientific literature, regardless of study outcome. Vaxxinity supports recognized standards concerning authorship and publication.

Vaxxinity will provide final statistical reports of protocol-derived outcomes to external authors. Vaxxinity reserves the right to review and comment on draft abstracts, manuscripts, presentations and other communications by external investigators regarding Vaxxinity - sponsored studies, prior to submission or public disclosure, in order to protect intellectual property and confidential information. As study sponsor, Vaxxinity does not approve or veto such publications.

18 REFERENCES

Flaxman A, Marchevsky NG, Jenkin D. et al. Reactogenicity and immunogenicity after a late second dose or a third dose of ChAdOx1 nCoV-19 in the UK: a substudy of two randomised controlled trials (COV001 and COV002). Lancet. 2021 Sep 11;398(10304):981-990. doi: 10.1016/S0140-6736(21)01699-8. Epub 2021 Sep 1. PMID: 34480858; PMCID: PMC8409975.

FDA. Guidance for Industry. Toxicity Grading Scale for Healthy Adult and Adolescent Volunteers Enrolled in Preventive Vaccine Clinical Trials. September 2007

Kanokudom K, Assawakosri S, Suntronwong N, Auphimai C, Nilyanimit P, Vichaiwattana P, et al. Safety and immunogenicity of the third booster dose with inactivated, viral vector, and mRNA COVID-19 vaccines in fully immunized healthy adults with inactivated vaccine. medRxiv 2021. Available at: https://www.medrxiv.org/content/10.1101/2021.12.03.21267281v1.full-text. Accessed 05 January 2022.

Petrosillo N, Viceconte G, Ergonul O, Ippolito G, Petersen E. COVID-19, SARS and MERS: are they closely related? Clin Microbiol Infect. 2020;26(6):729-734.

Wang C, Horby PW, Hayden FG, Gao GF. A novel coronavirus outbreak of global health concern. Lancet. 2020;395(10223):470-473.

Zent O, Arras-Reiter C, Broeker M, Hennig R. Immediate allergic reactions after vaccinations--a post-marketing surveillance review. Eur J Pediatr. 2002;161(1):21-5.

19 APPENDICES

Appendix 1: Double-Blind Evaluation of UB-612 as a Heterologous Boost and BNT162b2 SARS CoV-2 Vaccine as a Homologous Boost. Sub Study to the Platform Protocol: A Phase 3 Multi-Center Platform Randomized, Active-Controlled Trial to Compare Homologous Boost of Authorized COVID-19 Vaccines and Heterologous Boost with UB-612 83
Appendix 2: Open-Label Evaluation of UB-612 as a Heterologous Boost and BNT162b2 SARS CoV-2 Vaccine as a Homologous Boost. Sub-study to the Platform Protocol: A Phase 3 Multi-Center Platform Randomized, Active-Controlled Trial to Compare Homologous Boost of Authorized COVID-19 Vaccines and Heterologous Boost with UB-612 85
Appendix 3: Double-Blind Evaluation of UB-612 as a Heterologous Boost and ChAdOx1-S SARS CoV-2 Vaccine as a Homologous Boost. Sub-study to the Platform Protocol: A Phase 3 Multi-Center Platform Randomized, Active-Controlled Trial to Compare Homologous Boost of Authorized COVID-19 Vaccines and Heterologous Boost with UB-612 87
Appendix 4: Open-Label Evaluation of UB-612 as a Heterologous Boost and ChAdOx1-S SARS CoV-2 Vaccine as a Homologous Boost. Sub-study to the Platform Protocol: A Phase 3 Multi-Center Platform Randomized, Active-Controlled Trial to Compare Homologous Boost of Authorized COVID-19 Vaccines and Heterologous Boost with UB-612 89
Appendix 5: Double-Blind Evaluation of UB-612 as a Heterologous Boost and BIBP COVID-19 Vaccine as a Homologous Boost. An Appendix to the Platform Protocol: A Phase 3 Multi-Center Platform Randomized, Active-Controlled Trial to Compare Homologous Boost of Authorized COVID-19 Vaccines and Heterologous Boost with UB-612 91
Appendix 6: Laboratory Abnormality Grading Scale 94
Appendix 7: List of Potentially Immune-Mediated Medical Conditions 95
Appendix 8: WHO Clinical Progression Scale 99

APPENDIX 1: DOUBLE-BLIND EVALUATION OF UB-612 AS A HETEROLOGOUS BOOST AND BNT162B2 SARS COV-2 VACCINE AS A HOMOLOGOUS BOOST. SUB STUDY TO THE PLATFORM PROTOCOL: A PHASE 3 MULTI-CENTER PLATFORM RANDOMIZED, ACTIVE-CONTROLLED TRIAL TO COMPARE HOMOLOGOUS BOOST OF AUTHORIZED COVID-19 VACCINES AND HETEROLOGOUS BOOST WITH UB-612

Sub-study Design

This is a double blinded, multi-center international sub-study. A total of 400 subjects planned to be enrolled. It is estimated that up to 600 subjects may be screened for the study to ensure required sample size. Sub-study Specific Entrance Criteria

In addition to the criteria specified in Section 8.1 and Section 8.2, subjects must meet the following criteria to be eligible for participation in this sub-study:

Inclusion Criteria

In addition to the criteria specified in Section 8.1, subjects must meet the following criterion to be eligible for participation in this sub-study:

A1-1. Fully vaccinated with 2 injections of Pfizer/BioNTech BNT162b2 vaccine. The last dose of the Pfizer/BioNTech BNT162b2 vaccine must have been administered at least five (5) months from the study Day 1, taking into consideration the local and national regulations. Documentation, such as the National Health Service (NHS) COVID Pass, United States Centers for Disease Control vaccine card, or equivalent documentation (e.g., medical records, vaccine passport; in accordance with local approved vaccination record documentation) will be required for proof of vaccination, vaccine manufacturer and vaccination dates.

Exclusion Criteria

There are no sub-study-specific exclusion criteria.

Comparative Vaccine Information: BNT162b2 (Pfizer/BioNTech)

BNT162b2 (Pfizer/BioNTech) is a sterile suspension for injection for IM use.

Each 0.3 mL dose of BNT162b2 also includes the following ingredients: lipids (0.43 mg ((4-hydroxybutyl)azanediyl)bis(hexane-6,1-diyl)bis(2-hexyldecanoate), 0.05 mg 2-(polyethylene glycol 2000)-N,N-ditetradecylacetamide, 0.09 mg 1,2-distearoyl-sn-glycero-3-phosphocholine, and 0.2 mgk cholesterol), 0.01 mg potassium chloride, 0.01 mg monobasic potassium phosphate, 0.36 mkg sodium chloride, 0.07 mg dibasic sodium phosphate dihydrate, and 6 mg sucrose. The diluent (0.9% Sodium Chloride Injection, USP) contributes an additional 2.16 mg sodium chloride per dose. BNT162b2 does not contain preservative.

BNT162b2 is supplied as a frozen suspension in multiple dose vials; each vial must be diluted with 1.8 mL of sterile 0k.9% Sodium Chloride Injection, USP prior to use to form the vaccine. Each dose of BNT162b2 contains 30 µg of a nucleoside-messenger ribonucleic acid (mRNA) encoding the viral S glycoprotein of SARS-CoV-2.

The vial stoppers are not made with natural rubber latex.

BNT162b2 Dosing Regimen

Subjects assigned to the BNT162 arm will receive BNT162b2 0.3 ml dose (30 µg) COVID-19 mRNA Vaccine BNT162b2 IM on Day 1 in a double-blind fashion.

The study vaccine will be administered as an IM injection into the deltoid muscle, preferably of the nondominant arm, by an unblinded administrator. To maintain the blind to the subject and other study team members, the barrel of the syringe containing study vaccine (prepared by the unblinded study pharmacist) will be masked.

The study vaccines will be administered by an appropriately qualified and trained member of the study staff (e.g., physician, nurse, physician's assistant, nurse practitioner, pharmacist, or medical assistant) as allowed by local, state, and institutional guidance. The date, time, and vaccine injection site will be recorded in the source documentation and CRF.

All subjects will be observed by a trained site staff member for at least 30 (±5) minutes after being vaccinated. Appropriate medication and other supportive measures for management of an acute hypersensitivity reaction will be available at the site in accordance with local guidelines for standard immunization practices.

BNT162b2 (Pfizer/BioNTech) Formulation, Appearance, Packaging, and Labelling

BNT162b2 solution for injection is a white to off-white frozen solution.

Refer to the BNT162b2 Regulation 174 Information for United Kingdom (UK) healthcare professionals for further information:

https://assets.publishing.service.gov.uk/government/uploads/system/uploads/attachment_data/file/1020925/Temporary_Authorisation_HCP_Information_BNT162_-_24-09-2021.pdf

BNT162b2 (Pfizer/BioNTech) Preparation

Refer to the BNT162b2 Regulation 174 Information for UK healthcare professionals for vaccine preparation information:

https://assets.publishing.service.gov.uk/government/uploads/system/uploads/attachment_data/file/1020925/Temporary_Authorisation_HCP_Information_BNT162_-_24-09-2021.pdf

Comparator Arm-specific Sub-study Concomitant Medication Requirements or Restrictions

There are no specific requirements or restrictions regarding concomitant medications during the study beyond those specified in Section 9.5.

Comparator Arm-specific Sub-study Assessments

There are no additional tests or procedures specific to this sub-study.

Comparator Arm-specific Sub-study Statistical Methods

Statistical Methods

There are no sub-study-specific statistical methods beyond those specified in Section 12.6.

APPENDIX 2: OPEN-LABEL EVALUATION OF UB-612 AS A HETEROLOGOUS BOOST AND BNT162B2 SARS COV-2 VACCINE AS A HOMOLOGOUS BOOST. SUB-STUDY TO THE PLATFORM PROTOCOL: A PHASE 3 MULTI-CENTER PLATFORM RANDOMIZED, ACTIVE-CONTROLLED TRIAL TO COMPARE HOMOLOGOUS BOOST OF AUTHORIZED COVID-19 VACCINES AND HETEROLOGOUS BOOST WITH UB-612

This is an open label sub-study. After subject randomized to the study, subject will be administered assigned vaccine as described in this appendix.

Sub-study Specific Entrance Criteria

In addition to the criteria specified in Section 8.1 and Section 8.2, subjects must meet the following criteria to be eligible for participation in this sub-study:

Inclusion Criteria

In addition to the criteria specified in Section 8.1, subjects must meet the following criterion to be eligible for participation in this sub-study:

A2-1 Fully vaccinated with 2 injections of Pfizer/BioNTech BNT162b2 vaccine. The last dose of the Pfizer/BioNTech BNT162b2 vaccine must have been administered at least five (5) months from the study Day 1, taking into consideration the local and national regulations. Documentation, such as the National Health Service (NHS) COVID Pass, United States Centers for Disease Control vaccine card, or equivalent documentation (e.g., medical records, vaccine passport; in accordance with local approved vaccination record documentation) will be required for proof of vaccination, vaccine manufacturer and vaccination dates.

Exclusion Criteria

There are no sub-study-specific exclusion criteria.

Comparative Vaccine Information: BNT162b2 (Pfizer/BioNTech)

BNT162b2 (Pfizer/BioNTech) is a sterile suspension for injection for IM use.

Each 0.3 mL dose of BNT162b2 also includes the following ingredients: lipids (0.43 mg ((4-hydroxybutyl)azanediyl)bis(hexane-6,1-diyl)bis(2-hexyldecanoate), 0.05 mg 2-(polyethylene glycol 2000)-N,N-ditetradecylacetamide, 0.09 mg 1,2-distearoyl-sn-glycero-3-phosphocholine, and 0.2 mgk cholesterol), 0.01 mg potassium chloride, 0.01 mg monobasic potassium phosphate, 0.36 mkg sodium chloride, 0.07 mg dibasic sodium phosphate dihydrate, and 6 mg sucrose. The diluent (0.9% Sodium Chloride Injection, USP) contributes an additional 2.16 mg sodium chloride per dose. BNT162b2 does not contain preservative.

BNT162b2 is supplied as a frozen suspension in multiple dose vials; each vial must be diluted with 1.8 mL of sterile 0k.9% Sodium Chloride Injection, USP prior to use to form the vaccine. Each dose of BNT162b2 contains 30 µg of a nucleoside-mRNA encoding the viral S glycoprotein of SARS-CoV-2.

The vial stoppers are not made with natural rubber latex.

BNT162b2 Dosing Regimen

Subjects assigned to the BNT162b2 arm are to receive BNT162b2 0.3 ml dose (30 µg) COVID-19 mRNA Vaccine BNT162b2 IM on Day 1 in an open-label fashion. Subjects are to receive BNT162b2 vaccine at a local vaccination location (eg, doctor's office, clinic, pharmacy) of their choice, provided the local vaccination location is operating by local and national regulations. After receiving the comparator vaccine, the subject is required to provide proof of vaccination to the study center to continue participation in the study. As a minimum, the date, time (if available), vaccine injection site, the name of vaccine, lot number, and expiration date should be captured and shared with the study staff during the next visit.

The study vaccine will be administered as an IM injection into the deltoid muscle, preferably of the nondominant arm, accordingly to standard institutional practice.

Subjects will be observed post-vaccination according to standard institutional practice. Appropriate medication and other supportive measures for management of an acute hypersensitivity reaction will be available at the site in accordance with local guidelines for standard immunization practices.

BNT162b2 (Pfizer/BioNTech) Formulation, Appearance, Packaging, and Labelling

BNT162b2 solution for injection is a white to off-white frozen solution.

Refer to the BNT162b2 Regulation 174 Information for UK healthcare professionals for further information:

https://assets.publishing.service.gov.uk/government/uploads/system/uploads/attachment_data/file/1020925/Temporary_Authorisation_HCP_Information_BNT162_-_24-09-2021.pdf

BNT162b2 (Pfizer/BioNTech) Preparation

Refer to the BNT162b2 Regulation 174 Information for UK healthcare professionals for vaccine preparation information:

https://assets.publishing.service.gov.uk/government/uploads/system/uploads/attachment_data/file/1020925/Temporary_Authorisation_HCP_Information_BNT162_-_24-09-2021.pdf

Comparator Arm-specific Sub-study Concomitant Medication Requirements or Restrictions

There are no specific requirements or restrictions regarding concomitant medications during the study beyond those specified in Section 9.5.

Comparator Arm-specific Sub-study Assessments

There are no additional tests or procedures specific to this sub-study.

There are no sub-study-specific statistical methods beyond those specified in Section 12.6.

APPENDIX 3: DOUBLE-BLIND EVALUATION OF UB-612 AS A HETEROLOGOUS BOOST AND CHADOX1-S SARS COV-2 VACCINE AS A HOMOLOGOUS BOOST. SUB-STUDY TO THE PLATFORM PROTOCOL: A PHASE 3 MULTI-CENTER PLATFORM RANDOMIZED, ACTIVE-CONTROLLED TRIAL TO COMPARE HOMOLOGOUS BOOST OF AUTHORIZED COVID-19 VACCINES AND HETEROLOGOUS BOOST WITH UB-612

This study will enroll approximately 190 subjects. It is estimated that up to 400 subjects may be screened for the study to ensure enrolment of 190 subjects.

Inclusion Criteria

In addition to the criteria specified in Section 8.1, subjects must meet the following criterion to be eligible for participation in this sub-study:

A3-.1 Fully vaccinated with 2 injections of ChAdOx1-S vaccine. The last dose of ChAdOx1-S vaccine must have been administered at least three (3) months from Day 1, taking into consideration the current local and national regulations. Documentation, such as the National Health Service (NHS) COVID Pass, United States Centers for Disease Control vaccine card, or equivalent documentation (e.g., medical records, vaccine passport; in accordance with local approved vaccination record documentation) will be required for proof of vaccination, vaccine manufacturer and vaccination dates.

Exclusion Criteria

There are no sub-study-specific exclusion criteria.

Comparative Vaccine Information: Astra Zeneca COVID-19 Vaccine (ChAdOx1-S)

Astra Zeneca COVID-19 vaccine (ChAdOx1-S) is Chimpanzee Adenovirus encoding the SARS-CoV-2 Spike glycoprotein (ChAdOx1-S). One dose (0.5 mL) contains approximately 5.0×10^{10} viral particles, and excipients (L-Histidine, L-Histidine hydrochloride monohydrate, Magnesium chloride hexahydrate, Polysorbate 80 (E 433), Ethanol, Sucrose, Sodium chloride, Disodium edetate dihydrate).

ChAdOx1-S Dosing Regimen

Subjects assigned to the ChAdOx1-S arm will receive ChAdOx1-S 0.5 ml dose (5.0×10^{10} viral particles) COVID-19 Vaccine IM on Day 1 in a double-blind fashion.

The study vaccine will be administered as an IM injection into the deltoid muscle, preferably of the nondominant arm, by a blinded administrator. To maintain the blind, the barrel of the syringe containing study vaccine (prepared by the unblinded study pharmacist) will be masked.

The study vaccines will be administered by an appropriately qualified and trained member of the study staff (e.g., physician, nurse, physician's assistant, nurse practitioner, pharmacist, or medical assistant) as allowed by local, state, and institutional guidance. The date, time, and vaccine injection site will be recorded in the source documentation and CRF.

All subjects will be observed by a trained site staff member for at least 30 (±5) minutes after being vaccinated. Appropriate medication and other supportive measures for management of

an acute hypersensitivity reaction will be available at the site in accordance with local guidelines for standard immunization practices.

ChAdOx1-S (Astra Zeneca) Formulation, Appearance, Packaging, and Labelling

ChAdOx1-S solution for injection is a clear to slightly opaque, colorless to slightly brown, sterile, particle free, preservative-free, solution for intramuscular injection.

Refer to the ChAdOx1-S product monograph for further information:

https://pdf.hres.ca/dpd_pm/00060048.PDF

One dose (0.5 ml) of AstraZeneca COVID-19 Vaccine contains:

- COVID-19 Vaccine (ChAdOx1-S* recombinant) 5 x 10^{10} viral particles (not less than 2.5 x 10^{8} infectious units)

  *Recombinant, replication-deficient chimpanzee adenovirus vector encoding the unmodified SARS-CoV-2 Spike (S) glycoprotein (GP) produced in genetically modified human embryonic kidney (HEK) 293 cells by recombinant DNA technology.

AstraZeneca COVID-19 Vaccine is packaged in (not all pack sizes may be available):

- 5 mL of solution in a 10-dose vial (clear type I glass) with stopper (elastomeric with aluminium overseal).
- 4 mL of solution in a 8-dose vial (clear type I glass) with stopper (elastomeric with aluminium overseal).

ChAdOx1-S (Astra Zeneca) Preparation

ChAdOx1-S (Astra Zeneca) COVID-19 Vaccine must not be reconstituted, mixed with other medicinal products, or diluted.

Comparator Arm-specific Sub-study Concomitant Medication Requirements or Restrictions

There are no specific requirements or restrictions regarding concomitant medications during the study beyond those specified in Section 9.5.

Comparator Arm-specific Sub-study Assessments

There are no additional tests or procedures specific to this sub-study.

Comparator Arm-specific Sub-study Statistical Methods

There are no sub-study-specific statistical methods beyond those specified in Section 12.6.

APPENDIX 4: OPEN-LABEL EVALUATION OF UB-612 AS A HETEROLOGOUS BOOST AND CHADOX1-S SARS COV-2 VACCINE AS A HOMOLOGOUS BOOST. SUB-STUDY TO THE PLATFORM PROTOCOL: A PHASE 3 MULTI-CENTER PLATFORM RANDOMIZED, ACTIVE-CONTROLLED TRIAL TO COMPARE HOMOLOGOUS BOOST OF AUTHORIZED COVID-19 VACCINES AND HETEROLOGOUS BOOST WITH UB-612

This is an open label sub-study. After subject randomized to the study, subject will be administered assigned vaccine as described in Section 9.3.

Inclusion Criteria

In addition to the criteria specified in Section 8.1, subjects must meet the following criterion to be eligible for participation in this sub-study:

A.4-1 Fully vaccinated with 2 injections of ChAdOx1-S vaccine. The last dose of ChAdOx1-S vaccine must have been administered at least three (3) months from Day 1, taking into consideration the current local and national regulations. Documentation, such as the National Health Service (NHS) COVID Pass, United States Centers for Disease Control vaccine card, or equivalent documentation (e.g., medical records, vaccine passport; in accordance with local approved vaccination record documentation) will be required for proof of vaccination, vaccine manufacturer and vaccination dates.

Exclusion Criteria

There are no sub-study-specific exclusion criteria.

Comparative Vaccine Information: Astra Zeneca COVID-19 Vaccine (ChAdOx1-S)

Astra Zeneca COVID-19 vaccine (ChAdOx1-S) is Chimpanzee Adenovirus encoding the SARS-CoV-2 Spike glycoprotein (ChAdOx1-S). One dose (0.5 mL) contains approximately 5.0×10^{10} viral particles, and excipients (L-Histidine, L-Histidine hydrochloride monohydrate, Magnesium chloride hexahydrate, Polysorbate 80 (E 433), Ethanol, Sucrose, Sodium chloride, Disodium edetate dihydrate).

ChAdOx1-S Dosing Regimen

Subjects assigned to the ChAdOx1-S arm will receive ChAdOx1-S 0.5 ml dose (5.0×10^{10} viral particles) COVID-19 Vaccine IM on Day 1 in an open-label fashion. Subjects assigned to the ChAdOx1-S arm are to receive ChAdOx1-S 0.3 ml dose (30 µg) COVID-19 mRNA Vaccine ChAdOx1-S IM on Day 1 in an open-label fashion. Subjects are to receive ChAdOx1-S vaccine at a local vaccination location (e.g., doctor's office, clinic, pharmacy) of their choice, provided the local vaccination location is operating by local and national regulations. After receiving the comparator vaccine, the subject is required to provide proof of vaccination to the study center to continue participation in the study. As a minimum, the date, time (if available), vaccine injection site, the name of vaccine, lot number, and expiration should be captured and shared with the study staff during the next visit.

The study vaccine will be administered as an IM injection into the deltoid muscle, preferably of the nondominant arm, accordingly to standard institutional practice.

Subjects will be observed post-vaccination according to standard institutional practice. Appropriate medication and other supportive measures for management of an acute hypersensitivity reaction will be available at the site in accordance with local guidelines for standard immunization practices.

ChAdOx1-S (Astra Zeneca) Formulation, Appearance, Packaging, and Labelling

ChAdOx1-S solution for injection is a clear to slightly opaque, colorless to slightly brown, sterile, particle free, preservative-free, solution for intramuscular injection.

Refer to the ChAdOx1-S product monograph for further information:

https://pdf.hres.ca/dpd_pm/00060048.PDF

One dose (0.5 ml) of AstraZeneca COVID-19 Vaccine contains:

- COVID-19 Vaccine (ChAdOx1-S* recombinant) 5 x 10^{10} viral particles (not less than 2.5 x 10^{8} infectious units)

  *Recombinant, replication-deficient chimpanzee adenovirus vector encoding the unmodified SARS-CoV-2 Spike (S) glycoprotein (GP) produced in genetically modified human embryonic kidney (HEK) 293 cells by recombinant DNA technology.

AstraZeneca COVID-19 Vaccine is packaged in (not all pack sizes may be available):

- 5 mL of solution in a 10-dose vial (clear type I glass) with stopper (elastomeric with aluminium overseal).
- 4 mL of solution in a 8-dose vial (clear type I glass) with stopper (elastomeric with aluminium overseal).

ChAdOx1-S (Astra Zeneca) Preparation

ChAdOx1-S (Astra Zeneca) COVID-19 Vaccine must not be reconstituted, mixed with other medicinal products, or diluted.

Comparator Arm-specific Sub-study Concomitant Medication Requirements or Restrictions

There are no specific requirements or restrictions regarding concomitant medications during the study beyond those specified in Section 9.5.

Comparator Arm-specific Sub-study Assessments

There are no additional tests or procedures specific to this sub-study.

Comparator Arm-specific Sub-study Statistical Methods

There are no sub-study-specific statistical methods beyond those specified in Section 12.6.

APPENDIX 5: DOUBLE-BLIND EVALUATION OF UB-612 AS A HETEROLOGOUS BOOST AND BIBP COVID-19 VACCINE AS A HOMOLOGOUS BOOST. AN APPENDIX TO THE PLATFORM PROTOCOL: A PHASE 3 MULTI-CENTER PLATFORM RANDOMIZED, ACTIVE-CONTROLLED TRIAL TO COMPARE HOMOLOGOUS BOOST OF AUTHORIZED COVID-19 VACCINES AND HETEROLOGOUS BOOST WITH UB-612

This study will enroll approximately 334 subjects who have completed 2-dose primary immunization with inactivated COVID-19 vaccines manufactured by SinoPharm or Sinovac. It is estimated that up to 600 subjects may be screened for the study to ensure enrolment of 334 subjects. The rationale for enrolling subjects who received primary immunization with any of two inactivated vaccines manufactured by Sinovac and SinoPharm is based on the following: 1) both vaccines use matching manufacturing platforms yielding comparable vaccine compositions, 2) clinical evidence supports similarity in both vaccines' immune responses and protection, 3) available clinical experience supports the interchangeability of both vaccines during the course of primary and booster immunization, 4) national authorities recommend using both vaccines interchangeably.

For the primary endpoint of GMT ratio, assuming a $\log_{10}$ standard deviation of 0.44 (based on 6 µg group [Kanokudom et al, 2021]), a sample size of 266 (133 per arm) will have 90% power to establish non-inferiority, with a non-inferiority margin of 1.5. A total of 334 subjects will be enrolled to account for an additional 25% of that sample size allocated for dropouts and major protocol deviations.

Inclusion Criteria

In addition to the criteria specified in Section 8.1, subjects must meet the following criterion to be eligible for participation in this sub-study:

A.5-.1 Fully vaccinated with 2-dose primary series of the following inactivated COVID-19 vaccines: (i) BIBP COVID-19 vaccine (SinoPharm), (ii) Coronavac COVID-19 vaccine (Sinovac), (iii) a mixed regimen with BIBP or Coronavac administered in any order. The last dose of inactivated COVID-19 vaccine must have been administered at least three (3) months from Day 1, taking into consideration the current local and national regulations. Documentation, such as the National Health Service (NHS) COVID Pass, United States Centers for Disease Control vaccine card, or equivalent documentation (e.g., medical records, vaccine passport; in accordance with local approved vaccination record documentation) will be required for proof of vaccination, vaccine manufacturer and vaccination dates.

Exclusion Criteria

In addition to the criteria specified in Section 8.2, subjects meeting the following exclusion criterion are excluded from participation in this sub-study:

A.5-1. Receipt of a booster (3rd dose) BIBP or other COVID-19 booster vaccination in addition to the primary vaccine series.

Comparative Vaccine Information: Sinopharm COVID-19 Vaccine (COVID-19 vaccine BIBP)

COVID-19 vaccine BIBP is a Vero cell-based, aluminium hydroxide-adjuvanted, β-propiolactone-inactivated vaccine based on the 19nCOV-CDC-TAN-HB02 strain (HB02 strain).

COVID-19 Vaccine BIBP Dosing Regimen

Subjects assigned to the BIBP (SinoPharm) will receive a 0.5 ml dose (4 µg/6.5U) of BIBP COVID-19 Vaccine IM on Day 1 in a double-blind fashion.

The study vaccine will be administered as an IM injection into the deltoid muscle, preferably of the nondominant arm, by a blinded administrator. To maintain the blind, the barrel of the syringe containing study vaccine (prepared by the unblinded study pharmacist) will be masked.

The study vaccines will be administered by an appropriately qualified and trained member of the study staff (e.g., physician, nurse, physician's assistant, nurse practitioner, pharmacist, or medical assistant) as allowed by local, state, and institutional guidance. At a minimum, the date, time, and vaccine injection site will be recorded in the source documentation and CRF.

All subjects will be observed by a trained site staff member for at least 30 (±5) minutes after being vaccinated. Appropriate medication and other supportive measures for management of an acute hypersensitivity reaction will be available at the site in accordance with local guidelines for standard immunization practices.

COVID-19 Vaccine BIBP Formulation, Appearance, Packaging, and Labelling

The final vaccine product in each 0.5 ml dose is composed of 6.5 U (4 µg) of inactivated SARS-CoV-2 antigens and aluminium hydroxide adjuvant in phosphate-buffered saline (PBS).

The dosage form of the vaccine is injectable liquid. The product is a semi-transparent suspension, slightly white in color (after shaking), in a single-dose vial or prefilled syringe. The vial (2 ml) is composed of middle borosilicate glass, with an aluminum foil cap and a film-coated rubber stopper. The prefilled syringe (1 ml) is composed of the needle cover, needle-bearing glass tube, plunger rubber cap and plunger stick.

Prefilled syringes are available in boxes of 300 as follows: one syringe and the product leaflet are packed in a carton, ten cartons are wrapped with polyethylene film, and 30 of these carton wraps are packed in an outer box. Single-dose vials are available in boxes of 400 as follows: one vial and the product leaflet are packed in a carton, ten cartons are wrapped with polyethylene film, and 40 of these carton wraps are packed in an outer box. A larger box, containing 600 vials, is also available, packaged as follows: three vials and the product leaflet are packed in a carton, ten cartons are wrapped with polyethylene film, and twenty of these carton wraps are packed into an outer box.

COVID-19 Vaccine BIBP Preparation

BIBP COVID-19 Vaccine is not to be reconstituted, mixed with other medicinal products, or diluted.

Comparator Arm-specific Sub-study Concomitant Medication Requirements or Restrictions

There are no specific requirements or restrictions regarding concomitant medications during the study beyond those specified in Section 9.5.

Comparator Arm-specific Sub-study Assessments

There are no additional tests or procedures specific to this sub-study.

Comparator Arm-specific Sub-study Statistical Methods

There are no sub-study-specific statistical methods beyond those specified in Section 12.6.

APPENDIX 6: LABORATORY ABNORMALITY GRADING SCALE

Parameter	Mild (Grade 1)	Moderate (Grade 2)	Severe (Grade 3)	Potentially Life Threatening (Grade 4)
Hemoglobin (g/dL)				
Female	11.0–12.0	9.5–10.9	8.0–9.4	<8.0
Male	12.5–13.5	10.5–12.4	8.5–10.4	<8.5
WBC increase (cells/mm^3)	10,800–15,000	15,001–20,000	20,001–25,000	>25,000
WBC decrease (cells/mm^3)	2500–3500	1500–2499	1000–1499	<1000
Lymphocytes decrease (cells/mm^3)	750–1000	500–749	250–499	<250
Neutrophils decrease (cells/mm^3)	1500–2000	1000–1499	500–999	<500
Eosinophils (cells/mm^3)	650–1500	1501–5000	>5000	Hypereosinophilic
Platelets decreased (cells/mm^3)	125,000–140,000	100,000–124,000	25,000–99,000	<25,000
BUN (mg/dL)	23–26	27–31	>31	Requires dialysis
Creatinine (mg/dL)	1.5–1.7	1.8–2.0	2.1–2.5	>2.5 or requires dialysis
ALP increase	1.1–2.0 × ULN	2.1–3.0 × ULN	3.1–10 × ULN	>10 × ULN
ALT or AST increase	1.1–2.5 × ULN	2.6–5.0 × ULN	5.1–10 × ULN	>10 × ULN
Bilirubin increase (when accompanied by any increase in ALT or AST)	1.1–1.25 × ULN	1.26–1.5 × ULN	1.51–1.75 × ULN	>1.75 × ULN
Bilirubin increase (when ALT or AST is normal)	1.1–1.5 × ULN	1.6–2.0 × ULN	2.0–3.0 × ULN	>3.0 × ULN
PT-increase by factor (prothrombin time)	1.0–1.10× ULN	1.11–1.20 × ULN	1.21-1.25 × ULN	>1.25 × ULN
PTT-increase by factor (partial thromboplastin time	1.0–1.2 × ULN	1.21–1.4 × ULN	1.41-1.5 × ULN	>1.5 × ULN
hsCRP (mg/L)	11-30	31-100	101-200	>200

Abbreviations: ALT = alanine aminotransferase; AST = aspartate aminotransferase; BUN = blood urea nitrogen; ULN = upper limit of normal; WBC = white blood cell; hsCRP – high sensitivity C-reactive protein.

APPENDIX 7: LIST OF POTENTIALLY IMMUNE-MEDIATED MEDICAL CONDITIONS

The following AESIs are to be followed during safety assessments:

Gastrointestinal disorders

- Acute pancreatitis
- Appendicitis
- Celiac disease
- Crohn’s disease
- Ulcerative colitis
- Ulcerative proctitis

Liver disorders

- Autoimmune cholangitis
- Autoimmune hepatitis
- Primary biliary cirrhosis
- Primary sclerosing cholangitis

Metabolic diseases

- Addison’s disease
- Autoimmune thyroiditis (including Hashimoto thyroiditis)
- Diabetes mellitus type I
- Subacute thyroiditis
- Grave's or Basedow’s disease

Musculoskeletal disorders

- Acute aseptic arthritis
- Antisynthetase syndrome
- Dermatomyositis
- Juvenile chronic arthritis (including Still’s disease)
- Mixed connective tissue disorder
- Multisystem inflammatory syndrome (children & adults)
- Polymyalgia rheumatic
- Polymyositis
- Psoriatic arthropathy
- Relapsing polychondritis
- Rhabdomyolysis
- Rheumatoid arthritis

- Scleroderma, including diffuse systemic form and CREST syndrome
- Spondyloarthritis, including ankylosing spondylitis, reactive arthritis (Reiter's Syndrome) and undifferentiated spondyloarthritis
- Systemic lupus erythematosus
- Systemic sclerosis

Neuroinflammatory disorders

- Acute disseminated encephalomyelitis, including site specific variants (e.g., non-infectious encephalitis, encephalomyelitis, myelitis, radiculomyelitis)
- Aseptic meningitis
- Cranial nerve disorders, including paralyses/paresis (e.g., Bell's palsy)
- Generalized convulsion
- Guillain-Barré syndrome, including Miller Fisher syndrome and other variants
- Immune-mediated peripheral neuropathies and plexopathies, including chronic inflammatory demyelinating polyneuropathy, multifocal motor neuropathy and polyneuropathies associated with monoclonal gammopathy
- Multiple sclerosis
- Narcolepsy
- Optic neuritis
- Transverse myelitis
- Myasthenia gravis, including Eaton-Lambert syndrome

Skin disorders

- Alopecia areata
- Autoimmune bullous skin diseases, including pemphigus, pemphigoid and dermatitis herpetiformis
- Cutaneous lupus erythematosus
- Erythema multiforme
- Erythema nodosum
- Morphoea
- Lichen planus
- Psoriasis
- Rosacea
- Single Organ Cutaneous Vasculitis
- Sweet's syndrome
- Vitiligo

Cardiac

- Autoimmune myocarditis/cardiomyopathy
- Acute cardiovascular injury (includes: myocarditis/pericarditis, microangiopathy, heart failure, stress cardiomyopathy, coronary artery disease arrhythmia)

Circulatory Disorders

- Deep Vein Thrombosis (DVT)
- Hemorrhagic Stroke
- Large vessels vasculitis including: giant cell arteritis such as Takayasu's arteritis and temporal arteritis
- Medium sized and/or small vessels vasculitis including: polyarteritis nodosa, Kawasaki's disease, microscopic polyangiitis, Wegener's granulomatosis, Churg–Strauss syndrome (allergic granulomatous angiitis), Buerger's disease thromboangiitis obliterans, necrotizing vasculitis and anti-neutrophil cytoplasmic antibody (ANCA) positive vasculitis (type unspecified), Henoch- Schonlein purpura, Behcet's syndrome, leukocytoclastic vasculitis
- Non-hemorrhagic Stroke
- Pulmonary Embolism (PE)
- Raynaud's phenomenon
- Thrombosis with thrombocytopenia syndrome

Blood

- Antiphospholipid syndrome
- Autoimmune hemolytic anemia
- Autoimmune thrombocytopenia
- Coagulation disorder (includes: thrombotic disorders, bleeding disorders, disseminated intravascular coagulation)

Others

- Acute kidney injury
- Acute respiratory distress syndrome
- Anaphylaxis
- Anosmia, ageusia Autoimmune glomerulonephritis (including IgA nephropathy, glomerulonephritis rapidly progressive, membranous glomerulonephritis, membranoproliferative glomerulonephritis, and mesangioproliferative glomerulonephritis)
- Goodpasture syndrome
- Idiopathic pulmonary fibrosis
- Pernicious anemia

- Sarcoidosis
- Sjögren's syndrome
- Stevens-Johnson syndrome
- Uveitis
- Vaccine associated enhanced disease

APPENDIX 8: WHO CLINICAL PROGRESSION SCALE

Patient State	Description	Score
Uninfected	Uninfected; no viral RNA detected	0
Ambulatory; mild disease	Asymptomatic; viral RNA detected	1
	Symptomatic; independent	2
	Symptomatic; assistance needed	3
Hospitalized; moderate disease	Hospitalized; no oxygen therapy[a]	4
	Hospitalized; oxygen by mask or nasal prongs	5
Hospitalized; severe disease	Hospitalized; oxygen by NIV or high flow	6
	Intubation and mechanical ventilation, $pO_2/FiO_2 \geq 150$ or $SpO_2/FiO_2 \geq 200$	7
	Mechanical ventilation, $pO_2/FiO_2 < 150$ or $SpO_2/FiO_2 < 200$ or vasopressors	8
	Mechanical ventilation, $pO_2/FiO_2 < 150$ and vasopressors, dialysis, or ECMO	9
Dead	Dead	10

Abbreviations: ECMO = extracorporeal membrane oxygenation; FiO_2 = fraction of inspired oxygen; NIV = non-invasive ventilation; pO_2 = partial pressure of oxygen; SpO_2 = oxygen saturation

[a] If hospitalized for isolation only, record status as for ambulatory subject.

Source: WHO Working Group on the Clinical Characterisation and Management of COVID-19 infection. A minimal common outcome measure set for COVID-19 clinical research. Lancet Infect Dis. 2020 Aug;20(8):e192 e197.
