# Supplementary material for: Safety and immunogenicity of UB-612 heterologous booster in adults primed with mRNA, adenovirus, or inactivated COVID-19 vaccines: a randomized, active-controlled, Phase 3 trial
Source: eClinicalMedicine. 2025 Jul 21;86:103349. doi: 10.1016/j.eclinm.2025.103349 (PMC12301762; doi:10.1016/j.eclinm.2025.103349)
Supplement: Appendix 2 [file mmc2.pdf]

Supplemental table 1. Baseline characteristics of intent-to-treat population (cont'ed).

	**BBIBP-CorV substudy**		**ChAdOx1-S substudy**		**BNT162b2 substudy**	
	BBIBP-CorV (n=168)	UB-612 (n=172)	ChAdOx1-S (n=98)	UB-612 (n=96)	BNT162b2 (n=205)	UB-612 (n=205)
Height (cm)						
Mean (SD)	158.2 (8.2)	158.1 (8.2)	157.0 (8.9)	155.8 (7.3)	164.8 (10.7)	164.3 (9.6)
Range (min, max)	140.0, 179.5	131.0, 177.0	136.0, 176.0	141.0, 176.0	137.6, 191.6	140.0, 200.0
Weight (kg)						
Mean (SD)	58.8 (11.7)	59.9 (12.8)	57.1 (11.7)	59.5 (13.5)	69.9 (18.4)	70.6 (16.8)
Range (min, max)	39.0, 100.0	34.0, 109.0	33.0, 106.0	36.0, 103.0	35.0, 129.0	35.0, 135.0
BMI (kg/m2)						
Mean (SD)	23.5 (4.2)	23.9 (4.5)	23.2 (4.4)	24.6 (5.5)	25.5 (5.3)	26.0 (5.3)
Range (min, max)	16.0, 40.4	15.5, 38.4	15.3, 41.4	15.8, 46.6	16.2, 42.6	14.6, 46.0
Comorbidities, n (%)						
Any	44 (26.2)	47 (27.3)	38 (38.8)	28 (29.2)	76 (37.1)	83 (40.5)
Chronic lung disease	1 (0.6)	0	1 (1.0)	0	7 (3.4)	5 (2.4)
Diabetes Mellitus	1 (0.6)	1 (0.6)	0	0	0	0
Cardiovascular diseases	7 (4.2)	7 (4.1)	1 (1.0)	0	6 (2.9)	12 (5.9)
Chronic liver disease	0	0	0	0	1 (0.5)	0
Obesity	13 (7.1)	17 (9.9)	9 (9.2)	12 (12.5)	38 (18.5)	47 (22.9)
Smoking	28 (16.7)	27 (15.7)	29 (29.6)	19 (19.8)	40 (19.5)	30 (14.6)
Other chronic diseases	0	0	0	1 (1.0)	0	2 (1.0)
Baseline N-protein seropositivity by confirmatory antibody ELISA test, n (%)						
>=200	159 (94.6)	162 (94.2)	-	-	-	-
<200	9 (5.4)	8 (4.7)	-	-	-	--
Missing	0	2 (1.2)	-	-		-
Baseline N-protein seropositivity by confirmatory antibody COBAS test, n (%)						
Reactive	-	-	90 (91.8)	90 (91.8)	194 (94.6)	190 (92.7)
No reactive	-	-	5 (5.1)	6 (6.3)	10 (4.9)	15 (7.3)
Missing	-	-	3 (3.1)	4 (4.2)	1 (0.5)	0
Fc-Mediated Functional Antibody (ADCP phagocytic score)						
	n=18	n=13	n=10	n=10	n=25	n=24
Geometric mean (geometric SD)	41.0 (1.4)	37.8 (1.3)	46.5 (1.4)	41.7 (1.2)	81.9 (1.5)	97.3 (1.6)
Range (min, max)	30.6, 105.9	24.4, 69.9	24.0, 74.6	33.5, 55.4	44.2, 170.3	39.0, 203.0

The confirmatory N-protein antibody test for subjects primed with BBIBP-CorV, a COVID-19 vaccine containing N protein, was based on semi-quantitative ELISA with the LLOD of 200, and ULOD of 102,400. Sera of subjects primed with ChAdOx1-S or BNT162b2 were tested in Cobas e411 (Roche) calibrated to detect N-protein reactive serum samples with cut-off index ≥1.0 while non-reactive were <1.0.

Supplemental table 2A. UB-612-305 BBIBP-CorV substudy Wuhan neutralizing antibodies

Neutralizing Antibody Titer: GMT

		Baseline	Day 15	Day 29	Month 6
	UB-612				
n		167	167	167	163
Geometric mean (geometric SD)		478.63 (3.701)	2341.44 (3.708)	2781.57 (2.906)	770.04 (2.827)
Median		452.50	1810.20	2560.00	905.10
Q1, Q3		160.00, 1280.00	905.10, 7240.80	1280.00, 5120.00	452.50, 1280.00
Min, Max		80.0, 20480.0	80.0, 40960.0	160.0, 40960.0	80.0, 40960.0
	BBIBP-CorV				
n		166	166	166	160
Geometric mean (geometric SD)		430.43 (3.584)	427.75 (3.248)	454.44 (3.651)	389.72 (2.977)
Median		386.25	386.25	452.50	452.50
Q1, Q3		160.00, 1280.00	160.00, 905.10	160.00, 905.10	160.00, 905.10
Min, Max		80.0, 7240.8	80.0, 10240.0	80.0, 14481.5	80.0, 7240.8
	UB-612 vs. BBIBP-CorV				
GMR			5.20	5.77	1.90
95% CI			4.088, 6.622	4.624, 7.202	1.540, 2.349

Seroconversion Rate

		Baseline	Day 15	Day 29	Month 6
	UB-612				
n			167	167	163
Rate, n(%)			100 (59.9)	108 (64.7)	44 (27.0)
95% CI			52.45, 67.31	57.42, 71.92	20.18, 33.81
	BBIBP-CorV				
n			166	166	160
Rate, n(%)			20 (12.0)	13 (7.8)	24 (15.0)
95% CI			7.10, 17.00	3.74, 11.92	9.47, 20.53
	UB-612 vs. BBIBP-CorV				
Rate difference			47.8	56.8	12.0
95% CI			38.90, 56.76	48.52, 65.16	3.22, 20.77

Increase in Neutralizing Antibody Titer: GMFI

		Baseline	Day 15	Day 29	Month 6
	UB-612				
n			167	167	163
Geometric mean (geometric SD)			4.892 (4.4915)	5.812 (4.0167)	1.600 (3.7427)
Median			5.656	5.657	1.414
Q1, Q3			2.000, 11.315	2.000, 16.000	0.707, 4.000
Min, Max			0.02, 181.02	0.06, 181.02	0.04, 45.26
	BBIBP-CorV				
n			166	166	160
Geometric mean (geometric SD)			0.994 (3.0845)	1.056 (2.9543)	0.905 (3.4318)
Median			1.000	1.000	1.000
Q1, Q3			0.500, 1.414	0.707, 2.000	0.354, 2.000
Min, Max			0.04, 45.26	0.06, 181.02	0.06, 22.62

Supplemental table 2B. UB-612-305 BBIBP-CorV substudy Omicron BA.5 neutralizing antibodies

Neutralizing Antibody Titer: GMT

		Baseline	Day 15	Day 29	Month 6
	UB-612				
n		167	167	167	163
Geometric mean (geometric SD)		69.48 (4.321)	510.44 (4.117)	573.34 (3.848)	284.69 (3.357)
Median		56.60	640.00	640.00	320.00
Q1, Q3		20.00, 160.00	160.00, 1280.00	226.30, 1810.20	160.00, 640.00
Min, Max		10.0, 3620.4	10.0, 10240.0	20.0, 7240.8	10.0, 10240.0
	BBIBP-CorV				
n		166	166	166	160
Geometric mean (geometric SD)		64.53 (3.819)	95.14 (4.215)	92.60 (4.125)	181.42 (3.552)
Median		56.60	80.00	56.60	160.00
Q1, Q3		28.30, 160.00	40.00, 226.30	28.30, 226.30	80.00, 452.50
Min, Max		10.0, 1810.2	10.0, 7240.8	10.0, 5120.0	10.0, 7240.8
	UB-612 vs. BBIBP-CorV				
GMR			5.12	5.93	1.52
95% CI			3.892, 6.741	4.596, 7.645	1.185, 1.959

Seroconversion Rate

		Baseline	Day 15	Day 29	Month 6
	UB-612				
n			167	167	163
Rate, n(%)			110 (65.9)	117 (70.1)	82 (50.3)
95% CI			58.68, 73.06	63.11, 77.01	42.63, 57.98
	BBIBP-CorV				
n			166	166	160
Rate, n(%)			29 (17.5)	20 (12.0)	70 (43.8)
95% CI			11.69, 23.25	7.10, 17.00	36.06, 51.44
	UB-612 vs. BBIBP-CorV				
Rate difference			48.4	58.0	6.6
95% CI			39.17, 57.62	49.48, 66.54	-4.31, 17.42

Increase in Neutralizing Antibody Titer: GMFI

		Baseline	Day 15	Day 29	Month 6
	UB-612				
n			167	167	163
Geometric mean (geometric SD)			7.347 (5.2737)	8.252 (4.4926)	4.000 (4.3355)
Median			7.995	8.000	4.000
Q1, Q3			2.828, 22.628	2.828, 22.630	1.000, 11.307
Min, Max			0.00, 256.00	0.04, 512.00	0.18, 180.92
	BBIBP-CorV				
n			166	166	160
Geometric mean (geometric SD)			1.474 (3.5525)	1.435 (3.3628)	2.810 (4.8527)
Median			1.000	1.414	2.827
Q1, Q3			0.707, 2.000	0.707, 2.001	0.854, 9.654
Min, Max			0.03, 64.00	0.04, 181.02	0.09, 128.00

Supplemental table 2C. UB-612-305 BBIBP-CorV substudy RBD-binding IgG antibodies

Neutralizing Antibody Titer: GMT

		Baseline	Day 15	Day 29	Month 6
	UB-612				
n		167	167	167	163
Geometric mean (geometric SD)		1680.71 (3.220)	18828.59 (3.815)	13662.78 (2.586)	4364.14 (2.475)
Median		1510.50	20550.60	12327.20	4098.20
Q1, Q3		400.00, 3259.20	8850.60, 46345.60	7824.10, 25362.20	2663.10, 6399.50
Min, Max		400.0, 46225.3	400.0, 332396.2	400.0, 154793.1	400.0, 162585.3
	BBIBP-CorV				
n		166	166	166	160
Geometric mean (geometric SD)		1642.21 (3.138)	2412.62 (3.163)	1596.95 (3.107)	1891.46 (3.022)
Median		1511.20	2417.60	1494.75	1713.80
Q1, Q3		400.00, 3912.30	1144.40, 5367.50	400.00, 3548.80	1098.10, 3094.35
Min, Max		400.0, 42213.0	400.0, 50916.0	400.0, 54409.4	400.0, 94681.4
	UB-612 vs. BBIBP-CorV				
GMR			7.69	8.42	2.31
95% CI			6.004, 9.857	6.857, 10.327	1.875, 2.842

Seroconversion Rate

		Baseline	Day 15	Day 29	Month 6
	UB-612				
n			167	167	163
Rate, n(%)			129 (77.2)	110 (65.9)	58 (35.6)
95% CI			70.89, 83.60	58.68, 73.06	28.23, 42.93
	BBIBP-CorV				
n			166	166	160
Rate, n(%)			24 (14.5)	13 (7.8)	23 (14.4)
95% CI			9.11, 19.81	3.74, 11.92	8.94, 19.81
	UB-612 vs. BBIBP-CorV				
Rate difference			62.8	58.0	21.2
95% CI			54.48, 71.10	49.77, 66.31	12.07, 30.35

Increase in Neutralizing Antibody Titer: GMFI

		Baseline	Day 15	Day 29	Month 6
	UB-612				
n			167	167	163
Geometric mean (geometric SD)			11.203 (4.7889)	8.129 (3.8534)	2.554 (3.3449)
Median			11.474	8.654	2.692
Q1, Q3			4.419, 29.082	2.807, 22.201	1.158, 5.768
Min, Max			0.10, 452.04	0.08, 229.46	0.17, 65.81
	BBIBP-CorV				
n			166	166	160
Geometric mean (geometric SD)			1.469 (2.9151)	0.972 (2.8384)	1.139 (3.5937)
Median			1.109	1.000	1.000
Q1, Q3			0.836, 2.477	0.533, 1.254	0.444, 2.777
Min, Max			0.03, 61.78	0.05, 136.02	0.06, 40.18

Supplemental table 2E. UB-612-305 ChAdOx1-S substudy Wuhan neutralizing antibodies

Neutralizing Antibody Titer: GMT

		Baseline	Day 15	Day 29	Month 6
	UB-612				
n		91	91	91	87
Geometric mean (geometric SD)		604.46 (3.863)	2473.74 (2.872)	2215.07 (2.713)	658.09 (2.871)
Median		452.50	2560.00	2560.00	640.00
Q1, Q3		226.30, 1280.00	1280.00, 5120.00	1280.00, 5120.00	320.00, 1280.00
Min, Max		80.0, 20480.0	226.3, 40960.0	226.3, 28963.1	80.0, 10240.0
	ChAdOx1-S				
n		93	93	93	92
Geometric mean (geometric SD)		632.88 (3.432)	993.47 (2.772)	1153.17 (2.831)	526.15 (2.487)
Median		640.00	1280.00	1280.00	640.00
Q1, Q3		320.00, 1810.20	640.00, 1810.20	640.00, 2560.00	320.00, 905.10
Min, Max		80.0, 14481.5	80.0, 10240.0	80.0, 10240.0	80.0, 7240.8
	UB-612 vs. ChAdOx1-S				
GMR			2.53	1.92	1.24
95% CI			1.895, 3.374	1.440, 2.562	0.936, 1.656

Seroconversion Rate

		Baseline	Day 15	Day 29	Month 6
	UB-612				
n			91	91	87
Rate, n(%)			50 (54.9)	45 (49.5)	22 (25.3)
95% CI			44.72, 65.17	39.18, 59.72	16.15, 34.42
	ChAdOx1-S				
n			93	93	92
Rate, n(%)			19 (20.4)	24 (25.8)	10 (10.9)
95% CI			12.24, 28.62	16.91, 34.70	4.51, 17.23
	UB-612 vs. ChAdOx1-S				
Rate difference			34.5	23.6	14.4
95% CI			21.41, 47.62	10.06, 37.23	3.29, 25.55

Increase in Neutralizing Antibody Titer: GMFI

		Baseline	Day 15	Day 29	Month 6
	UB-612				
n			91	91	87
Geometric mean (geometric SD)			4.092 (5.1223)	3.665 (4.6932)	1.041 (4.8839)
Median			4.000	4.000	2.000
Q1, Q3			1.414, 11.315	1.414, 11.314	0.354, 4.000
Min, Max			0.02, 90.51	0.12, 90.51	0.03, 22.63
	ChAdOx1-S				
n			93	93	92
Geometric mean (geometric SD)			1.570 (3.3357)	1.822 (3.7170)	0.844 (3.5702)
Median			1.414	2.000	0.707
Q1, Q3			0.707, 2.829	0.707, 4.000	0.500, 2.000
Min, Max			0.06, 90.51	0.09, 90.51	0.02, 16.00

Supplemental table 2E. UB-612-305 ChAdOx1-S substudy Omicron BA.5 neutralizing antibodies

Neutralizing Antibody Titer: GMT

		Baseline	Day 15	Day 29	Month 6
	UB-612				
n		91	91	91	87
Geometric mean (geometric SD)		90.72 (3.963)	558.01 (3.174)	499.66 (3.531)	289.67 (2.908)
Median		80.00	640.00	452.50	320.00
Q1, Q3		28.30, 226.30	320.00, 1280.00	226.30, 1280.00	160.00, 640.00
Min, Max		10.0, 2560.0	28.3, 10240.0	28.3, 7240.8	10.0, 2560.0
	ChAdOx1-S				
n		93	93	93	92
Geometric mean (geometric SD)		96.76 (3.837)	187.11 (3.165)	178.27 (3.620)	203.62 (3.132)
Median		80.00	226.30	160.00	226.30
Q1, Q3		28.30, 320.00	80.00, 452.50	56.60, 452.50	113.10, 452.50
Min, Max		10.0, 2560.0	10.0, 3620.4	10.0, 2560.0	10.0, 3620.4
	UB-612 vs. ChAdOx1-S				
GMR			3.05	2.85	1.40
95% CI			2.224, 4.169	2.002, 4.054	1.020, 1.910

Seroconversion Rate

		Baseline	Day 15	Day 29	Month 6
	UB-612				
n			91	91	87
Rate, n(%)			55 (60.4)	53 (58.2)	40 (46.0)
95% CI			50.39, 70.49	48.11, 68.37	35.50, 56.45
	ChAdOx1-S				
n			93	93	92
Rate, n(%)			20 (21.5)	27 (29.0)	34 (37.0)
95% CI			13.16, 29.86	19.81, 38.26	27.09, 46.82
	UB-612 vs. ChAdOx1-S				
Rate difference			38.9	29.2	9.0
95% CI			25.87, 52.00	15.51, 42.91	-5.37, 23.41

Increase in Neutralizing Antibody Titer: GMFI

		Baseline	Day 15	Day 29	Month 6
	UB-612				
n			91	91	87
Geometric mean (geometric SD)			6.151 (5.1031)	5.507 (6.3179)	3.087 (4.9169)
Median			7.995	5.657	3.998
Q1, Q3			2.000, 22.615	1.000, 16.005	1.000, 11.307
Min, Max			0.18, 256.00	0.12, 256.00	0.04, 128.00
	ChAdOx1-S				
n			93	93	92
Geometric mean (geometric SD)			1.934 (3.4453)	1.842 (3.8963)	2.148 (4.1434)
Median			1.998	2.000	2.827
Q1, Q3			1.000, 3.996	0.707, 4.000	0.707, 5.657
Min, Max			0.09, 256.00	0.06, 181.02	0.06, 45.23

Supplemental 2F. UB-612-305 ChAdOx1-S substudy RBD-binding IgG antibodies

Neutralizing Antibody Titer: GMT

		Baseline	Day 15	Day 29	Month 6
	UB-612				
n		91	91	91	87
Geometric mean (geometric SD)		2619.58 (3.110)	26225.63 (2.854)	14832.33 (2.526)	3609.53 (2.311)
Median		2711.00	25506.90	17754.80	3048.00
Q1, Q3		1325.80, 5599.00	11857.70, 51026.60	7474.20, 25987.60	2180.80, 6207.10
Min, Max		400.0, 39766.7	1543.6, 402777.3	973.9, 99322.0	400.0, 25100.2
	ChAdOx1-S				
n		93	93	93	92
Geometric mean (geometric SD)		2665.41 (2.778)	6288.15 (2.324)	4178.86 (2.544)	2209.23 (2.466)
Median		2674.10	5681.10	3638.00	2153.80
Q1, Q3		1423.90, 5463.30	3223.60, 10086.20	2610.50, 6293.10	1412.15, 3506.95
Min, Max		400.0, 21996.0	1346.0, 130356.1	400.0, 79540.9	400.0, 31256.5
	UB-612 vs. ChAdOx1-S				
GMR			4.18	3.58	1.63
95% CI			3.175, 5.497	2.732, 4.692	1.259, 2.113

Seroconversion Rate

		Baseline	Day 15	Day 29	Month 6
	UB-612				
n			91	91	87
Rate, n(%)			66 (72.5)	55 (60.4)	16 (18.4)
95% CI			63.36, 81.70	50.39, 70.49	10.25, 26.53
	ChAdOx1-S				
n			93	93	92
Rate, n(%)			17 (18.3)	11 (11.8)	8 (8.7)
95% CI			10.42, 26.13	5.26, 18.39	2.94, 14.45
	UB-612 vs. ChAdOx1-S				
Rate difference			54.2	48.6	9.7
95% CI			42.17, 66.32	36.61, 60.61	-0.28, 19.67

Increase in Neutralizing Antibody Titer: GMFI

		Baseline	Day 15	Day 29	Month 6
	UB-612				
n			91	91	87
Geometric mean (geometric SD)			10.011 (4.7253)	5.662 (4.4288)	1.398 (3.7620)
Median			11.091	6.549	1.494
Q1, Q3			2.720, 30.341	1.987, 16.310	0.569, 3.643
Min, Max			0.41, 269.87	0.15, 119.92	0.05, 27.49
	ChAdOx1-S				
n			93	93	92
Geometric mean (geometric SD)			2.359 (3.0275)	1.568 (3.3220)	0.840 (3.6549)
Median			1.954	1.397	0.746
Q1, Q3			1.195, 3.371	0.832, 2.517	0.420, 1.640
Min, Max			0.15, 325.89	0.12, 198.85	0.02, 78.14

Supplemental table 2H. UB-612-305 BNT162b2 substudy Wuhan neutralizing antibodies

Neutralizing Antibody Titer: GMT

		Baseline	Day 15	Day 29	Month 6	Month 12
	UB-612					
n		201	200	201	191	110
Geometric mean (geometric SD)		1426.87 (3.088)	6391.46 (2.751)	6971.25 (2.595)	1585.61 (2.390)	2411.24 (2.467)
Median		1810.20	7240.80	7240.80	1280.00	1810.20
Q1, Q3		640.00, 3620.40	3090.20, 14481.50	3620.40, 14481.50	905.10, 2560.00	1280.00, 5120.00
Min, Max		80.0, 20480.0	640.0, 81920.0	452.5, 81920.0	160.0, 20480.0	320.0, 57926.2
	BNT162b2					
n		198	197	198	194	118
Geometric mean (geometric SD)		1389.74 (2.987)	7114.51 (2.172)	6576.21 (2.246)	1424.81 (2.478)	1783.80 (2.541)
Median		1280.00	7240.80	7240.80	1810.20	1810.20
Q1, Q3		640.00, 3620.40	3620.40, 14481.50	3620.40, 10240.00	905.10, 2560.00	905.10, 3620.40
Min, Max		80.0, 57926.2	905.1, 40960.0	640.0, 40960.0	80.0, 28963.1	160.0, 14481.5
	UB-612 vs. BNT162b2					
GMR			0.89	1.04	1.09	1.31
95% CI			0.765, 1.035	0.893, 1.210	0.922, 1.290	1.051, 1.633

Seroconversion Rate

		Baseline	Day 15	Day 29	Month 6	Month 12
	UB-612					
n			200	201	191	110
Rate, n(%)			114 (57.0)	127 (63.2)	22 (11.5)	15 (13.6)
95% CI			50.14, 63.86	56.52, 69.85	6.99, 16.05	7.22, 20.05
	BNT162b2					
n			197	198	194	118
Rate, n(%)			125 (63.5)	124 (62.6)	26 (13.4)	9 (7.6)
95% CI			56.73, 70.18	55.89, 69.36	8.61, 18.20	2.84, 12.42
	UB-612 vs. BNT162b2					
Rate difference			-6.5	0.6	-1.9	6.0
95% CI			-16.06, 3.16	-8.92, 10.04	-8.48, 4.71	-1.99, 14.01

Increase in Neutralizing Antibody Titer: GMFI

		Baseline	Day 15	Day 29	Month 6	Month 12
	UB-612					
n			200	201	191	110
Geometric mean (geometric SD)			4.485 (2.9412)	4.886 (2.9241)	1.093 (3.3346)	1.271 (3.2725)
Median			4.000	5.657	1.000	1.414
Q1, Q3			2.828, 8.000	2.000, 11.312	0.500, 2.828	0.500, 2.828
Min, Max			0.13, 90.51	0.18, 64.00	0.02, 128.00	0.06, 90.51
	BNT162b2					
n			197	198	194	118
Geometric mean (geometric SD)			5.126 (2.8402)	4.732 (2.6481)	1.013 (2.9384)	1.024 (2.4574)
Median			5.657	4.000	1.000	1.000
Q1, Q3			2.828, 11.314	2.828, 8.000	0.500, 2.000	0.500, 2.000
Min, Max			0.13, 90.51	0.35, 90.51	0.06, 16.00	0.12, 16.00

Supplemental table 2I. UB-612-305 BNT162b2 substudy Omicron BA.5 neutralizing antibodies

Neutralizing Antibody Titer: GMT

		Baseline	Day 15	Day 29	Month 6	Month 12
	UB-612					
n		201	200	201	191	110
Geometric mean (geometric SD)		157.54 (3.541)	1076.35 (3.161)	795.29 (2.869)	434.84 (2.787)	449.70 (3.261)
Median		160.00	905.10	905.10	452.50	452.50
Q1, Q3		80.00, 452.50	452.50, 2560.00	452.50, 1810.20	226.30, 905.10	226.30, 905.10
Min, Max		10.0, 5120.0	56.6, 10240.0	56.6, 10240.0	20.0, 7240.8	10.0, 5120.0
	BNT162b2					
n		198	197	198	194	118
Geometric mean (geometric SD)		178.97 (3.497)	1051.07 (2.390)	743.96 (2.391)	393.69 (2.703)	430.51 (3.331)
Median		160.00	905.10	905.10	452.50	452.50
Q1, Q3		80.00, 452.50	640.00, 1810.20	452.50, 1280.00	226.30, 640.00	226.30, 905.10
Min, Max		10.0, 5120.0	113.1, 10240.0	80.0, 7240.8	20.0, 5120.0	10.0, 10240.0
	UB-612 vs. BNT162b2					
GMR			1.06	1.11	1.16	1.14
95% CI			0.891, 1.263	0.940, 1.310	0.956, 1.396	0.847, 1.523

Seroconversion Rate

		Baseline	Day 15	Day 29	Month 6	Month 12
	UB-612					
n			200	201	191	110
Rate, n(%)			136 (68.0)	117 (58.2)	81 (42.4)	42 (38.2)
95% CI			61.54, 74.46	51.39, 65.03	35.40, 49.42	29.10, 47.26
	BNT162b2					
n			197	198	194	118
Rate, n(%)			128 (65.0)	107 (54.0)	64 (33.0)	31 (26.3)
95% CI			58.31, 71.64	47.10, 60.98	26.37, 39.61	18.33, 34.21
	UB-612 vs. BNT162b2					
Rate difference			3.0	4.2	9.4	11.9
95% CI			-6.26, 12.31	-5.56, 13.90	-0.22, 19.06	-0.15, 23.97

Increase in Neutralizing Antibody Titer: GMFI

		Baseline	Day 15	Day 29	Month 6	Month 12
	UB-612					
n			200	201	191	110
Geometric mean (geometric SD)			6.821 (3.3567)	5.048 (3.5075)	2.783 (3.6293)	2.319 (4.2678)
Median			5.658	4.000	2.829	2.828
Q1, Q3			2.829, 16.000	2.000, 11.314	1.000, 5.659	1.000, 5.657
Min, Max			0.25, 256.00	0.18, 362.04	0.13, 128.00	0.06, 181.02
	BNT162b2					
n			197	198	194	118
Geometric mean (geometric SD)			5.901 (3.2280)	4.157 (3.0731)	2.140 (3.6752)	1.794 (3.2417)
Median			5.657	4.000	2.000	1.415
Q1, Q3			2.828, 15.989	2.000, 8.000	0.708, 5.654	1.000, 4.000
Min, Max			0.25, 512.00	0.35, 362.04	0.13, 127.93	0.01, 32.00

Supplemental table 2J. UB-612-305 BNT162b2 substudy RBD-Binding IgG antibodies

Neutralizing Antibody Titer: GMT

		Baseline	Day 15	Day 29	Month 6	Month 12
	UB-612					
n		201	200	201	191	110
Geometric mean (geometric SD)		4982.12 (2.602)	46036.99 (2.650)	38301.19 (2.667)	11454.13 (2.490)	12242.92 (2.239)
Median		4787.90	44982.70	37141.60	11188.50	12098.90
Q1, Q3		2873.10, 9123.20	23024.90, 89076.05	20063.20, 79255.00	5837.10, 22624.00	6343.10, 22199.60
Min, Max		400.0, 79368.3	3918.4, 409600.0	3074.6, 379184.7	1183.2, 403916.8	1666.5, 101295.0
	BNT162b2					
n		198	197	198	194	118
Geometric mean (geometric SD)		5281.97 (2.538)	31415.56 (2.199)	22369.22 (2.207)	6975.99 (2.323)	7652.97 (2.275)
Median		5537.50	30549.80	21758.90	6519.05	7616.50
Q1, Q3		2831.90, 11221.40	17933.10, 54357.70	12422.40, 38747.60	3935.90, 12162.30	4933.60, 12357.60
Min, Max		400.0, 56902.4	1505.5, 223772.5	3314.1, 398044.1	400.0, 82483.7	879.1, 90694.0
	UB-612 vs. BNT162b2					
GMR			1.47	1.72	1.70	1.64
95% CI			1.257, 1.717	1.466, 2.022	1.454, 1.993	1.351, 1.990

Seroconversion Rate

		Baseline	Day 15	Day 29	Month 6	Month 12
	UB-612					
n			200	201	191	110
Rate, n(%)			156 (78.0)	147 (73.1)	51 (26.7)	22 (20.0)
95% CI			72.26, 83.74	67.01, 79.26	20.43, 32.98	12.52, 27.48
	BNT162b2					
n			197	198	194	118
Rate, n(%)			127 (64.5)	102 (51.5)	17 (8.8)	4 (3.4)
95% CI			57.78, 71.15	44.55, 58.48	4.78, 12.74	0.12, 6.66
	UB-612 vs. BNT162b2					
Rate difference			13.5	21.6	17.9	16.6
95% CI			4.72, 22.34	12.35, 30.89	10.51, 25.37	8.45, 24.77

Increase in Neutralizing Antibody Titer: GMFI

		Baseline	Day 15	Day 29	Month 6	Month 12
	UB-612					
n			200	201	191	110
Geometric mean (geometric SD)			9.251 (2.8335)	7.688 (2.9556)	2.295 (2.7688)	1.769 (2.8269)
Median			9.022	7.021	2.167	1.707
Q1, Q3			4.375, 18.899	3.861, 14.520	1.232, 4.292	0.880, 3.316
Min, Max			0.45, 508.84	0.35, 203.57	0.26, 44.27	0.18, 34.87
	BNT162b2					
n			197	198	194	118
Geometric mean (geometric SD)			5.953 (2.5340)	4.235 (2.4692)	1.288 (2.3947)	1.063 (2.1591)
Median			5.459	4.058	1.151	1.097
Q1, Q3			3.290, 9.798	2.224, 7.122	0.793, 2.280	0.675, 1.582
Min, Max			0.27, 219.11	0.58, 58.85	0.07, 20.04	0.07, 12.00

	BBIBP-CorV (n)	UB-612 (n)	GMT Ratio (95% CI)	P-value non-inferiority	P Value superiority
Age					
18-25	44	42	10.69 (6.91, 16.53)	<.0001	<.0001
26-49	99	88	4.32 (3.21, 5.82)	<.0001	<.0001
50-64	22	33	5.91 (3.46, 10.08)	<.0001	<.0001
Gender					
Male	95	96	5.86 (4.38, 7.85)	<.0001	<.0001
Female	71	71	5.90 (4.20, 8.29)	<.0001	<.0001
Comorbidities					
Any	44	45	6.15 (4.15, 9.12)	<.0001	<.0001
Obesity	13	16	4.37 (2.26, 8.46)	<.0001	0.0001
Smoking	28	26	6.87 (4.14, 11.41)	<.0001	<.0001
Cardiovascular Disease	7	7	13.96 (3.00, 64.88)	0.0009	0.0042
N-protein Seropositivity ELISA					
>= 200	157	158	5.55 (4.43, 6.96)	<.0001	<.0001
< 200	9	8	10.43 (3.04, 35.79)	0.0002	0.0014

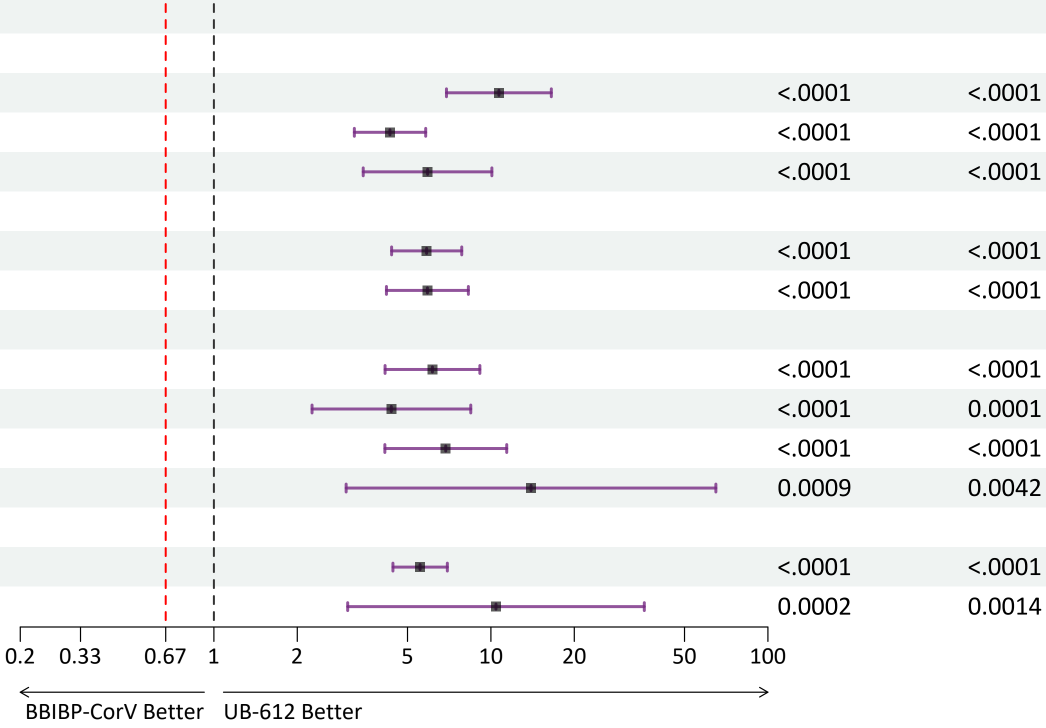

Supplemental figure 1A. BBIBP-CorV substudy subgroup analysis of day 29 Wuhan neutralizing antibody GMT

	BBIBP-CorV (n)	UB-612 (n)	GMT Ratio (95% CI)	P-value non-inferiority	P Value superiority
Age					
18-25	44	42	10.50 (6.77, 16.29)	<.0001	<.0001
26-49	99	88	4.26 (2.98, 6.09)	<.0001	<.0001
50-64	22	33	6.82 (3.66, 12.72)	<.0001	<.0001
Gender					
Male	95	96	6.26 (4.39, 8.92)	<.0001	<.0001
Female	71	71	5.71 (4.01, 8.14)	<.0001	<.0001
Comorbidities					
Any	44	45	5.13 (3.15, 8.36)	<.0001	<.0001
Obesity	13	16	5.17 (2.55, 10.48)	<.0001	<.0001
Smoking	28	26	4.99 (2.55, 9.78)	<.0001	<.0001
Cardiovascular Disease	7	7	9.70 (2.19, 42.97)	0.0016	0.0079
N-protein Seropositivity ELISA					
>= 200	157	158	5.90 (4.56, 7.64)	<.0001	<.0001
< 200	9	8	4.54 (0.77, 26.68)	0.0181	0.0870

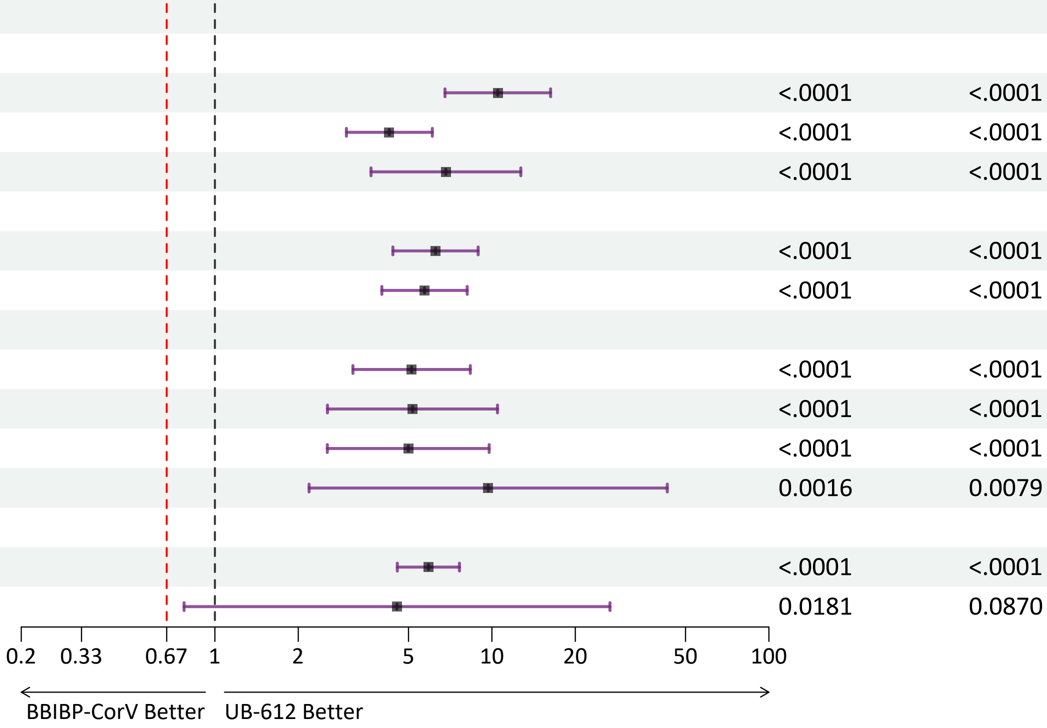

Supplemental figure 1B. BBIBP-CorV substudy subgroup analysis of day 29 Omicron BA.5 neutralizing antibody GMT

	BBIBP-CorV (n)	UB-612 (n)	Sero-response Rate Difference (95% CI)	P-value non-inferiority	P Value superiority
Age					
18-25	44	42	64.5 (49.23, 79.78)	<.0001	<.0001
26-49	99	88	48.9 (37.10, 60.63)	<.0001	<.0001
50-64	22	33	71.2 (54.20, 88.23)	<.0001	<.0001
Gender					
Male	95	96	59.3 (48.39, 70.18)	<.0001	<.0001
Female	71	71	53.5 (40.69, 66.35)	<.0001	<.0001
Comorbidities					
Any	44	45	59.9 (44.62, 75.18)	<.0001	<.0001
Obesity	13	16	54.8 (27.01, 82.60)	<.0001	0.0025
Smoking	28	26	54.1 (33.92, 74.32)	<.0001	<.0001
Cardiovascular Disease	7	7	71.4 (37.96, 100.00)	<.0001	0.0053
N-protein Seropositivity ELISA					
>= 200	157	158	55.6 (47.00, 64.28)	<.0001	<.0001
< 200	9	8	75.0 (44.99, 100.00)	<.0001	0.0012

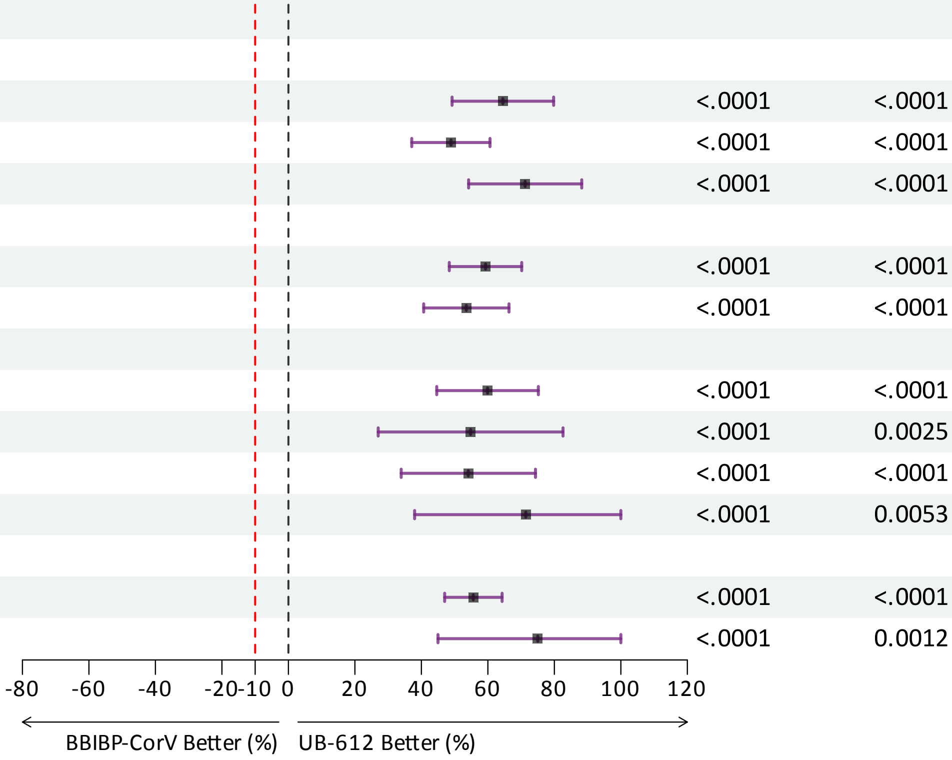

Supplemental figure 1C. BBIBP-CorV substudy subgroup analysis of day 29 Wuhan neutralizing antibody seroconversion rates

	BBIBP-CorV (n)	UB-612 (n)	Sero-response Rate Difference (95% CI)	P-value non-inferiority	P Value superiority
Age					
18-25	44	42	69.2 (54.80, 83.51)	<.0001	<.0001
26-49	99	88	48.6 (36.27, 60.95)	<.0001	<.0001
50-64	22	33	68.2 (48.72, 87.64)	<.0001	<.0001
Gender					
Male	95	96	53.0 (41.38, 64.61)	<.0001	<.0001
Female	71	71	64.8 (52.43, 77.14)	<.0001	<.0001
Comorbidities					
Any	44	45	62.0 (46.00, 77.93)	<.0001	<.0001
Obesity	13	16	70.7 (44.88, 96.47)	<.0001	<.0001
Smoking	28	26	54.4 (33.40, 75.39)	<.0001	<.0001
Cardiovascular Disease	7	7	71.4 (37.96, 100.00)	<.0001	0.0053
N-protein Seropositivity ELISA					
>= 200	157	158	57.5 (48.72, 66.32)	<.0001	<.0001
< 200	9	8	63.9 (27.53, 100.00)	<.0001	0.0075

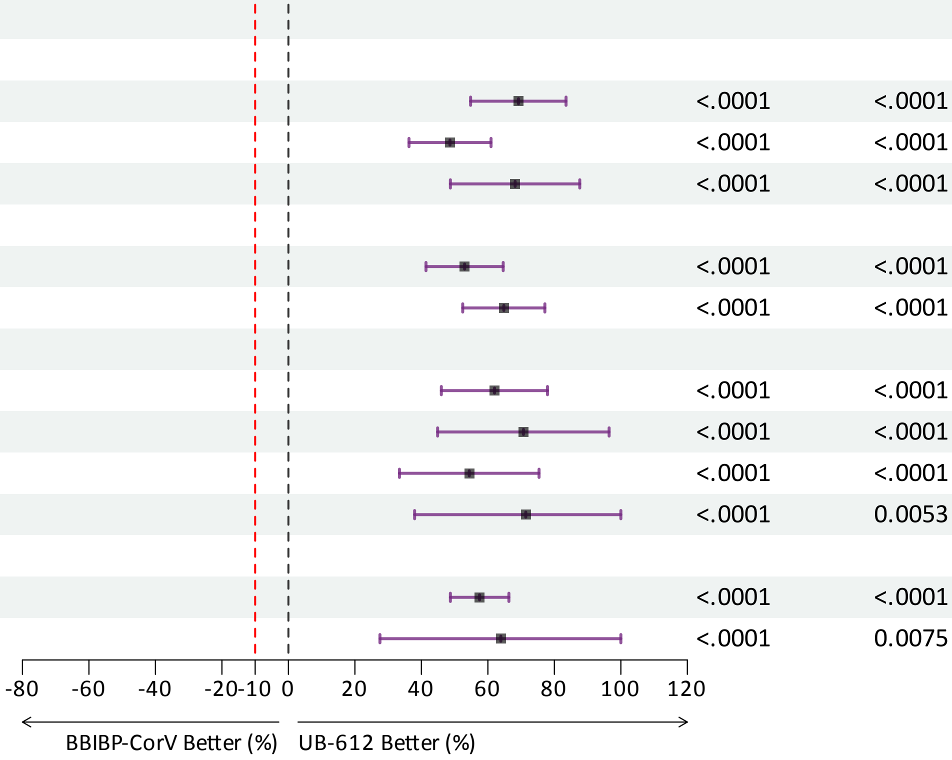

Supplemental figure 1D. BBIBP-CorV substudy subgroup analysis of day 29 Omicron BA.5 neutralizing antibody seroconversion rates

	ChAdOx1-S (n)	UB-612 (n)	GMT Ratio (95% CI)	P-value non-inferiority	P-value superiority
Age					
18-25	38	31	2.00 (1.25, 3.19)	<.0001	0.0044
26-49	50	45	1.61 (1.09, 2.37)	<.0001	0.0172
50-64	5	15	4.35 (1.30, 14.55)	0.0024	0.0202
Gender					
Male	52	50	1.63 (1.10, 2.41)	<.0001	0.0158
Female	41	41	2.36 (1.53, 3.63)	<.0001	0.0002
Comorbidities					
Any	38	26	1.53 (0.89, 2.63)	0.0018	0.1237
Obesity	9	11	1.10 (0.31, 3.99)	0.2098	0.8712
Smoking	29	18	2.10 (1.13, 3.90)	0.0003	0.0195
N-protein Seropositivity ELISA					
Reactive	88	84	1.97 (1.46, 2.66)	<.0001	<.0001
Not Reactive	5	6	1.12 (0.23, 5.45)	0.2359	0.8747

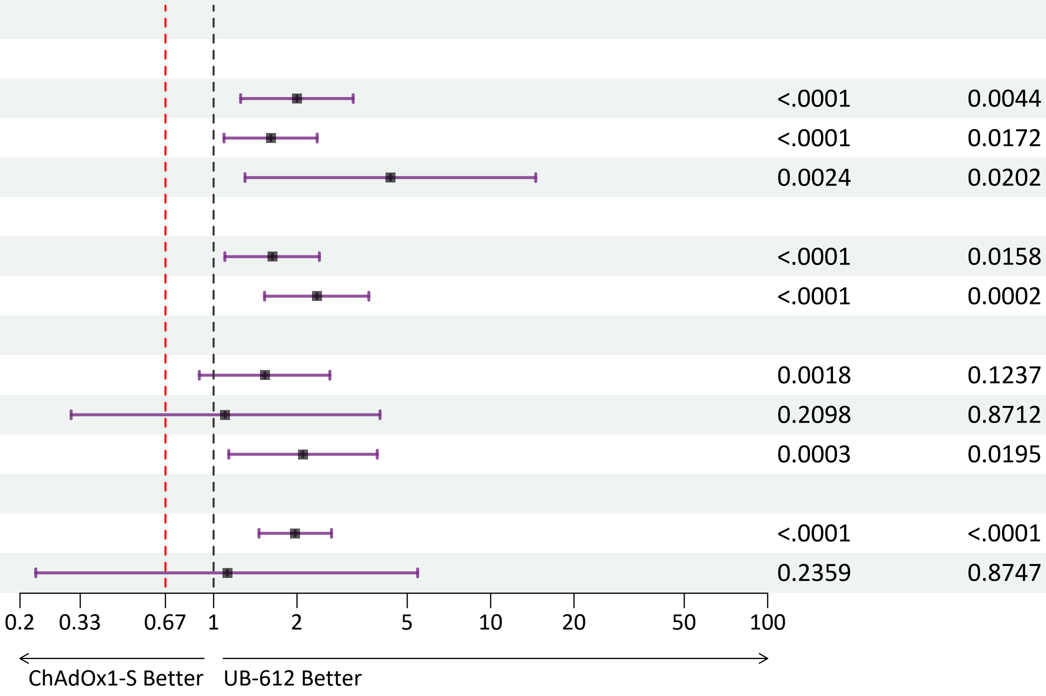

Supplemental figure 2A. ChAdOx1-S substudy subgroup analysis of day 29 Wuhan neutralizing antibody GMT

	ChAdOx1-S (n)	UB-612 (n)	GMT Ratio (95% CI)	P-value non-inferiority	P-value superiority
Age					
18-25	38	31	2.60 (1.45, 4.65)	<.0001	0.0017
26-49	50	45	2.78 (1.72, 4.49)	<.0001	<.0001
50-64	5	15	2.34 (0.47, 11.61)	0.0584	0.2763
Gender					
Male	52	50	2.99 (1.87, 4.79)	<.0001	<.0001
Female	41	41	2.69 (1.55, 4.65)	<.0001	0.0006
Comorbidities					
Any	38	26	2.81 (1.44, 5.47)	<.0001	0.0031
Obesity	9	11	1.58 (0.35, 7.08)	0.1216	0.5279
Smoking	29	18	4.09 (1.89, 8.84)	<.0001	0.0007
N-protein Seropositivity ELISA					
Reactive	88	84	2.80 (1.94, 4.04)	<.0001	<.0001
Not Reactive	5	6	2.15 (0.32, 14.66)	0.0972	0.3776

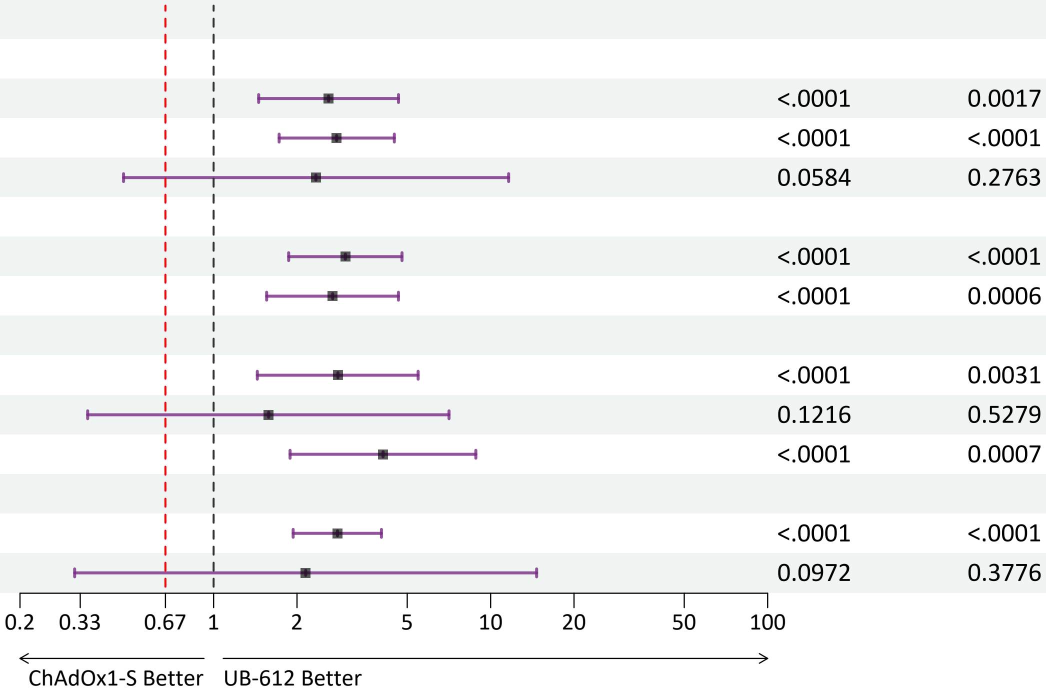

Supplemental figure 2B. ChAdOx1-S substudy subgroup analysis of day 29 Omicron BA.5 neutralizing antibody GMT

	ChAdOx1-S (n)	UB-612 (n)	Sero-response Rate Difference (95% CI)	P-value non-inferiority	P-value superiority
Age					
18-25	38	31	20.0 (2.94, 43.01)	0.0052	0.0918
26-49	50	45	26.7 (8.35, 44.98)	<.0001	0.0056
50-64	5	15	13.3 (-36.48, 63.15)	0.1793	0.6056
Gender					
Male	52	50	13.4 (-5.56, 32.33)	0.0078	0.1697
Female	41	41	36.6 (17.85, 55.32)	<.0001	0.0004
Comorbidities					
Any	38	26	18.4 (-5.82, 42.67)	0.0108	0.1379
Obesity	9	11	3.0 (-38.88, 44.94)	0.2711	0.8876
Smoking	29	18	30.1 (1.96, 58.20)	0.0026	0.0426
N-protein Seropositivity ELISA					
Reactive	88	84	26.1 (12.22, 40.05)	<.0001	0.0004
Not Reactive	5	6	-10.0 (-68.69, 48.69)	0.5000	0.7401

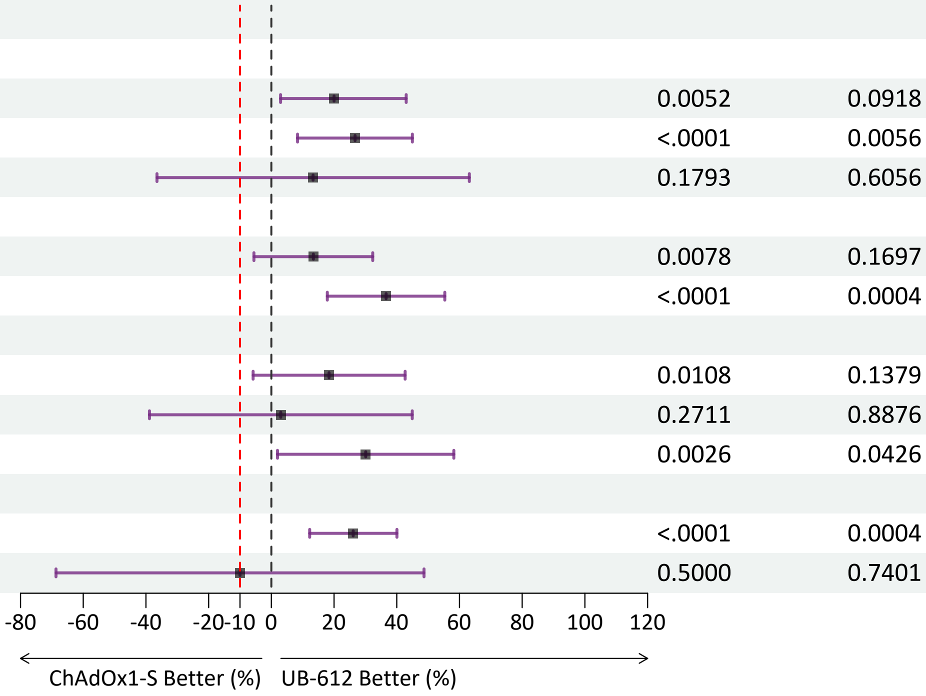

Supplemental figure 2C. ChAdOx1-S substudy subgroup analysis of day 29 Wuhan neutralizing antibody seroresponse rates

	ChAdOx1-S (n)	UB-612 (n)	Sero-response Rate Difference (95% CI)	P-value non-inferiority	P-value superiority
Age					
18-25	38	31	27.1 (4.24, 49.92)	0.0007	0.0249
26-49	50	45	33.6 (15.05, 52.07)	<.0001	0.0008
50-64	5	15	0.0 (-49.58, 49.58)	0.3463	1.0000
Gender					
Male	52	50	33.2 (14.92, 51.39)	<.0001	0.0008
Female	41	41	24.4 (3.73, 45.05)	0.0006	0.0250
Comorbidities					
Any	38	26	35.0 (11.73, 58.31)	<.0001	0.0059
Obesity	9	11	8.1 (-35.07, 51.23)	0.2058	0.7136
Smoking	29	18	41.2 (14.51, 67.86)	<.0001	0.0059
N-protein Seropositivity ELISA					
Reactive	88	84	31.0 (17.00, 45.02)	<.0001	<.0001
Not Reactive	5	6	3.3 (-42.69, 49.36)	0.2851	0.8865

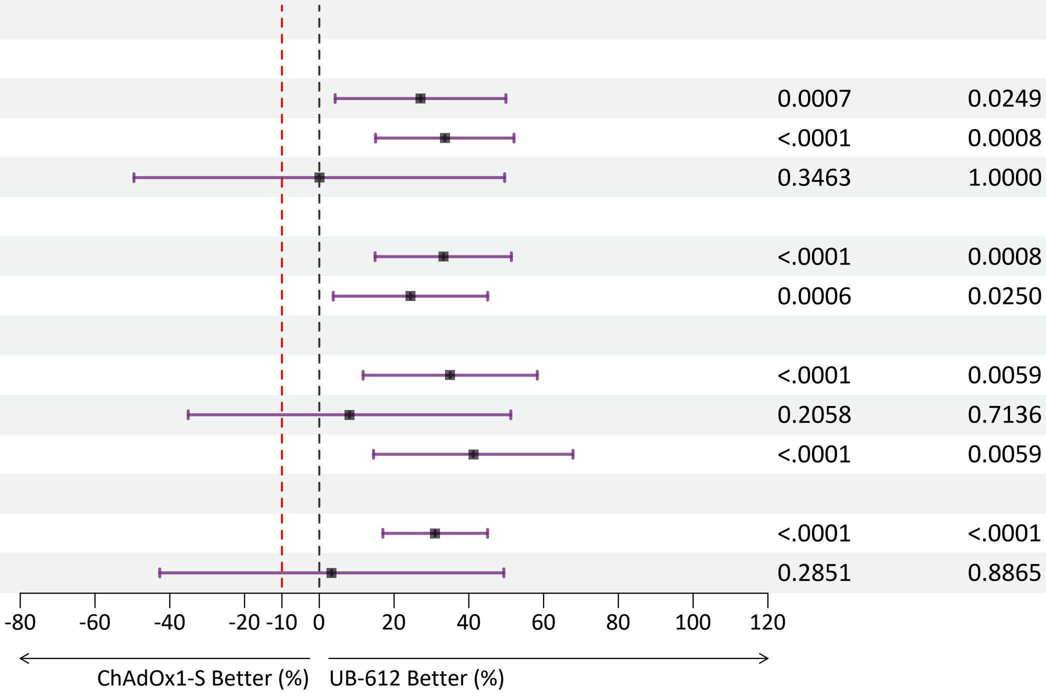

Supplemental figure 2D. ChAdOx1-S substudy subgroup analysis of day 29 Omicron BA.5 neutralizing antibody seroresponse rates

	BNT162b2 (n)	UB-612 (n)	GMT Ratio (95% CI)	P-value non-inferiority	P-value superiority
Age					
18-25	68	79	0.86 (0.68, 1.10)	0.0208	0.2256
26-49	111	98	1.15 (0.93, 1.43)	<.0001	0.2038
50-64	18	17	1.22 (0.81, 1.84)	0.0027	0.3224
Gender					
Male	108	109	1.05 (0.85, 1.30)	<.0001	0.6392
Female	90	92	1.02 (0.82, 1.26)	<.0001	0.8679
Comorbidities					
Any	73	79	1.26 (0.95, 1.66)	<.0001	0.1046
Obesity	36	45	1.26 (0.90, 1.77)	0.0002	0.1781
Smoking	38	28	1.19 (0.77, 1.84)	0.0054	0.4324
Cardiovascular Disease	6	11	2.77 (1.12, 6.81)	0.0025	0.0301
Chronic Lung Disease	7	5	2.35 (0.67, 8.20)	0.0246	0.1497
N-protein Seropositivity ELISA					
Reactive	188	186	1.04 (0.89, 1.22)	<.0001	0.6009
Not Reactive	10	15	0.94 (0.54, 1.66)	0.1097	0.8325

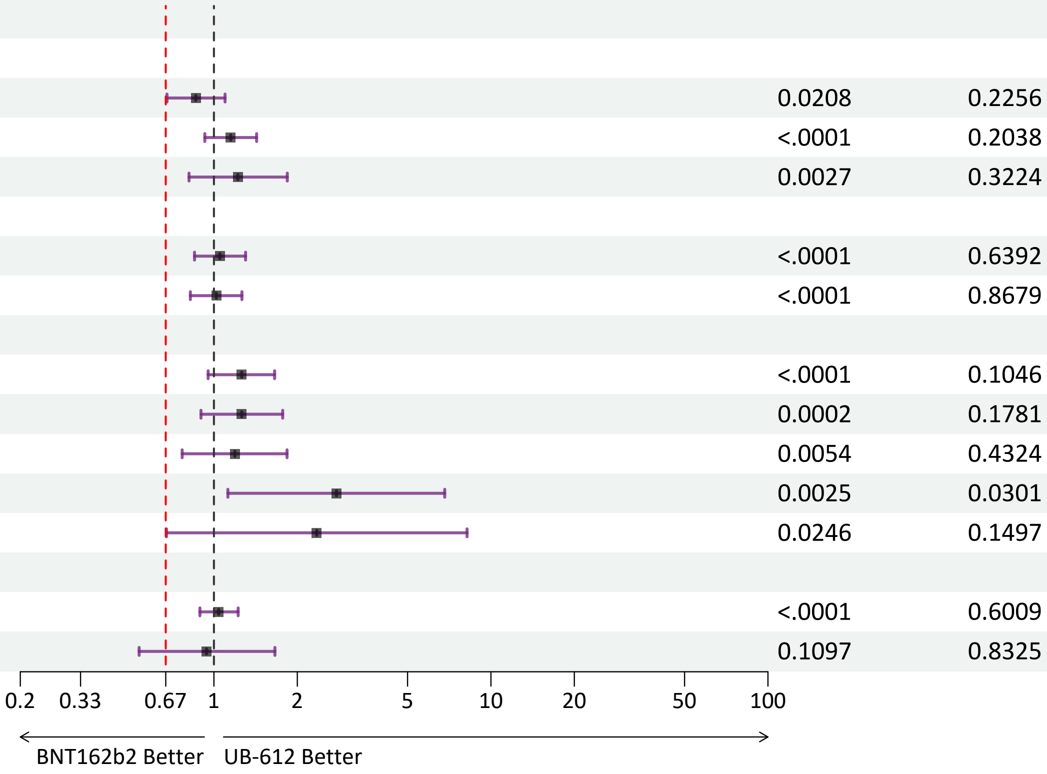

Supplemental figure 3A. BNT162b2 substudy subgroup analysis of day 29 Wuhan neutralizing antibody GMT

	BNT162b2 (n)	UB-612 (n)	GMT Ratio (95% CI)	P-value non-inferiority	P-value superiority
Age					
18-25	68	79	0.90 (0.69, 1.17)	0.0136	0.4153
26-49	111	98	1.22 (0.97, 1.54)	<.0001	0.0871
50-64	18	17	1.28 (0.65, 2.49)	0.0292	0.4622
Gender					
Male	108	109	1.06 (0.84, 1.35)	<.0001	0.6308
Female	90	92	1.14 (0.91, 1.43)	<.0001	0.2585
Comorbidities					
Any	73	79	1.36 (1.03, 1.81)	<.0001	0.0328
Obesity	36	45	1.22 (0.82, 1.81)	0.0018	0.3240
Smoking	38	28	1.45 (0.96, 2.18)	0.0002	0.0760
Cardiovascular Disease	6	11	1.22 (0.24, 6.16)	0.2184	0.7949
Chronic Lung Disease	7	5	4.96 (1.55, 15.89)	0.0024	0.0141
N-protein Seropositivity ELISA					
Reactive	188	186	1.13 (0.95, 1.34)	<.0001	0.1532
Not Reactive	10	15	1.03 (0.47, 2.27)	0.1341	0.9386

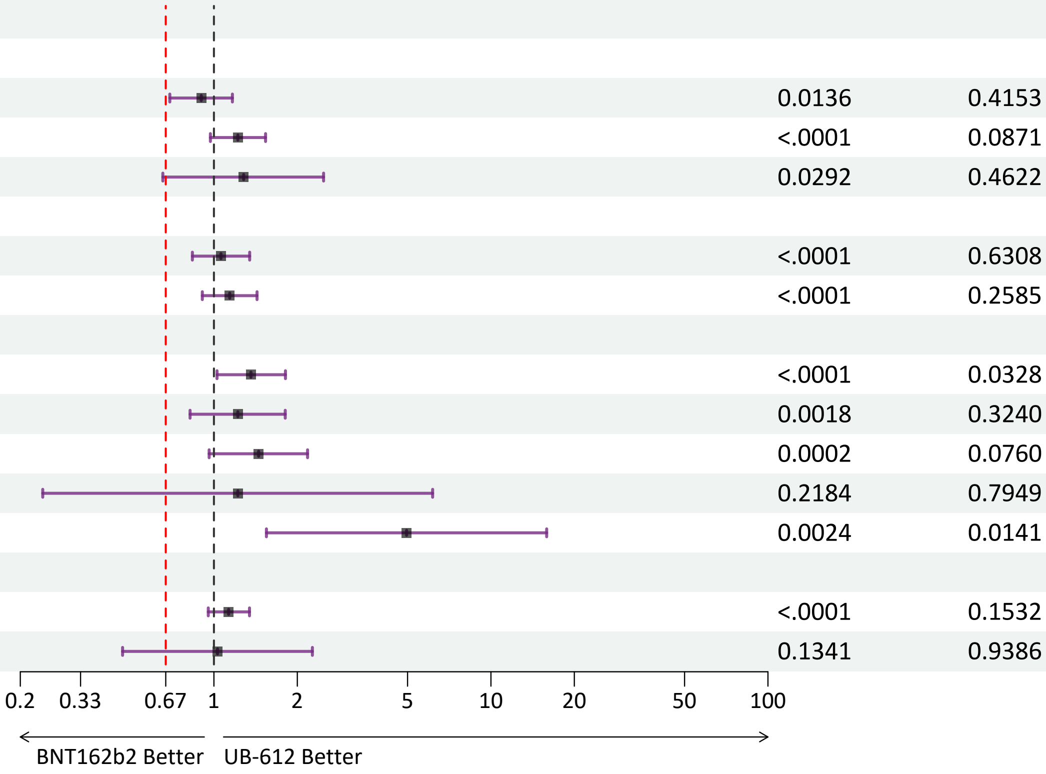

Supplemental figure 3B. BNT162b2 substudy subgroup analysis of day 29 Omicron BA.5 neutralizing antibody GMT

	BNT162b2 (n)	UB-612 (n)	Sero-response Rate Difference (95% CI)	P-value non-inferiority	P-value superiority
Age					
18-25	68	79	-6.9 (-22.99, 9.14)	0.3538	0.4000
26-49	111	98	6.1 (-6.89, 19.06)	0.0075	0.3600
50-64	18	17	4.9 (-18.14, 27.94)	0.1025	0.6787
Gender					
Male	108	109	1.3 (-11.60, 14.14)	0.0431	0.8472
Female	90	92	-0.3 (-14.30, 13.72)	0.0872	0.9677
Comorbidities					
Any	73	79	0.3 (-15.07, 15.63)	0.0947	0.9717
Obesity	36	45	-6.1 (-25.68, 13.46)	0.3485	0.5445
Smoking	38	28	2.6 (-21.76, 27.02)	0.1551	0.8326
Cardiovascular Disease	6	11	-1.5 (-39.05, 36.02)	0.3289	0.9376
Chronic Lung Disease	7	5	-5.7 (-49.32, 37.89)	0.4236	0.7934
N-protein Seropositivity ELISA					
Reactive	188	186	0.1 (-9.72, 9.98)	0.0220	0.9800
Not Reactive	10	15	0.0 (-32.01, 32.01)	0.2701	1.0000

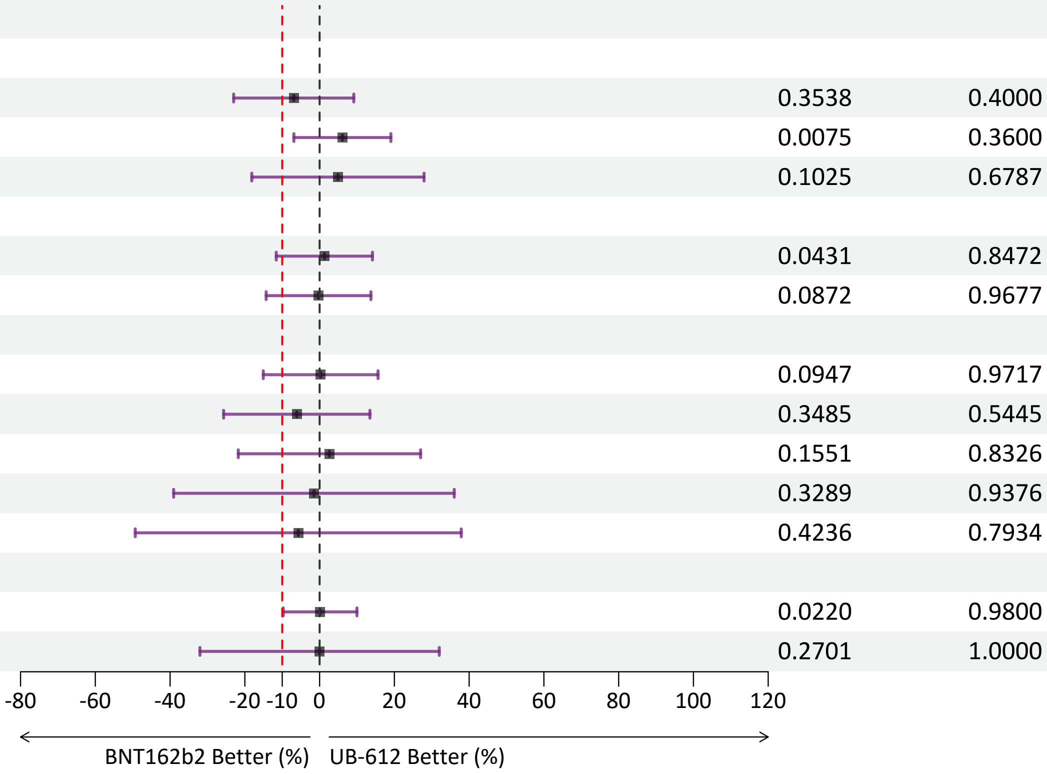

Supplemental figure 3C. BNT162b2 substudy subgroup analysis of day 29 Wuhan neutralizing antibody seroresonse rates

	BNT162b2 (n)	UB-612 (n)	Sero-response Rate Difference (95% CI)	P-value non-inferiority	P-value superiority
Age					
18-25	68	79	-2.9 (-19.01, 13.16)	0.1943	0.7220
26-49	111	98	10.8 (-2.63, 24.17)	0.0012	0.1178
50-64	18	17	-2.3 (-34.76, 30.19)	0.3208	0.8902
Gender					
Male	108	109	5.9 (-7.10, 18.89)	0.0083	0.3747
Female	90	92	2.2 (-12.35, 16.70)	0.0502	0.7693
Comorbidities					
Any	73	79	6.6 (-9.29, 22.46)	0.0203	0.4169
Obesity	36	45	-4.4 (-26.28, 17.39)	0.3090	0.6904
Smoking	38	28	23.9 (0.16, 47.59)	0.0026	0.0548
Cardiovascular Disease	6	11	3.0 (-44.20, 50.26)	0.2944	0.9006
Chronic Lung Disease	7	5	37.1 (-13.58, 87.87)	0.0343	0.1982
N-protein Seropositivity ELISA					
Reactive	188	186	2.2 (-7.89, 12.28)	0.0089	0.6704
Not Reactive	10	15	26.7 (-8.23, 61.56)	0.0197	0.1262

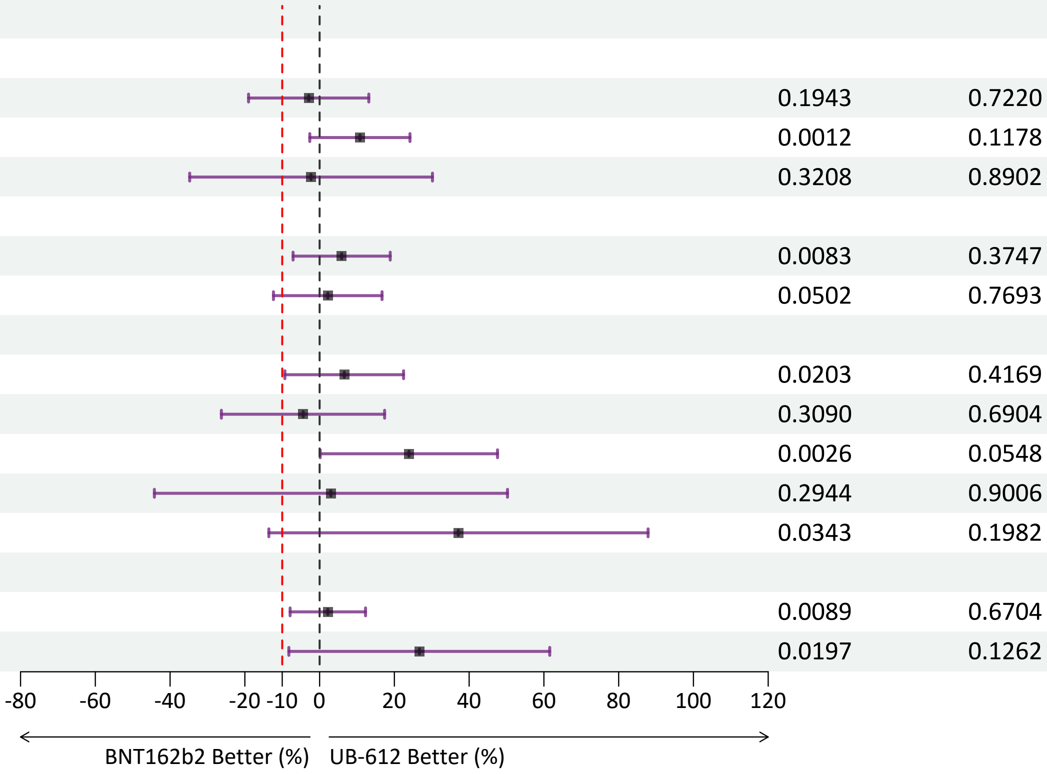

Supplemental figure 3D. BNT162b2 substudy subgroup analysis of day 29 Omicron BA.5 neutralizing antibody seroresponse rates

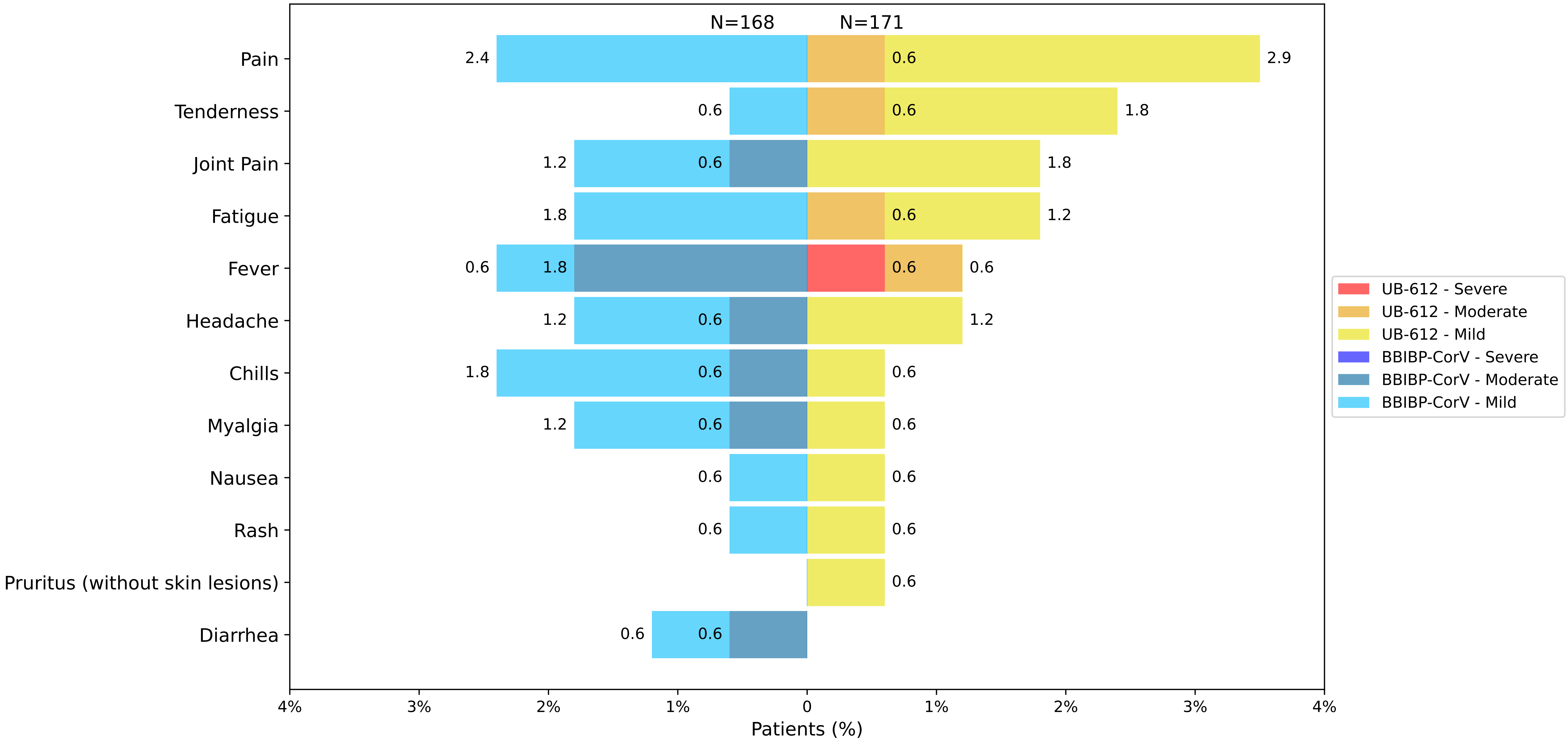

Supplemental figure 4A. BBIBP-CorV substudy solicited AEs (subjects diaries) by preferred term and severity

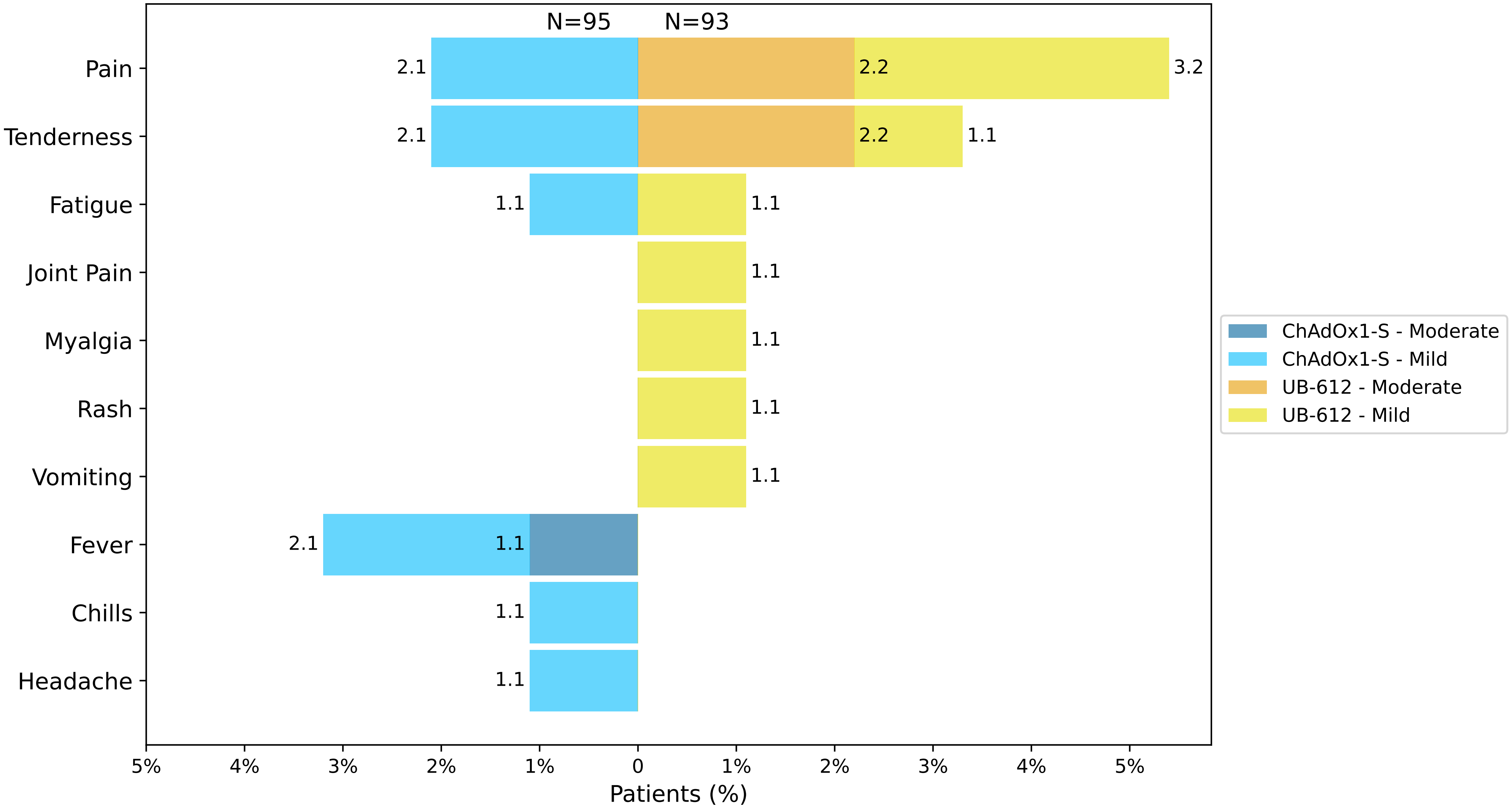

Supplemental figure 4B. ChAdOx1-S substudy solicited AEs (subjects diaries) by preferred term and severity

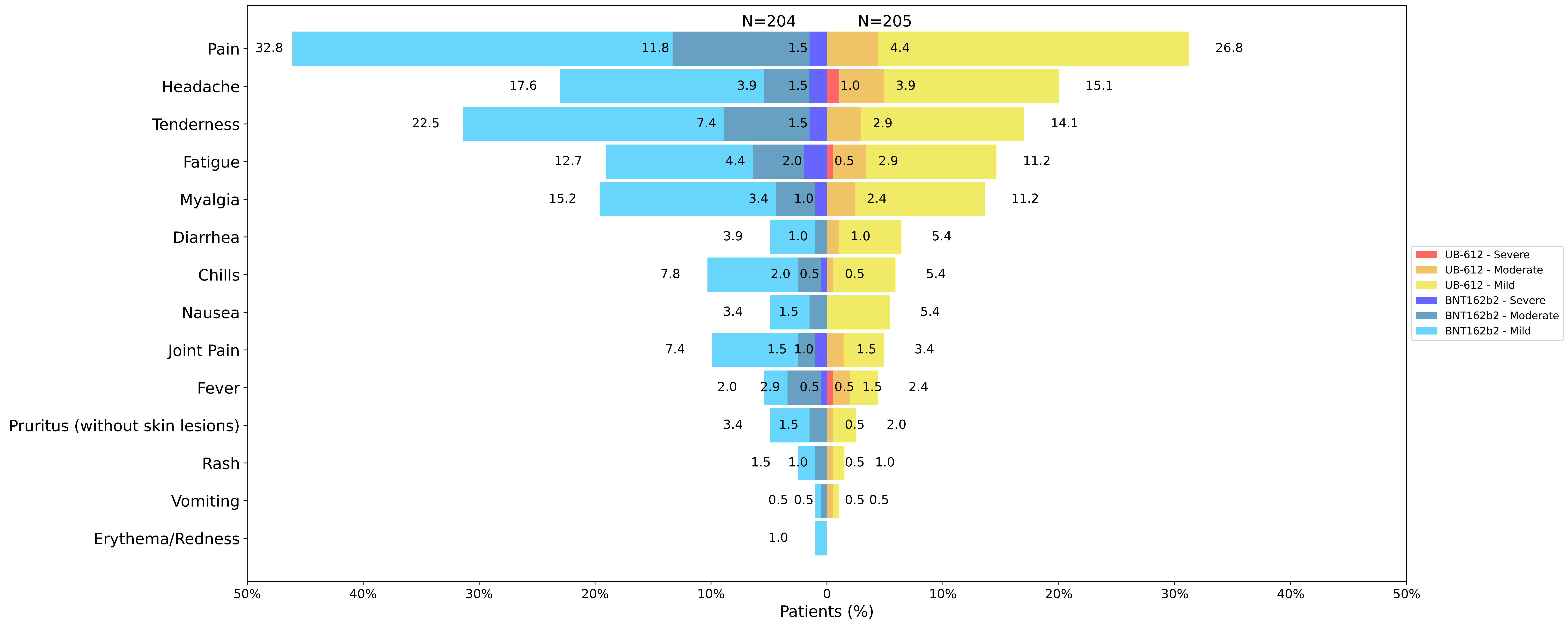

Supplemental figure 4C. BNT162b2 substudy solicited AEs (subjects diaries) by preferred term and severity

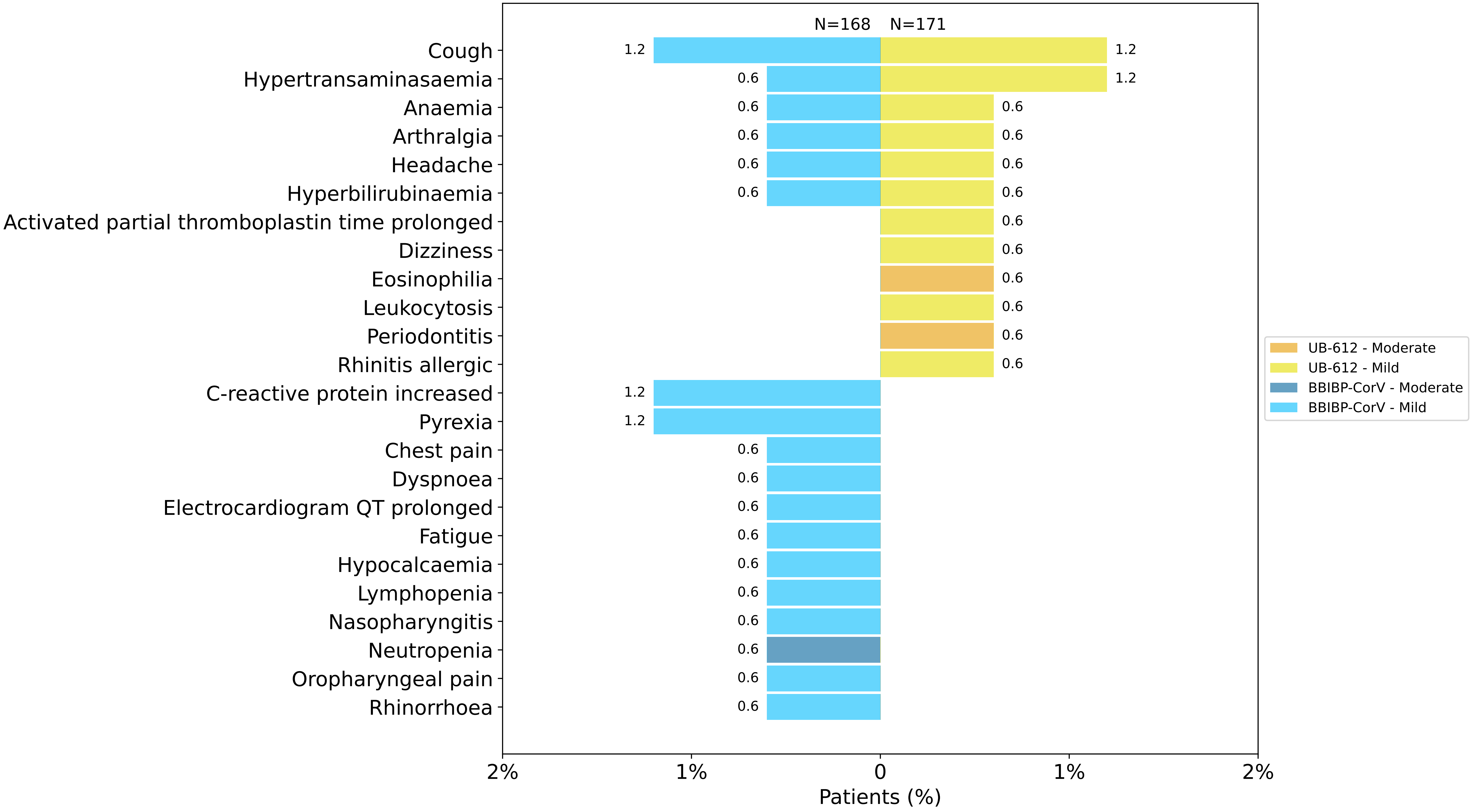

Supplemental figure 5A. BBIBP-CorV substudy TEAE (without AEs matching the definition of solicited AEs) by preferred term and severity

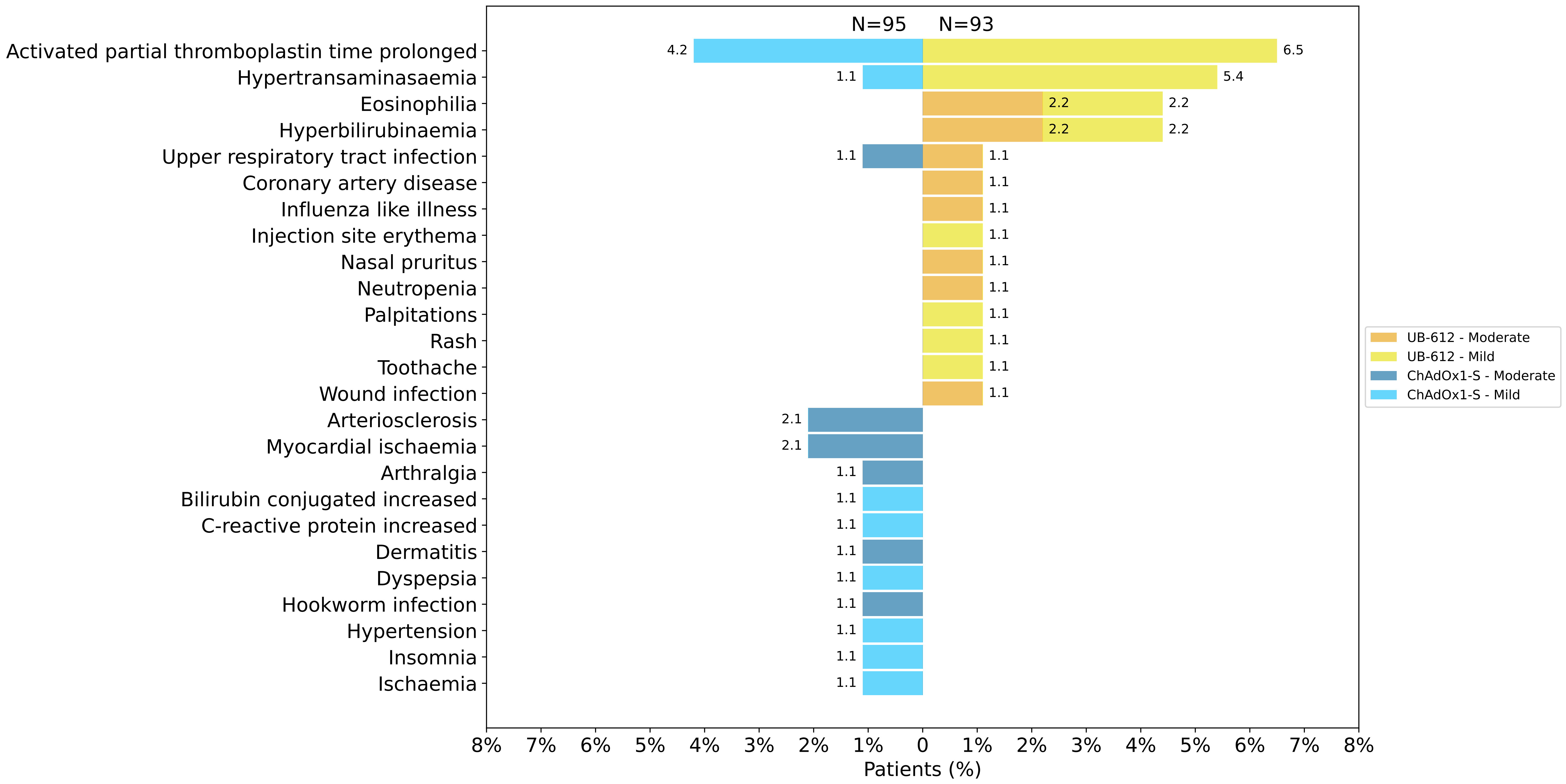

Supplemental figure 5B. ChAdOx1-S substudy TEAE (without AEs matching the definition of solicited AEs) by preferred term and severity

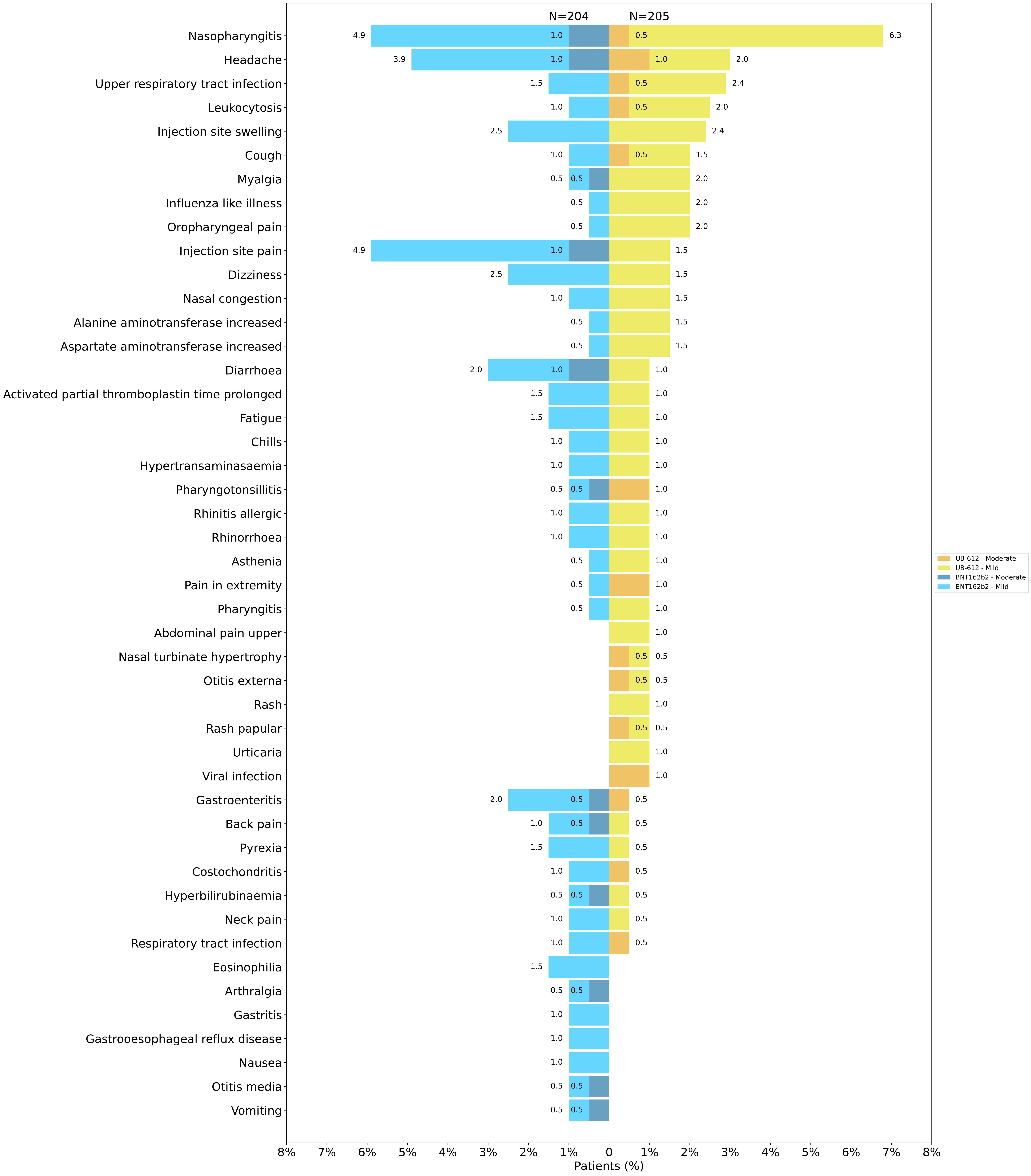

Supplemental figure 5C. BNT162b2 substudy TEAE (without AEs matching the definition of solicited AEs) by preferred term and severity

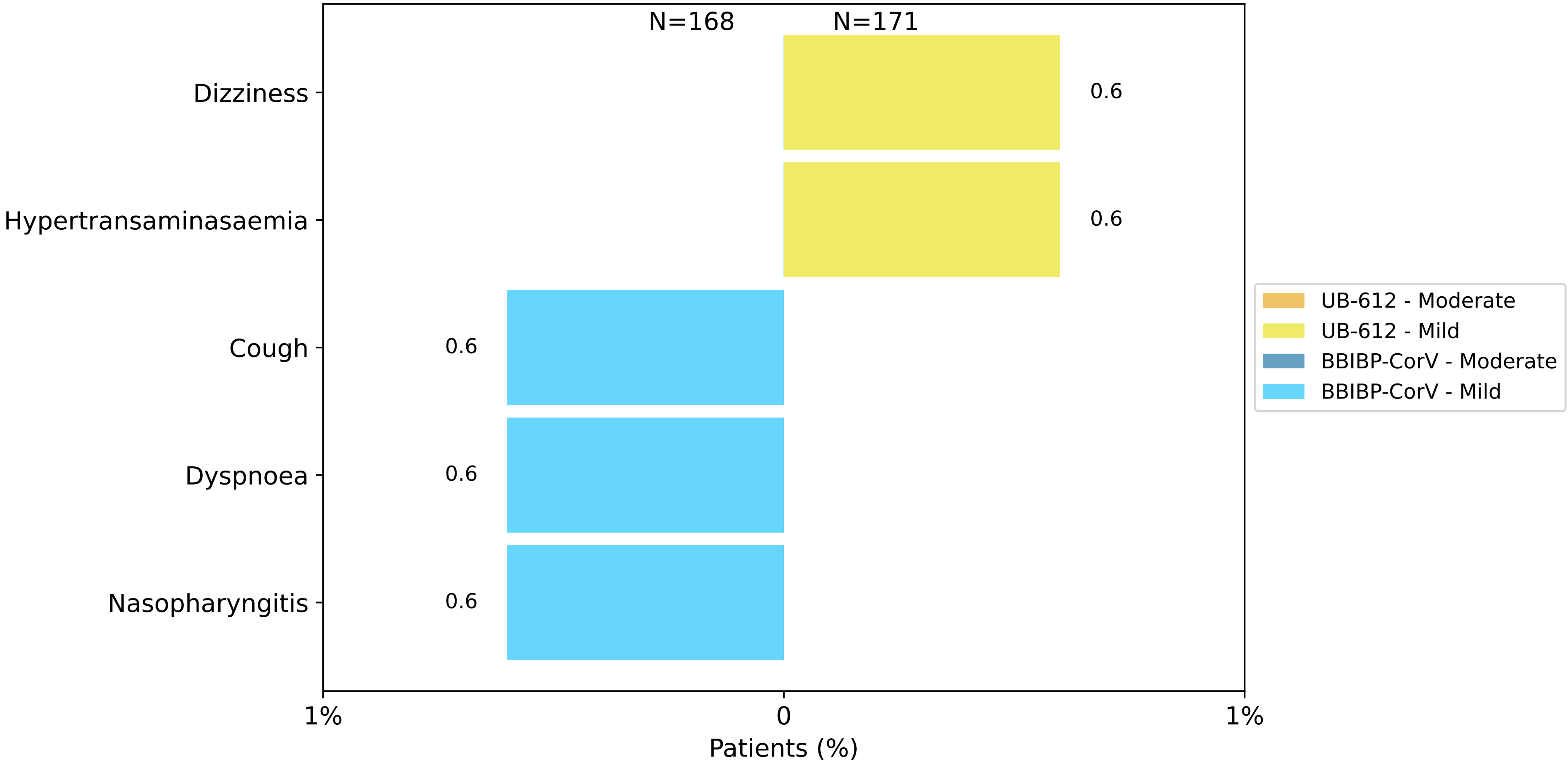

Supplemental figure 6A. BBIBP-CorV substudy treatement-related TEAE (without AEs matching the definition of solicited AEs) by preferred term and severity

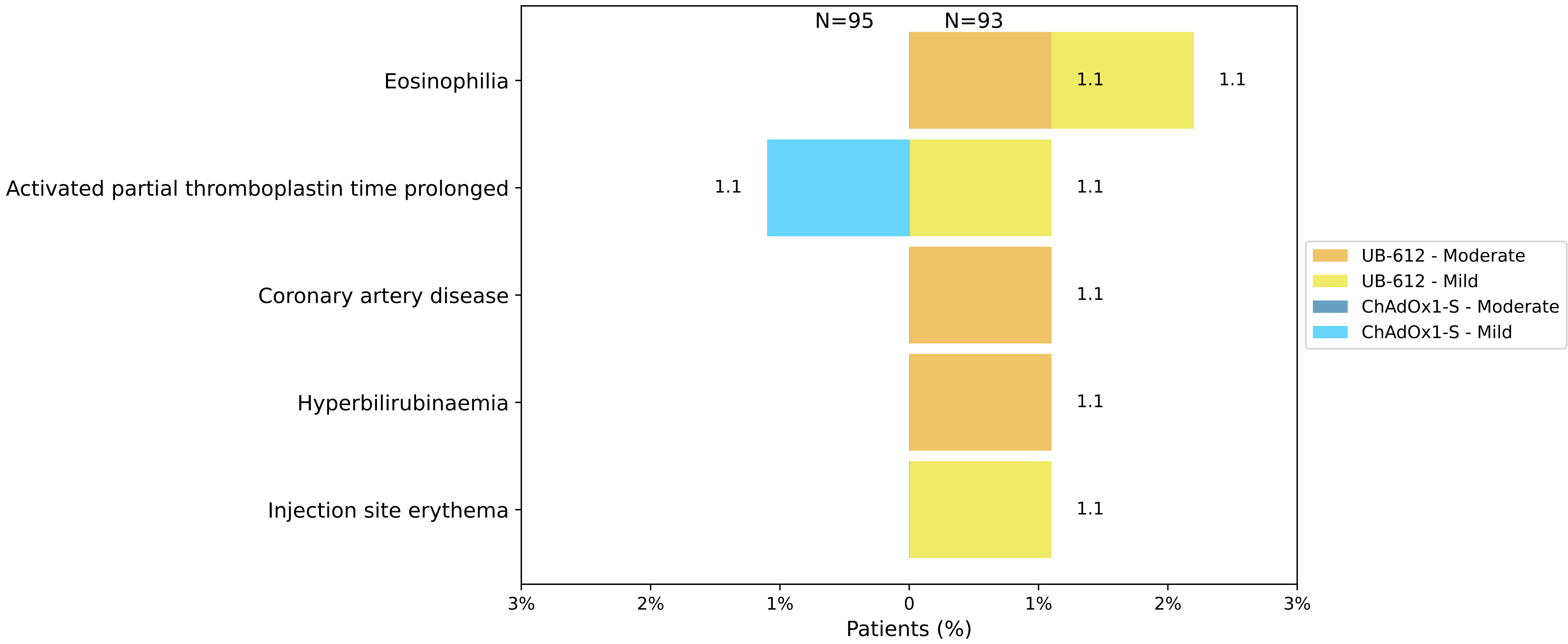

Supplemental figure 6B. ChAdOx1-Ssubstudy treatement-related TEAE (without AEs matching the definition of solicited AEs) by preferred term and severity

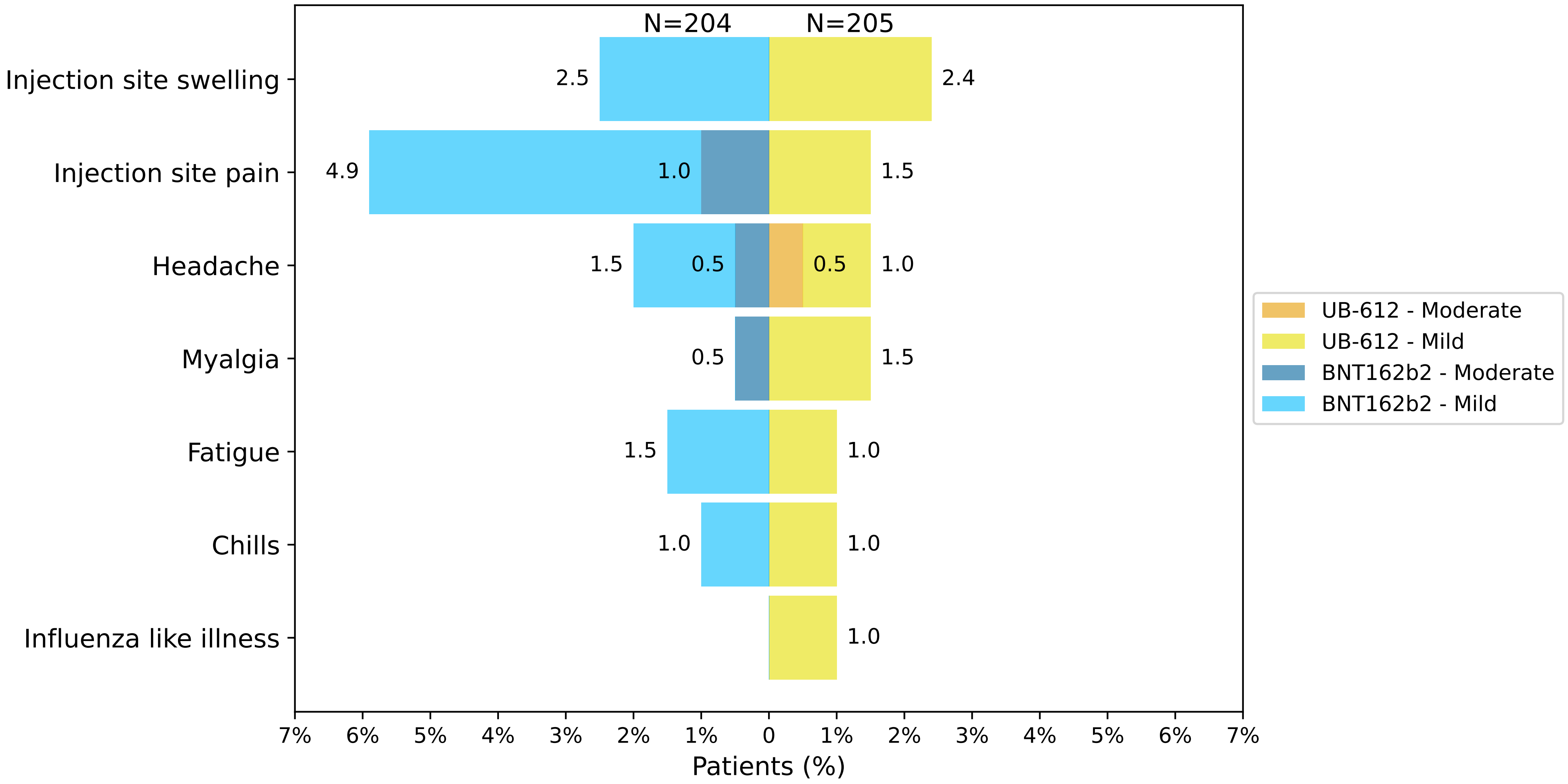

Supplemental figure 6C. BNT162b2 substudy treatement-related TEAE (without AEs matching the definition of solicited AEs) by preferred term and severity

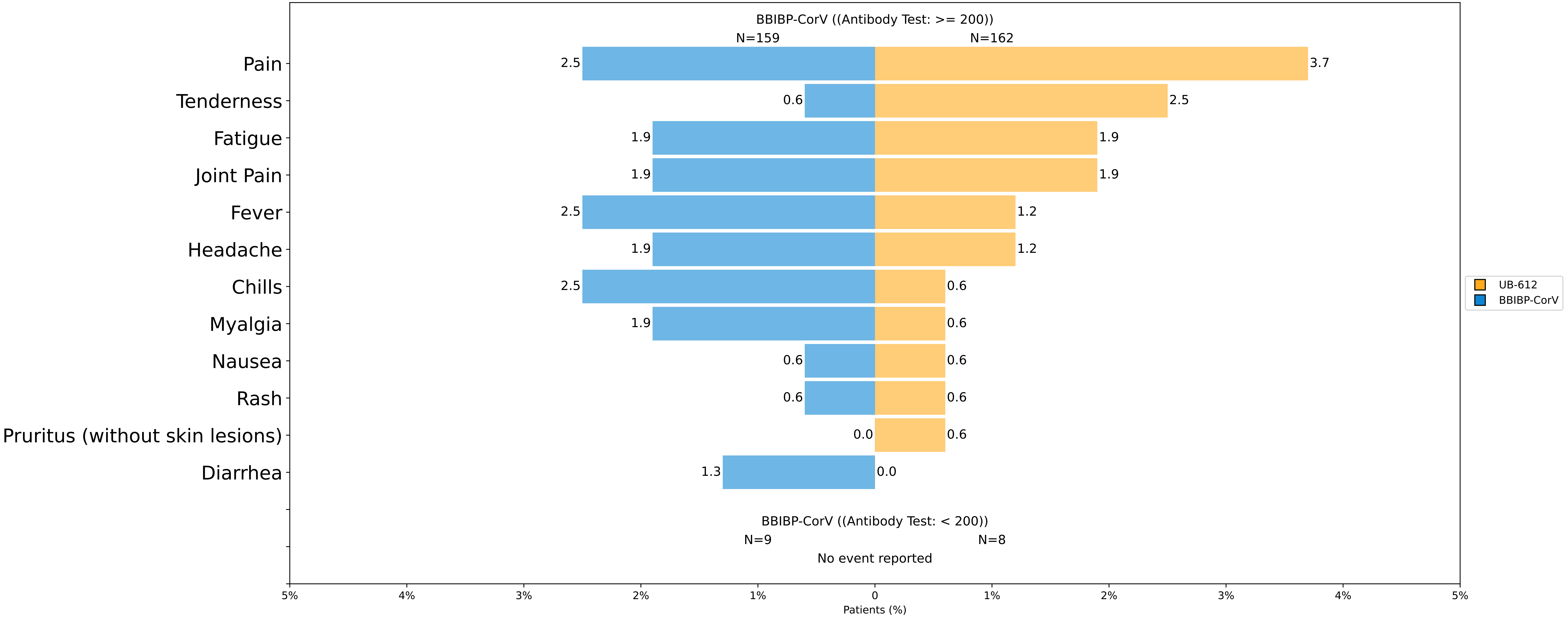

Supplemental figure 7A. BBIBP-CorV substudy subgroup reactogenicity (solicited AEs on subjects diaries and reported by PI) by N-protein seropositivity.

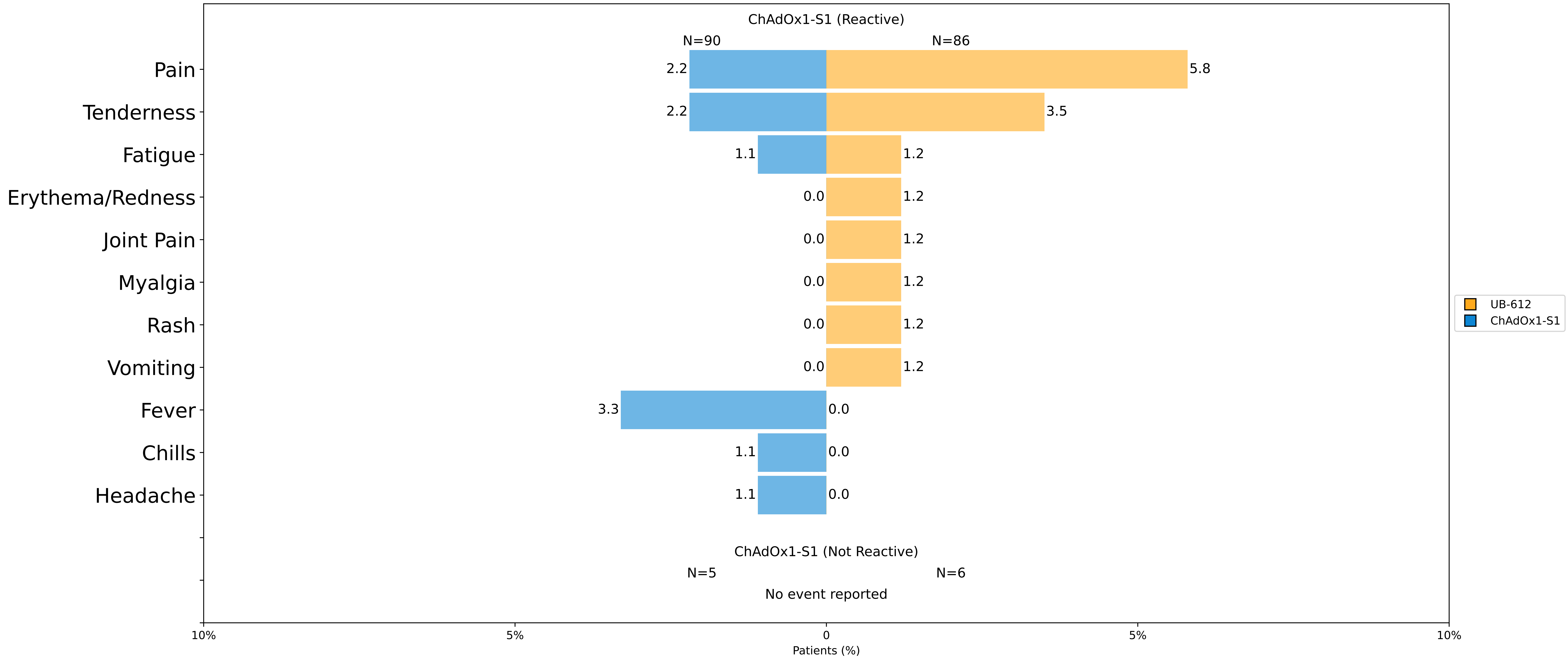

Supplemental figure 7B. ChAdOx1-S substudy subgroup reactogenicity (solicited AEs on subjects diaries and reported by PI) by N-protein seropositivity.

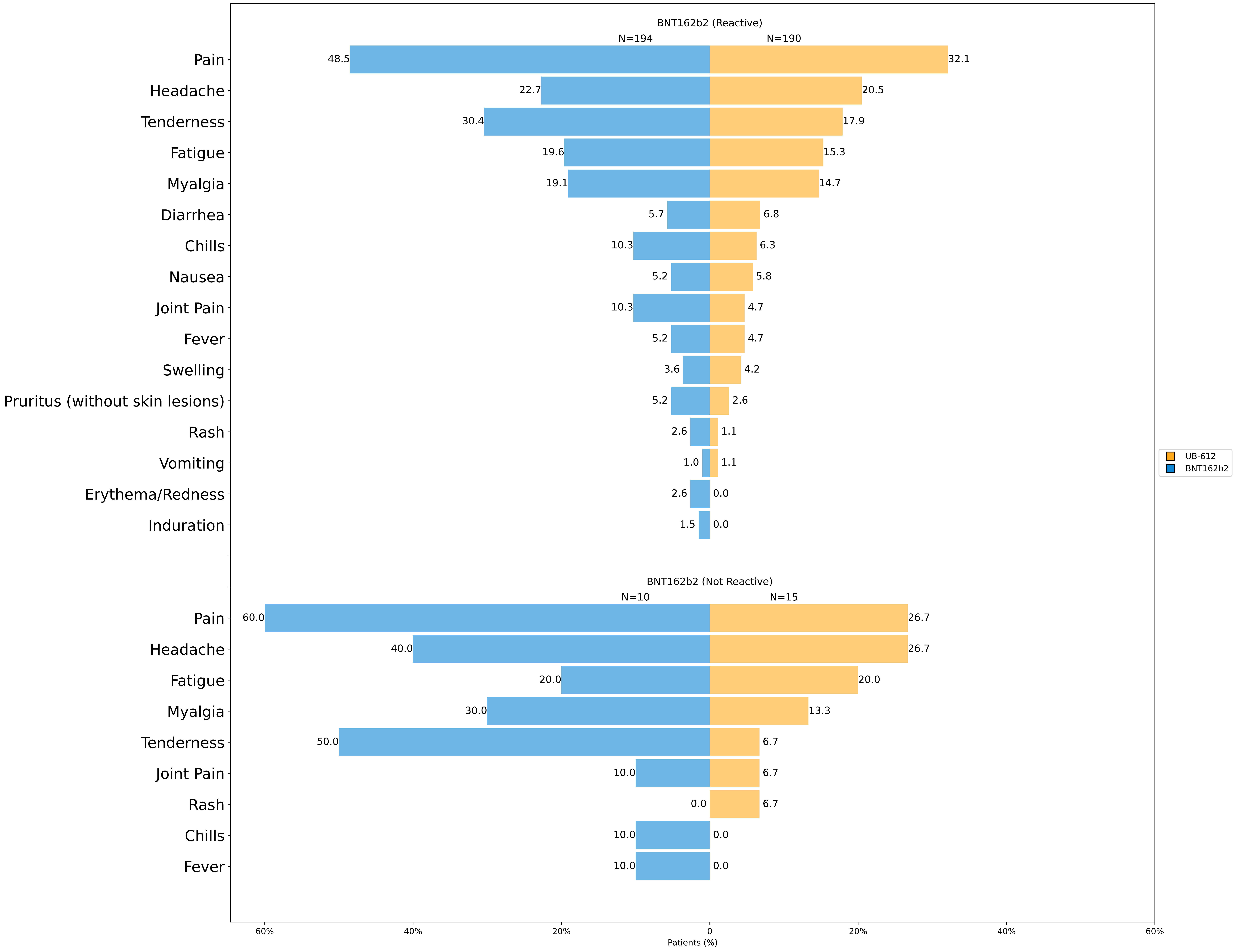

Supplemental figure 7C. BNT162b2 substudy subgroup reactogenicity (solicited AEs on subjects diaries and reported by PI) by N-protein seropositivity.

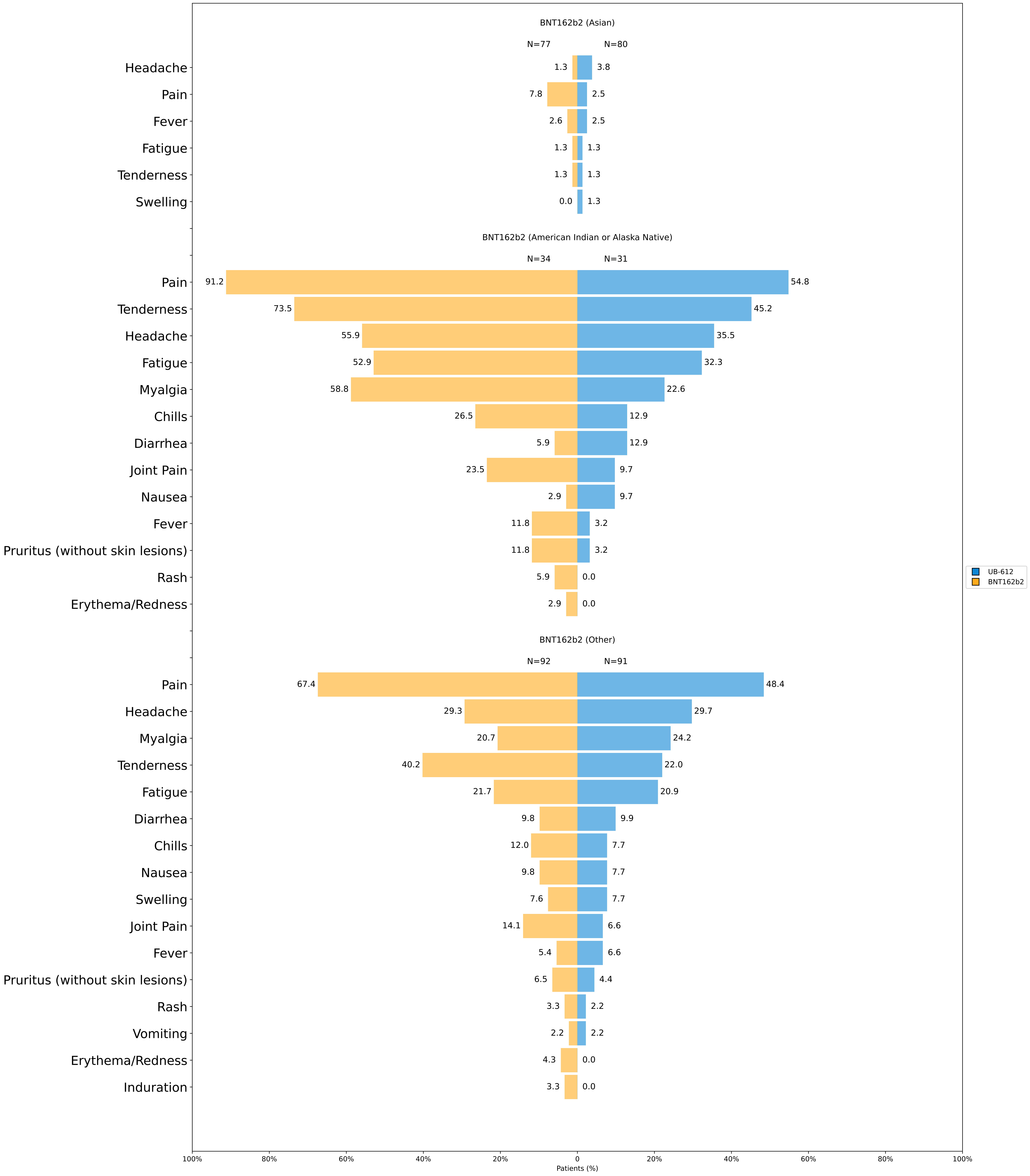

Supplemental figure 7D. BNT162b2 substudy subgroup reactogenicity (solicited AEs on subjects diaries and reported by PI) by race

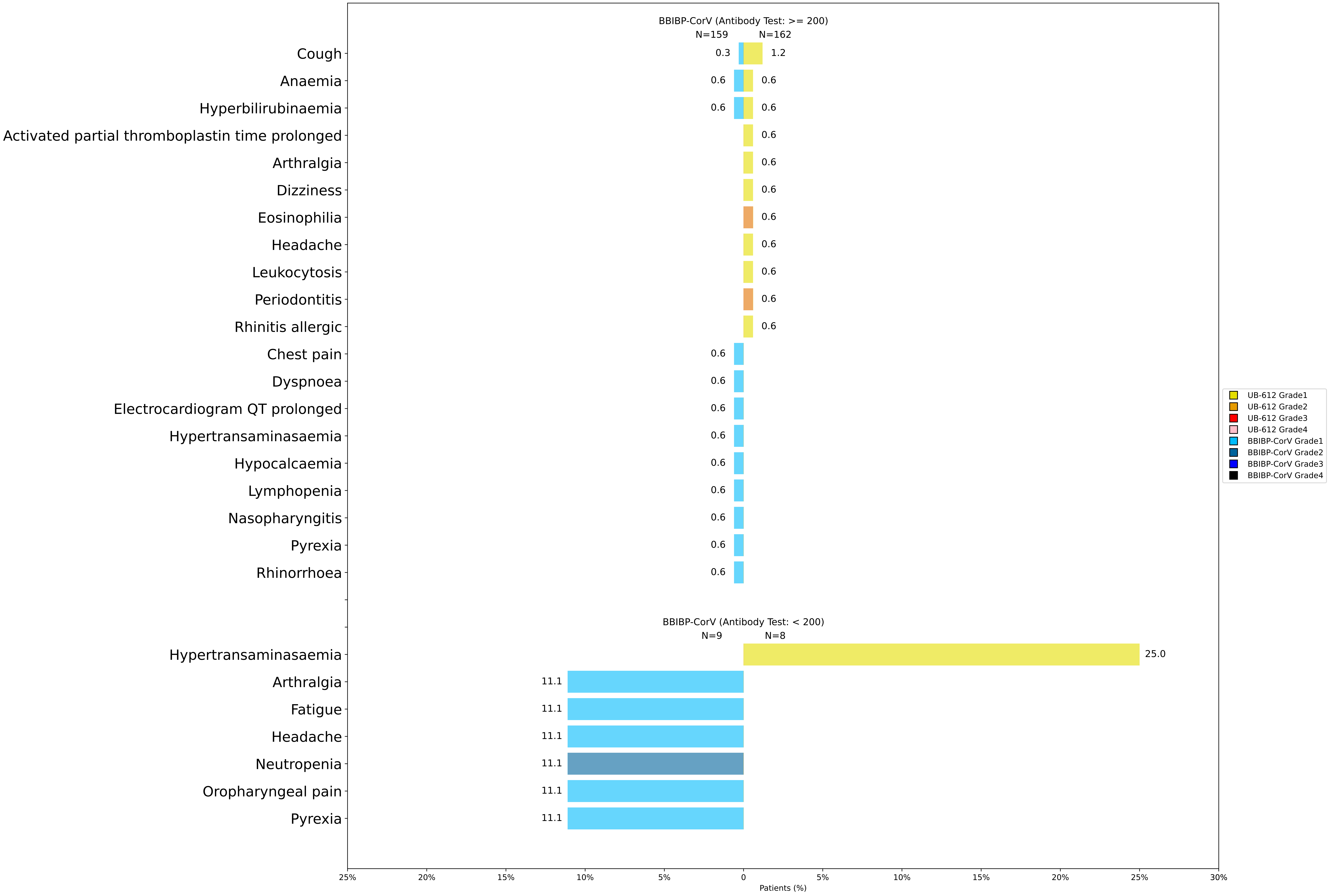

Supplemental figure 8A. BBIBP-CorV substudy subgroup TEAE (excluding terms matching solicited AEs) by N-protein seropositivity.

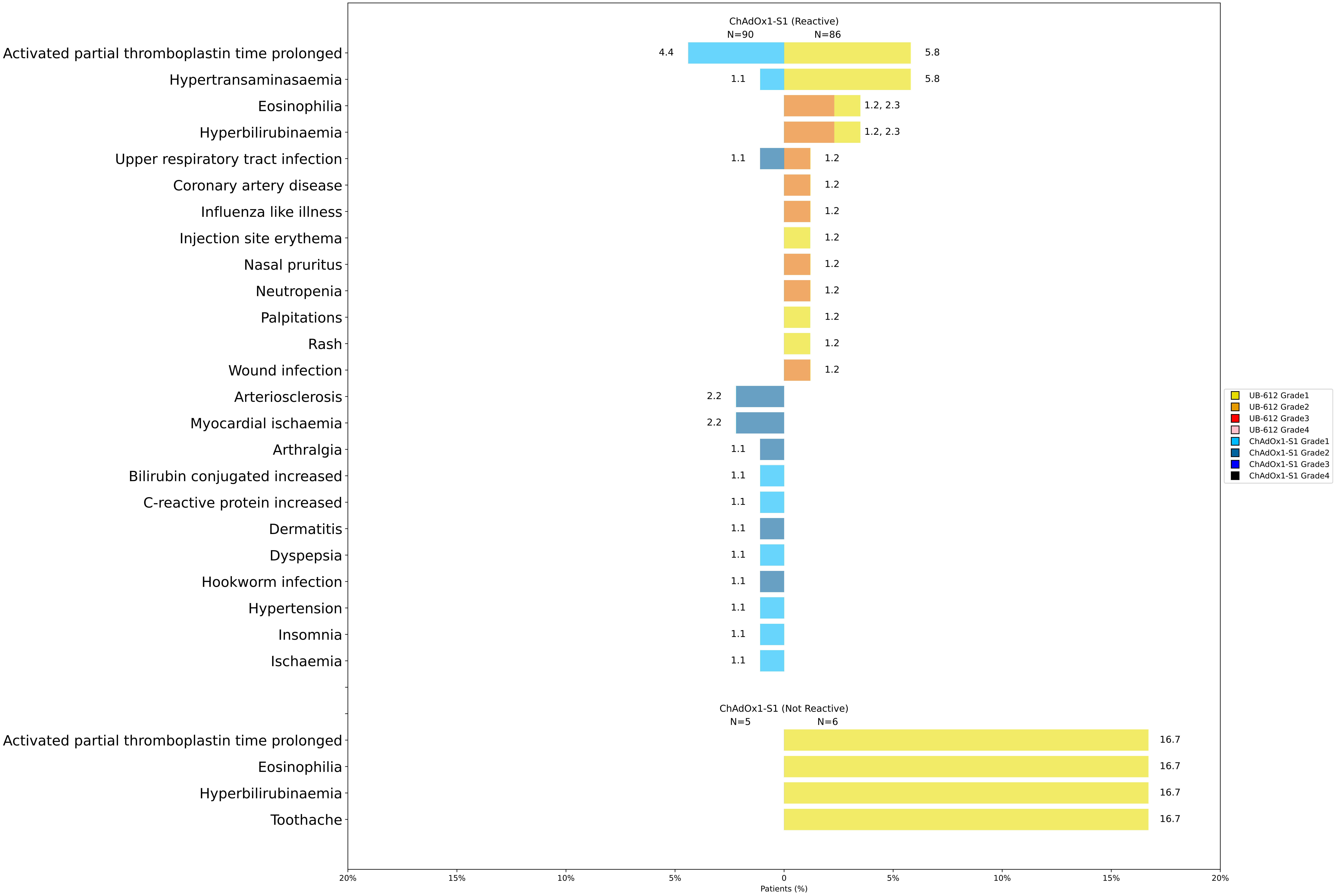

Supplemental figure 8B. ChAdOx1-S substudy subgroup TEAE (excluding terms matching solicited AEs) by N-protein seropositivity.

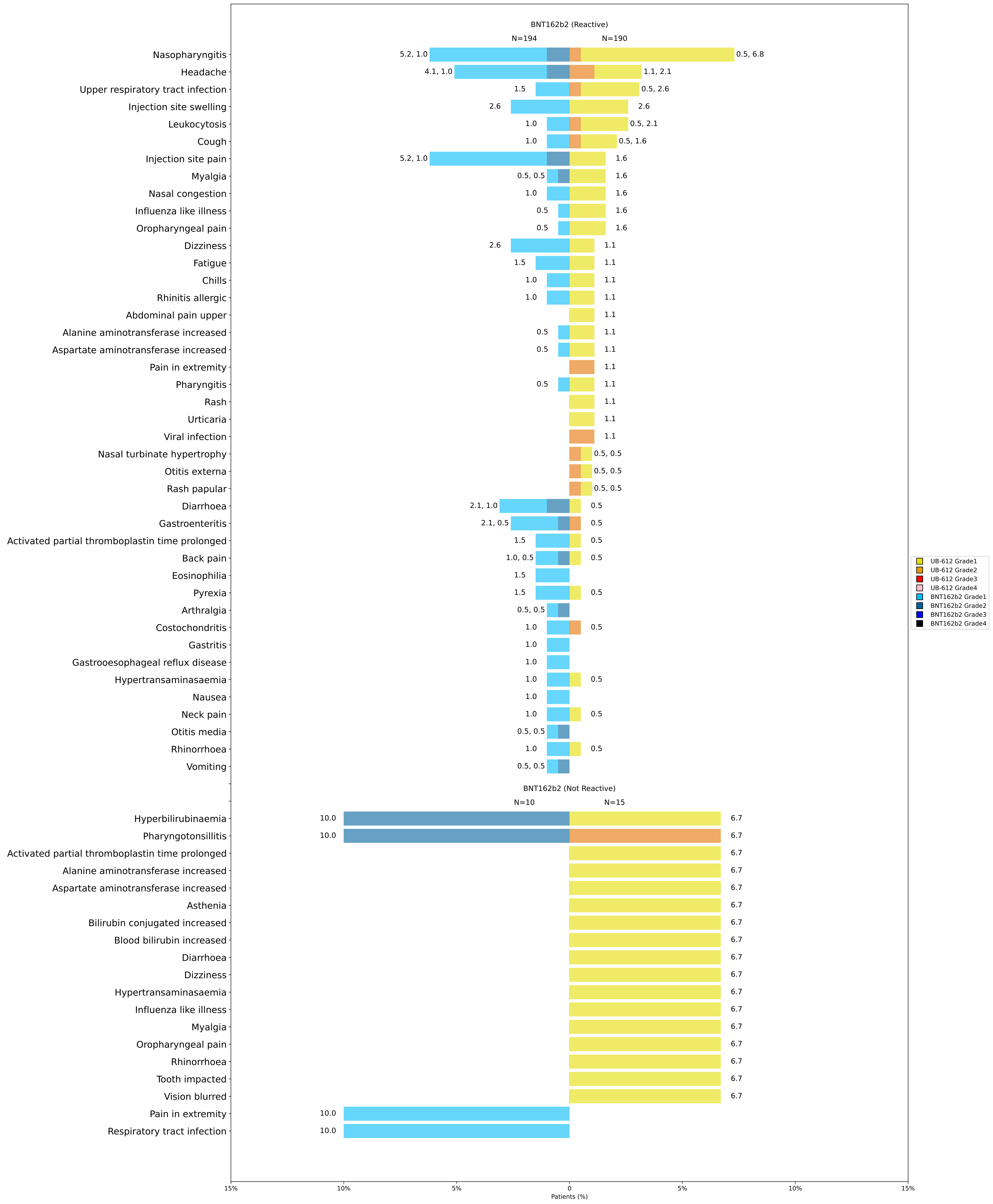

Supplemental figure 8C. BNT162b2 substudy subgroup TEAE (excluding terms matching solicited AEs) by N-protein seropositivity.

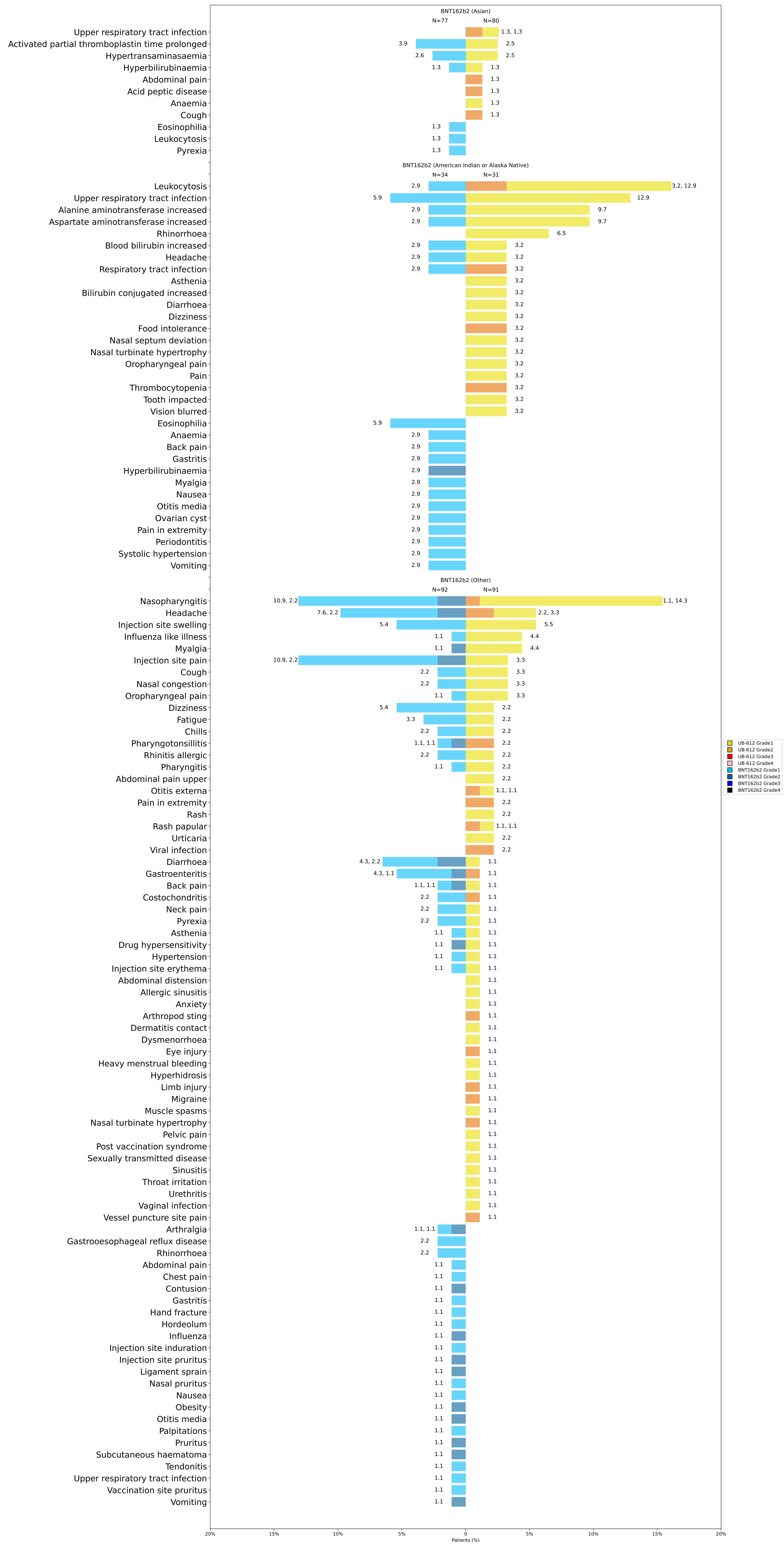

Supplemental figure 8D. BBNT162b2 substudy subgroup TEAE (excluding terms matching solicited AEs) by race

Supplemental analysis: Modeling of neutralizing antibody responses in the elderly subjects after a booster immunization with UB-612.

In the previous Phase 2 study, UB-612 was shown to be immunogenic in adults (18-64 years old) and the elderly (65 and over) subjects when delivered as a third-dose homologous booster; however, in the pivotal Phase 3 study, there was limited enrollment of elderly subjects. Vaxxinity used modeling trained on Phase 2 study results to predict the antibody responses in the elderly subjects after heterologous booster with UB-612. To validate the forecasted antibody responses, Vaxxinity compared UB-612 modeling results to reported BNT162b2 booster studies.

Methodology

For the purpose of modeling, we used the three-dose data from Phase 2, V-205 [1]. A linear model was trained on combined Wuhan live virus neutralization data from Phase 2 and Phase 3, UB-612-305, using Age, Sex, BMI, Country, and Trial as features. All modeling and analyses were performed in Python, using the sci-kit learn and scipy packages [2,3]. Approximations of neutralizing antibody titer from published BNT162b2 trials were made by assuming linearity with age and fitting to the geometric mean titers provided [4–6].

Results

In the Phase 2 study testing UB-612 third-dose homologous booster, the GMT ratio of Wuhan neutralizing antibodies of elderly subjects (65 years and over) years to adults (18-64 years) was 0.70. Statistical analysis determined that the p-value resulting from a Mann-Whitney-U statistical test of these two distributions was 0.11, a nonsignificant value. To further investigate, the adults' group was further subdivided into two subgroups: 18-34 years of age (Young) and 35-64 years of age (Mid).

Table 1: Geometric mean titer of Wuhan neutralizing antibodies after UB-612 homologous third-dose booster vaccination, V-205 data

Age group	Number of subjects	GMT (95% CI)
18-34 (Young)	98	999 (847, 1179)
35-64 (Mid)	124	652 (546, 779)
65+ (Elderly)	97	551 (409, 743)

Table 2: Geometric mean ratio of Wuhan neutralizing antibody levels after UB-612 homologous third-dose booster vaccination, V-205 data

Compared groups	GMT ratio	p-value
Mid / Young	0.65	0.003
Elderly / Mid	0.84	0.723
Elderly / Young	0.55	0.008

According to the refined age grouping in Phase 2, the majority of the age-related decline in antibody levels occurred before the age of 35, and the antibody levels of the middle aged group are not statistically different from those of the elderly group. The number of subjects in each age group as well as Mann-Whitney-U p-values for comparisons between adjacent groups are shown in Table 1 and 2.

We trained a model to predict neutralizing antibody levels for the elderly population in Phase 3, with combined Phase 2 and Phase 3 trial data. The model was trained first on only the 35 and older population and then on adults (18 years and older) population (Figure 2).

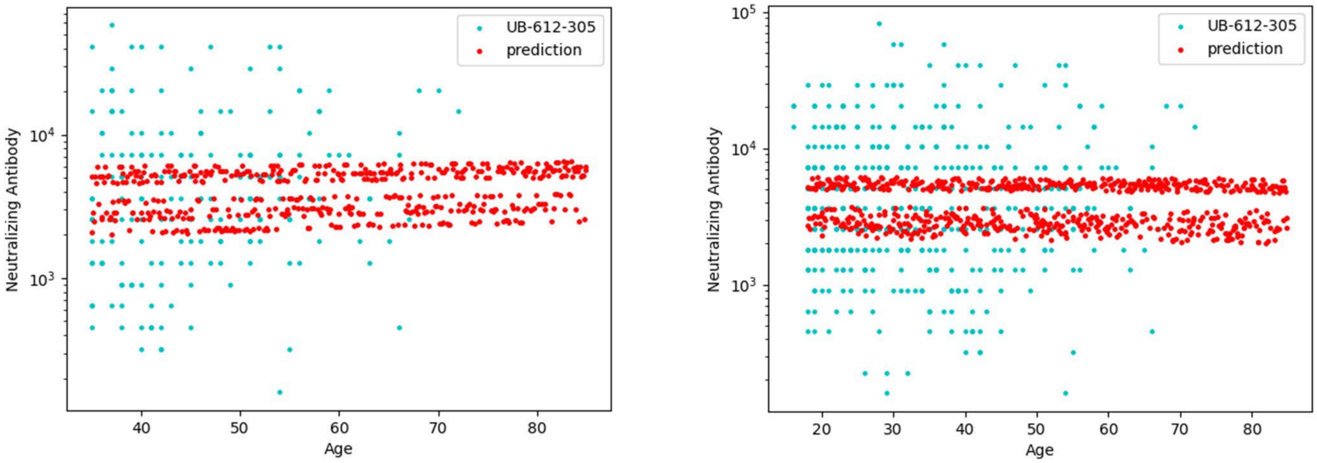

Figure 2: Wuhan neutralizing antibody data after third vaccination from Phase 3 (blue) and model predictions based on simulated data (red). The model trained in the 35 and older population (left) and the adults (18 and older) population (right).

The GMT ratio of Elderly / Mid for the first model (trained on only subjects 35 and older) was 1.11, indicating no statistical age-related decline, as predicted. The GMT ratio of Elderly / Young for the second model (trained on all subjects 18+) was 0.98, also reflecting no statistical age-related decline.

When varying the cutoff age, the analysis of the Phase 2 trial data produces fairly stable Wuhan neutralizing antibody response results for the Mid / Young GMT ratio, ranging from 0.64 to 0.75 (Figure 3). For the Elderly / Mid GMT ratio, increasing the cutoff age generally increases the GMT ratio, which ranges from 0.73 to 0.91. This is consistent with our conclusion that the majority of the decrease in neutralizing antibody level with age occurs between the 18-34 and 35-64 adult age range, rather than between the 35-64 and 65 and older age range, regardless of where the exact cutoff age for these age groups is set. The Phase 3 trial data does not show a statistically significant change in neutralizing antibody level with age, and the GMT ratio here ranges from 0.93 to 1.07.

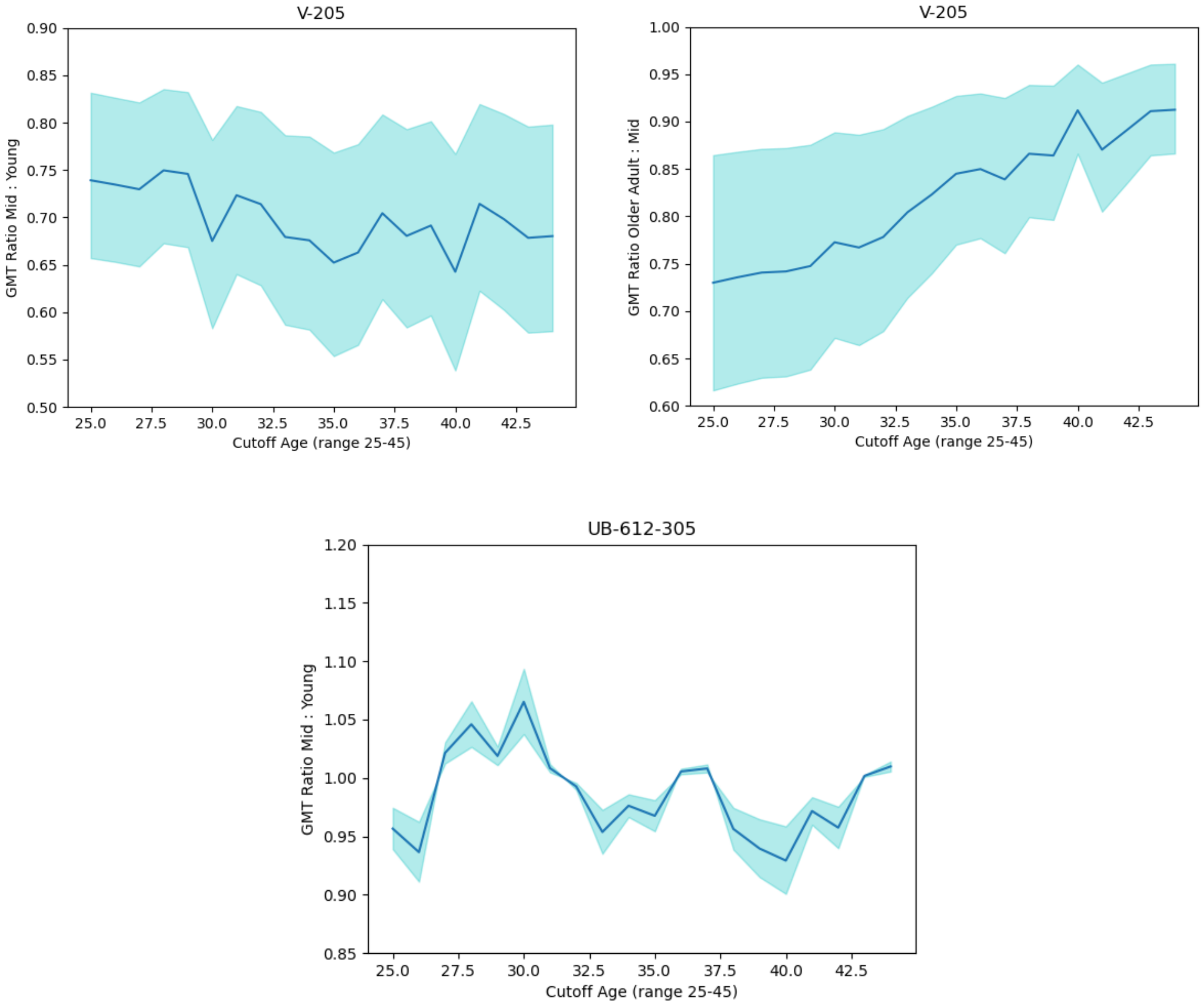

Figure 3: The analyses of Wuhan neutralizing antibody response using varying cut-off age. GMT ratio, including 95% confidence interval, plotted against cutoff age for the Phase 2 (Mid / Young top left, Elderly / Mid top right) and the Phase 3 study (bottom).

To validate the forecasted age dependent immune responses, the model trained on UB-612 third-dose immunization study results was applied to evaluate reported immune responses after homologous third-dose boosting with mRNA vaccine BNT162b2.

In Falsey et al, 2021, Wuhan neutralizing antibody responses ranging from 7 days to 1 month after dose 3 were included in the analysis with fitted neutralizing antibody data shown in Figure 4. There was no significant difference between BNT162b2 and UB-612 responses post dose 3. It should also be noted that the sample size for the mRNA study was extremely small (n=23) and so a high variance of these values would be expected (actual variance was not reported).

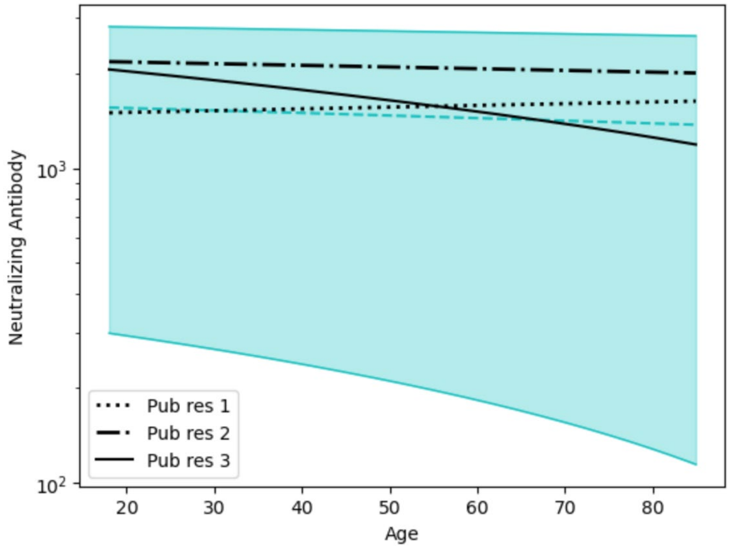

Figure 4: Comparison of reported in Falsey, 2021, and forecasted age dependent Wuhan neutralizing antibody responses after homologous booster with BNT162b2. UB-612 model prediction range (cyan). Minima and maxima shown in solid lines with mean prediction per age shown as dashed line. Black lines indicate fitted mean neutralizing antibody titer based on data extracted from Falsey et al, 2021. Shown are 1 month after dose 3 of BNT162b2 (Figure 1 panel B, Pub res 1, and panel B, Pub res 2), and 7 days after does 3 of BNT162b2 (Figure 1 panel B, Pub res 3).

In Atmar et al, 2022, the groups had a larger sample size (n=48), and 95% confidence intervals for geometric mean titer values were provided. These values were used to fit neutralizing antibody data both for 14 days and 28 days post dose 3 (Figure 5). The BNT162b2 neutralizing antibody titer values were found within (or below) the range predicted by the UB-612 model.

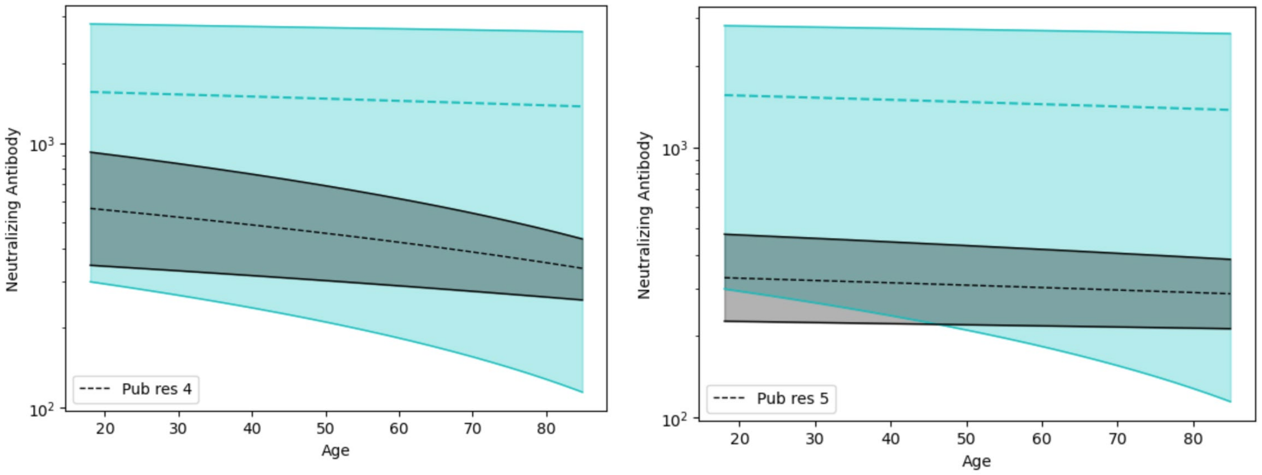

Figure 5: Comparison of reported in Atmar, 2022, and forecasted age dependent Wuhan neutralizing antibody responses after homologous booster with BNT162b2. UB-612 model prediction range (cyan). Minima and maxima shown in solid lines with mean prediction per age shown as dashed line. Black lines indicate fitted mean neutralizing antibody titer based on data extracted from the reference publication; mean values are shown with black dashed line, with shaded region indicating 95% confidence interval. Shown are data from 14 days after dose 3 of BNT162b2 (Pub res 4, left), and 28 days after dose 3 of BNT162b2 (Pub res 5, right).

In Tut et al., 2023, the relevant results described in Figure 1a of the reference article indicate no significant difference in BNT162b2 vaccine response in the <65 population compared to the >65 population for patients with a prior infection post dose 3. This is in line with the findings reported previously for UB-612, and so we determined that no additional analysis is necessary.

Conclusion

The modeling results demonstrated that there is no significant age-related decline in the 65 and over age group after received a third-dose of UB-612. Although there seems to be a significant drop in neutralizing antibodies after the age of 35 compared to the level seen in the younger age category of 18-34 years, it has a minimum impact on the modeling results. Therefore, it is reasonable to conclude, based on the modeling, that there is no significant decline in the neutralizing response of the elderly population for UB- 612. The published results of BNT162b2 vaccine response post dose 3 all fall within the prediction range of the UB-612 model, implying no significant difference in performance between the two vaccines for any age.

References:

1 Wang CY, Hwang K-P, Kuo H-K, *et al.* A multitope SARS-CoV-2 vaccine provides long-lasting B cell and T cell immunity against Delta and Omicron variants. *J Clin Invest* 2022; **132**. DOI:10.1172/JCI157707.

2 Virtanen P, Gommers R, Oliphant TE, *et al.* SciPy 1.0: fundamental algorithms for scientific computing in Python. *Nat Methods* 2020; **17**: 261–72.

3 Pedregosa F, et al. Scikit-learn: Machine learning in Python. *Journal of machine Learning research* 2011; **12**: 2825–30.

4 Falsey AR, Frenck RW, Walsh EE, *et al.* SARS-CoV-2 Neutralization with BNT162b2 Vaccine Dose 3. *New England Journal of Medicine* 2021; **385**: 1627–9.

5 Atmar RL, Lyke KE, Deming ME, *et al.* Homologous and Heterologous Covid-19 Booster Vaccinations. *New England Journal of Medicine* 2022; **386**: 1046–57.

6 Tut G, Lancaster T, Krutikov M, *et al.* Strong peak immunogenicity but rapid antibody waning following third vaccine dose in older residents of care homes. *Nat Aging* 2023; **3**: 93–104.

Supplementary Materials and Methods:

Cell culture

Vero E6 cells (American Type Culture Collection [ATCC] #CRL-1586) were cultured in Dulbecco's Modified Eagle's Medium (DMEM) high glucose supplemented with 2 mM L-Glutamine, 100 U/mL of penicillin- streptomycin mixture (complete DMEM) and 10% Fetal Bovine Serum (FBS). Cells were maintained in humified atmosphere at 37°C, 5% CO_2 and passaged every 3-4 days. The day prior to the execution of the microneutralization assay, Vero E6 cells diluted in complete DMEM supplemented with 2% FBS were added to 96-well plates ($1.5x10^4$ cell/well) and incubated at 37°C, 5% CO_2 until use.

Live Viruses

Authentic, sequence-verified wild type SARS CoV-2 stock (SARS-CoV-2 hCoV-19/Australia/VIC01/2020, also referred to as “Wuhan”) was kindly provided by CEPI. SARS-CoV-2 Omicron BA.5 as well as Omicron XBB.1.5 were kindly provided by Rega Institute Leuven, Belgium. All the strains were propagated in VisMederi Research s.r.l. (Siena, Italy) as described elsewhere **[1]**. The propagated viruses were titrated in 96-well plates containing a sub-confluent Vero E6 cells monolayer. 10-fold serial dilution of virus (10^{-1} to 10^{-11}) were incubated with the cells and checked daily for signs of Cytopathic Effect (CPE) for a total of 3 days (Wuhan) or 4 days (Omicron variants). The viral titer was calculated using the 50% tissue culture infectious dose per mL (TCID50/mL) as endpoint and defined as the reciprocal of the highest virus dilution yielding at least 50% CPE in the inoculated wells, according to the Reed and Muench formula **[2]**.

SARS-CoV-2 live-virus neutralization assay

SARS-CoV-2 live-virus neutralization was performed via a CPE-based microneutralization assay. Briefly, ten 2-fold serial dilutions of serum samples (starting dilution: 1:20) were prepared in duplicate in DMEM 2% FBS and added to two different 96-well plates. The plates were then incubated for 1 h at 37°C, 5% CO_2 with 25 TCID50 of authentic SARS-CoV-2 Wuhan or Omicron variants (serum-virus ratio 1:1) to allow binding of antigen-specific antibodies to the virus. The virus-serum mixture was then added to sub-confluent Vero E6 cells to allow their infection from the unbound virus. After 3 days (Wuhan) or 4 days (Omicron variants) cells were inspected for signs of CPE under an inverted light microscope. The reciprocal of the highest sample dilution able to protect at least 50% of the cells from CPE was regarded as the neutralization titer and reported as Geometric Mean Titer (GMT) (mean of two replicates). In absence of neutralization, an arbitrary titer value of 10 (half of the limit of detection – LOD -) was reported.

S1-RBD ELISA IgG assay

Specific anti-SARS-CoV-2 Receptor Binding Domain (RBD) IgG antibodies were detected through an in-house ELISA test as described previously **[3]** with minor modifications. Briefly, 96-well plates were coated with 1 µg/mL Spike-RBD-His Recombinant Protein (code 40592-V08H) (Sino Biological, China), diluted in ELISA coating buffer (0.05 M carbonate-bicarbonate solution, pH 9.6), and incubated overnight at 4°C. The plates were then washed three times with 300 µl/well of ELISA washing buffer (50mM Tris Buffered Saline (TBS) pH 8.0, 0.05% Tween-20) and saturated with 200 µl/well of ELISA blocking buffer (TBS-0.05% Tween 20containing 5% of Non-Fat Dry Milk (NFDM); Euroclone, Pero, Italy) for 1 hour at 37°C. After further 3x washing, serum samples were two-fold serially diluted (starting dilution: 1:800) in ELISA diluent buffer (TBS-0.05% Tween 20 5% NFDM) and added to the ELISA plate (100 µL/well). After 1 h incubation at 37◦C, plates were washed as previously, and 100 µL/well of Goat anti-Human IgG-Fc Horse Radish Peroxidase (HRP)-conjugated antibody diluted 1:100000 (code A80-104P) (Bethyl Laboratories, Montgomery USA) were added. Plates were incubated at 37° C for 30 min, washed, and filled with 100 µL/well of 3,3 ′ µ ,5,5 ′-Tetramethylbenzidine (TMB) substrate (Bethyl Laboratories, Montgomery, USA). Following a 20-minute incubation in the dark at room temperature, the reaction was stopped by adding 100 µL/well of ELISA stop solution (0,5M HCl) (Fischer Scientific/Fischer Chemical). The Optical Density (OD) at 450 nm (OD_{450}) was read within 30 min via a SpectraMax ELISA plate reader (Medical Device). A cut-off value was defined at OD = 0.311 (Cut Off = average of blank samples

at OD_{450} (Limit of Blank OD_{450}) + 3 Standard Deviations + Matrix Effect)). Samples showing OD under the cut off value at dilution 1:800 dilution were assigned as negative, otherwise as positive. The antibody titer was calculated by interpolation based on the pre-determined cut-off.

N PROTEIN ELISA IgG assay

Anti-SARS-CoV-2-specific N protein IgG antibodies were assessed via an in-house ELISA assay. 96-well plates were coated with 1 µg/mL SARS-CoV-2 Nucleocapsid-His recombinant Protein (cod. 40588-V08B) (Sino Biological, China) resuspended in ELISA coating buffer, and incubated overnight at 4°C. The plates were then washed three times with 300 µl/well of ELISA washing buffer, blocked with 200 µl/well of ELISA blocking buffer for 1 hour at 37°C, and washed 3x as previously. Serum samples were two-fold serially diluted (starting dilution: 1:200) in ELISA diluent buffer, added to the plate (100 µL/well) and incubated for 1 h incubation at 37◦C. The subsequent steps were carried out as done for the S1-RBD ELISA IgG assay. For the ELISA N protein assay, the cut-off value was defined as 3 times the mean of blank. Samples showing OD under the cut off value at dilution 1:200 dilution were assigned as negative, otherwise as positive. The antibody titer was calculated by interpolation based on the pre-determined cut-off.

ELECSYS ANTI-SARS-CoV-2 N protein antibodies detection ON COBAS e411

Immunoglobulin levels towards the N protein of SARS-CoV-2 were evaluated by using the Elecsys® enzyme immunoassay (Roche Diagnostic International Ltd) as previously done **[4].** The assay was performed with the Cobas e411 (Roche Diagnostic International Ltd) platform, an automated system for immunological analysis that uses electrochemiluminescence technology. Briefly, 20 µl of serum sample, biotinilated SARS-CoV-2-specific recombinant N protein and SARS-COV-2-specific recombinant N protein labelled with a ruthenium complex were incubated to form a sandwich complex; afterwards, addition of streptavidin-coated microparticles leads the complex become bound to the solid phase via interaction of biotin and streptavidin. The reaction mixture was aspirated into the measuring cell where the microparticles are magnetically captured on the surface of the electrode. Unbound substances were then removed with ProCell reagent. A voltage was applied at the electrode that induces chemiluminescent emission which was measured by a photomultiplier. Results were determined automatically by the instrument software. The electrochemiluminescence signal obtained from the reaction product of the sample is compared with the signal of the cut-off value previously obtained by calibration. The result of a sample is given either as "reactive" or "non-reactive" as well as in the form of a cut-off index (COI; signal sample/cut-off).

ADCP Assay

THP-1 cells were purchased from ATCC and SARS-CoV2 S Protein RBD-His Tag from Acro Biosystems. High-, Medium-, and Low-activity human serum screened for controls from previously infected individuals; negative control were serum from seronegative individuals collected before 2019. Biotinylated antigen was incubated with 1 µm fluorescent neutravidin beads (Invitrogen, F8776) overnight at 4°C. Beads were placed in each well of round bottom 96 well plate. Antibodies were added to each well and the plate was incubated for a 2 hours at 37°C. Following equilibration, 2×10^4 THP-1 cells were added to each well in a final volume of 200 µl, and the plate was incubated overnight. Cells were treated with 100 µl of 4% paraformaldehyde and analyzed by flow cytometry on a BD LSR II yielding at least 2,000 cell events per sample. A phagocytic score was determined by gating the samples on events representing cells, and calculated as follows: (% bead positive × mean fluorescence intensity (MFI) bead positive, or integrated MFI **[5]**.

References:

1 Manenti A, et al. Manenti A, Maggetti M, Casa E, Martinuzzi D, Torelli A, Trombetta CM, Marchi S, Montomoli E. Evaluation of SARS-CoV-2 neutralizing

antibodies using a CPE-based colorimetric live virus micro-neutralization assay in human serum samples. J Med Virol. 2020 Oct;92(10):2096-2104. doi: 10.1002/jmv.25986. Epub 2020 May 17. PMID: 32383254; PMCID: PMC7267461.

2 L.J. REED, H. MUENCH, A SIMPLE METHOD OF ESTIMATING FIFTY PER CENT ENDPOINTS, American Journal of Epidemiology, Volume 27, Issue 3, May 1938, Pages 493–497, https://doi.org/10.1093/oxfordjournals.aje.a118408

3 Mazzini L, Martinuzzi D, Hyseni I, Benincasa L, Molesti E, Casa E, Lapini G, Piu P, Trombetta CM, Marchi S, Razzano I, Manenti A, Montomoli E. Comparative analyses of SARS-CoV-2 binding (IgG, IgM, IgA) and neutralizing antibodies from human serum samples. J Immunol Methods. 2021 Feb;489:112937. doi: 10.1016/j.jim.2020.112937. Epub 2020 Nov 28. PMID: 33253698; PMCID: PMC7695554.

4 Manenti A, Gianchecchi E, Dapporto F, Leonardi M, Cantaloni P, Fattorini F, Piu P, Bollati V, Pastorino U, Apolone G, Sozzi G, Montomoli E. Evaluation and correlation between SARS-CoV-2 neutralizing and binding antibodies in convalescent and vaccinated subjects. J Immunol Methods. 2022 Jan;500:113197. doi: 10.1016/j.jim.2021.113197. Epub 2021 Nov 26. PMID: 34843712; PMCID: PMC8619878.

5 Ackerman ME, Moldt B, Wyatt RT, Dugast AS, McAndrew E, Tsoukas S, Jost S, Berger CT, Sciaranghella G, Liu Q, Irvine DJ, Burton DR, Alter G. A robust, high-throughput assay to determine the phagocytic activity of clinical antibody samples. J Immunol Methods. 2011 Mar 7;366(1-2):8-19. doi: 10.1016/j.jim.2010.12.016. Epub 2010 Dec 27. Erratum in: J Immunol Methods. 2012 Feb 28;376(1-2):156. PMID: 21192942; PMCID: PMC3050993.

Sudden cardiac death narrative.

A 51-year-old Asian male participated in Study UB-612-305, a Phase 3 trial comparing COVID-19 vaccine boosters, starting on 09SEP2022. He received two Sinopharm BIBP vaccines in August and September 2021. Following randomization, he received a booster on 09SEP2022.

At screening on 08SEP2022, the subject reported a history of pulmonary tuberculosis (diagnosed in 2020, resolved by 2022) treated with not-specified medications. Screening tests showed high C-reactive protein (10 mg/L; reference range 0-0.99 mg/L), but the investigator deemed it not clinically significant. C-reactive protein values on Day 15 and Day 29 were both within the normal reference range. All other screening laboratory tests were within the normal range. There were no physical exam findings noted. Abnormal ECG, QTcB interval, 453 single beat (msec), was identified but considered not clinically significant by the investigator.

Laboratory test results outside the normal reference range on Day 15 or Day 29 were all evaluated as not clinically significant by the investigator. These included: on Day 15: eosinophils (high) (0.91 10^9/L, reference range 0.02-0.5), Lymphocytes/leukocytes ratio (low) (0.19, reference range 0.22-0.4), eosinophil/leukocyte ratio (high) (0.11, reference range 0.01-0.04), activated partial thromboplastin time (prolonged) 38.60 sec, reference range 28.9-38.1); and on Day 29: aspartate aminotransferase (low) 9.90 U/L, reference range 15-37, activated partial thromboplastin time (prolonged) 39.80 sec, reference range 28.9-38.1). No treatment was given

It was reported that the subject was well and had good functional capacity until three weeks prior to the event, when he began experiencing progressive exertional dyspnea. He self-medicated with nebulized salbutamol (1 mg/mL, 1 nebule four times a day) starting in December 2022, which offered temporary relief of symptoms.

On 18JAN2023, the subject died of sudden cardiac death, one hour after reporting chest pain, dyspnea, diaphoresis, and palpitations. The investigator concluded the death was unrelated to the study and caused by heart failure and arrhythmia. No autopsy was performed, and post-mortem SARS-CoV-2 testing was negative. The subject was discontinued from the study on 18JAN2023.

Medical history	Start date	End date
ulmonary tuberculosis	2020	2022

Prior medication	Dosage/ Frequency	Indication	Start date/ Study Day	End date/ Study Day
Sinopharm BIBP SARS-CoV-2 Vaccine	0.5 mL / ONCE	SARS-CoV-2	2021-09-16 / -358	2021-09-16 / -358
Sinopharm BIBP SARS-CoV-2 Vaccine	0.5 mL / ONCE	SARS-CoV-2	2021-08-19 / -386	2021-08-19 / -386

Concomitant medication	Dosage/ Frequency	Indication	Start date/ Study Day	End date/ Study Day
The subject did not take any concomitant medication				

Lab test	Visit day	Value	Reference range	Clinically Significant Y/N
Platelets (10^9/L)	Screening	443	150 - 450	
	Day 15	315	150 - 450	
	Day 29	307	150 - 450	
Hemoglobin (g/L)	Screening	163	135 - 180	
	Day 15	164	135 - 180	
	Day 29	158	135 - 180	
Hematocrit (RATIO)	Screening	0.48	0.4 - 0.54	
	Day 15	0.48	0.4 - 0.54	
	Day 29	0.47	0.4 - 0.54	
Erythrocytes (10^12/L)	Screening	5.56	4.2 - 6	
	Day 15	5.62	4.2 - 6	
	Day 29	5.53	4.2 - 6	
Leukocytes (10^9/L)	Screening	10.31	4.5 - 11	
	Day 15	8.64	4.5 - 11	
	Day 29	7.46	4.5 - 11	
Neutrophils (10^9/L)	Screening	6.49	2 - 7	
	Day 15	5.61	2 - 7	
	Day 29	5.02	2 - 7	
Lymphocytes (10^9/L)	Screening	2.63	0.8 - 4	
	Day 15	1.61	0.8 - 4	
	Day 29	1.75	0.8 - 4	
Monocytes (10^9/L)	Screening	0.72	0.12 - 1.2	
	Day 15	0.46	0.12 - 1.2	
	Day 29	0.35	0.12 - 1.2	
Eosinophils (10^9/L)	Screening	0.41	0.02 - 0.5	
	Day 15	0.91 H	0.02 - 0.5	N
	Day 29	0.31	0.02 - 0.5	
Basophils (10^9/L)	Screening	0.06	0 - 0.1	
	Day 15	0.05	0 - 0.1	

Lab test	Visit day	Value	Reference range	Clinically Significant Y/N
	Day 29	0.03	0 - 0.1	
Neutrophils/Leukocytes (%)	Screening	63	50 - 70	
	Day 15	65	50 - 70	
	Day 29	67	50 - 70	
Lymphocytes/Leukocytes (%)	Screening	26	22 - 40	
	Day 15	19 L	22 - 40	N
	Day 29	24	22 - 40	
Monocytes/Leukocytes (%)	Screening	7	3 - 8	
	Day 15	5	3 - 8	
	Day 29	5	3 - 8	
Eosinophils/Leukocytes (%)	Screening	4	1 - 4	
	Day 15	11 H	1 - 4	N
	Day 29	4	1 - 4	
Basophils/Leukocytes (%)	Screening	1	0 - 1	
	Day 15	0	0 - 1	
	Day 29	0	0 - 1	
Aspartate Aminotransferase (U/L)	Screening	16.5	15 - 37	
	Day 15	18.2	15 - 37	
	Day 29	9.9 L	15 - 37	N
Alanine Aminotransferase (U/L)	Screening	36	16 - 63	
	Day 15	40	16 - 63	
	Day 29	28	16 - 63	
Bilirubin (umol/L)	Screening	5.61	3 - 17	
	Day 15	10.45	3 - 17	
	Day 29	8.76	3 - 17	
Direct Bilirubin (umol/L)	Screening	1.78	0 - 3	
	Day 15	2.19	0 - 3	
	Day 29	2.59	0 - 3	
Creatinine (umol/L)	Screening	104.99	49 - 115	

Lab test	Visit day	Value	Reference range	Clinically Significant Y/N
	Day 15	104.29	49 - 115	
	Day 29	102.72	49 - 115	
C Reactive Protein (mg/L)	Day 1	10 H	0 - 0.99	N
	Day 15	2.25	0 - 3	
	Day 29	1.6	0 - 3	
Partial Thromboplastin Time (sec)	Screening	35.2	28.9 - 38.1	
	Day 15	38.6 H	28.9 - 38.1	N
	Day 29	39.8 H	28.9 - 38.1	N
Prothrombin Time (sec)	Screening	13	11.5 - 15.5	
	Day 15	14.1	11.5 - 15.5	
	Day 29	13.4	11.5 - 15.5	
Prothrombin Intl. Normalized Ratio (RATIO)	Screening	1	0 - 1.209	
	Day 15	1.09	0 - 1.209	
	Day 29	1.03	0 - 1.209	
Hemoglobin A1C (mmol/mol)	Screening	36.615	-23.5 - 38.692	
