# Supplementary material for: Safety and immunogenicity of UB-612 heterologous booster in adults primed with mRNA, adenovirus, or inactivated COVID-19 vaccines: a randomized, active-controlled, Phase 3 trial
Source: eClinicalMedicine. 2025 Jul 21;86:103349. doi: 10.1016/j.eclinm.2025.103349 (PMC12301762; doi:10.1016/j.eclinm.2025.103349)
Supplement: Vaxxinity_UB-612-305_SAP [file mmc3.pdf]

Study UB-612-305

A Phase 3 Multi-Center International, Randomized, Active-Controlled Platform Trial to Compare Homologous Boost of Authorized COVID-19 Vaccines and Heterologous Boost with UB-612 Vaccine

Statistical Analysis Plan

Date: 17 October 2022

Version 2.0

Protocol Version: 10.0 dated on 4Oct2022

SIGNATURE PAGE

I have reviewed this Statistical Analysis Plan and approve its contents.

Kate Wang, MS — Date
Author
Senior Director, Biostatistics, AvanSight, Inc.

DocuSigned by:
Signer Name: Tanya Granston
Signing Reason: I approve this document
Signing Time: 18-Oct-2022 | 09:04:42 EDT
CB1FC9DB0B954BD3824B61B37B73BCBA

18-Oct-2022 | 09:04:47 EDT

Tanya Granston, PhD — Date
Senior Director, Biostatistics, Vaxxinity, Inc.

DocuSigned by:
Signer Name: Lixia Wang
Signing Reason: I approve this document
Signing Time: 19-Oct-2022 | 09:39:33 PDT
775AC1C8E14445F7B7EE30A3840209DC

19-Oct-2022 | 09:39:40 PDT

Lixia Wang, PhD — Date
Senior VP, Data Science, Vaxxinity, Inc.

DocuSigned by:
Signer Name: Sasha Rumyantsev
Signing Reason: I have reviewed this document
Signing Time: 18-Oct-2022 | 06:04:29 PDT
2F2[illegible]93A4639B47649C8AA18A68E12FD0

18-Oct-2022 | 06:04:36 PDT

Sasha Rumyantsev, MD, PhD, MBA — Date
Therapeutic Area Head, Infectious Diseases, Vaxxinity, Inc.

DocuSigned by:
Signer Name: Ulo Palm
Signing Reason: I approve this document
Signing Time: 19-Oct-2022 | 17:34:53 PDT
[illegible]

19-Oct-2022 | 17:34:56 PDT

Ulo Palm, MD, PhD, MBA — Date
Chief Medical Officer, Vaxxinity, Inc.

TABLE OF CONTENTS

1 INTRODUCTION 7
1.1 Study Objectives 7
1.2 Study Design 9
2 STATISTICAL HYPOTHESES 11
2.1 Multiplicity Adjustment 12
3 ANALYSIS SETS 13
4 STATISTICAL ANALYSES 14
4.1 General Consideration 14
4.2 Primary Immunogenicity Endpoints Analyses 14
4.2.1 Geometric Mean Titer (GMT) 14
4.2.2 Main Analytical Approach 14
4.3 Secondary Immunogenicity Endpoints Analyses 15
4.3.1 Secondary Endpoints 15
4.4 Exploratory Immunogenicity Endpoints and Analyses 16
4.5 Pooling Efficacy Data across Sub-studies 17
4.6 Safety Analyses 17
4.6.1 Extent of Exposure 17
4.6.2 Adverse Events 17
4.6.2.1 Primary Safety Endpoints 17
4.6.2.2 Treatment-Emergent Adverse Events 18
4.6.2.3 Exploratory Analysis for Solicited Adverse Events 18
4.6.2.4 Exploratory Analysis of Standardised MedDRA Queries (SMQs) Relating to Myocarditis or Pericarditis 18
4.6.2.5 Laboratory Data 18
4.6.3 Other Safety Parameters 19
4.6.3.1 Vital Signs 19
4.6.3.2 Height and Weight 19
4.6.3.3 Physical and Neurological Examination 19
4.6.3.4 Electrocardiogram (ECG) 19
4.6.3.5 Pregnancy Test 19
4.6.3.6 Antigen Test 19

4.7 Other Analyses ... 20
4.7.1 Study Subjects, Demographics and other Baseline Characteristics ... 20
4.7.1.1 Disposition of Subjects ... 20
4.7.1.2 Protocol Deviation ... 20
4.7.1.3 Demographics and Other Baseline Characteristics ... 20
4.7.1.4 Medical History ... 21
4.7.1.5 Prior and Concomitant Medications or Procedures ... 21
4.7.2 Subgroup Analysis ... 21
4.8 Interim Analysis ... 21
4.9 Changes to Protocol-planned Analyses ... 21
5 SAMPLE SIZE DETERMINATION ... 22
5.1 BNT162b2 Sub-study ... 22
5.2 ChAdOx1-S Sub-study ... 22
5.3 BIBP Sub-study ... 22
6 REFERENCES ... 23
7 VERSION HISTORY ... 24

LIST OF TABLES

Table 1: Objectives and Endpoints ... 7

LIST OF FIGURES

Figure 1: Trial Design Flow Chart ... 10

LIST OF ABBREVIATIONS

Abbreviation	Description
ADCP	Antibody Dependent Cell-mediated Phagocytosis
AE	Adverse Event
AESI	Adverse Event of Special Interest
ATC	Anatomical Therapeutic Chemical
AUC	Area Under the Curve
BMI	Body Mass index
CI	Confidence Interval
COVID-19	Coronavirus Disease 2019
CRF	Case Report Form
CSR	Clinical Study Report
CTMS	Clinical Trial Management System
DMC	Data Monitoring Committee
ECG	Electrocardiogram
ELISA	Enzyme-linked Immunosorbent Assay
ELISpot	Enzyme-linked Immunosorbent Spot
GCP	Good Clinical Practice
GMFI	Geometric Mean Fold Increase
GMR	Geometric Mean Ratio
GMT	Geometric Mean Titer
HCV	Hepatitis C Virus
HIV	Human Immunodeficiency Virus
ICF	Informed Consent Form
ICH	International Conference on Harmonization
ICS	Intracellular Cytokine Staining
IFN	Interferon
IgG	Immunoglobulin G
IL	Interleukin
IM	Intramuscular
ITT	Intent-to-Treat
LLoQ	Lower Limit of Quantification

**Abbreviation**	**Description**
MAAE	Medically Attended Adverse Event
Max	Maximum
MedDRA	Medical Dictionary for Regulatory Activities
Min	Minimum
MITT	Modified Intent-to-Treat
N/A	Not Applicable
NA	Not Applicable
NHS	National Health Service
PBMC	Peripheral Blood Mononuclear Cell
PP	Patient Privacy
PPS	Per Protocol Set
PT	Preferred Term
QC	Quality Control
QTc	Corrected QT Interval
RBD	Receptor Binding Domain
RT-PCR	Reverse Transcriptase-Polymerase Chain Reaction
SAE	Serious Adverse Event
SAP	Statistical Analysis Plan
SD	Standard Deviation
SI	Standard International System of Units
SOC	System Organ Class
SOP	Standard Operating Procedure
SS	Safety Set
TEAE	Treatment-Emergent Adverse Event
TFL	Table, Figure and Listing
TNF	Tumor Necrosis Factor
ULoQ	Upper Limit of Quantification
VOC	Variant of Concern
WHO	World Health Organization
WOCBP	Woman of Childbearing Potential

1 INTRODUCTION

This Statistical Analysis Plan (SAP) describes the statistical analysis methods planned for Protocol UB-612-305: A Phase 3 Multi-Center International, Randomized, Active-Controlled Platform Trial to Compare Homologous Boost of Authorized COVID-19 Vaccines and Heterologous Boost with UB-612 Vaccine. The SAP is for the platform trial and will apply to each comparator sub-study unless specified otherwise.

The analyses will be performed under the following timepoints: 1) the primary analyses when the last subject has reached Day 56, which includes analyses of the immunogenicity endpoints assessed at Day 29, and 2) the end of study analyses when all subjects reach the end of the study.

Both timepoints will apply to each comparator sub-study, i.e., in the case where there are double-blind and open label sub-studies for the same comparator, the analysis will be performed with both sub-studies combined.

This SAP will be finalized prior to the first sub-study database cutoff for primary analyses and any changes in the plans for analysis in any sub-study will be documented in the sub-study CSR.

Critical analyses (e.g., baseline, primary safety endpoints, and primary and secondary immunogenicity endpoints analyses) will be performed for the CSR. Other analysis (e.g., exploratory endpoints and subgroup analyses) will not be included in CSR unless there are findings.

The SAP will not be updated in case of administrative changes or amendments to the protocol unless the changes impact the analyses.

1.1 Study Objectives

Table 1: Objectives and Endpoints

**PRIMARY**	
**Primary Safety Objective**	**Primary Safety Endpoints**
To evaluate the safety and tolerability profiles of UB-612 and a comparator vaccine in subjects who have received primary immunization[1] with a COVID- 19 vaccine at least 3 months or more before the study start.	• Injection site adverse events (AEs) for up to 7 days following receipt of the study vaccine<br>• Prompted systemic AEs for up to 7 days following receipt of the study vaccine<br>• Unsolicited AEs following receipt of the study vaccine to 28 days after the dose<br>• Serious adverse events (SAEs), medically attended adverse events (MAAEs) and adverse events of special interest (AESIs) from receipt of the study vaccine to 12 months after the dose
**Primary Immunogenicity Objective**	**Primary Immunogenicity Endpoints**
To compare UB-612 vaccine to a comparator vaccine in the ability to boost short-term neutralizing antibody immunity against SARS-CoV-2, Wuhan strain, in	SARS-CoV-2 neutralizing antibody titers measured in Vero cells using replicating virus (prototype Wuhan strain)

subjects with no known history of natural infection who have received primary immunization[1] with the same comparator vaccine at least 3 months or more before the day of immunization	• Neutralizing antibody geometric mean titer ratio (GMR) at Day 29 post-boost
**SECONDARY**	
**Secondary Immunogenicity Objectives**	**Secondary Immunogenicity Endpoints**
• To compare UB-612 vaccine to a comparator vaccine in the ability to boost short-term neutralizing antibody immunity to the SARS-CoV-2, Omicron variant of concern (VOC)	SARS-CoV-2 neutralizing antibody titers measured in Vero cells using replicating virus Omicron variant<br>• Neutralizing antibody geometric mean titer ratio (GMR) at Day 29 post-boost
• To evaluate the kinetics and duration of humoral immunity to the SARS-CoV-2 Wuhan strain and Omicron variant after UB-612 or the comparator vaccine boosting dose	SARS-CoV-2 neutralizing antibody titers measured in Vero cells using replicating virus Wuhan and Omicron variant<br>• Area under the curve (AUC) of neutralizing antibody response by treatment group and virus variants from Day 15 to Month 12<br>• Neutralizing antibody geometric mean titer (GMT) on Days 15, 29, and Months 6 and 12 post-boost<br>• Geometric mean fold increase (GMFI) in neutralizing antibodies titers from before study product injection on Day 1 to Day 15, Day 29, and Months 6 and 12 post-boost<br>• Proportion of subjects with ≥4-fold rise of neutralizing antibodies from before study product injection to Days 15 and 29, and Months 6 and 12 post-boost<br>• Distribution of neutralizing antibody titers determined on Day 29 and Month 6 and 12 post-boost, displayed as reverse cumulative distribution curves by the treatment group and virus variant<br><br>SARS-CoV-2 immunoglobulin G (IgG) antibody titers measured by direct S1-RBD binding ELISA<br>• IgG antibody GMT on Days 15, 29, and Months 6 and 12 post-boost<br>• GMFI in of IgG antibodies titers from before study product injection on Day 1 to Day 15 and Day 29, and Months 6 and 12 post-boost<br>• Proportion of subjects with ≥4-fold rise of IgG antibodies from before study product injection to Days 15 and 29, and Months 6 and 12 post-boost<br>• Distribution of IgG antibody titers determined on Day 29 and Months 6 and 12 post-boost, displayed as reverse cumulative distribution curves by the treatment group and virus variant

	• Area under the curve (AUC) of IgG antibody response by the treatment group and the virus variant from Day 15 to Month 12 post-boost
**EXPLORATORY**	
**Exploratory Objectives**	**Exploratory Endpoints**
To evaluate the ability of UB-612 and a comparator vaccine to boost cellular immunity in a subset of subjects	The number of cytokine secreting spots per million cells and % cells staining for cytokines at Days 1, 15 and 29, and Months 6 and 12 post-boost<br>• Interferon-gamma (IFN-γ) and interleukin (IL)-4 secreting cells/10^6 cells in enzyme-linked immunosorbent spot (ELISpot) assays and intracellular cytokine staining (ICS) for IFN-γ, IL-2, IL-4, IL-10, tumor necrosis factor-alpha (TNF-α), and granzyme B measured against the protein and pooled peptide components separately
To compare the ability of UB-612 vaccine or the comparator vaccine to boost humoral immunity in a subset of subjects	• Fc mediated (non-neutralizing) functional antibody responses at baseline and Day 29 post-boost<br>− ADCP (antibody dependent cell-mediated phagocytosis)<br>• SARS-CoV-2 neutralizing antibody titers measured in Vero cells using replicating additional variant live viruses<br>− GMT, GMR, GMFI, and seroresponse rates based on neutralizing titers determined at Day 1 (baseline) and Day 29 post-boost

[1] Primary immunization is defined as 2 doses spaced approximately 3-17 month apart. The time interval to booster is 3 months or more, taking into consideration the local and national regulations, is be described in detail in a relevant sub-study section linked to the master protocol.

1.2 Study Design

This is a multicenter, international, randomized, active-controlled platform study to evaluate the ability of UB-612 vaccine to boost immunity in subjects 16 years of age and older who previously received a COVID-19 vaccine primary series. In each comparative sub-study, eligible subjects will be randomized in 1:1 ratio to receive either UB-612 or a comparator vaccine booster as shown in Figure 1. The randomization will be stratified by the following factors: age group (16-64 vs ≥65 years old), gender at birth (male vs female), baseline N-protein seropositivity (positive vs. negative), and the time since the last primary immunization dose (≥3-<5 months, vs. ≥5 months). Refer to the study's Randomization Materials Specifications document for randomization details. Data from subjects who receive an authorized COVID-19 vaccine will be compared with data from subjects who receive UB-612 vaccine as a booster vaccine.

Figure 1: Trial Design Flow Chart

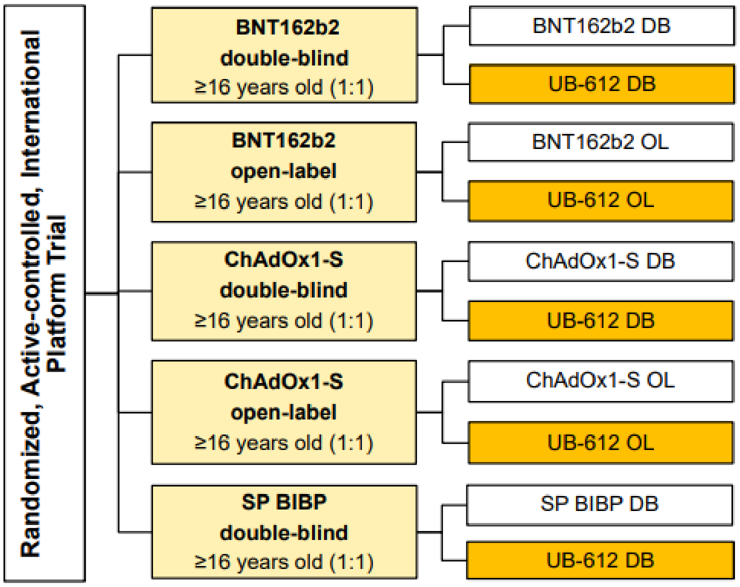

A double-blinded or an open label sub-study is designed for the BNT162b2 and ChAdOx1-S comparator vaccines. With double blinded studies being a priority, open-label sub-studies enable recruitment at participating sites that otherwise do not have capabilities to administer comparators vaccines in a double-blinded fashion. All subjects will be assessed for safety and immunogenicity endpoints after a single booster immunization with UB-612 or a comparator vaccine.

2 STATISTICAL HYPOTHESES

In each comparative sub-study, the hypotheses for the non-inferiority of the UB-612 vaccine to the comparator vaccine in the ability to boost short-term neutralizing antibody immunity against SARS-CoV-2, Wuhan strain will be

Primary hypotheses

- Null Hypothesis $H_{0,1}$: The neutralizing antibody GMT of UB-612 is unacceptably worse than the neutralizing antibody GMT of the comparator at Day 29 post-boost, vs.

- Alternative Hypothesis $H_{a,1}$: The neutralizing antibody GMT of UB-612 is not unacceptably worse than the neutralizing antibody GMT of the comparator at Day 29 post-boost.

$H_{0,1}$ will be rejected if the lower 95% confidence limit of the GMR of UB-612 to comparator is >0.67. The study will be declared successful if $H_{0,1}$ is rejected.

If the primary null hypothesis is rejected, the following superiority hypothesis will be further tested:

- Null Hypothesis $H_{0,2}$: The neutralizing antibody GMT of UB-612 is ≤ the neutralizing antibody GMT of the comparator at Day 29 post-boost, vs.

- Alternative Hypothesis $H_{a,2}$: The neutralizing antibody GMT of UB-612 is > the neutralizing antibody GMT of the comparator at Day 29 post-boost.

$H_{0,2}$ will be rejected if the lower 95% confidence limit of the GMR of UB-612 to comparator is >1.0.

Secondary hypotheses

To the SARS-CoV-2 Omicron strain

- Null Hypothesis $H_{0,3}$: The neutralizing antibody GMT of UB-612 is unacceptably worse than the neutralizing antibody GMT of the comparator at Day 29 post-boost, vs.

- Alternative Hypothesis $H_{a,3}$: The neutralizing antibody GMT of UB-612 is not unacceptably worse than the neutralizing antibody GMT of the comparator at Day 29 post-boost.

$H_{0,3}$ will be rejected if the lower 95% confidence limit of the GMR of UB-612 to comparator is > 0.67.

If the null hypothesis $H_{0,3}$ is rejected, the following superiority hypothesis will be further tested:

- Null Hypothesis $H_{0,4}$: The neutralizing antibody GMT of UB-612 is ≤ the neutralizing antibody GMT of the comparator at Day 29 post-boost, vs.

- Alternative Hypothesis $H_{a,4}$: The neutralizing antibody GMT of UB-612 is > the neutralizing antibody GMT of the comparator at Day 29 post-boost.

$H_{0,4}$ will be rejected if the lower 95% confidence limit of the GMR of UB-612 to comparator is >1.0.

To the SARS-CoV-2 Wuhan strain

- Null Hypothesis $H_{0,5}$: The seroresponse rate of UB-612 is unacceptably worse than the seroresponse rate of the comparator at Day 29 post-boost, vs.

- Alternative Hypothesis $H_{a,5}$: The seroresponse rate of UB-612 is not unacceptably worse than the seroresponse rate of the comparator at Day 29 post-boost.

$H_{0,5}$ will be rejected if the lower 95% confidence limit of the difference in seroresponse rates between UB-612 and the comparator is > -10%.

To the SARS-CoV-2 Omicron strain

- Null Hypothesis $H_{0,6}$: The seroresponse rate of UB-612 is unacceptably worse than the seroresponse rate of the comparator at Day 29 post-boost, vs.

- Alternative Hypothesis $H_{a,6}$: The seroresponse rate of UB-612 is not unacceptably worse than the seroresponse rate of the comparator at Day 29 post-boost.

$H_{0,6}$ will be rejected if the lower 95% confidence limit of the difference in seroresponse rates between UB-612 and the comparator is > -10%.

Similar superiority hypothesis tests will be performed if the corresponding non-inferiority secondary null hypotheses are rejected, where the superiority null hypotheses based on seroresponse rates will be rejected if the lower 95% confidence limit of the difference in seroresponse rates between UB-612 and the comparator is > 0%.

2.1 Multiplicity Adjustment

All hypotheses will be tested to establish conclusions independently for the primary analysis for each sub-study; therefore, no statistical adjustment for multiplicity within or across sub-studies will be applied.

3 ANALYSIS SETS

The following populations will be used for the analyses.

- Intent to Treat (ITT) Population: All eligible subjects who are randomized to the study treatment. This population will be used for the summaries of subject disposition and baseline characteristics.

- Modified ITT (mITT) Population: All ITT subjects who receive study vaccine, have baseline and Day 29 immunogenicity antibody titer value, with no other major protocol deviations which would directly impact assessments of immunogenicity. This population will be used for the analyses of immunogenicity and will be analyzed according to the vaccine received.

- Safety Population: All subjects who received study vaccine. This population will be used for all analyses of safety endpoints and will be analyzed according to the vaccine received.

Major protocol deviations which would directly impact assessments of immunogenicity are described in Appendix 1. Details of the protocol deviations assessment process are in the study's Protocol Deviation Handling Plan.

4 STATISTICAL ANALYSES

4.1 General Consideration

The analyses described below will be conducted by comparator sub-study. In the case where there are double-blind and open label sub-studies for the same comparator, the analysis will be performed with both sub-studies combined. All analyses will be conducted using observed data only and no missing data will be imputed. Programming guidelines are on file with additional data handling rules and conventions.

4.2 Primary Immunogenicity Endpoints Analyses

The primary immunogenicity endpoint, based on SARS-CoV-2 neutralizing antibody titers measured using replicating or pseudotyped virus (prototype Wuhan strain), is:

- GMT at Day 29 post-boost

4.2.1 Geometric Mean Titer (GMT)

The GMT of SARS-CoV-2 antibody is calculated by 10 to the power of mean values of log-transformed titers with base 10, i.e.,

Day 29 GMT = 10^(mean values of log10 titers).

GMR of SARS-CoV-2 antibody between UB-612 and a comparator at Day 29 is defined as the ratio of GMTs in the UB-612 group and in the comparator group.

GMR = GMT for UB-612 / GMT for a comparator.

4.2.2 Main Analytical Approach

An analysis of covariance (ANCOVA) model will be run with dependent variable of log-transformed (base 10) titer values at Day 29, and with factors of treatment, age group (16-64 vs. ≥65 years old), gender at birth (male vs female), the time since the last primary immunization dose (≥3-<5 months, vs. ≥5 months), baseline N-protein seropositivity (positive, negative) and baseline log titers as covariates. The estimates of the GMTs and the GMR and their CIs will be the antilogs (base 10) of the least square means (LSM) of log antibody titer and their CIs for each treatment and the treatment difference, respectively. The GMR point estimate and 95% confidence interval (CI) will be provided. Noninferiority (superiority) will be established when the lower bound of the 95% CI is >0.67 (>1). Where both double-blinded and open-label sub-studies are performed with the same comparator, the analyses will be based on the pooled data from both sub-studies and the sub-study type will be added as a stratum factor in the analysis to reduce the variability.

A sensitivity analysis will be performed using baseline N-protein seropositivity results from the ELISA test in place of results from the N-protein rapid test used for stratification.

4.3 Secondary Immunogenicity Endpoints Analyses

4.3.1 Secondary Endpoints

The secondary immunogenicity endpoints will be based on three types of immunoassays, including SARS-CoV-2 neutralizing antibody titers measured using replicating or pseudotyped virus Wuhan Strain and Omicron variants, and SARs-CoV-2 IgG antibody titers measured by direct S1-RBD binding ELISA. Specifically, the secondary immunogenicity endpoints are below.

SARS-CoV-2 neutralizing antibody titers measured using replicating or pseudotyped virus Omicron variant

- GMT at Day 29 post-boost

SARS-CoV-2 neutralizing antibody titers measured using replicating or pseudotyped virus Wuhan variant

- Seroresponse rate at Day 29 post-boost. Numbers and proportions of subjects with a ≥4-fold rise from before study vaccine injection to Day 29 post-boost (seroresponse rate).

SARS-CoV-2 neutralizing antibody titers measured using replicating or pseudotyped virus Omicron variant

- Seroresponse rate at Day 29 post-boost. Numbers and proportions of subjects with a ≥4-fold rise from before study vaccine injection to Day 29 post-boost (seroresponse rate)

SARS-CoV-2 neutralizing antibody titers measured using replicating or pseudotyped virus Wuhan and Omicron variants.

- AUCs of neutralizing antibody response from Day 15 to Month 12.
- GMTs on Days 15, 29, and Months 6 and 12 post-boost.
- GMFI in neutralizing antibody titers from before study vaccine injection on Day 1 to Day 15, Day 29, and Months 6 and 12 post-boost.
- Seroresponse rates at Day 15, Day 29, and Months 6 and 12 post-boost.
- Distribution of neutralizing antibody titers determined on Day 29, Months 6 and 12 post-boost, displayed as reverse cumulative distribution curves by treatment group and virus variant.

SARs-CoV-2 IgG antibody titers measured by direct S1-RBD binding ELISA.

- IgG antibody GMTs on Days 15, 29 and Months 6 and 12 post-boost.

- GMFI of IgG antibody from before study product injection on Day 1 to Day 15, Day 29, and Months 6 and 12 post-boost.

- Seroresponse rates based on IgG antibodies from before study vaccine injection to Day 15, Day 29, Months 6 and 12 post-boost.

- Distribution of IgG antibody titers determined on Day 29, Months 6 and 12 post-boost, displayed as reverse cumulative distribution curves by treatment group and virus variant.

- AUCs of IgG antibody response by the treatment group and virus variant from Day 15 to Month 12 post-boost.

For each type of immunoassay, GMTs will be summarized descriptively by treatment group at each scheduled visit, along with 95% CIs. GMRs will be calculated for all scheduled visits as the ratio of GMTs for UB-612 and the comparator. Seroresponse rates will be calculated for all post-baseline visits along with 95% CIs. In addition, the difference in the seroresponse rates between UB-612 and the comparator will be calculated and the 95% CIs based on normal approximation will be displayed. The GMFI will be calculated for all post-baseline visits along with 95% CIs, where GMFI is defined as the geometric mean of the ratio of the post-vaccination titer value to the pre-vaccination titer value.

The AUC between Day 15 and Month 12 post-boost for each immunoassay will be calculated for each subject based on observed data using trapezoidal method, where linear trapezoidal method will be used when the antibody titers increase or are equal between scheduled visits and linear-log trapezoidal method will be used when the antibody titers decrease between scheduled visits. Summary statistics will be calculated once individual AUCs are obtained.

4.4 Exploratory Immunogenicity Endpoints and Analyses

Each sub-study will select a subset of approximately 10% of subjects, in proportion to the study size, to provide PBMC samples and serum samples for Fc-mediated assays and neutralization against additional SARS-CoV-2 variants.

- Exploratory immunogenicity endpoints and analysis methods are described below. The number of cytokine secreting spots per million cells and % cells staining for cytokines at Days 1, 15 and 29, and Months 6 and 12 post-boost, including IFN-γ and IL-4 secreting cells/10^6 cells in ELISpot assays and ICS for IFN- γ, IL-2, IL-4, IL-10, TNF-α, and granzyme B measured against the protein and pooled peptide components separately.

- Descriptive statistics will be provided for each parameter by study visit and treatment group.

- Fc mediated (non-neutralizing) functional antibody responses at baseline and Day 29 post-boost in terms of ADCP.

- GMTs and GMRs at baseline and Day 29 post-boost will be calculated. GMFI at Day 29 will be calculated within each treatment group. All relevant statistics will be calculated using similar methods described in Sections 10.1 and 10.2.

- GMT, GMR, GMFI and seroresponse rate based on SARS-CoV-2 neutralizing antibody titers measured using replicating additional variant live or pseudotyped viruses at Day 1 and Day 29 post-boost when appliable.

Once additional variant live viruses are identified, GMT, GMR, GMFI, seroresponse rate and difference in seroresponse rate will be calculated using methods described in Section 4.3.1.

4.5 Pooling Efficacy Data across Sub-studies

If two or more sub-study immunogenicity results indicate they can be pooled, additional analyses will be performed in the pooled sub-studies data as supportive analyses.

4.6 Safety Analyses

Safety endpoints will be analyzed descriptively by treatment per sub-study as well as with UB-612 arms pooled cross all sub studies.

4.6.1 Extent of Exposure

A frequency table of exposure (study vaccine completion and injection site) will be summarized.

4.6.2 Adverse Events

Adverse events (AEs) will be codded using MedDRA. The actual version of MedDRA will be noted in the statistical tables and clinical study report. The intensity of the AEs is graded according to the Common Terminology Criteria for Adverse Events (CTCAE), version 5.0

An AE will be considered a treatment-emergent adverse event (TEAE) if the AE began or worsened (increased in severity or became serious) after the administration of study drug.

A treatment-emergent serious adverse event (TESAE) is defined as an SAE that is also a TEAE. SAEs are defined in protocol Section 11.3.1.5.

Solicited local and systemic AEs are defined in protocol Section 11.2.10. AESIs (Adverse Events of Special Interest) are defined in protocol Section 11.3.1.4 and Protocol Appendix 7. MAAEs (Medically Attended Adverse Event) are defined in protocol Section 11.3.1.3.

TEAEs will be summarized by subject incidence rates. The number and percentage of subjects with TEAEs will be tabulated for overall (i.e., any preferred term), primary MedDRA SOC and PT.

4.6.2.1 Primary Safety Endpoints

Solicited local and systemic reactions after study product injection will be presented by severity and cumulatively across severity levels. Descriptive summary statistics will include counts and

percentages of subjects with the indicated endpoint and the associated 95% confidence intervals. Summaries will be presented by treatment group within each previous vaccination group.

Unsolicited AEs through 29 days after study product injection, and MAAEs, SAEs, and AESIs through 12 months after study product injection) will be summarized descriptively by pre-study treatment (i.e., previous vaccine), study treatment, age and gender group, MedDRA SOC and PT.

4.6.2.2 Treatment-Emergent Adverse Events

Summaries will be done for the below categories of AEs.

- Overall summary of TEAEs
- TEAEs by SOC and PT
- TEAEs by SOC, PT and maximum severity
- TEAEs by SOC, PT and maximum relationship
- Treatment-related TEAEs by SOC and PT
- TEAEs leading to study discontinuation by SOC and PT

4.6.2.3 Exploratory Analysis for Solicited Adverse Events

All analyses described in this section will be based on pooled data in the Safety Set from all sub-studies.

An additional exploratory analysis that subdivides participants into those who are seropositive vs seronegative at baseline may be performed for solicited local and systemic reactions, depending the counts of subjects in each group. Also, the analysis of blinding impact may be performed on subjects reporting safety after a common IP injection with the data collected in a blinded and open-label sub-studies.

4.6.2.4 Exploratory Analysis of Standardised MedDRA Queries (SMQs) Relating to Myocarditis or Pericarditis

The following SMQs will be summarized descriptively by sub-SMQs, PTs and study treatment: Noninfectious myocarditis/pericarditis, Ischemic heart disease, Cardiac arrhythmias, Cardiac failure, and Cardiomyopathy.

4.6.2.5 Laboratory Data

Laboratory assessments are detailed in protocol Section 11.2.7 and Table 1.

4.6.2.5.1 Hematology and Chemistry Parameters

Clinical laboratory values will be reported in conventional International System of Units (SI) units and categorized as normal/abnormal based on normal ranges collected from local laboratories. Additionally, particular laboratory abnormalities are graded according to Protocol Appendix 6: LABORATORY ABNORMALITY GRADING SCALE. Since parameters were tested at local

labs and are not comparable across labs, analyses will be restricted to abnormalities and grade shifts.

The number and percentage of subjects who experienced laboratory test abnormalities will be summarized according to worst toxicity grade observed for each lab assay.

For graded parameters, shift tables in laboratory toxicity grades will be tabulated by shift from baseline to the worst post baseline grade over all visits (scheduled and unscheduled) presenting counts and percentages of subjects.

For parameters where there is no toxicity grade defined, shifts from baseline (low, normal, high) to at least one result above normal or at least one result below normal will be presented.

4.6.2.5.2 Other Laboratory Parameters

Quantitative data for all other laboratory parameters (e.g., hs-CRP) will be presented in a similar way to chemistry and hematology parameters.

4.6.3 Other Safety Parameters

4.6.3.1 Vital Signs

Descriptive statistics for semi-recumbent vital signs including heart rate (HR), systolic and diastolic blood pressure (BP), respiratory rate (RR), oral temperature at each visit will be presented.

4.6.3.2 Height and Weight

Descriptive summaries will be presented for height, weight and derived BMI.

4.6.3.3 Physical and Neurological Examination

Results will be summarized or listed.

4.6.3.4 Electrocardiogram (ECG)

The number and percentage of subjects with abnormalities will be summarized. Shift tables will be tabulated.

4.6.3.5 Pregnancy Test

Positive results will be listed.

4.6.3.6 Antigen Test

Positive results will be listed.

4.7 Other Analyses

4.7.1 Study Subjects, Demographics and other Baseline Characteristics

4.7.1.1 Disposition of Subjects

The total number of subjects will be summarized for below.

- Subjects who were screened (who signed informed consent)

Number and percentage of subjects in each of the following category will be summarized.

- Subjects who were randomized
- Subjects who were treated
- Subjects who withdrew early from the study
    - Reasons for study discontinuation

The number of subjects in each analysis population will be summarized. Subjects excluded from any analysis population will be listed.

4.7.1.2 Protocol Deviation

Major protocol deviations will be summarized.

4.7.1.3 Demographics and Other Baseline Characteristics

Demographics parameters (age, age group, gender, race, ethnicity) will be summarized descriptively.

Baseline characteristics as listed below will be summarized.

- Height, Weight, BMI
- Baseline N-protein seropositivity (positive, negative)
- Time since the last primary immunization dose (≥3 - <5 months, ≥5 months)
- Neutralizing antibody titer, Wuhan strain
- Neutralizing antibody titer, Omicron variant
- IgG antibody
- Neutralizing antibody titer, other variants if assessed

4.7.1.4 Medical History

The number and percentage of patients with medical history will be summarized by SOC and PT. Patients reporting more than one condition/diagnosis will be counted only once in each row (overall, SOC or PT).

In particular, the co-morbidities associated with COVID-19 will be summarized using a frequency table by treatment group and overall based on ITT Population.

4.7.1.5 Prior and Concomitant Medications or Procedures

Prior medication is defined as a medication with a stopping date prior to the dosing date. Concomitant medication is defined as a medication started on or after dosing date or started prior to dosing date but continued post dosing date.

Prior and concomitant medications will be coded using World Health Organization (WHO) Drug Dictionary. The actual version of WHO will be noted in the statistical tables and clinical study report. The number and percentage of subjects reporting prior or concomitant medications will be summarized separately by Anatomical Therapeutic Chemical (ATC) 4th level (or most specific level available if 4th level is unavailable) class and preferred drug name. If more than one medication is coded to the same preferred drug name for the same subject, the subject will be counted only once for that preferred drug name.

Concomitant procedures will be coded using MedDRA and summarized by SOC and PT.

4.7.2 Subgroup Analysis

Subgroup analysis will be performed for selected safety and immunogenicity endpoints in the analyses pooled across sub-studies if there are at least 5 subjects in each group based on, but not limited to:

- Age group (16-64, ≥65 years old)
- Gender at birth (Female, Male)
- Baseline N-protein seropositivity (positive, negative)
- Time since the last primary immunization dose (≥3 - <5 months, ≥5 months)
- Immunogenicity only: BMI group (< 30 kg/m^2, ≥30 kg/m^2)
- Safety only: subjects who got additional comparator vaccinations within the study period

4.8 Interim Analysis

There is no interim analysis planned for this study.

4.9 Changes to Protocol-planned Analyses

Not applicable.

5 SAMPLE SIZE DETERMINATION

The sample size was determined by targeting at least 90% power (BNT162b2 Sub-studies and BIBP Sub-study) and 80 to 85% power (ChAdOx1-S Sub-studies) with 1-sided alpha of 0.025 to test non-inferiority based on the geometric mean titer (GMT) ratio using a margin ratio of GMT of 0.67 between UB-612 and each comparator vaccine.

5.1 BNT162b2 Sub-study

For the primary endpoint of GMT, assuming a log10 standard deviation of 0.38 and a difference in log10 GMT of -0.038 (equivalent to a GMT ratio of 0.92), a sample size of 320 (160 per arm) has 90% power to establish non-inferiority, with a non-inferiority margin on the ratio of 0.67. A total of 400 subjects was required from both double-blinded and open label sub-studies enrolled to account for participants who are excluded from the analysis population (mITT) during the study.

5.2 ChAdOx1-S Sub-study

For the primary endpoint of GMT, assuming a log10 standard deviation of 0.40 (based on neutralizing antibodies vs alpha strain [Flaxman et al., 2021]), the achievable sample size in these sub-studies is approximately 190. This sample size will have 80 to 85% power to establish non-inferiority, with a non-inferiority margin on the ratio of 0.67 if no subjects are lost prior to the assessment of the primary endpoint at Day 29.

5.3 BIBP Sub-study

For the primary endpoint of GMT, assuming a log10 standard deviation of 0.44 (based on 6 µg group [Kanokudom et al, 2021]), a sample size of 266 (133 per arm) has 90% power to establish non-inferiority, with a non-inferiority margin on the ratio of 0.67. A total of 334 subjects was enrolled to account for participants who are excluded from the analysis population (mITT) during the study.

6 REFERENCES

Flaxman A, Marchevsky NG, Jenkin D, Aboagye J, Aley PK, Angus B, Belij-Rammerstorfer S, Bibi S, Bittaye M, Cappuccini F, Cicconi P, Clutterbuck EA, Davies S, Dejnirattisai W, Dold C, Ewer KJ, Folegatti PM, Fowler J, Hill AVS, Kerridge S, Minassian AM, Mongkolsapaya J, Mujadidi YF, Plested E, Ramasamy MN, Robinson H, Sanders H, Sheehan E, Smith H, Snape MD, Song R, Woods D, Screaton G, Gilbert SC, Voysey M, Pollard AJ, Lambe T; Oxford COVID Vaccine Trial group. Reactogenicity and immunogenicity after a late second dose or a third dose of ChAdOx1 nCoV-19 in the UK: a substudy of two randomised controlled trials (COV001 and COV002). Lancet. 2021 Sep 11;398(10304):981-990. doi: 10.1016/S0140-6736(21)01699-8. Epub 2021 Sep 1. PMID: 34480858; PMCID: PMC8409975.

Kanokudom S, Assawakosri S, Suntronwong N, Auphimai C, Nilyanimit P, Vichaiwattana P, Thongmee T, Yorsaeng R, Srimuan D, Thatsanatorn T, Klinfueng S, Sudhinaraset N, Wanlapakorn N, Honsawek S, Poovorawan Y. Safety and Immunogenicity of the Third Booster Dose with Inactivated, Viral Vector, and mRNA COVID-19 Vaccines in Fully Immunized Healthy Adults with Inactivated Vaccine. Vaccines (Basel). 2022 Jan 6;10(1):86. doi: 10.3390/vaccines10010086. PMID: 35062747; PMCID: PMC8779615.

7 VERSION HISTORY

SAP Version	Approval Date	Change	Rationale
1.0	26 Aug 2022	Not Applicable	Original version
2.0	17 Oct 2022	Change to align with protocol version 10.0 (no interim analysis (Section 4.8) and sample size/power in ChAdOx1-S sub-study (sections 5.0 and 5.2).	Protocol amendment
		Add protocol deviation potentially affecting immunogenicity to Appendix 1.	The criterion (having grade 2 or more abnormalities of specific labs) identifies conditions with the potential to impact the interpretation of immunogenicity.
		Added clarification that the linear trapezoidal method will be used for AUC also when titers are equal across visits (Section 4.3).	What will be done for equal titers was not clear.
		The logistic regression planned for exploratory analysis of solicited AEs has been removed from Section 4.6.2.3.	It is observed that solicited AEs by type are sparse with few to none with severity of 3 or 4. The planned analysis is no longer feasible.
		BMI group added as a subgroup to explore the effect of BMI on primary immunogenicity outcomes (Section 4.7.2).	BMI has been identified as a potential variable affecting immunogenicity because of its association with inflammation.
		Referenced other study plans for more details on randomization, protocol deviations assessments, and data handling rules.	Provide source of more details surrounding the data used for analysis.
		Clarified that immunogenicity and safety analyses would be grouped according to treatment received.	This information was not made clear in the protocol.

APPENDIX 1: MAJOR PROTOCOL DEVIATIONS POTENTIALLY IMPACTING IMMUNOGENICITY

Major protocol deviations which would directly impact assessments of immunogenicity are:

1. Not receiving booster or receiving the wrong booster.
2. Missing baseline or Day 29 immunogenicity visit/assessment/results.
3. Day 29 immunogenicity results outside a 10-day window of nominal Day 29.
4. Subject is missing 1 dose of 2-dose primary, has mixed primary dose (except Sinopharm and Sinovac), or already received booster dose. (Inclusion criterion #2)
5. Known history of COVID-19 or SARS-CoV-2 infection within six (6) months prior to Day 1 (Exclusion criterion #1)
6. Subject has positive SARS-CoV-2 reverse transcriptase-polymerase chain reaction (RT-PCR) or antigen test within 24-48 hours prior to receipt of injections on Day 1 (active infection). (Inclusion criterion #4)
7. Subject has positive SARS-CoV-2 reverse transcriptase-polymerase chain reaction (RT-PCR) or antigen test after randomization and through Day 29 (active infection).
8. Subject has chronic kidney disease with dialysis. (Exclusion criterion #8)
9. Subject received systemic corticosteroids (≥0.5 mg/kg per day of prednisone or equivalent) for ≥7 days from 28 days before enrollment through conclusion of the study. (Exclusion criterion #9)
10. Subject received any cytotoxic or immunosuppressive drug within six (6) months before Day 1 visit. (Exclusion criterion #10)
11. Subject received any cytotoxic or immunosuppressive drug prior to Day 29 visit.
12. Subject received or plans to receive a live attenuated vaccine or licensed adjuvanted (non-aluminum compound) vaccination within 28 days before or after planned administration of study vaccine (Day 1) or another type of vaccine (including influenza vaccine) within 14 days before or after planned administration of study vaccine on Day 1 visit. (Exclusion criterion #12). Same for Day 29.
13. Subject is human immunodeficiency virus (HIV) or hepatitis B surface antigen (HBsAg) positive; hepatitis C virus (HCV) antibody positive subjects may be tested for RNA and if negative may be enrolled. (Exclusion criterion #13)

14. Any Grade 2 or greater clinical or laboratory abnormalities at screening. Specifically Grade 2 or higher abnormalities for hematology (hemoglobin, WBC, lymphocytes, neutrophils, eosinophils, and platelets) as well as the inflammation (hsCRP) labs per protocol Appendix 6.

15. Subject is in an immunocompromised state (weakened immune system) from solid organ transplant, immunosuppressive or immunodeficient state, autoimmune diseases, asplenia and, recurrent severe infections. (Exclusion criterion #15)

16. Subject has an active malignancy or history of metastatic or hematologic malignancy except non-melanoma skin cancers. (Exclusion criterion #16)

17. Subject was administered immunoglobulins and/or any blood products within the 120 days preceding Day 1 or planned administration during the study period. (Exclusion criterion #18)

DocuSign

Certificate Of Completion

Envelope Id: 0F2FF7960BFB497BBE610ACFECE52E6E

Status: Completed

Subject: Complete with DocuSign: Vaxxinity UB-612-305 SAP 2.0 20221017.docx

Source Envelope:

Document Pages: 26

Signatures: 4

Certificate Pages: 5

Initials: 0

AutoNav: Enabled

EnvelopeId Stamping: Disabled

Time Zone: (UTC-05:00) Eastern Time (US & Canada)

Envelope Originator:
Severina Hutchinson
2622 Commerce St
Dallas, TX 75226
severina@vaxxinity.com
IP Address: 68.237.58.225

Record Tracking

Status: Original
10/17/2022 9:11:18 PM

Holder: Severina Hutchinson
severina@vaxxinity.com

Location: DocuSign

Signer Events | Signature | Timestamp

Lixia Wang
lixia@vaxxinity.com
SVP, Data Science
Vaxxinity
Security Level: Email, Account Authentication (Required)

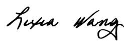

Signature Adoption: Pre-selected Style
Signature ID:
775AC1C8-E144-45F7-B7EE-30A3840209DC
Using IP Address: 73.254.106.22

With Signing Authentication via DocuSign password
With Signing Reasons (on each tab):
I approve this document

Sent: 10/17/2022 9:12:53 PM
Viewed: 10/19/2022 12:39:03 PM
Signed: 10/19/2022 12:39:40 PM

Electronic Record and Signature Disclosure:
Accepted: 7/11/2022 5:53:25 PM
ID: 8687599f-392d-476b-b3f9-2e2c5c238178

Sasha Rumyantsev
sasha@vaxxinity.com
TA Head, Infectious Diseases
Security Level: Email, Account Authentication (Required)

DocuSigned by:

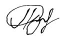

Signer Name: Sasha Rumyantsev
Signing Reason: I have reviewed this document
Signing Time: 18-Oct-2022 | 06:04:29 PDT
2F2F93A4639B47649C8AA13A68E12FD0

Signature Adoption: Drawn on Device
Signature ID:
2F2F93A4-639B-4764-9C8A-A13A68E12FD0
Using IP Address: 209.6.225.85

With Signing Authentication via DocuSign password
With Signing Reasons (on each tab):
I have reviewed this document

Sent: 10/17/2022 9:12:54 PM
Viewed: 10/18/2022 9:04:06 AM
Signed: 10/18/2022 9:04:36 AM

Electronic Record and Signature Disclosure:
Accepted: 10/18/2022 9:04:06 AM
ID: ff866c80-fbed-4569-aec5-eb2907461f5f

Signer Events	Signature	Timestamp
Tanya Granston<br>tanya@vaxxinity.com<br>Senior Director, Biostatistics<br>Security Level: Email, Account Authentication (Required)	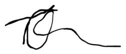<br>Signature Adoption: Drawn on Device<br>Signature ID:<br>CB1FC9DB-0B95-4BD3-824B-61B37B73BCBA<br>Using IP Address: 72.252.37.35<br><br>With Signing Authentication via DocuSign password<br>With Signing Reasons (on each tab):<br>I approve this document	Sent: 10/17/2022 9:12:53 PM<br>Viewed: 10/18/2022 9:03:44 AM<br>Signed: 10/18/2022 9:04:47 AM
**Electronic Record and Signature Disclosure:**<br>Accepted: 8/24/2022 12:44:32 PM<br>ID: 5ac1ce6f-5026-431b-8ab6-7e7e06b5a877		
Ulo Palm<br>ulo@vaxxinity.com<br>CMO<br>Security Level: Email, Account Authentication (Required)	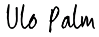<br>Signature Adoption: Pre-selected Style<br>Signature ID:<br>067FEF26-85EB-47D5-B9AB-C58055009D1A<br>Using IP Address: 173.63.97.91<br><br>With Signing Authentication via DocuSign password<br>With Signing Reasons (on each tab):<br>I approve this document	Sent: 10/17/2022 9:12:54 PM<br>Viewed: 10/19/2022 8:32:05 PM<br>Signed: 10/19/2022 8:34:56 PM
**Electronic Record and Signature Disclosure:**<br>Accepted: 7/5/2022 10:10:09 AM<br>ID: 8df92247-60d8-48ca-b790-df03c146db19		

In Person Signer Events	Signature	Timestamp

Editor Delivery Events	Status	Timestamp

Agent Delivery Events	Status	Timestamp

Intermediary Delivery Events	Status	Timestamp

Certified Delivery Events	Status	Timestamp

Carbon Copy Events	Status	Timestamp

Witness Events	Signature	Timestamp

Notary Events	Signature	Timestamp

Envelope Summary Events	Status	Timestamps
Envelope Sent	Hashed/Encrypted	10/17/2022 9:12:55 PM
Certified Delivered	Security Checked	10/19/2022 8:32:05 PM
Signing Complete	Security Checked	10/19/2022 8:34:56 PM
Completed	Security Checked	10/19/2022 8:34:56 PM

Payment Events	Status	Timestamps

Electronic Record and Signature Disclosure

ELECTRONIC RECORD AND SIGNATURE DISCLOSURE

From time to time, Vaxxinity Inc - Part 11 (we, us or Company) may be required by law to provide to you certain written notices or disclosures. Described below are the terms and conditions for providing to you such notices and disclosures electronically through the DocuSign system. Please read the information below carefully and thoroughly, and if you can access this information electronically to your satisfaction and agree to this Electronic Record and Signature Disclosure (ERSD), please confirm your agreement by selecting the check-box next to ‘I agree to use electronic records and signatures’ before clicking ‘CONTINUE’ within the DocuSign system.

Getting paper copies

At any time, you may request from us a paper copy of any record provided or made available electronically to you by us. You will have the ability to download and print documents we send to you through the DocuSign system during and immediately after the signing session and, if you elect to create a DocuSign account, you may access the documents for a limited period of time (usually 30 days) after such documents are first sent to you. After such time, if you wish for us to send you paper copies of any such documents from our office to you, you will be charged a $0.00 per-page fee. You may request delivery of such paper copies from us by following the procedure described below.

Withdrawing your consent

If you decide to receive notices and disclosures from us electronically, you may at any time change your mind and tell us that thereafter you want to receive required notices and disclosures only in paper format. How you must inform us of your decision to receive future notices and disclosure in paper format and withdraw your consent to receive notices and disclosures electronically is described below.

Consequences of changing your mind

If you elect to receive required notices and disclosures only in paper format, it will slow the speed at which we can complete certain steps in transactions with you and delivering services to you because we will need first to send the required notices or disclosures to you in paper format, and then wait until we receive back from you your acknowledgment of your receipt of such paper notices or disclosures. Further, you will no longer be able to use the DocuSign system to receive required notices and consents electronically from us or to sign electronically documents from us.

All notices and disclosures will be sent to you electronically

Unless you tell us otherwise in accordance with the procedures described herein, we will provide electronically to you through the DocuSign system all required notices, disclosures, authorizations, acknowledgements, and other documents that are required to be provided or made available to you during the course of our relationship with you. To reduce the chance of you inadvertently not receiving any notice or disclosure, we prefer to provide all of the required notices and disclosures to you by the same method and to the same address that you have given us. Thus, you can receive all the disclosures and notices electronically or in paper format through the paper mail delivery system. If you do not agree with this process, please let us know as described below. Please also see the paragraph immediately above that describes the consequences of your electing not to receive delivery of the notices and disclosures electronically from us.

How to contact Vaxxinity Inc - Part 11:

You may contact us to let us know of your changes as to how we may contact you electronically, to request paper copies of certain information from us, and to withdraw your prior consent to receive notices and disclosures electronically as follows:
To contact us by email send messages to: it@vaxxinity.com

To advise Vaxxinity Inc - Part 11 of your new email address

To let us know of a change in your email address where we should send notices and disclosures electronically to you, you must send an email message to us at it@vaxxinity.com and in the body of such request you must state: your previous email address, your new email address. We do not require any other information from you to change your email address.

If you created a DocuSign account, you may update it with your new email address through your account preferences.

To request paper copies from Vaxxinity Inc - Part 11

To request delivery from us of paper copies of the notices and disclosures previously provided by us to you electronically, you must send us an email to it@vaxxinity.com and in the body of such request you must state your email address, full name, mailing address, and telephone number. We will bill you for any fees at that time, if any.

To withdraw your consent with Vaxxinity Inc - Part 11

To inform us that you no longer wish to receive future notices and disclosures in electronic format you may:

i. decline to sign a document from within your signing session, and on the subsequent page, select the check-box indicating you wish to withdraw your consent, or you may;

ii. send us an email to it@vaxxinity.com and in the body of such request you must state your email, full name, mailing address, and telephone number. We do not need any other information from you to withdraw consent.. The consequences of your withdrawing consent for online documents will be that transactions may take a longer time to process..

Required hardware and software

The minimum system requirements for using the DocuSign system may change over time. The current system requirements are found here: https://support.docusign.com/guides/signer-guide-signing-system-requirements.

Acknowledging your access and consent to receive and sign documents electronically

To confirm to us that you can access this information electronically, which will be similar to other electronic notices and disclosures that we will provide to you, please confirm that you have read this ERSD, and (i) that you are able to print on paper or electronically save this ERSD for your future reference and access; or (ii) that you are able to email this ERSD to an email address where you will be able to print on paper or save it for your future reference and access. Further, if you consent to receiving notices and disclosures exclusively in electronic format as described herein, then select the check-box next to 'I agree to use electronic records and signatures' before clicking 'CONTINUE' within the DocuSign system.

By selecting the check-box next to 'I agree to use electronic records and signatures', you confirm that:

- You can access and read this Electronic Record and Signature Disclosure; and
- You can print on paper this Electronic Record and Signature Disclosure, or save or send this Electronic Record and Disclosure to a location where you can print it, for future reference and access; and
- Until or unless you notify Vaxxinity Inc - Part 11 as described above, you consent to receive exclusively through electronic means all notices, disclosures, authorizations, acknowledgements, and other documents that are required to be provided or made available to you by Vaxxinity Inc - Part 11 during the course of your relationship with Vaxxinity Inc - Part 11.
